# Effect of radiotherapy after mastectomy and axillary surgery on 10-year recurrence and 20-year breast cancer mortality: meta-analysis of individual patient data for 8135 women in 22 randomised trials

**DOI:** 10.1016/S0140-6736(14)60488-8

**Published:** 2014-06-20

**Authors:** EBCTCG (Early Breast Cancer Trialists' Collaborative Group)

## Abstract

**Background:**

Postmastectomy radiotherapy was shown in previous meta-analyses to reduce the risks of both recurrence and breast cancer mortality in all women with node-positive disease considered together. However, the benefit in women with only one to three positive lymph nodes is uncertain. We aimed to assess the effect of radiotherapy in these women after mastectomy and axillary dissection.

**Methods:**

We did a meta-analysis of individual data for 8135 women randomly assigned to treatment groups during 1964–86 in 22 trials of radiotherapy to the chest wall and regional lymph nodes after mastectomy and axillary surgery versus the same surgery but no radiotherapy. Follow-up lasted 10 years for recurrence and to Jan 1, 2009, for mortality. Analyses were stratified by trial, individual follow-up year, age at entry, and pathological nodal status.

**Findings:**

3786 women had axillary dissection to at least level II and had zero, one to three, or four or more positive nodes. All were in trials in which radiotherapy included the chest wall, supraclavicular or axillary fossa (or both), and internal mammary chain. For 700 women with axillary dissection and no positive nodes, radiotherapy had no significant effect on locoregional recurrence (two-sided significance level [2p]>0·1), overall recurrence (rate ratio [RR], irradiated *vs* not, 1·06, 95% CI 0·76–1·48, 2p>0·1), or breast cancer mortality (RR 1·18, 95% CI 0·89–1·55, 2p>0·1). For 1314 women with axillary dissection and one to three positive nodes, radiotherapy reduced locoregional recurrence (2p<0·00001), overall recurrence (RR 0·68, 95% CI 0·57–0·82, 2p=0·00006), and breast cancer mortality (RR 0·80, 95% CI 0·67–0·95, 2p=0·01). 1133 of these 1314 women were in trials in which systemic therapy (cyclophosphamide, methotrexate, and fluorouracil, or tamoxifen) was given in both trial groups and, for them, radiotherapy again reduced locoregional recurrence (2p<0·00001), overall recurrence (RR 0·67, 95% CI 0·55–0·82, 2p=0·00009), and breast cancer mortality (RR 0·78, 95% CI 0·64–0·94, 2p=0·01). For 1772 women with axillary dissection and four or more positive nodes, radiotherapy reduced locoregional recurrence (2p<0·00001), overall recurrence (RR 0·79, 95% CI 0·69–0·90, 2p=0·0003), and breast cancer mortality (RR 0·87, 95% CI 0·77–0·99, 2p=0·04).

**Interpretation:**

After mastectomy and axillary dissection, radiotherapy reduced both recurrence and breast cancer mortality in the women with one to three positive lymph nodes in these trials even when systemic therapy was given. For today's women, who in many countries are at lower risk of recurrence, absolute gains might be smaller but proportional gains might be larger because of more effective radiotherapy.

**Funding:**

Cancer Research UK, British Heart Foundation, UK Medical Research Council.

## Introduction

For many women with early-stage breast cancer, mastectomy can remove any detectable macroscopic disease, but some tumour foci might remain in locoregional tissue (ie, chest wall or regional lymph nodes) that could, if untreated, lead to recurrence of the disease and death from breast cancer. Radiotherapy has the potential to eliminate such tumour foci, and guidelines^1^, ^2^, ^3^, ^4^, ^5^, ^6^ now recommend that postmastectomy radiotherapy be given for women with four or more positive axillary lymph nodes, but not given for most women with node-negative disease. Most of these guidelines conclude, however, that there is insufficient evidence to make firm recommendations for women with one to three positive lymph nodes. A previous Early Breast Cancer Trialists' Collaborative Group (EBCTCG) analysis of individual patient data from randomised trials of postmastectomy radiotherapy^7^ did not give detailed results for women who had one to three positive lymph nodes after axillary dissection to at least level II, nor did it distinguish between trials in which radiotherapy included the chest wall and the regional lymph nodes and other trials in which radiotherapy was given only to the regional lymph nodes. For the present report, additional data regarding the extent of axillary dissection and regarding the number of positive lymph nodes have been obtained and reviewed for each woman, and we present detailed results according to these factors for trials that included radiotherapy to the chest wall, as is usual in current practice.

## Methods

### Study design

Trials beginning before 2000 of adjuvant radiotherapy versus no radiotherapy but the same surgery after mastectomy for invasive cancer were eligible for inclusion in our meta-analysis of individual patient data. Trial identification and data handling were as previously reported.^7^ For every woman, information was sought about initial patient and tumour characteristics, allocated treatment, time to first recurrence, whether the first recurrence was locoregional or distant, and date last known alive or date and underlying cause of death. When no recurrence was reported before breast cancer death, distant recurrence was assumed to have just preceded it. If contralateral breast cancer occurred before any other recurrence, follow-up was censored on that date in analyses of recurrence. Information about the extent of axillary surgery was reviewed. Women were classified as having axillary dissection if they were in a trial in which the protocol required removal of axillary lymph nodes in at least levels I and II. For the few trials in which the extent of axillary dissection was not described in terms of levels, women were classified as having axillary dissection if the trial protocol or publications indicated that the median number of resected nodes was at least ten or, if individual information was available,^8^, ^9^, ^10^, ^11^ at least ten nodes had been resected. Women with less extensive axillary surgery were classified as having axillary sampling.

### Statistical analysis

As in the recent overview of radiotherapy after breast-conserving surgery,^12^ the main emphasis in analyses of recurrence was on overall recurrence (ie, any first recurrence, irrespective of whether locoregional or distant), although observed risks of locoregional recurrence as a first event are also presented. All recurrence analyses present data only to year 10, because many trials did not follow women beyond this time for recurrence. Analyses of mortality present data to year 20. Deaths of unknown cause before recurrence were assumed to be from causes other than breast cancer, because most occurred many years after trial entry, by which time non-breast-cancer mortality predominated. Most other aspects of the statistical methods were as previously described,^7^ but further information is given in the appendix pp 7–9. Person-years up to Jan 1, 2009, were included. Analyses were stratified by trial, individual follow-up year, age at entry (<40, 40–49, 50–59, 60–69, or <70 years), and pathological nodal status (node-negative disease, one to three positive nodes, four or more positive nodes, node-positive disease with unknown number of nodes affected, or nodal status unknown). Analyses were programmed in the statistical package Stata (version 12.1) and the programming language R (version 2.13.2).

### Role of the funding sources

The Secretariat had full access to all data and analyses. The funding agencies had no role in data collection, analysis, interpretation, or reporting. A preliminary manuscript was presented to collaborators in October, 2013, and then revised on the basis of their comments. SD, CT, and PMcG had responsibility for submission of the report for publication.

## Results

**Figure 1:**
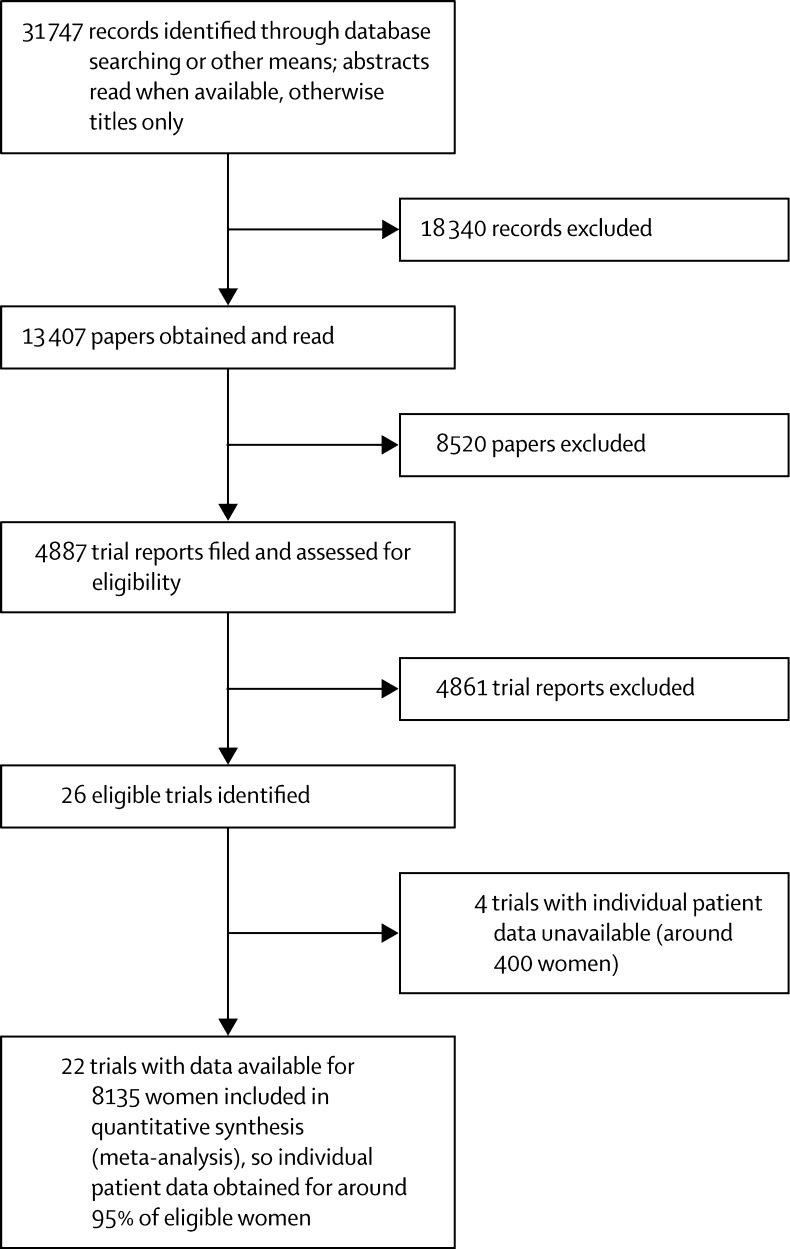
Trials included in analysis

Information was available for 8135 women in 22 trials in which radiotherapy included the chest wall and regional lymph nodes (table; figure 1). Median length of follow-up was 9·4 years per woman (IQR 3·7–17·3) and 5424 women (67%) were known to have died. The extent of axillary surgery was known for all but 183 (2%) women. 1594 (20%) women had pathologically node-negative disease, 5821 (72%) had pathologically node-positive disease, and for 720 pathological nodal status was unknown. There were 3831 women for whom pathological nodal status was known and who were classified as having axillary dissection (however, for 45 women with node-positive disease, the number of nodes affected was not known); all of these women were in trials in which radiotherapy included the chest wall, the supraclavicular or axillary fossa (or both), and the internal mammary chain (appendix pp 10–12).

**Table:** Availability of data from randomised trials beginning before the year 2000 and comparing radiotherapy to the chest wall and regional nodes after mastectomy and axillary surgery versus no radiotherapy but the same surgery

		**Women**	**Deaths**	Woman-years since diagnosis*					**Women given systemic therapy**†**(%)**		
				**Median per woman**	**Total (×10^3^)**	**Distribution by years (×10^3^)**			**Chemotherapy**	**Tamoxifen and ER+**‡	**Any**
						<10	10–19	≥20			
**(A) Axillary dissection**											
pN0		700	480	20·1	13·5	6·1	4·4	3·0	22%	27%	47%
pN+		3131	2074	7·2	30·1	20·3	7·9	1·9	75%	22%	91%
	pN1–3	1314	759	12·3	17·3	10·3	5·3	1·6	65%	24%	86%
	pN4+	1772	1286	4·8	12·4	9·7	2·5	0·3	81%	21%	95%
	pN?+	45	29	6·7	0·4	0·3	0·1	<0·1	100%	0%	100%
pN unknown		56	39	10·6	0·7	0·4	0·2	0·1	29%	71%	98%
Total		3887	2593	9·0	44·3	26·8	12·5	4·9	64%	24%	83%
**(B) Axillary sampling**											
pN0		870	595	17·6	15·4	7·5	5·1	2·8	10%	11%	21%
pN+		2541	1689	7·8	24·2	17·0	6·4	0·2	56%	28%	84%
pN unknown		654	460	9·3	7·1	4·6	2·1	0·4	44%	30%	74%
Total		4065	2744	9·8	46·8	29·1	13·7	4·0	44%	25%	69%
**(C) Axillary surgery, but extent unknown**											
pN0		24	12	8·5	0·2	0·2	<0·1	..	100%	0%	100%
pN+		149	69	11·5	1·3	1·1	0·2	..	100%	0%	100%
pN unknown		10	6	11·0	0·1	0·1	<0·1	..	100%	0%	100%
Total		183	87	10·1	1·6	1·4	0·2	..	100%	0%	100%
Total (A)+(B)+(C)		8135	5424	9·4	92·7	57·3	26·4	9·0	55%	24%	77%

Data were available for 22 trials, start dates 1964–86, and were unavailable for four trials including about 400 women. In all 22 trials for which data were available, radiotherapy was given to the chest wall and the supraclavicular or the axillary fossa (or both). In 20 of these 22 trials it was also given to the internal mammary chain. Details of the treatments given in these 22 trials are in appendix pp 10–12. Details of other trials of radiotherapy in combination with mastectomy are in appendix pp 52–53, 64–65, 70–71, 78–79. pN0=pathologically node-negative. pN+=pathologically node-positive. pN1–3=one to three pathologically positive nodes. pN4+=at least four pathologically positive nodes. pN?+=known to be pN+, but not whether pN1–3 or pN4+. pN unknown=pathological nodal status unknown.

* Numbers of woman-years of follow-up for mortality; many trials followed up women for only 10 years for recurrence.

† Chemotherapy was usually cyclophosphamide, methotrexate, and fluorouracil (CMF); only 3% of women were classified as oestrogen-receptor positive (ER+) and were in trials where both tamoxifen and chemotherapy were given.

‡ Oestrogen-receptor positive.

**Figure 2:**
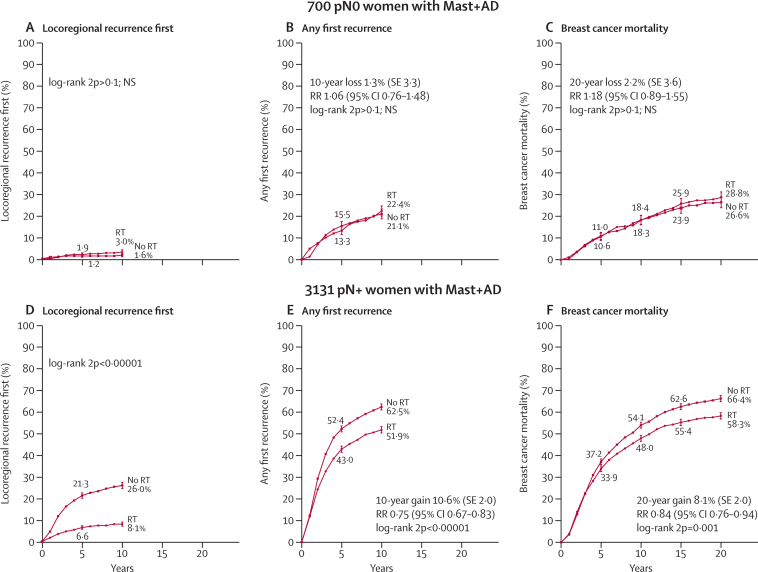
Effect of radiotherapy (RT) after mastectomy and axillary dissection (Mast+AD) on 10-year risks of locoregional and overall recurrence and on 20-year risk of breast cancer mortality in 700 women with pathologically node-negative (pN0) disease and in 3131 women with pathologically node-positive (pN+) disease Analyses of locoregional recurrence first ignore distant recurrences, see appendix pp 8–9 for details. See appendix pp 14, 16, for analyses of both locoregional and distant recurrences, and appendix pp 13, 15, for analyses of overall mortality. RR=rate ratio. NS=not significant. Vertical lines indicate 1 SE above or below the 5, 10, 15, and 20 year percentages.

Of the 1594 women with node-negative disease, 700 (44%) had axillary dissection, 870 (55%) had axillary sampling, and for 24 (1%) the extent of axillary surgery was unknown. For the 700 women who had axillary dissection, only 1·4% of unirradiated women had a locoregional recurrence before a distant recurrence (appendix p 14), and radiotherapy had no significant effect on locoregional recurrence (2p>0·1), overall recurrence (rate ratio [RR], irradiated *vs* not, 1·06, 95% CI 0·76–1·48, 2p>0·1), or breast cancer mortality (RR 1·18, 95% CI 0·89–1·55, 2p>0·1; figure 2A–C). Radiotherapy did, however, increase overall mortality (RR 1·23, 95% CI 1·02–1·49, 2p=0·03; appendix p 13). By contrast, for the 870 women with node-negative disease who had only axillary sampling, 16·3% of unirradiated women had a locoregional recurrence before any distant recurrence (appendix p 37), and radiotherapy reduced locoregional recurrence (2p<0·00001) and overall recurrence (RR 0·61, 95% CI 0·47–0·80, 2p=0·0003), but had no significant effect either on breast cancer mortality (RR 0·97, 95% CI 0·77–1·22, 2p>0·1) or on overall mortality (RR 1·00, 95% CI 0·84–1·18, 2p>0·1; appendix p 36).

Of the 5821 women with node-positive disease, 3131 (54%) had axillary dissection, 2541 (44%) had axillary sampling, and for 149 (2%) the extent of axillary surgery was unknown. Radiotherapy reduced locoregional recurrence and overall recurrence both for women who had axillary dissection and for women who had axillary sampling (2p<0·00001 for all four comparisons). However, 29·0% of the unirradiated women with axillary sampling had a locoregional recurrence before any distant recurrence (appendix p 39) compared with only 19·4% of the unirradiated women who had axillary dissection (appendix p 16) and the proportional reduction in the rate of overall recurrence was larger after axillary sampling (RR 0·59, 95% CI 0·53–0·66; appendix p 38) than after axillary dissection (RR 0·75, 95% CI 0·67–0·83, figure 2E; 2p for difference between RRs, 0·003).

For the 3131 women with node-positive disease who had axillary dissection, the proportional reductions in the overall recurrence rates did not differ significantly between years 0–4 and 5–9 (appendix p 17). Overall recurrence rates in the absence of radiotherapy were, however, higher during years 0–4 than during years 5–9 (figure 2E). Consequently, the absolute reduction with radiotherapy in the 10-year overall recurrence risk (10·6%—ie, 62·5% *vs* 51·9%), was only slightly greater than the 5-year absolute reduction (9·4%—ie, 52·4% *vs* 43·0%). The proportional reduction in the overall recurrence rate with radiotherapy did not differ according to whether or not there was a trial policy of giving systemic therapy (ie, either chemotherapy or, for oestrogen-receptor positive women, tamoxifen), or with any other known factor. For these 3131 women, radiotherapy reduced breast cancer mortality (RR 0·84, 95% CI 0·76–0·94, 2p=0·001). There was no significant heterogeneity in the proportional reduction in the breast cancer mortality rate according to any known tumour-related or treatment-related factor. There appeared to be little effect of radiotherapy on breast cancer mortality in the first few years, but after that the breast cancer mortality rate was lower in the radiotherapy group than in the controls until years 10–15 and possibly beyond (appendix p 17). Radiotherapy also reduced overall mortality in these 3131 women with node-positive disease who had axillary dissection (RR 0·89, 95% CI 0·81–0·97, 2p=0·01; appendix p 15).

**Figure 3:**
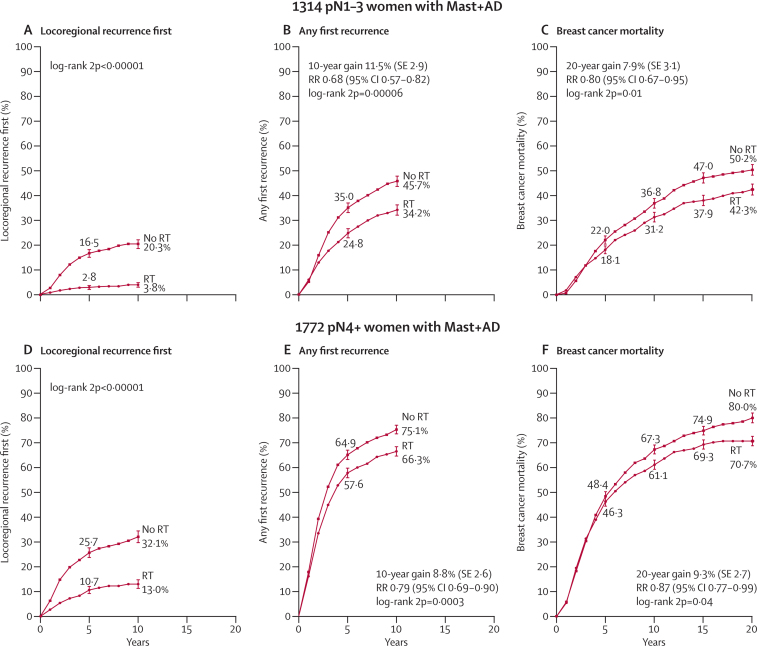
Effect of radiotherapy (RT) after mastectomy and axillary dissection (Mast+AD) on 10-year risks of locoregional and overall recurrence and on 20-year risk of breast cancer mortality in 1314 women with one to three pathologically positive nodes (pN1–3) and in 1772 women with four or more pathologically positive nodes (pN4+) Analyses of locoregional recurrence first ignore distant recurrences, see appendix pp 8–9 for details. See appendix pp 19, 28, for analyses of both locoregional and distant recurrences, and appendix pp 18, 27, for analyses of overall mortality. RR=rate ratio. NS=not significant. Vertical lines indicate 1 SE above or below the 5, 10, 15, and 20 year percentages.

**Figure 4:**
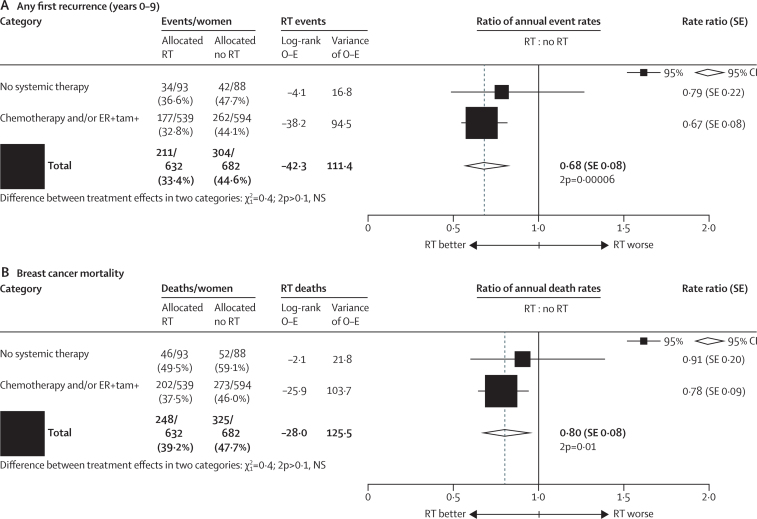
Effect of radiotherapy (RT) after mastectomy and axillary dissection on overall recurrence during years 0–9 and on breast cancer mortality for the entire follow-up in 1314 women with one to three pathologically positive nodes, according to whether or not they were in trials in which systemic therapy was given to both randomised treatment groups Chemotherapy was usually cyclophosphamide, methotrexate, and fluorouracil. ER-negative women in trials in which tamoxifen was given to both groups are included in the “no systemic” category. ER=oestrogen receptor. tam=tamoxifen. NS=not significant. SE=standard error.

**Figure 5:**
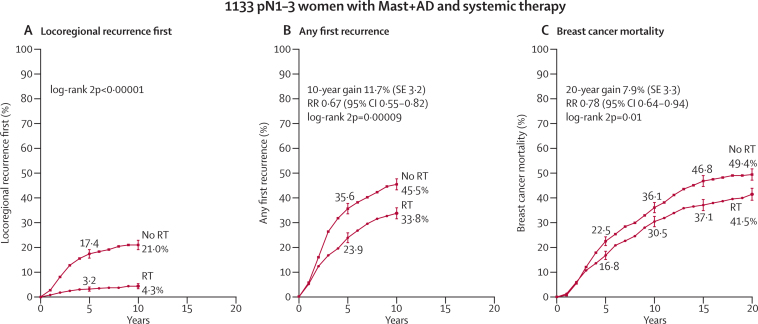
Effect of radiotherapy (RT) after mastectomy and axillary dissection (Mast+AD) on 10-year risks of locoregional and overall recurrence and on 20-year risk of breast cancer mortality in 1133 women with one to three pathologically positive nodes (pN1–3) in trials in which systemic therapy was given to both randomised treatment groups Analyses of locoregional recurrence first ignore distant recurrences, see appendix pp 8–9 for details. See appendix p 22 for analyses of both locoregional and distant recurrences, and appendix p 21 for analyses of overall mortality. RR=rate ratio. Vertical lines indicate 1 SE above or below the 5, 10, 15, and 20 year percentages.

Among the 1314 women who had axillary dissection and only one to three positive nodes, radiotherapy reduced locoregional recurrence (2p<0·00001), overall recurrence (RR 0·68, 95% CI 0·57–0·82, 2p=0·00006), and breast cancer mortality (RR 0·80, 95% CI 0·67–0·95, 2p=0·01; figure 3A–C). 813 women (62%) were in trials in which the policy regarding systemic therapy for both randomised groups was to give only chemotherapy, 274 (21%) had oestrogen-receptor positive disease and were in trials in which the policy was only to give tamoxifen, 46 (3%) had oestrogen-receptor positive disease and were in trials in which the policy was to give both tamoxifen and chemotherapy, and 181 (14%) were in trials in which the policy was not to give systemic therapy. The proportional reductions in the rates of overall recurrence and of breast cancer mortality did not differ significantly according to whether or not systemic therapy was given (figure 4) or according to any other known tumour-related or treatment-related factor (appendix p 20). When we considered separately the 1133 women with one to three positive nodes after axillary dissection who were in trials in which the policy was to give systemic therapy, radiotherapy reduced the rates of overall recurrence by a third (RR 0·67, 95% CI 0·55–0·82, 2p=0·00009) and breast cancer mortality by slightly more than a fifth (RR 0·78, 95% CI 0·64–0·94, 2p=0·01; figure 5).

**Figure 6:**
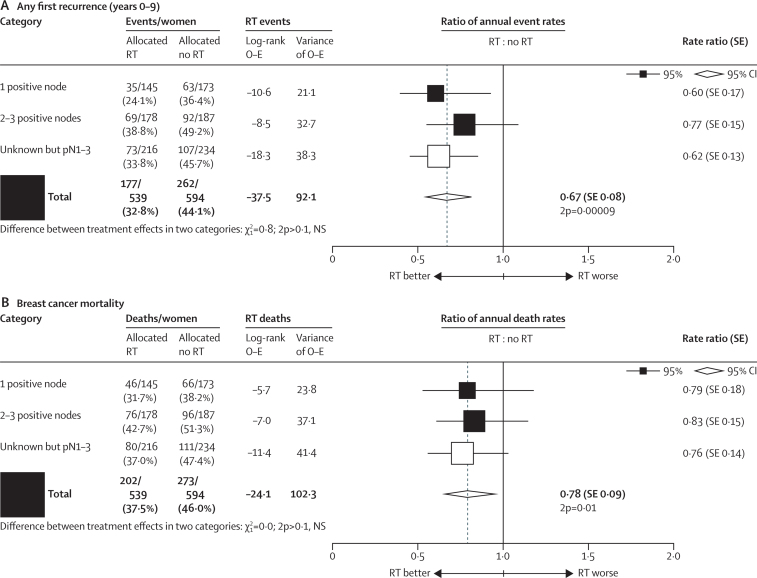
Effect of radiotherapy (RT) after mastectomy and axillary dissection on overall recurrence during years 0–9 and on breast cancer mortality for the entire follow-up in 1133 women with one to three pathologically positive nodes (pN1–3) in trials in which systemic therapy was given to both randomised treatment groups, by number of positive nodes See also appendix pp 23–26. NS=not significant. SE=standard error.

For 683 of these 1133 women, additional information about the number of positive nodes was available. 318 women had only one positive node, of whom 145 were randomly assigned to receive radiotherapy and 173 were randomly assigned not to receive it. 17·8% of the 173 unirradiated women with one positive node had a locoregional recurrence before any distant recurrence compared with only 2·3% of the irradiated women (2p<0·00001; appendix p 24), and radiotherapy reduced the rate of overall recurrence (0·60, 95% CI 0·39–0·92, 2p=0·02; figure 6A). This proportional reduction did not differ significantly from the corresponding reduction for the 365 women with two to three positive nodes after axillary dissection and who received systemic therapy (2p for difference >0·10). Likewise the proportional reduction in breast cancer mortality did not differ significantly according to the number of positive nodes (figure 6B).

For the 1772 women who had four or more positive nodes after axillary dissection, radiotherapy reduced locoregional recurrence (2p<0·00001) and overall recurrence (RR 0·79, 95% CI 0·69–0·90, 2p=0·0003) and breast cancer mortality (RR 0·87, 95% CI 0·77–0·99, 2p=0·04; figure 3D–F). 1677 (95%) of these women received systemic therapy and among them the proportional reductions in the overall recurrence and breast cancer mortality rates with radiotherapy did not differ significantly from those for the few who did not (2p for all differences >0·1; appendix p 29). Additional information about the number of positive nodes was available for 973 of the 1772 women with four or more positive nodes after axillary dissection. There were no significant differences in the proportional reductions with radiotherapy in the rates of overall recurrence or breast cancer mortality between the 542 women with four to nine positive nodes and the 431 women with ten or more positive nodes (2p>0·1 for all differences). Further information about women with four or more positive lymph nodes is given in appendix pp 27–35.

Information was also available from several other trials of postmastectomy radiotherapy. In eight trials (see appendix pp 52–53 for references) in which axillary surgery was performed and radiotherapy was given to the regional nodes but not to the chest wall, a total of 2304 women were randomised and the median follow-up was 7·2 years per woman (IQR 3·4–16·5; appendix pp 52–63). Pathological nodal status was available for 1494 women. Among the 1029 women with node-positive disease, 20·9% of unirradiated women had a locoregional recurrence before any distant recurrence compared with only 6·8% of irradiated women (2p<0·00001; appendix p 57) but radiotherapy had no significant effect on overall recurrence (RR 0·88, 95% CI 0·73–1·06, 2p>0·1), and no effect at all on the rate of breast cancer mortality (RR 1·00, 95% CI 0·82–1·20, 2p>0·1; appendix p 56). Information about further categories of trials of radiotherapy in combination with mastectomy is presented in appendix pp 64–85.

## Discussion

Previous meta-analyses have shown that, for women with node-positive disease, postmastectomy radiotherapy reduced the risks of both recurrence and breast cancer mortality.^7^ There has, however, been much debate as to whether this benefit was seen only because some women had limited axillary surgery. To investigate this issue, we reviewed all available evidence regarding the extent of axillary surgery for the women in these trials, including trial protocols, publications, and individual patient information if it was available. Additionally, we included the longer follow-up now available for many trials. The revised analyses of these updated data show that in these women who had mastectomy and axillary dissection of at least levels I and II, radiotherapy that included the chest wall, the supraclavicular or axillary fossa (or both), and the internal mammary chain reduced recurrence, breast cancer mortality, and overall mortality for all node-positive women considered together. When women with one to three and four or more involved axillary nodes were considered separately, the benefits of postmastectomy radiotherapy were clearly seen in each group. In these trials, 90% of women with one to three positive nodes and 95% of women with four or more positive nodes received some form of systemic treatment for their breast cancer. The most common chemotherapy was cyclophosphamide, methotrexate, and fluorouracil and the most common endocrine therapy was tamoxifen. The beneficial effects of radiotherapy on recurrence and breast cancer mortality remained apparent when women with one to three involved lymph nodes who were in trials in which the policy was to give systemic therapy were considered on their own. By contrast, in women with node-negative disease who received mastectomy and axillary dissection, among whom the proportion of women who had a locoregional recurrence before any distant recurrence was small, there was no evidence that radiotherapy provided any benefit. Non-breast-cancer mortality and the incidence of contralateral and other second cancers in these and other trials of radiotherapy in early breast cancer will be reported elsewhere.

There have been substantial changes in practice since these women were treated. For example, breast screening has improved and local therapies are more targeted. Also, the accuracy of lymph-node analysis has increased, with more frequent use of serial sectioning and more frequent recognition of micrometastases. Hence, some of the women who were classified as having node-negative disease in these trials might have been found to be node-positive if they had been assessed today.^13^, ^14^ Furthermore, many women now receive better systemic therapy that is more effective at treating both local and distant disease.^15^, ^16^ Therefore the absolute risk of a recurrence is likely to be lower for women being considered for postmastectomy radiotherapy today than for the women in these trials and the absolute risk reductions achieved with radiotherapy are also likely to be smaller.

The proportional risk reductions we observed could be applied to women today with one to three positive nodes if they have appreciable risks of recurrence in the absence of radiotherapy and they might be the best guide that is currently available to help to estimate the likely absolute benefits from radiotherapy for women today. For example, for cancer recurrence, the unirradiated women in these trials with one to three positive nodes had an absolute 10-year risk of overall recurrence of 45·7%, which was reduced to 34·2% by their radiotherapy, so their absolute gain was 11·5%. If the absolute 10-year risk of overall recurrence for women being considered for postmastectomy radiotherapy today were about half of this (ie, 23%), then the absolute gain might also be roughly halved (ie, to around 6%). Similarly, if the 20-year risk of breast cancer mortality in women with one to three positive nodes were roughly halved for women today, the absolute gain from radiotherapy would also be roughly halved from 7·9% to around 4%.

The proportional gains from radiotherapy might, however, be greater for women irradiated today than suggested by this example, because radiotherapy planning has changed substantially and women today receive better coverage of target areas. Furthermore, doses to normal tissues are lower today, so the risks of radiotherapy are also likely to be lower.

The most common site of locoregional cancer recurrence after mastectomy is the chest wall.^8^, ^17^ Eight trials have been carried out, including around 2000 women, in which the chest wall was not irradiated. In these eight trials radiotherapy reduced locoregional recurrence in women with pathologically node-positive disease but had no significant effect on overall recurrence and no effect at all on breast cancer mortality. This finding suggests that the chest wall is an important target in postmastectomy radiotherapy. Direct evidence regarding the effect of radiotherapy to the chest wall is being collected,^18^ but mature results from this current trial will not be available for some time. In the trials that are the main focus of the present report the chest wall, the supraclavicular or the axillary fossa (or both), and the internal mammary chain were all irradiated. Which regional lymph nodes should be irradiated after mastectomy is currently uncertain. Other randomised trials are investigating the risks and benefits of regional node irradiation in addition to chest wall or whole breast radiotherapy,^19^, ^20^, ^21^ but again long-term results of these trials are not yet available.

In circumstances in which, even without radiotherapy, only a small percentage of women have locoregional recurrence as a first event after mastectomy, radiotherapy is unlikely to reduce either overall recurrence or breast cancer mortality. This scenario is illustrated by the women in these 22 trials who had axillary dissection and node-negative disease. If, however, a substantial percentage of women have locoregional recurrence as a first event in the absence of radiotherapy, then radiotherapy is likely to provide benefit both by preventing locoregional recurrence and by reducing distant recurrence and breast cancer mortality. This situation is illustrated by the 3131 women in these 22 trials who had axillary dissection and node-positive disease, for whom radiotherapy reduced the 10-year risk of a recurrence of any type by 10·6% (62·5% *vs* 51·9%) and the 20-year risk of death from breast cancer by 8·1% (66·4% *vs* 58·3%). We previously reported that in the trials of radiotherapy after breast-conserving surgery, the findings for both node-negative and node-positive disease were consistent with about one breast cancer death being avoided in the first 15 years after radiotherapy for every four recurrences of any type (ie, either locoregional or distant) avoided in the 10 years after radiotherapy.^12^ The women with node-positive disease in the 22 trials reported here generally had more advanced cancers and more extensive radiotherapy than the women in the trials of radiotherapy after breast-conserving surgery and, for these women, about one breast cancer death was avoided in the 20 years after radiotherapy for every 1·5 recurrences of any type (ie, either locoregional or distant) avoided during the first 10 years after radiotherapy.

Breast cancer is a disease with a long natural history. Many of the women in these trials have now been followed up for 20 years and therefore they provide information about the long-term benefits of radiotherapy. Radiotherapy techniques have improved in the past few decades and so the proportional benefits of radiotherapy are likely to be larger than in these trials. However, the absolute risks of breast cancer recurrence and mortality have reduced in many countries because of advances in detection and treatment of breast cancer, so the absolute benefits from postmastectomy radiotherapy today are likely to be smaller than those reported here.
