# Supplementary material for: Effect of radiotherapy after mastectomy and axillary surgery on 10-year recurrence and 20-year breast cancer mortality: meta-analysis of individual patient data for 8135 women in 22 randomised trials
Source: Lancet. 2014 Jun 20;383(9935):2127–35. doi: 10.1016/S0140-6736(14)60488-8 (PMC5015598; doi:10.1016/S0140-6736(14)60488-8)
Supplement: Supplementary appendix [file mmc1.pdf]

THE LANCET

Supplementary appendix

This appendix formed part of the original submission and has been peer reviewed. We post it as supplied by the authors.

Supplement to: EBCTCG (Early Breast Cancer Trialists' Collaborative Group). Effect of radiotherapy after mastectomy and axillary surgery on 10-year recurrence and 20-year breast cancer mortality: meta-analysis of individual patient data for 8135 women in 22 randomised trials. *Lancet* 2014; published online March 19. http://dx.doi.org/10.1016/S0140-6736(14)60488-8.

This online publication has been corrected. The corrected version first appeared at thelancet.com on Nov 21, 2014

Effect of radiotherapy after mastectomy and axillary surgery on 10-year recurrence and 20-year breast cancer mortality: meta-analysis of individual patient data for 8135 women in 22 randomised trials

EBCTCG (Early Breast Cancer Trialists' Collaborative Group)

Webappendix

Webfigure 1 **Methodological note**

Trials of radiotherapy to the chest wall and regional lymph nodes versus not after mastectomy and axillary dissection (Mast+AD)

Webtable 1 Randomised trials beginning before the year 2000 and comparing radiotherapy to the chest wall and regional lymph nodes versus not after mastectomy and axillary dissection (Mast+AD) or axillary sampling (Mast+AS) – treatment details.

Node negative (pN0)

Webfigure 2 Effect of radiotherapy (RT) to the chest wall and regional lymph nodes versus not after mastectomy and axillary dissection (Mast+AD): 10-year risk of locoregional recurrence and recurrence of any type and 20-year risk of breast cancer and all-cause mortality in 700 women with pathologically node-negative (pN0) disease.

Webfigure 3 Effect of radiotherapy (RT) to the chest wall and regional lymph nodes versus not after mastectomy and axillary dissection (Mast+AD):10-year risk of recurrence and type of first recurrence, by allocated treatment, in 700 women with pathologically node-negative (pN0) disease.

Node positive (pN+)

Webfigure 4 Effect of radiotherapy (RT) to the chest wall and regional lymph nodes versus not after mastectomy and axillary dissection (Mast+AD): 10-year risk of locoregional recurrence and recurrence of any type and 20-year risk of breast cancer and all-cause mortality in 3131 women with pathologically node-positive (pN+) disease.

Webfigure 5 Effect of radiotherapy (RT) to the chest wall and regional lymph nodes versus not after mastectomy and axillary dissection (Mast+AD): 10-year risk of recurrence and type of first recurrence, by allocated treatment, in 3131 women with pathologically node-positive (pN+) disease.

Webfigure 6 Effect of radiotherapy (RT) to the chest wall and regional lymph nodes versus not after mastectomy and axillary dissection (Mast+AD): Event rate ratios and 95% confidence intervals for locoregional recurrence and recurrence of any type during years 0-9 and for breast cancer mortality in 3131 women with pathologically node-positive (pN+) disease by prognostic and other factors.

1-3 positive nodes (pN1-3)

Webfigure 7 Effect of radiotherapy (RT) to the chest wall and regional lymph nodes versus not after mastectomy and axillary dissection (Mast+AD): 10-year risk of locoregional recurrence and recurrence of any type and 20-year risk of breast cancer and all-cause mortality in 1314 women with 1-3 pathologically positive nodes (pN1-3).

Webfigure 8 Effect of radiotherapy (RT) to the chest wall and regional lymph nodes versus not after mastectomy and axillary dissection (Mast+AD): 10-year risk of recurrence and type of first recurrence, by allocated treatment, in 1314 women with 1-3 pathologically positive nodes (pN1-3).

Webfigure 9 Effect of radiotherapy (RT) to the chest wall and regional lymph nodes versus not after mastectomy and axillary dissection (Mast+AD): Event rate ratios and 95% confidence intervals for locoregional recurrence and recurrence of any type during years 0-9 and for breast cancer mortality in 1314 women with 1-3 pathologically positive nodes (pN1-3) by prognostic and other factors.

1-3 positive nodes (pN1-3) who received systemic therapy

Webfigure 10	Effect of radiotherapy (RT) to the chest wall and regional lymph nodes versus not after mastectomy and axillary dissection (Mast+AD): 10-year risk of locoregional recurrence and recurrence of any type and 20-year risk of breast cancer and all-cause mortality in 1133 women with 1-3 pathologically positive nodes (pN1-3) in trials where systemic therapy was given to both randomised treatment groups.
Webfigure 11	Effect of radiotherapy (RT) to the chest wall and regional lymph nodes versus not after mastectomy and axillary dissection (Mast+AD): 10-year risk of recurrence and type of first recurrence, by allocated treatment, in 1133 women with 1-3 pathologically positive nodes (pN1-3) in trials where systemic therapy was given to both randomised treatment groups.

1-3 positive nodes (pN1-3) who received systemic therapy subdivided according to number of positive nodes

Webfigure 12	Effect of radiotherapy (RT) to the chest wall and regional lymph nodes versus not after mastectomy and axillary dissection (Mast+AD): 10-year risk of locoregional recurrence and recurrence of any type and 15-year risk of breast cancer mortality in 1133 women with 1-3 pathologically positive nodes (pN1-3) in trials where systemic therapy was given to both randomised treatment groups subdivided according to number of positive nodes.
Webfigure 13	Effect of radiotherapy (RT) to the chest wall and regional lymph nodes versus not after mastectomy and axillary dissection (Mast+AD): 10-year risk of recurrence and type of first recurrence, by allocated treatment, in 318 women with 1 pathologically positive node (pN1) and where systemic therapy was given to both randomised treatment groups.
Webfigure 14	Effect of radiotherapy (RT) to the chest wall and regional lymph nodes versus not after mastectomy and axillary dissection (Mast+AD): 10-year risk of recurrence and type of first recurrence, by allocated treatment, in 365 women with 2-3 pathologically positive nodes (pN2-3) and where systemic therapy was given to both randomised treatment groups.
Webfigure 15	Effect of radiotherapy (RT) to the chest wall and regional lymph nodes versus not after mastectomy and axillary dissection (Mast+AD): 10-year risk of recurrence and type of first recurrence, by allocated treatment, in 450 women with 1-3 pathologically positive nodes (pN1-3) but the exact number of positive nodes unknown and where systemic therapy was given to both randomised treatment groups.

4+ positive nodes (pN4+)

Webfigure 16	Effect of radiotherapy (RT) to the chest wall and regional lymph nodes versus not after mastectomy and axillary dissection (Mast+AD): 10-year risk of locoregional recurrence and recurrence of any type and 20-year risk of breast cancer and all-cause mortality in 1772 women with 4+ pathologically positive nodes (pN4+).
Webfigure 17	Effect of radiotherapy (RT) to the chest wall and regional lymph nodes versus not after mastectomy and axillary dissection (Mast+AD): 10-year risk of recurrence and type of first recurrence, by allocated treatment, in 1772 women with 4+ pathologically positive nodes (pN4+).
Webfigure 18	Effect of radiotherapy (RT) to the chest wall and regional lymph nodes versus not after mastectomy and axillary dissection (Mast+AD): Event rate ratios and 95% confidence intervals for locoregional recurrence and recurrence of any type during years 0-9 and for breast cancer mortality in 1772 women with 4+ pathologically positive nodes (pN4+) by prognostic and other factors.

4+ positive nodes (pN4+) who received systemic therapy

Webfigure 19	Effect of radiotherapy (RT) to the chest wall and regional lymph nodes versus not after mastectomy and axillary dissection (Mast+AD): 10-year risk of locoregional recurrence and recurrence of any type and 20-year risk of breast cancer and all-cause mortality in 1677 women with 4+ pathologically positive nodes (pN4+) in trials where systemic therapy was given to both randomised treatment groups.
Webfigure 20	Effect of radiotherapy (RT) to the chest wall and regional lymph nodes versus not after mastectomy and axillary dissection (Mast+AD): 10-year risk of recurrence and type of first recurrence, by allocated treatment, in 1677 women with 4+ pathologically positive nodes (pN4+) in trials where systemic therapy was given to both randomised treatment groups.

4+ positive nodes (pN4+) who received systemic therapy subdivided according to number of positive nodes

Webfigure 21	Effect of radiotherapy (RT) to the chest wall and regional lymph nodes versus not after mastectomy and axillary dissection (Mast+AD): 10-year risk of locoregional recurrence and recurrence of any type and 15-year risk of breast cancer mortality in 1677 women with 4+ pathologically positive nodes (pN4+) in trials where systemic therapy was given to both randomised treatment groups subdivided according to number of positive nodes.

Webfigure 22 Effect of radiotherapy (RT) to the chest wall and regional lymph nodes versus not after mastectomy and axillary dissection (Mast+AD): 10-year risk of recurrence and type of first recurrence, by allocated treatment, in 479 women with 4-9 pathologically positive nodes (pN4-9) in trials where systemic therapy was given to both randomised treatment groups.

Webfigure 23 Effect of radiotherapy (RT) to the chest wall and regional lymph nodes versus not after mastectomy and axillary dissection (Mast+AD): 10-year risk of recurrence and type of first recurrence, by allocated treatment, in 403 women with 10+ pathologically positive nodes (pN10+) in trials where systemic therapy was given to both randomised treatment groups.

Webfigure 24 Effect of radiotherapy (RT) to the chest wall and regional lymph nodes versus not after mastectomy and axillary dissection (Mast+AD): 10-year risk of recurrence and type of first recurrence, by allocated treatment, in 795 women with 4+ pathologically positive nodes but the exact number of positive nodes unknown in trials where systemic therapy was given to both randomised treatment groups.

Trials of radiotherapy to the chest wall and regional lymph nodes versus not after mastectomy and axillary sampling (Mast+AS)

Node negative (pN0)

Webfigure 25 Effect of radiotherapy (RT) to the chest wall and regional lymph nodes versus not after mastectomy and axillary sampling (Mast+AS): 10-year risk of locoregional recurrence and recurrence of any type and 20-year risk of breast cancer and all-cause mortality in 870 women with pathologically node-negative (pN0) disease.

Webfigure 26 Effect of radiotherapy (RT) to the chest wall and regional lymph nodes versus not after mastectomy and axillary sampling (Mast+AS): 10-year risk of recurrence and type of first recurrence, by allocated treatment, in 870 women with pathologically node negative (pN0) disease.

Node positive (pN+)

Webfigure 27 Effect of radiotherapy (RT) to the chest wall and regional lymph nodes versus not after mastectomy and axillary sampling (Mast+AS): 10-year risk of locoregional recurrence and recurrence of any type and 20-year risk of breast cancer and all-cause mortality in 2541 women with pathologically node-positive (pN+) disease.

Webfigure 28 Effect of radiotherapy (RT) to the chest wall and regional lymph nodes versus not after mastectomy and axillary sampling (Mast+AS): 10-year risk of recurrence and type of first recurrence, by allocated treatment, in 2541 women with pathologically node-positive (pN+) disease.

Trials of radiotherapy to the chest wall and regional lymph nodes versus not after mastectomy and axillary dissection (Mast+AD) or axillary sampling (Mast+AS). Event rate ratios, one line per trial.

Webfigure 29 Effect of radiotherapy (RT) to the chest wall and regional lymph nodes versus not after mastectomy and axillary dissection (Mast+AD) or axillary sampling (Mast+AS): Event rate ratios, one line per trial, for locoregional recurrence and recurrence of any type during years 0-9 and for breast cancer and all-cause mortality in 1594 women with pathologically node-negative (pN0) disease.

Webfigure 30 Effect of radiotherapy (RT) to the chest wall and regional lymph nodes versus not after mastectomy and axillary dissection (Mast+AD) or axillary sampling (Mast+AS): Event rate ratios, one line per trial, for locoregional recurrence and recurrence of any type during years 0-9 and for breast cancer and all-cause mortality in 5821 women with pathologically node-positive (pN+) disease.

Webfigure 31 Effect of radiotherapy (RT) to the chest wall and regional lymph nodes versus not after mastectomy and axillary dissection (Mast+AD) or axillary sampling (Mast+AS): Event rate ratios, one line per trial, for locoregional recurrence and recurrence of any type during years 0-9 and for breast cancer and all-cause mortality in 2801 women with 1-3 pathologically positive nodes (pN1-3).

Webfigure 32 Effect of radiotherapy (RT) to the chest wall and regional lymph nodes versus not after mastectomy and axillary dissection (Mast+AD) or axillary sampling (Mast+AS): Event rate ratios, one line per trial, for locoregional recurrence and recurrence of any type during years 0-9 and for breast cancer and all-cause mortality in 2557 women with 4+ pathologically positive nodes (pN4+).

Webfigure 33 Effect of radiotherapy (RT) to the chest wall and regional lymph nodes versus not after mastectomy and axillary dissection (Mast+AD) or axillary sampling (Mast+AS): Event rate ratios, one line per trial, for locoregional recurrence and recurrence of any type during years 0-9 and for breast cancer and all-cause mortality in 463 women with pathologically positive nodes (pN?+) but unknown if they were 1-3 or 4+ positive.

Webfigure 34 Effect of radiotherapy (RT) to the chest wall and regional lymph nodes versus not after mastectomy and axillary dissection (Mast+AD) or axillary sampling (Mast+AS): Event rate ratios, one line per trial, for locoregional recurrence and recurrence of any type during years 0-9 and for breast cancer and all-cause mortality in 720 women with unknown pathological nodal status (pN?).

Trials of radiotherapy to the regional lymph nodes alone versus not after mastectomy and axillary dissection (Mast+AD)

Webtable 2 Availability of data from randomised trials beginning before the year 2000 and comparing radiotherapy to the regional lymph nodes alone versus not after mastectomy and axillary dissection (Mast+AD) or axillary sampling (Mast+AS).

Webtable 3 Randomised trials beginning before the year 2000 and comparing radiotherapy to the regional lymph nodes alone versus not after mastectomy and axillary dissection (Mast+AD) or axillary sampling (Mast+AS) – treatment details.

Node negative (pN0)

Webfigure 35 Effect of radiotherapy (RT) to the regional lymph nodes alone versus not after mastectomy and axillary dissection (Mast+AD): 10-year risk of locoregional recurrence and recurrence of any type and 20-year risk of breast cancer and all-cause mortality in 465 women with pathologically node-negative (pN0) disease.

Webfigure 36 Effect of radiotherapy (RT) to the regional lymph nodes alone versus not after mastectomy and axillary dissection (Mast+AD): 10-year risk of recurrence and type of first recurrence, by allocated treatment, in 465 women with pathologically node-negative (pN0) disease.

Node positive (pN+)

Webfigure 37 Effect of radiotherapy (RT) to the regional lymph nodes alone versus not after mastectomy and axillary dissection (Mast+AD): 10-year risk of locoregional recurrence and recurrence of any type and 20-year risk of breast cancer and all-cause mortality in 1029 women with pathologically node-positive (pN+) disease.

Webfigure 38 Effect of radiotherapy (RT) to the regional lymph nodes alone versus not after mastectomy and axillary dissection (Mast+AD): 10-year risk of recurrence and type of first recurrence, by allocated treatment, in 1029 women with pathologically node positive (pN+) disease.

Event rate ratios, one line per trial

Webfigure 39 Effect of radiotherapy (RT) to the regional lymph nodes alone versus not after mastectomy and axillary dissection (Mast+AD) or axillary sampling (Mast+AS): Event rate ratios, one line per trial, for locoregional recurrence and recurrence of any type during years 0-9 and for breast cancer and all-cause mortality in 465 women with pathologically node-negative (pN0) disease.

Webfigure 40 Effect of radiotherapy (RT) to the regional lymph nodes alone versus not after mastectomy and axillary dissection (Mast+AD) or axillary sampling (Mast+AS): Event rate ratios, one line per trial, for locoregional recurrence and recurrence of any type during years 0-9 and for breast cancer and all-cause mortality in 1029 women with pathologically node-positive (pN+) disease.

Webfigure 41 Effect of radiotherapy (RT) to the regional lymph nodes alone versus not after mastectomy and axillary dissection (Mast+AD) or axillary sampling (Mast+AS): Event rate ratios, one line per trial, for locoregional recurrence and recurrence of any type during years 0-9 and for breast cancer and all-cause mortality in 810 women unknown with pathological nodal status (pN?).

Trials of radiotherapy to the chest wall and regional lymph nodes versus not after mastectomy alone (Mast alone)

Webtable 4 Availability of data from randomised trials beginning before the year 2000 and comparing radiotherapy to the regional lymph nodes alone versus not after mastectomy but no axillary surgery (Mast).

Webtable 5 Randomised trials beginning before the year 2000 and comparing radiotherapy to the chest wall and regional lymph nodes versus not after mastectomy but no axillary surgery (Mast) – treatment details.

	Clinically node positive (cN+)
Webfigure 42	Effect of radiotherapy (RT) to the chest wall and regional lymph nodes versus not after mastectomy but no axillary surgery (Mast): 10-year risk of locoregional recurrence and recurrence of any type and 20-year risks of breast cancer and all-cause mortality in 2896 women with clinically node-negative (cN-) disease.
Webfigure 43	Effect of radiotherapy (RT) to the chest wall and regional lymph nodes versus not after mastectomy but no axillary surgery (Mast): 10-year risk of recurrence and type of first recurrence in 2896 women with clinically node-negative (cN-) disease.
	Clinically node negative (cN-)
Webfigure 44	Effect of radiotherapy (RT) to the chest wall and regional lymph nodes versus not after mastectomy but no axillary surgery (Mast): 10-year risk of locoregional recurrence and recurrence of any type and 20-year risks of breast cancer and all-cause mortality in 1481 women with clinically node-positive (cN+) disease.
Webfigure 45	Effect of radiotherapy (RT) to the chest wall and regional lymph nodes versus not after mastectomy but no axillary surgery (Mast): 10-year risk of recurrence and type of first recurrence in 1481 women with clinically node-positive (cN+) disease.

Trials of radiotherapy to the regional lymph nodes alone versus not after mastectomy alone (Mast alone)

Webtable 6	Availability of data from randomised trials beginning before the year 2000 and comparing radiotherapy to the regional lymph nodes alone versus not after mastectomy but no axillary surgery (Mast).
Webtable 7	Randomised trials beginning before the year 2000 and comparing radiotherapy to the regional lymph nodes alone versus not after mastectomy but no axillary surgery (Mast) – treatment details.
Webfigure 46	Effect of radiotherapy (RT) to the regional lymph nodes alone versus not after mastectomy but no axillary surgery (Mast): 10-year risks of recurrence, breast cancer and all-cause mortality in 192 clinically node-positive (cN+) women. Note, due to the very small number (8) of clinically node-negative women in this set of trials they are shown only in webfigure 34.
Webfigure 47	Effect of radiotherapy (RT) to the regional lymph nodes versus not after mastectomy but no axillary surgery (Mast): 10-year risk of recurrence and type of first recurrence in 192 women with clinically node-positive (cN+) disease.
Webfigure 48	Effect of radiotherapy (RT) versus not after mastectomy but no axillary surgery (Mast): 10 year risks of recurrence during years 0-9, breast cancer mortality, and all-cause mortality in 2904 women with clinically node-negative (cN-) disease. Event rate ratios, one line per trial, trial subdivided according to whether or not radiotherapy was given to the chest wall.
Webfigure 49	Effect of radiotherapy (RT) versus not after mastectomy but no axillary surgery (Mast): 10 year risks of recurrence during years 0-9, breast cancer mortality, and all-cause mortality in 1673 women with clinically node-positive (cN+) disease. Event rate ratios, one line per trial, trial subdivided according to whether or not radiotherapy was given to the chest wall.

Trials of radiotherapy to the chest wall and regional lymph nodes versus not BEFORE mastectomy and axillary dissection (Mast+AD) or axillary sampling (Mast+AS)

Webtable 8	Availability of data from randomised trials beginning before the year 2000 and comparing radiotherapy to the chest wall and regional lymph nodes versus not before mastectomy and axillary dissection (Mast+AD) or axillary sampling (Mast+AS).
Webtable 9	Randomised trials beginning before the year 2000 and comparing radiotherapy to the chest wall and regional lymph nodes versus not before mastectomy and axillary dissection (Mast+AD) or axillary sampling (Mast+AS) – treatment details.
Webfigure 50	Effect of radiotherapy (RT) to the chest wall and regional lymph nodes versus not before mastectomy and axillary dissection (Mast+AD): 10-year risk of locoregional recurrence and recurrence of any type and 15-year risk of breast cancer and all-cause mortality in 255 women with unknown pathological nodal status (pN?) disease.
Webfigure 51	Effect of radiotherapy (RT) to the chest wall and regional lymph nodes versus not before mastectomy and axillary dissection (Mast+AD): 10-year risk of

	recurrence and type of first recurrence, by allocated treatment, in 255 women with unknown pathological nodal status (pN?).
Webfigure 52	Effect of radiotherapy (RT) to the chest wall and regional lymph nodes versus not before mastectomy and axillary sampling (Mast+AS): 10-year risk of locoregional recurrence and recurrence of any type and 15-year risk of breast cancer and all-cause mortality in 637 women with unknown pathological nodal status (pN?) disease
Webfigure 53	Effect of radiotherapy (RT) to the chest wall and regional lymph nodes versus not before mastectomy and axillary sampling (Mast+AS): 10-year risk of recurrence and type of first recurrence, by allocated treatment, in 637 women with unknown pathological nodal status (pN?).
Webfigure 54	Effect of radiotherapy (RT) to the chest wall and regional lymph nodes versus not before mastectomy and axillary dissection (Mast+AD) or axillary sampling (Mast+AS): Event rate ratios, one line per trial, for locoregional recurrence and recurrence of any type during years 0-9 and for breast cancer and all-cause mortality in 892 women with unknown pathological nodal status (pN?).
Webfigure 55	**EBCTCG collaborators, listed alphabetically by institution and then alphabetically by name.**

Webfigure 1. Methodological Note

The analyses presented in the main body of the accompanying paper and also in many of the figures in this webappendix are based on the methodology that has been used throughout by the Early Breast Cancer Trialists' Collaborative Group (EBCTCG) and which is described elsewhere.[1] Some of the figures in this webappendix also include additional methodological features. The purpose of this note is to point out some of the features of both types of analysis.

Overall Mortality

In analyses of overall mortality (eg, the lower right-hand panels of webfigures 2, 4, etc), the number of women who are known to have died in each randomised group is related to the number of women at risk of dying and the length of time during which they are at risk of dying in each time-period during follow-up. Some women are, however, lost to follow-up and are withdrawn from the analysis. Thus, whilst it is reported in the lower right-hand panel of webfigure 4 that the cumulative risk of death from any cause among the 1550 women randomised to radiotherapy is 65.4% at 20 years after randomisation, this does not mean that 1014 (ie 0.654x1550) of the women are known to have died. Rather, as shown in webfigure 30, only 1001 (ie 64.6%) of the women are known to have died. The difference between these two percentages is due to the fact that for 390 of these 1550 women the most recent information held in the EBCTCG database indicates only that they were known to be alive at some period less than 20 years after randomisation. These women were withdrawn from the analysis or 'censored' on the date they were last known to be alive. Each censored woman is no longer considered to be at risk of dying after her date of censoring and she is excluded from all calculations relating to subsequent time-periods and, in particular, from contributing to the number of years at risk in calculations of the death rate. The technique of censoring has been used routinely by statisticians and actuaries for many decades and theoretical calculations have shown that it is valid, provided that the women who are censored are not different in any respect that affects their mortality rate from the women who remain in the study so that, from the mathematical point of view, the censoring can be considered to be 'at random'. This assumption is unlikely ever to be precisely true but many of the major factors affecting risk of overall mortality, such as trial, follow-up year, age at trial entry, and nodal status, can be taken into account through stratification, ie by subdividing the data into separate groups according to the stratifying factors, carrying out the analysis separately within each stratum and then combining the results from the separate strata in the form of a weighted average, calculated with weights proportional to the amount of information in each stratum.

Mortality from Causes other than Breast Cancer

Analyses of causes of death other than breast cancer (eg EBCTCG, Lancet 2000; 355:1757-70, and 2005; 366: 2087-2106) are carried out in a fashion similar to that for analyses of overall mortality. Here, however, it is not only women who are lost to follow-up who are censored but all women who have a recurrence of their breast cancer are also censored on the date of that recurrence. This approach enables comparison of mortality rates from non-breast-cancer causes in the two trial arms. However, the resulting estimates of the cumulative risk of death from all non-breast-cancer causes (eg figure 6 lower panel of EBCTCG, Lancet 2000; 355:1757-70) reflect the cumulative risks that would be seen under the hypothetical scenario that no women in the trial die from breast cancer. This scenario is, of course, highly artificial. It is, however, a useful one in that it permits comparison of non-breast-cancer mortality rates in the two trial arms unencumbered by any differences in the rates of breast cancer recurrence/mortality. It therefore enables identification and characterization of specific treatment hazards such as the increased mortality from heart disease or second cancers that has undoubtedly occurred following some of the radiotherapy regimens used in the past (EBCTCG, Lancet 2005; 366: 2087-2106).

Breast Cancer Mortality

The method used in the EBCTCG meta-analyses for studying mortality from breast cancer (eg right-hand panels of figures 1, 2, 4 and lower left-hand panels of webfigures 2, 4, etc) is indirect and makes use of analyses of the two endpoints described above. The data are first subdivided into separate strata (eg, according to trial, follow-up year, age at trial entry, and nodal status). Then, for each trial arm, the mortality rate from non-breast-cancer causes during the period prior to any recurrence of breast cancer is subtracted from the overall mortality rate in the relevant stratum. This method has the advantage that it avoids the difficulties which arise for women who die after a recurrence of their breast cancer and where it is not entirely clear whether their death was, in fact, due to the cancer or due to other causes. As in analyses of non-breast-cancer mortality, the resulting estimates of the cumulative risk of death from breast cancer reflect the cumulative risks that would be seen under the hypothetical scenario that no women in the trial die from causes other than breast cancer. Once again, this is useful in the identification and characterization of the benefits of a randomised treatment separately from its hazards. It also allows comparison of the benefits of the randomised treatment separately from the effects of other factors, such as the increasing overall mortality rate that occurs in all populations with increasing attained age.

continued overleaf

Separate calculation of the effect of a particular treatment on breast cancer mortality and on non-breast-cancer causes can also have substantial advantages even when the main question of interest is the effect of a treatment on overall mortality. For example, information from randomised trials on the effect of radiotherapy in reducing breast cancer mortality can be combined with epidemiological information from other sources on the likely risk of death from the long-term adverse effects of radiotherapy, such as second primary cancers or heart disease.

Analyses of Overall Recurrence

Analyses of overall recurrence are presented in both the main paper (eg middle panels of figures 1,2 and 4) and in the webappendix (upper right panel of webfigures 2, 4, etc). Rather than using the indirect approach that is taken for analyses of breast cancer mortality, these analyses are carried out in a fashion similar to the analyses of mortality from non-breast-cancer causes in that the first reported recurrence of any type is related to the number of women who have not yet had a recurrence but who, if they did have one, would contribute an event. Women are censored and cease to contribute either events or years at risk after they have had a recurrence, die from a cause other than breast cancer, or are lost to follow-up. Any women who are reported as dying from breast cancer and for whom no recurrence has previously been reported are assumed to have had a distant recurrence immediately preceding their death. As with analyses of mortality from breast cancer and from causes other than breast cancer, these analyses lead to estimates of the cumulative risk of recurrence that would occur under the hypothetical scenario in which no other events occur. For analyses of overall recurrence this involves the assumption that no women in the trial die from causes other than breast cancer. This is similar to the assumption that is made for analyses of breast cancer mortality and, once again, although this assumption is unrealistic it is useful in that it enables identification and characterization of the benefits of the randomised treatment separately from its hazards.

Analyses of Locoregional and Distant Recurrence

Analyses of locoregional recurrence are also presented both in the main paper (eg left panel of figures 1,2 and 4) and in the webappendix (upper left panel of webfigures 2, 4, etc). These analyses are carried out in similar fashion to the analyses of overall recurrence described above. Only locoregional recurrences that occur before any distant recurrence are counted as events, and women are censored and cease to contribute events or to the years at risk after they have had one recurrence (either a local or a distant one), or they die from a cause other than breast cancer or are lost to follow-up. The interpretation of analyses of locoregional recurrence is in some respects, similar to that for overall recurrence and breast cancer mortality. Two aspects do, however, differ and, in some contexts it is important to be aware of them. These two aspects are discussed in the following two paragraphs.

Firstly, because estimates of the cumulative risk of locoregional recurrence make the hypothetical assumption that no distant recurrences occur, they over-estimate the cumulative risk of locoregional recurrence. In many circumstances, including most of the analyses presented in this paper and in these webappendices, this is by no means realistic as the number of women whose first recurrence is a distant one is substantial. Insight into the extent of this effect can be gained by considering the distribution of the two different types of recurrence in analyses of overall recurrence, and such analyses have been carried out to accompany all the analyses of locoregional recurrence presented in this paper. For example, webfigure 5 accompanies the analysis of locoregional recurrence shown in the bottom left panel of figure 1 (and also in the top left panel of webfigure 4). The estimated 10-year risk of a recurrence of any type is 62.5% among the women randomised to no radiotherapy (webfigure 5, right-hand panel), of which distant recurrence accounts for 43.1% and locoregional recurrence accounts for the remaining 19.4%. If distant recurrences are censored, as in the analyses of locoregional recurrences, the estimated 10-year risk of locoregional recurrence in this particular example, is 26.0% (bottom left panel of figure 1 and top left panel of webfigure 4). This is 6.6% higher (ie, 26.0% in figure 1 minus 19.4% in webfigure 5) than the estimate derived from an analysis that takes distant recurrences into account.

Secondly, as can be seen in webfigure 5, the 10-year risk of distant recurrence differs between the two treatment groups and in this example, the 10-year risk of distant recurrence is 46.9% among the women allocated to receive radiotherapy and 43.1% among the women allocated not to receive it, ie, the 10-year risk of a distant recurrence is *higher* in the women randomised to receive radiotherapy than in the women randomised to no radiotherapy. This does not, however, mean that radiotherapy increases the risk of distant recurrence. Rather, it arises from the fact that a proportion of the women who would have had a locoregional recurrence if they had not had radiotherapy have their locoregional recurrence prevented by radiotherapy. These women remain at risk of a distant recurrence for longer and their additional time at risk is taken into account by the fact that, while they remain at risk of a distant recurrence, they continue to contribute to the years at risk and to the denominator in calculation of event rates. However, women who are at a higher risk of locoregional recurrence (eg, because they have more aggressive cancers) are also at a higher risk of distant recurrence. Therefore, the additional contribution to the years at risk from these women whose locoregional recurrence was prevented by the radiotherapy does not compensate fully for the additional risk of distant recurrence that is observed among the women allocated to radiotherapy. Hence the censoring that arises from the distant recurrences cannot be considered to be ‘at random’. The relationship between the risks of locoregional and distant recurrence is unknown, either in the presence of radiotherapy or in its absence – and indeed the relationship is likely to differ between the two. Furthermore, the data from the trial provide no information about this relationship. Therefore it is not possible to carry out analyses of locoregional recurrence that take appropriate account of the occurrence of distant recurrences as a first event

continued overleaf
event, or vice versa. One consequence of this is that, in analyses of locoregional recurrence as a first event (left-hand panels of figures 1, 2, & 5 and top left panels of webfigures 2, 4, 7, 10, 12, 16, 19, 21, 25, 27, 35, 37, 42, 44 and 46), the difference between the cumulative risks in the two treatment arms is a consequence not only of the causal effect of radiotherapy on the local recurrence rate in the two treatment arms, but also of the different extent to which distant recurrence as a first event occurs in each of the two treatment arms. This has consequences both for the interpretation of cumulative risks arising from the analysis of locoregional recurrence and for the interpretation of analyses presenting the ratio of the local recurrence rate in the irradiated group compared with the unirradiated group (figures 3, and 5 and webfigures 30, 6, 9, 18, 29, 30, 31, 32, 33, 34, 39, 40, 41, 48, 49). Analyses of recurrence presenting explicitly the percentages of women whose first recurrence was locoregional or distant respectively are therefore given in this webappendix (webfigures 3,5,8,11,13, 14, 15, 17, 20, 22, 23, 24, 26, 28, 36, 38, 43, 45, 47)

These ideas are not new, but they have not previously been considered in the context of the EBCTCG analyses. A selection of papers either discussing the methodological aspects involved or applying them to other data sets is given below.

- Fisher B, Anderson S, Redmond CK, Wolmark N, Wickerham DL, Cronin WM. Reanalysis and result after 12 years of follow-up in a randomized clinical trial comparing total mastectomy with lumpectomy with or without irradiation in the treatment of breast cancer. *N Engl J Med* 1995; **30**: 1456-1461.
- Gelman R, Gelber R, Henderson IC, Coleman CN, Harris JR. Improved methodology for analyzing local and distant recurrence. *J Clin Oncol* 1990; **8**: 548-555.
- Moeschberger ML, Klein JP. Statistical methods for dependent competing risks. *Lifetime Data Anal* 1995; **1**: 195-204.
- Panzarella T, Meakin JW. Analysis of cause-specific failure endpoints using simple proportions: an example from a randomized controlled clinical trial in early breast cancer. *Int J Radiat Oncol Biol Phys* 1998; **41**: 1093-97.
- Peterson AV. Bounds for a joint distribution function with fixed sub-distribution functions: Application to competing risks. *Proc Natl Acad Sci U S A* 1976; **73**: 11-13.
- Prentice RL, Kalbfleisch JD, Peterson AV, Flournoy N, Farewell VT, Breslow NE. The analysis of failure times in the presence of competing risks. *Biometrics* 1978; **34**: 541-554.
- Schulgen G, Schomoor C, Sauerbrei W, Schumacher M. A note on estimating local recurrence rates in clinical trials on the treatment of breast cancer. *Breast Cancer Res Treat* 1998; **49**: 87-91.
- Tsiatis A. A nonidentifiability aspect of the problem of competing risks. *Proc Natl Acad Sci U S A* 1975; **72**: 20-22.
- Dignam JJ, Kocherginsky MN. Choice and interpretation of statistical tests used when competing risks are present. *J Clin Oncol* 2008; **26**: 4027-34.
- Putter H, Fiocco M, Geskus RB. Tutorial in biostatistics. *Stat Med* 2007; **26**: 2389-430.

Reference

1. http://www.ctsu.ox.ac.uk/research/meta-trials/ebctcg/original-methods-for-ebctcg-meta-analyses

Webtable 1: Randomised trials beginning before the year 2000 and comparing radiotherapy to the chest wall and regional lymph nodes versus not after mastectomy and axillary dissection (Mast+AD) or axillary sampling (Mast+AS) – treatment details.

Year code and study name	Breast surgery	Axillary Surgery* (number of patients)	Chest wall RT	Supraclavicular (SC) and axillary fossa (AF) RT	Internal mammary chain RT	Boost RT to scar	Common systemic chemoendocrine therapy
64B Oslo X-ray	RM	Axillary dissection (552)	25-41 Gy (1.3-2.1 Gy/f) o	36 Gy (1.8 Gy/f) o, SC; 18 Gy (u Gy/f) o, AF	25-41 Gy (1.3-2.1 Gy/f) o	None	Ovarian RT
71B Stockholm A	MRM	Axillary sampling (644)	45 Gy (1.8 Gy/f) e	45 Gy de (1.8 Gy/f) c	45 Gy (1.8 Gy/f) e	None	None
73A Southampton UK	SM	Axillary sampling (151)	46 Gy (2.3 Gy/f) c	55 Gy (2.5 Gy/f) c & b	46 Gy (2.3 Gy/f) c	None	None
74B Edinburgh I	SM	Axillary sampling (348)	42.5-45.0 Gy (4.25-4.5 Gy/f) m	42.5-45.0 Gy (4.25-4.5 Gy/f) m	None	None	F
74D DFCI Boston	MRM or RM	Axillary dissection (218)	45 Gy (2.3 Gy/f) c or m	45 Gy (2.3 Gy/f) c or m	0-45 Gy (0-2.3 Gy/f) c or m	None	Either (AC) 5 cycles or (AC) 10 cycles; or CMF or MF
74Q Piedmont OA (pN4+)	MRM or RM	Axillary dissection (120)	50 Gy (1.5-1.8 Gy/f) c or m	45-50 Gy (1.5-2.8 Gy/f) c or m	45-50 Gy (1.8-2.8 Gy/f) c or m	None	Mel or CMF
76A SECSG 1	MRM or RM	Axillary dissection (257)	50 Gy (2 Gy/f) u	50 Gy (2 Gy/f) u	50 Gy (2 Gy/f) u	None	CMF
76C Glasgow	SM	Axillary dissection (219)	37.8 Gy (2.5 Gy/f) o	37.8 Gy (2.5 Gy/f) o	37.8 Gy (2.5 Gy/f) o	None	CMF
77J MD Ander. 7730B	MRM or SM	Axillary dissection (80)<br>Axillary sampling (17)	45-50 Gy (1.8-2.0 Gy/f) c	45-50 Gy (1.8-2 Gy/f) c	45-50 Gy (1.8-2 Gy/f) c or e	12 Gy (uGy/f) u	bCG+FAC or FAC
78A S Swedish BCG	MRM	Axillary dissection (771)	38 Gy (1.9 Gy/f) e,o,m or c	48-60 Gy (2.4 Gy/f) c or m	48 Gy (2.4 Gy/f) e, c or m	None	Premen: C;<br>Postmen: tam
78G BCCA Vancouver	MRM	Axillary dissection (318)	37.5-40 Gy (2.3 Gy/f) c or m	37.5 Gy de (2.2 Gy/f) c or m	37.5 Gy de (2.3 Gy/f) c or m	None	CMFP+ovarian RT or CMF
78Q Düsseldorf U	Patey	Axillary dissection (88)	40 Gy (2 Gy/f) c	40 Gy (2 Gy/f) c	40 Gy (2 Gy/f) c	None	LMF
79F Coimbra	NS	Axillary sampling (124)	36 Gy (3 Gy/f) o or m	39-45 Gy (3.3-3.8 Gy/f) m	39 Gy (3.3 Gy/f) m	None	AC
79G Metaxas Athens	MRM, Patey MRM, or RM	Axillary dissection (71)	45-60 Gy (2 Gy/f) m	45-60 Gy (2 Gy/f) m	45-60 Gy (2 Gy/f) m	None	CAMF & tam<br>Premen: ovarian RT
80S Helsinki	RM	Axillary dissection (99)	45 Gy (3 Gy/f) c	45 Gy (3 Gy/f) c, SC;<br>45 Gy (3 Gy/f) c, AF	45 Gy (3 Gy/f) c	None	CAFt
80W NSABC Israel	NS	Unknown (112)	46-50 Gy (2 Gy/f) c or m	46-50 Gy (2 Gy/f) c or m	40 Gy (2 Gy/f) c or m	None	CMF
82B Danish BCG 82b pre	SM	Axillary dissection (418)<br>Axillary sampling (1,386)	36-50 Gy (1.8-2.2 Gy/f) o or e	36-50 Gy (1.8-2.2 Gy/f) o or m	36-50 Gy (1.8-2.2 Gy/f) o or e	None	CMF
82C Danish BCG 82c post	SM	Axillary dissection (344)<br>Axillary sampling (1,119)	36-50 Gy (1.8-2.2 Gy/f) o or e	36-50 Gy (1.8-2.2 Gy/f) o or m	36-50 Gy (1.8-2.2 Gy/f) o or e	None	tam
82Q ECOG EST3181	MRM or RM	Axillary dissection (332)	46 Gy (2 Gy/f) c or m	46-50 Gy (2 Gy/f) c or m	46 Gy (2 Gy/f) c, m or e	None	CAF&H&tam
84A GBSG 03 Germany	Patey	Axillary sampling (199)	50 Gy (2 Gy/f) c or m	50 Gy (2 Gy/f) c or m	44 Gy(1.8 Gy/f) c or m	None	CMF
85F Nottingham	SM	Axillary sampling (77)	45 Gy (3 Gy/f) m	45 Gy (3 Gy/f) m	None	None	Premen; CMF<br>Postmen;tam
86C CRC, UK	NS	Unknown (71)	Various	Various	Various	Various	None

* Based on the description of axillary surgery in the trial protocol or publications or on information on individual women. Women were classified as having axillary dissection if they were in a trial where the protocol required removal of axillary lymph nodes in at least levels I & II or, if individual information was available (MD Ander. 7730B, Danish BCG 82b pre, Danish BCG 82c post), resection of ≥10 nodes. In other trials, women were classified as having axillary dissection if the trial publication indicated that the median number of nodes removed was ≥ 10. Women with less extensive axillary surgery were classified as having axillary sampling. A=doxorubicin (adriamycin), AC=doxorubicin and cyclophosphamide, AF=axillary fossa, b= additional posterior boost to axilla, bCG=bacillus Calmette-Guérin, C=cyclophosphamide, c=cobalt-60, de=dose at depth (of nodes), F=fluorouracil, Ft=Ftorafur, f=fraction, Gy=Gray (intended dose), H=halotestin, L=chlorambucil, m=megavoltage, M=methotrexate, Mel=melphalan, MRM=modified radical mastectomy, NS=surgery not specified in detail (Patey mastectomy, or modified radical mastectomy), o=orthovoltage, P=prednisone, Patey= Patey mastectomy, RM=radical mastectomy (Halsted), RT=radiotherapy, SC=supraclavicular, SM=simple (total) mastectomy; tam=tamoxifen, u=unknown.

References for Webtable 1

Year code and study name	Reference
64B Oslo X-ray	Host H, Brennhovd IO, Loeb M. Postoperative radiotherapy in breast cancer-long-term results from the Oslo study. *Int J Radiat Oncol Biol Phys* 1986; **12**: 727–32.
71B Stockholm A	Gyenes G, Rutqvist LE, Liedberg A, Fornander T. Long-term cardiac morbidity and mortality in a randomized trial of pre- and postoperative radiation therapy versus surgery alone in primary breast cancer. *Radiother Oncol* 1998; **48**: 185–90.
73A Southampton UK	Turnbull AR, Turner DT, Chant AD, Shepherd JM, Buchanan RB, Fraser JD. Treatment of early breast cancer. *Lancet* 1978; **2**: 7–9.
74B Edinburgh I	Stewart HJ, Jack WJL, Everington D, Forrest APM, Rodger A, McDonald CC, et al. South-east Scottish trial of local therapy in node negative breast cancer. *The Breast* 1994; **3**: 31–9.
74D DFCI Boston	Shapiro CL, Hardenbergh PH, Gelman R, Blanks D, Hauptman P, Recht A, et al. Cardiac effects of adjuvant doxorubicin and radiation therapy in breast cancer patients. *J Clin Oncol* 1998; **16**: 3493–501.
74Q Piedmont OA	Muss HB, Cooper MR, Brockschmidt JK, Ferree C, Richards F, 2nd, White DR, et al. A randomized trial of chemotherapy (L-PAM vs CMF) and irradiation for node positive breast cancer. Eleven year follow-up of a Piedmont Oncology Association trial. *Breast Cancer Res Treat* 1991; **19**: 77–84.
76A SECSG 1	Velez-Garcia E, Carpenter JT, Jr., Moore M, Vogel CL, Marcial V, Ketcham A, et al. Postsurgical adjuvant chemotherapy with or without radiotherapy in women with breast cancer and positive axillary nodes: a South-Eastern Cancer Study Group (SEG) Trial. *Eur J Cancer* 1992; **28A**: 1833–7.
76C Glasgow	McArdle CS, McMillan DC, Greenlaw N, Morrison DS. Adjuvant radiotherapy and chemotherapy in breast cancer: 30 year follow-up of survival. *BMC Cancer* 2010; **10**: 398.
77J MD Ander. 7730B	Katz A, Strom EA, Buchholz TA, Thames HD, Smith CD, Jhingran A, et al. Locoregional recurrence patterns after mastectomy and doxorubicin-based chemotherapy: implications for postoperative irradiation. *J Clin Oncol* 2000; **18**: 2817–27.
78A S Swedish BCG	Killander F, Anderson H, Ryden S, Moller T, Aspegren K, Ceberg J, et al. Radiotherapy and tamoxifen after mastectomy in postmenopausal women - 20 year follow-up of the South Sweden Breast Cancer Group randomised trial SSBCG II:I. *Eur J Cancer* 2007; **43**: 2100–8.
78G BCCA Vancouver	Ragaz J, Jackson SM, Le N, Plenderleith IH, Spinelli JJ, Basco VE, et al. Adjuvant Radiotherapy and Chemotherapy in Node-Positive Premenopausal Women with Breast Cancer *N Engl J Med* 1997; **337**:956-962
78Q Düsseldorf U	Faber P, Jesdinsky H. Adjuvant chemotherapy in breast cancer-a multicenter trial. *Cancer Treat Rev* 1979; **6** Suppl: 75–8.
79F Coimbra	De Oliveira CF R, F, Gervasio H, Alves, R, Silva A, Pedro L. Adjuvant chemotherapy versus radiotherapy and chemotherapy in operable breast cancer. A randomized trial. Preliminary results. Instituto Portugues De Oncologia Coimbra, *Portugal* 1984.
79G Metaxas Athens	Papaioannou AN. Preoperative chemotherapy: advantages and clinical application in stage III breast cancer. Recent Results Cancer Res 1985; **98**: 65–90.
80S Helsinki	Saarto T, Blomqvist C, Rissanen P, Auvinen A, Elomaa I. Haematological toxicity: a marker of adjuvant chemotherapy efficacy in stage II and III breast cancer. *Br J Cancer* 1997; **75**: 301–5.

80W NSABC Israel	H Hayat GB, R Borovik, S Chaichick, P Rathm E Robinson, S Biran, HJ Brenner. Adjuvant chemotherapy and radiation therapy vs. chemotherapy alone for stage II breast cancer patients. *Ann Oncol* 1990; **21** (suppl, abstr).
82B Danish BCG 82b pre	Andersson M, Kamby C, Jensen MB, Mouridsen H, Ejlertsen B, Dombernowsky P, et al. Tamoxifen in high-risk premenopausal women with primary breast cancer receiving adjuvant chemotherapy. Report from the Danish Breast Cancer co-operative Group DBCG 82B Trial. *Eur J Cancer* 1999; **35**: 1659–66.
	Kyndi M, Overgaard M, Nielsen HM, Sorensen FB, Knudsen H, Overgaard J. High local recurrence risk is not associated with large survival reduction after postmastectomy radiotherapy in high-risk breast cancer: a subgroup analysis of DBCG 82 b&c. *Radiother Oncol* 2009; **90**: 74–9.
82C Danish BCG 82c post	Overgaard M, Jensen MB, Overgaard J, Hansen PS, Rose C, Andersson M, et al. Postoperative radiotherapy in high-risk postmenopausal breast-cancer patients given adjuvant tamoxifen: Danish Breast Cancer Cooperative Group DBCG 82c randomised trial. *Lancet* 1999; **353**: 1641–8.
	Kyndi M, Overgaard M, Nielsen HM, Sorensen FB, Knudsen H, Overgaard J. High local recurrence risk is not associated with large survival reduction after postmastectomy radiotherapy in high-risk breast cancer: a subgroup analysis of DBCG 82 b&c. *Radiother Oncol* 2009; **90**: 74–9.
82Q ECOG EST3181	Olson JE, Neuberg D, Pandya KJ, Richter MP, Solin LJ, Gilchrist KW, et al. The role of radiotherapy in the management of operable locally advanced breast carcinoma: results of a randomized trial by the Eastern Cooperative Oncology Group. *Cancer* 1997; **79**: 1138–49.
84A GBSG 03 Germany	Schmoor C, Olschewski M, Sauerbrei W, Schumacher M. Long-term follow-up of patients in four prospective studies of the German Breast Cancer Study Group (GBSG): A summary of key results. *Onkologie* 2002; **25**: 143–50.
85F Nottingham	Morgan DA, Berridge J, Blamey RW. Postoperative radiotherapy following mastectomy for high-risk breast cancer. A randomised trial. *Eur J Cancer* 2002; **38**: 1107–10.
86C CRC, UK	Houghton J PI, Tobias J, Baum M, Odling-Smee W. Prophylactic radiotherapy following surgery for early breast cancer: is the benefit mainly in patients with involved margins? Results from a Cancer Research Campaign trial. *Proc Am Soc Clin Oncol* 2001; **20**: 31a.

Webfigure 2. Effect of radiotherapy (RT) to the chest wall and regional lymph nodes versus not after mastectomy and axillary dissection (Mast+AD): 10-year risk of locoregional recurrence and recurrence of any type and 20-year risk of breast cancer and all-cause mortality in 700 women with pathologically node-negative (pN0) disease. See webfigure 1 for methodological note and also webfigure 3.
Note: 1 locoregional recurrence, 5 recurrences of any type and 5 breast cancer deaths were reported among the 9 pN0 women with tumours ⩾ 5 cm who were allocated to receive radiotherapy. 0 locoregional recurrences, 3 recurrences of any type and 4 breast cancer deaths were reported among the 11 pN0 women with tumours ⩾ 5 cm who were allocated to not to receive radiotherapy.

700 pN0 women with Mast+AD

Locoregional recurrence first

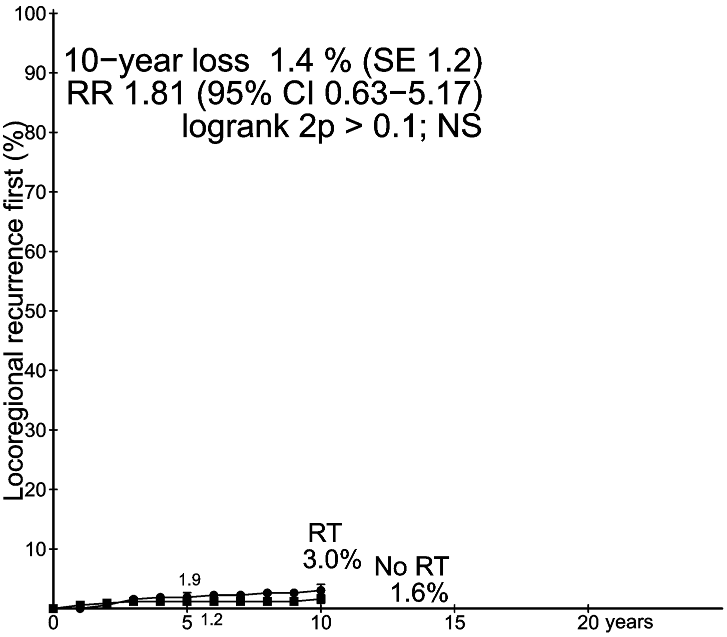

Locoregional recurrence first rates (%/year) and logrank analyses

	Years 0−4	Years 5−9
RT	0.39 (6/1554)	0.23 (3/1287)
No RT	0.25 (4/1580)	0.08 (1/1317)
Rate ratio, from	1.50 SE 0.78	2.89 SE 1.78
(O−E)/V	1.0/2.5	1.1/1.0

Any first recurrence

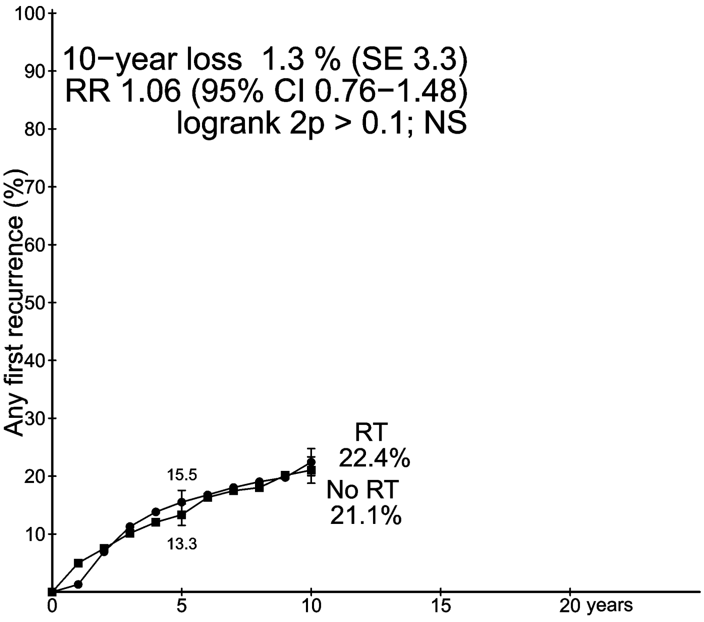

Any first recurrence rates (%/year) and logrank analyses

	Years 0−4	Years 5−9
RT	3.46 (54/1559)	1.62 (21/1296)
No RT	2.90 (46/1585)	2.02 (27/1339)
Rate ratio, from	1.16 SE 0.22	0.89 SE 0.28
(O−E)/V	3.3/23.0	−1.3/11.5

Breast cancer mortality

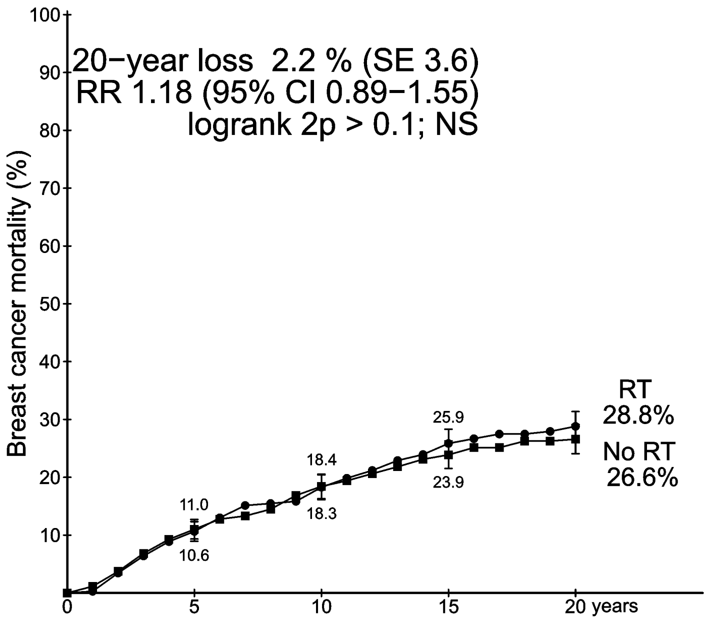

Breast cancer mortality rates (%/year) and logrank analyses

	Years 0−4	Years 5−9	Years 10−14	Years 15−19	Years 20+
RT	2.26 (37/1638)	1.77 (25/1411)	1.88 (22/1169)	0.73 (7/952)	1.45 (20/1375)
No RT	2.30 (38/1653)	1.75 (25/1430)	1.46 (18/1237)	0.87 (9/1034)	1.01 (16/1583)
Rate ratio, from	0.96 SE 0.24	1.06 SE 0.31	1.39 SE 0.39	1.16 SE 0.55	1.76 SE 0.49
(O−E)/V	−0.7/17.1	0.7/11.4	3.1/9.4	0.6/3.8	4.4/7.7

Any death

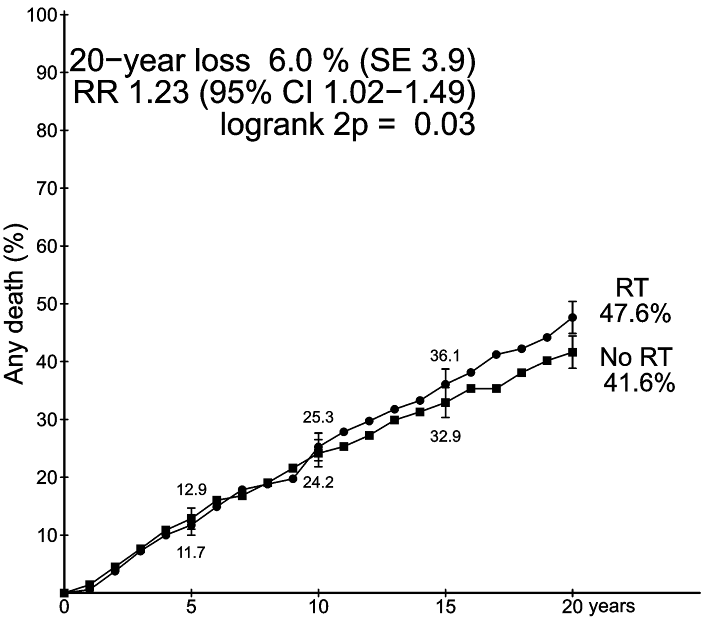

Any death rates (%/year) and logrank analyses

	Years 0−4	Years 5−9	Years 10−14	Years 15−19	Years 20+
RT	2.50 (41/1638)	3.26 (46/1411)	3.08 (36/1169)	3.88 (37/952)	5.96 (82/1375)
No RT	2.78 (46/1653)	2.87 (41/1430)	2.59 (32/1237)	3.09 (32/1034)	5.50 (87/1583)
Rate ratio, from	0.90 SE 0.21	1.20 SE 0.24	1.28 SE 0.28	1.45 SE 0.31	1.38 SE 0.20
(O−E)/V	−2.1/20.0	3.6/20.3	3.9/15.9	5.7/15.4	10.8/33.5

Webfigure 3. Effect of radiotherapy (RT) to the chest wall and regional lymph nodes versus not after mastectomy and axillary dissection (Mast+AD):
10-year risk of recurrence and type of first recurrence, by allocated treatment, in 700 women with pathologically node-negative (pN0) disease.
(r_L = number of women for whom first recurrence was locoregional, r_D = number women for whom distant recurrence was first.)

700 pN0 women with Mast+AD

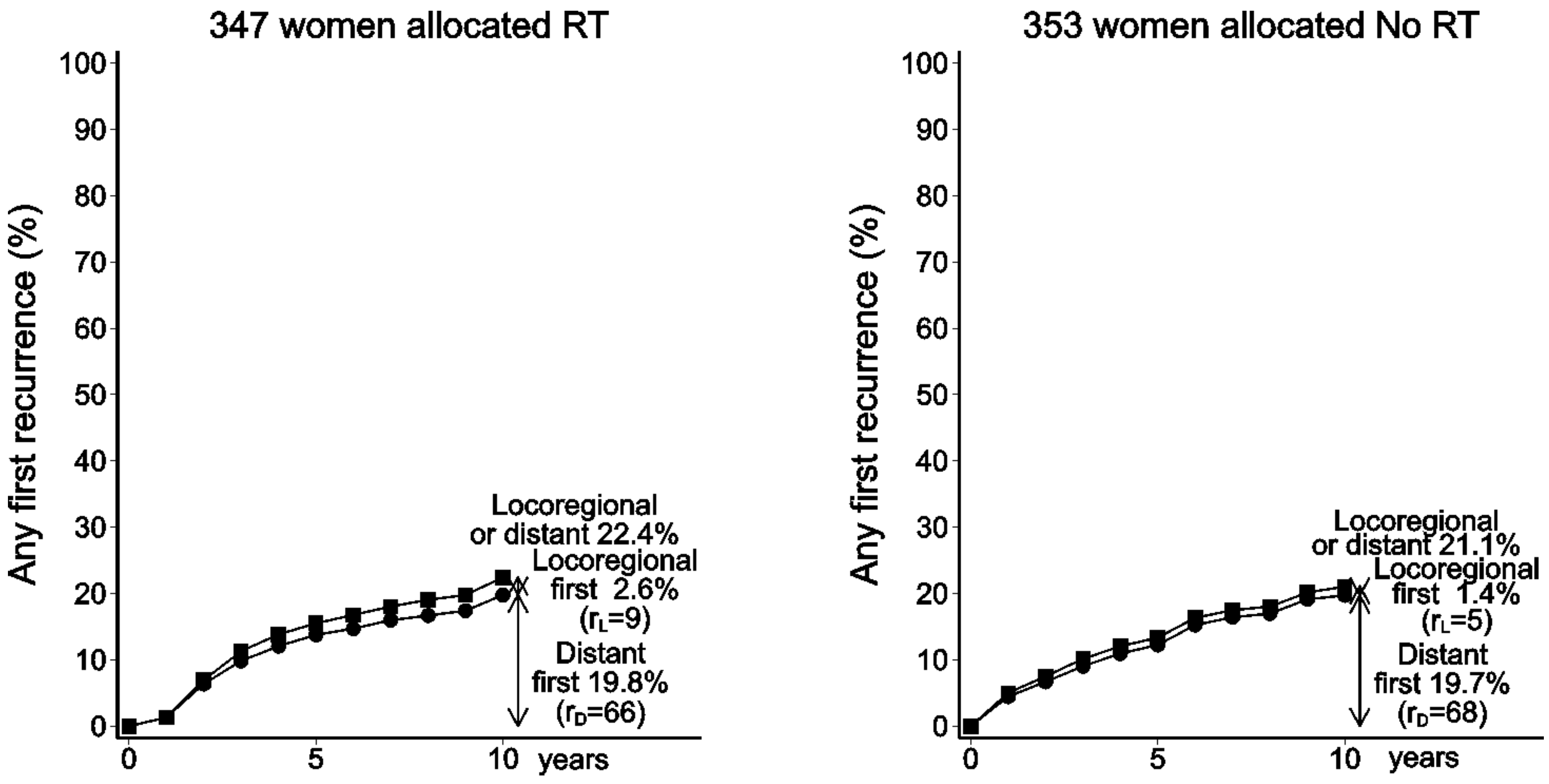

2p for difference between treatment arms in the proportion of all first recurrences that were locoregional: > 0.1; NS

Webfigure 4. Effect of radiotherapy (RT) to the chest wall and regional lymph nodes versus not after mastectomy and axillary dissection (Mast+AD): 10-year risk of locoregional recurrence and recurrence of any type and 20-year risk of breast cancer and all-cause mortality in 3131 women with pathologically node-positive (pN+) disease. See webfigure 1 for methodological note and also webfigure 5.

3131 pN+ women with Mast+AD

Locoregional recurrence first

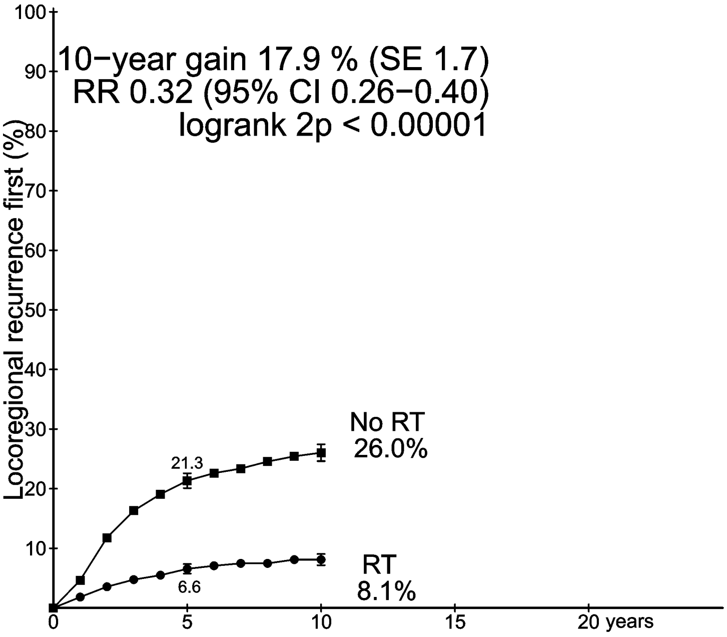

Locoregional recurrence first rates (%/year) and logrank analyses

	Years 0−4	Years 5−9
RT	1.61 (86/5335)	0.41 (13/3154)
No RT	5.04 (256/5075)	1.25 (34/2714)
Rate ratio, from	0.32 SE 0.07	0.32 SE 0.18
(O−E)/V	−88.6/77.9	−12.0/10.6

Any first recurrence

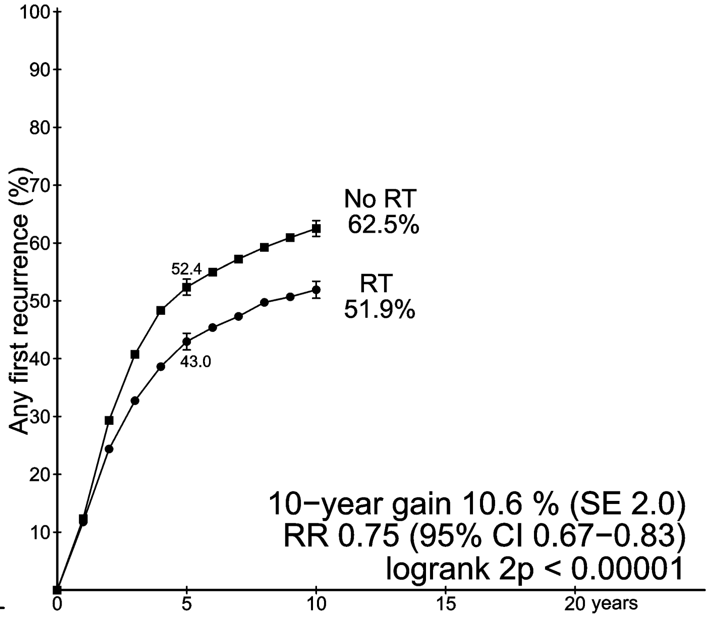

Any first recurrence rates (%/year) and logrank analyses

	Years 0−4	Years 5−9
RT	12.44 (677/5441)	3.88 (125/3221)
No RT	15.19 (796/5241)	5.10 (144/2822)
Rate ratio, from	0.76 SE 0.05	0.72 SE 0.11
(O−E)/V	−80.1/286.4	−18.3/55.8

Breast cancer mortality

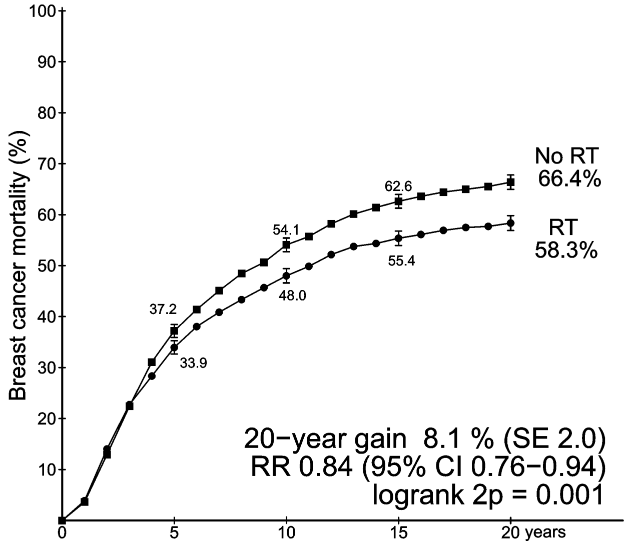

Breast cancer mortality rates (%/year) and logrank analyses

	Years 0−4	Years 5−9	Years 10−14	Years 15−19	Years 20+
RT	8.38 (520/6205)	5.02 (196/3905)	3.26 (85/2608)	1.23 (19/1544)	1.17 (12/1022)
No RT	8.81 (562/6382)	6.14 (233/3796)	4.09 (99/2420)	2.47 (33/1339)	1.62 (14/864)
Rate ratio, from	0.91 SE 0.06	0.77 SE 0.09	0.76 SE 0.14	0.67 SE 0.24	0.77 SE 0.37
(O−E)/V	−21.7/222.2	−23.5/89.3	−10.7/38.5	−4.6/11.4	−1.4/5.6

Any death

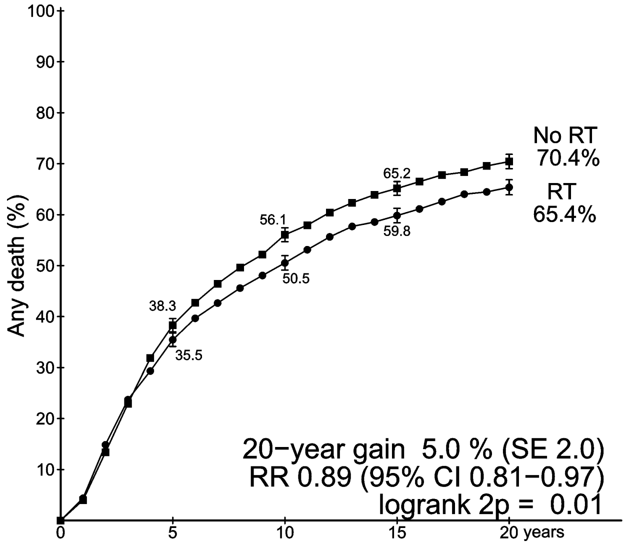

Any death rates (%/year) and logrank analyses

	Years 0−4	Years 5−9	Years 10−14	Years 15−19	Years 20+
RT	9.01 (559/6205)	5.89 (230/3905)	4.72 (123/2608)	3.17 (49/1544)	3.91 (40/1022)
No RT	9.35 (597/6382)	6.96 (264/3796)	5.00 (121/2420)	3.96 (53/1339)	4.40 (38/864)
Rate ratio, from	0.92 SE 0.06	0.79 SE 0.09	0.91 SE 0.13	0.94 SE 0.21	0.85 SE 0.23
(O−E)/V	−19.1/239.0	−24.4/103.7	−4.8/51.4	−1.3/20.8	−2.5/15.6

Webfigure 5. Effect of radiotherapy (RT) to the chest wall and regional lymph nodes versus not after mastectomy and axillary dissection (Mast+AD): 10-year risk of recurrence and type of first recurrence, by allocated treatment, in 3131 women with pathologically node-positive (pN+) disease. (r_L = number of women for whom first recurrence was locoregional, r_D = number women for whom distant recurrence was first.)

3131 pN+ women with Mast+AD

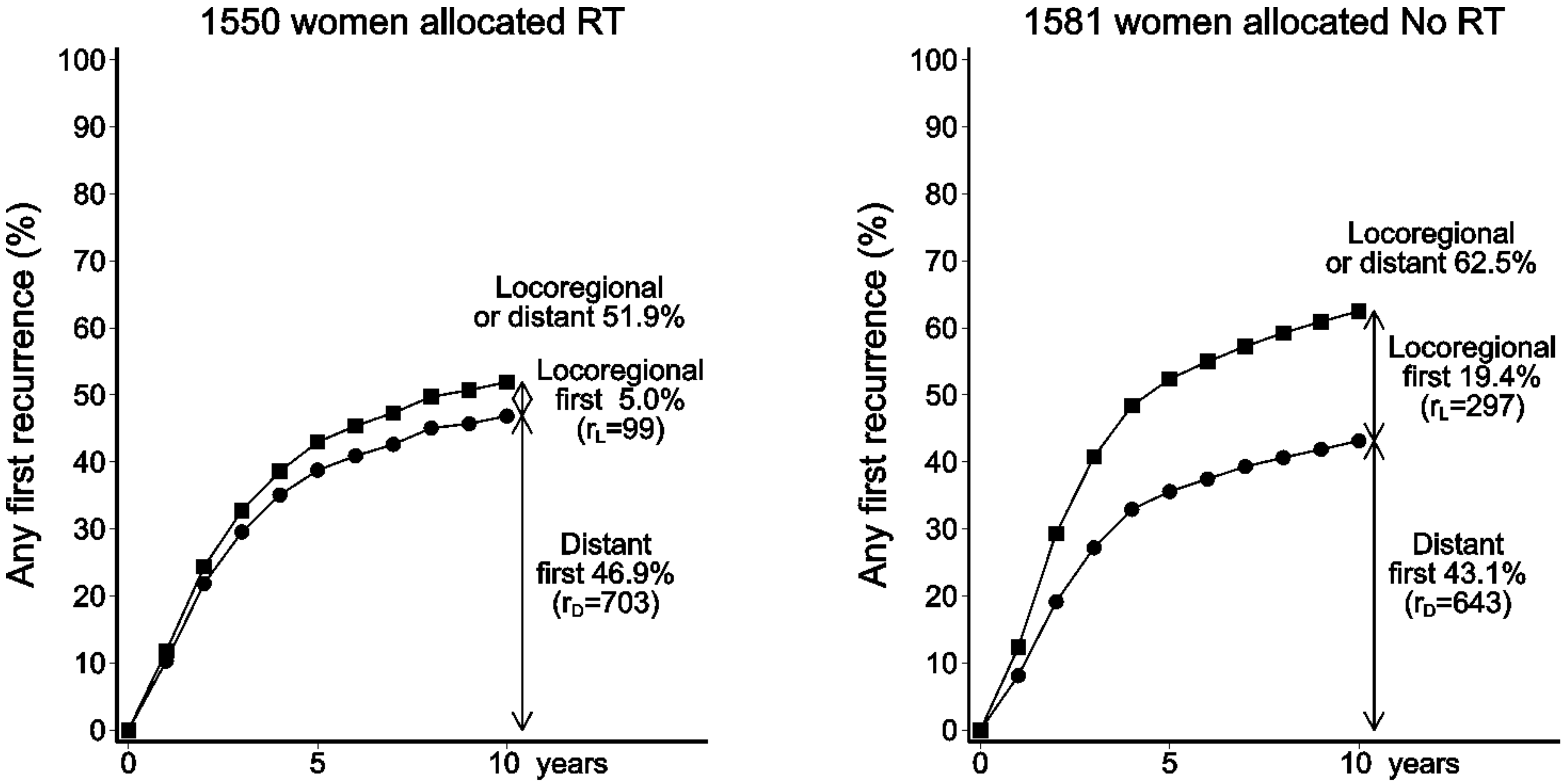

2p for difference between treatment arms in the proportion of all first recurrences that were locoregional: < 0.00001

Webfigure 6. Effect of radiotherapy (RT) to the chest wall and regional lymph nodes versus not after mastectomy and axillary dissection (Mast+AD): Event rate ratios and 95% confidence intervals for locoregional recurrence and recurrence of any type during years 0-9 and for breast cancer mortality in 3131 women with pathologically node-positive (pN+) disease by prognostic and other factors. Categories with unknowns are excluded from the heterogeneity and trend tests.

3131 pN+ women with Mast+AD

Locoregional recurrence first (years 0-9)

Category	Events/Women: Allocated RT	Events/Women: Allocated No RT	RT events: Logrank O−E	RT events: Variance of O−E	Rate Ratio (Standard Error)
**(a) Age at entry (χ^2_1 = 0.7; 2p = 0.4)**					
Age < 40 yrs	19/192	37/185	−12.1	11.4	0.35 (SE 0.18)
Age 40 − 49 yrs	27/452	77/485	−23.6	24.1	0.38 (SE 0.13)
Age 50 − 59 yrs	31/490	100/465	−36.5	29.9	0.30 (SE 0.11)
Age 60+ yrs	22/385	76/403	−28.3	23.1	0.29 (SE 0.12)
**(b) Tumour Grade (χ^2_1 = 0.1; 2p = 0.8)**					
Low grade	7/100	15/86	−4.6	4.2	0.33 (SE 0.30)
Intermediate grade	8/188	56/199	−24.6	14.1	0.17 (SE 0.13)
High grade	8/136	35/139	−11.6	9.6	0.30 (SE 0.19)
Unknown grade	76/1095	184/1114	−55.7	58.3	0.38 (SE 0.08)
**(c) Tumour size (χ^2_1 = 0.0; 2p = 0.9)**					
1−19 mm	10/234	48/249	−18.5	13.5	0.25 (SE 0.15)
20−49 mm	25/381	94/391	−36.7	26.4	0.25 (SE 0.11)
50+ mm	9/151	37/160	−11.4	8.6	0.27 (SE 0.19)
unknown	55/753	111/738	−29.7	36.1	0.44 (SE 0.11)
**(d) Mastectomy (χ^2_1 = 0.1; 2p = 0.8)**					
Simple	90/1309	259/1332	−89.1	79.7	0.33 (SE 0.07)
More extensive	9/210	31/206	−10.3	8.4	0.29 (SE 0.20)
**(e) Axillary surgery information (χ^2_2 = 5.6; p = 0.06)**					
Trial protocol	30/502	77/495	−24.5	23.5	0.35 (SE 0.13)
Trial median	54/667	125/667	−39.4	41.3	0.39 (SE 0.10)
Individual	15/350	88/376	−36.6	23.6	0.21 (SE 0.10)
**(f) Nodal status (χ^2_1 = 4.7; 2p = 0.03)**					
pN1−3	19/625	112/669	−44.5	30.9	0.24 (SE 0.10)
pN4+	78/869	172/849	−53.8	56.4	0.39 (SE 0.09)
pN?+	2/25	6/20	−2.2	1.1	0.14 (SE 0.41)
**(g) Any systemic therapy (χ^2_1 = 4.8; 2p = 0.03)**					
No systemic	1/149	23/127	−10.9	5.3	0.13 (SE 0.18)
Chemo and/or ER+tam+	98/1370	267/1411	−89.1	82.7	0.34 (SE 0.07)
**(h) Radiotherapy dose (χ^2_1 = 0.5; 2p = 0.5)**					
50+ Gy	35/548	123/593	−44.1	35.9	0.29 (SE 0.10)
<50 Gy	64/971	167/945	−56.5	52.5	0.34 (SE 0.08)
**(i) Date trial started (χ^2_1 = 3.0; 2p = 0.08)**					
Started <1980	68/961	160/953	−51.1	52.1	0.38 (SE 0.09)
Started 1980+	31/558	130/585	−49.4	36.4	0.26 (SE 0.09)
**(j) Period of follow−up (χ^2_1 = 0.0; 2p = 1.0)**					
Years 0−4	86/5335	256/5075	−88.6	77.9	0.32 (SE 0.07)
Years 5−9	13/3154	34/2714	−12.0	10.6	0.32 (SE 0.18)
**Total**	**99/ 1519 (6.5%)**	**290/ 1538 (18.9%)**	**−100.5**	**88.5**	**0.32 (SE 0.06)** 2p < 0.00001

Global heterogeneity: χ^2_{15} = 22.1; p > 0.1: NS

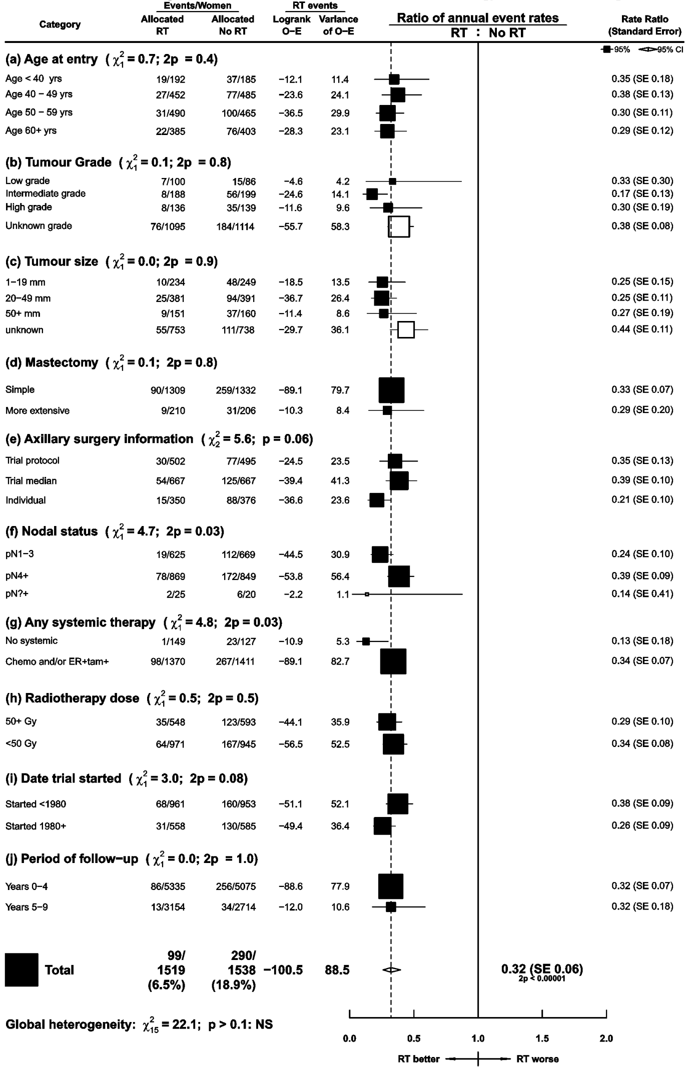

Any first recurrence (years 0-9)

Category	Events/Women: Allocated RT	Events/Women: Allocated No RT	RT events: Logrank O−E	RT events: Variance of O−E	Rate Ratio (Standard Error)
**(a) Age at entry (χ^2_1 = 0.6; 2p = 0.5)**					
Age < 40 yrs	113/196	126/194	−11.4	41.4	0.76 (SE 0.14)
Age 40 − 49 yrs	209/461	262/496	−23.9	98.2	0.78 (SE 0.09)
Age 50 − 59 yrs	273/500	296/481	−28.3	110.6	0.77 (SE 0.08)
Age 60+ yrs	207/393	256/410	−34.8	91.9	0.68 (SE 0.09)
**(b) Tumour Grade (χ^2_1 = 0.0; 2p = 0.9)**					
Low grade	42/102	40/86	−2.2	14.2	0.86 (SE 0.25)
Intermediate grade	96/188	139/199	−24.2	43.1	0.57 (SE 0.12)
High grade	86/136	101/139	−9.0	32.7	0.76 (SE 0.15)
Unknown grade	578/1124	660/1157	−55.4	238.5	0.79 (SE 0.06)
**(c) Tumour size (χ^2_1 = 3.0; 2p = 0.08)**					
1−19 mm	87/239	139/259	−23.7	43.0	0.58 (SE 0.12)
20−49 mm	239/399	261/415	−19.4	95.0	0.82 (SE 0.09)
50+ mm	99/159	119/169	−5.8	33.4	0.84 (SE 0.16)
unknown	377/753	421/738	−40.2	151.8	0.77 (SE 0.07)
**(d) Mastectomy (χ^2_1 = 0.9; 2p = 0.3)**					
Simple	700/1340	828/1375	−82.2	302.1	0.76 (SE 0.05)
More extensive	102/210	112/206	−15.6	35.6	0.65 (SE 0.14)
**(e) Axillary surgery information (χ^2_2 = 0.5; p = 0.8)**					
Trial protocol	245/502	264/495	−23.1	100.0	0.79 (SE 0.09)
Trial median	338/667	398/667	−46.0	146.9	0.73 (SE 0.07)
Individual	219/381	278/419	−29.3	95.2	0.74 (SE 0.09)
**(f) Nodal status (χ^2_1 = 1.5; 2p = 0.2)**					
pN1−3	211/632	304/682	−42.3	111.7	0.68 (SE 0.08)
pN4+	575/893	624/879	−54.1	226.4	0.79 (SE 0.06)
pN?+	16/25	12/20	−2.1	4.1	0.60 (SE 0.39)
**(g) Any systemic therapy (χ^2_1 = 0.2; 2p = 0.6)**					
No systemic	81/149	78/127	−10.5	27.9	0.69 (SE 0.16)
Chemo and/or ER+tam+	721/1401	862/1454	−87.6	312.5	0.76 (SE 0.05)
**(h) Radiotherapy dose (χ^2_1 = 0.4; 2p = 0.5)**					
50+ Gy	314/579	406/636	−45.3	137.1	0.72 (SE 0.07)
<50 Gy	488/971	534/945	−53.1	205.0	0.77 (SE 0.06)
**(i) Date trial started (χ^2_1 = 0.6; 2p = 0.4)**					
Started <1980	486/992	575/996	−66.6	207.2	0.73 (SE 0.06)
Started 1980+	316/558	365/585	−31.8	135.0	0.79 (SE 0.08)
**(j) Period of follow−up (χ^2_1 = 0.1; 2p = 0.7)**					
Years 0−4	677/5441	796/5241	−80.1	286.4	0.76 (SE 0.05)
Years 5−9	125/3221	144/2822	−18.3	55.8	0.72 (SE 0.11)
**Total**	**802/ 1550 (51.7%)**	**940/ 1581 (59.5%)**	**−98.4**	**342.1**	**0.75 (SE 0.05)** 2p < 0.00001

Global heterogeneity: χ^2_{15} = 11.8; p > 0.1: NS

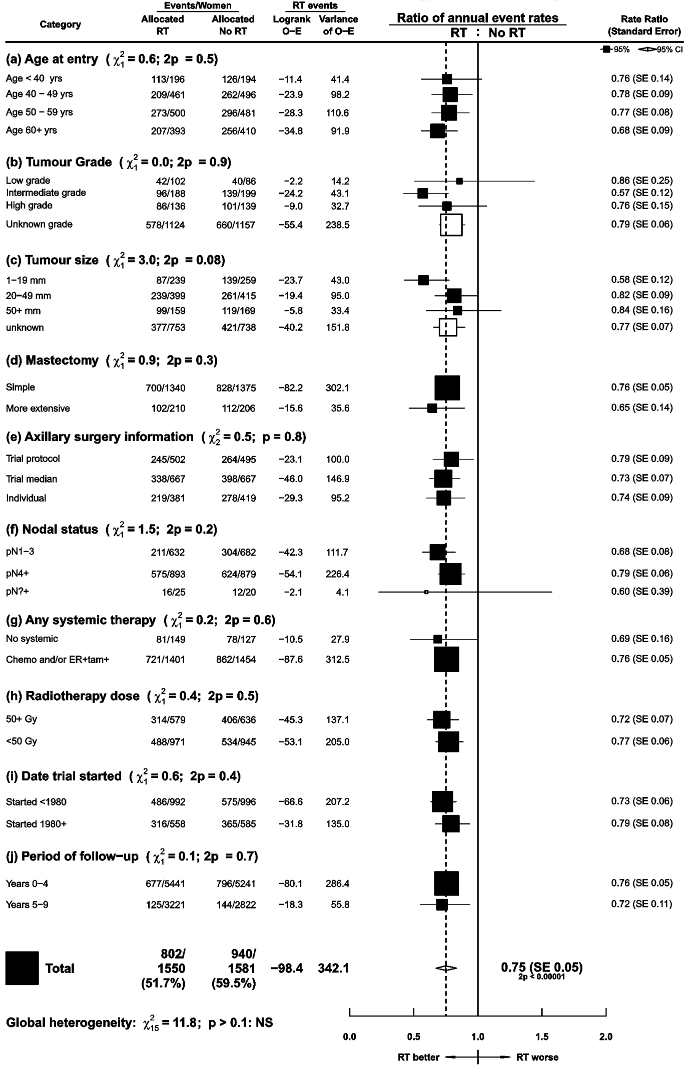

Breast cancer mortality

Category	Deaths/Women: Allocated RT	Deaths/Women: Allocated No RT	RT deaths: Logrank O−E	RT deaths: Variance of O−E	Rate Ratio (Standard Error)
**(a) Age at entry (χ^2_1 = 1.8; 2p = 0.2)**					
Age < 40 yrs	113/196	125/194	−12.1	45.9	0.77 (SE 0.13)
Age 40 − 49 yrs	213/461	274/496	−26.8	107.3	0.78 (SE 0.09)
Age 50 − 59 yrs	284/500	287/481	−14.7	117.0	0.88 (SE 0.09)
Age 60+ yrs	222/393	255/410	−8.4	96.8	0.92 (SE 0.10)
**(b) Tumour Grade (χ^2_1 = 0.1; 2p = 0.7)**					
Low grade	47/102	44/86	−2.0	16.8	0.89 (SE 0.23)
Intermediate grade	102/188	140/199	−14.8	49.6	0.74 (SE 0.12)
High grade	88/136	99/139	−2.9	34.8	0.92 (SE 0.16)
Unknown grade	595/1124	658/1157	−36.5	250.1	0.86 (SE 0.06)
**(c) Tumour size (χ^2_1 = 1.1; 2p = 0.3)**					
1−19 mm	106/239	146/259	−14.7	49.4	0.74 (SE 0.12)
20−49 mm	251/399	262/415	−2.0	106.6	0.98 (SE 0.10)
50+ mm	101/159	123/169	−3.9	37.5	0.90 (SE 0.16)
unknown	374/753	410/738	−30.5	152.9	0.82 (SE 0.07)
**(d) Mastectomy (χ^2_1 = 0.1; 2p = 0.8)**					
Simple	716/1340	826/1375	−54.1	322.0	0.85 (SE 0.05)
More extensive	116/210	115/206	−8.6	40.9	0.81 (SE 0.14)
**(e) Axillary surgery information (χ^2_2 = 0.5; p = 0.8)**					
Trial protocol	258/502	268/495	−18.7	104.3	0.84 (SE 0.09)
Trial median	344/667	395/667	−31.5	157.6	0.82 (SE 0.07)
Individual	230/381	278/419	−11.8	105.1	0.89 (SE 0.09)
**(f) Nodal status (χ^2_1 = 0.7; 2p = 0.4)**					
pN1−3	248/632	325/682	−28.4	125.9	0.80 (SE 0.08)
pN4+	567/893	605/879	−31.7	237.2	0.87 (SE 0.06)
pN?+	17/25	11/20	−1.9	3.9	0.61 (SE 0.40)
**(g) Any systemic therapy (χ^2_1 = 0.0; 2p = 0.9)**					
No systemic	96/149	89/127	−6.5	33.1	0.82 (SE 0.16)
Chemo and/or ER+tam+	736/1401	852/1454	−53.8	330.6	0.85 (SE 0.05)
**(h) Radiotherapy dose (χ^2_1 = 0.5; 2p = 0.5)**					
50+ Gy	317/579	384/636	−18.0	143.7	0.88 (SE 0.08)
<50 Gy	515/971	557/945	−44.0	223.3	0.82 (SE 0.06)
**(i) Date trial started (χ^2_1 = 2.3; 2p = 0.1)**					
Started <1980	500/992	576/996	−51.0	218.4	0.79 (SE 0.06)
Started 1980+	332/558	365/585	−10.9	148.6	0.93 (SE 0.08)
**(j) Period of follow−up (χ^2_1 = 2.4; 2p = 0.1)**					
Years 0−4	520/6205	562/6382	−21.7	222.2	0.91 (SE 0.06)
Years 5−9	196/3905	233/3796	−23.5	89.3	0.77 (SE 0.09)
Years 10−14	85/2608	99/2420	−10.7	38.5	0.76 (SE 0.14)
Years 15−19	19/1544	33/1339	−4.6	11.4	0.67 (SE 0.24)
Years 20+	12/1022	14/864	−1.4	5.6	0.78 (SE 0.37)
**Total**	**832/ 1550 (53.7%)**	**941/ 1581 (59.5%)**	**−62.0**	**367.0**	**0.84 (SE 0.05)** 2p = 0.001

Global heterogeneity: χ^2_{18} = 12.7; p > 0.1: NS

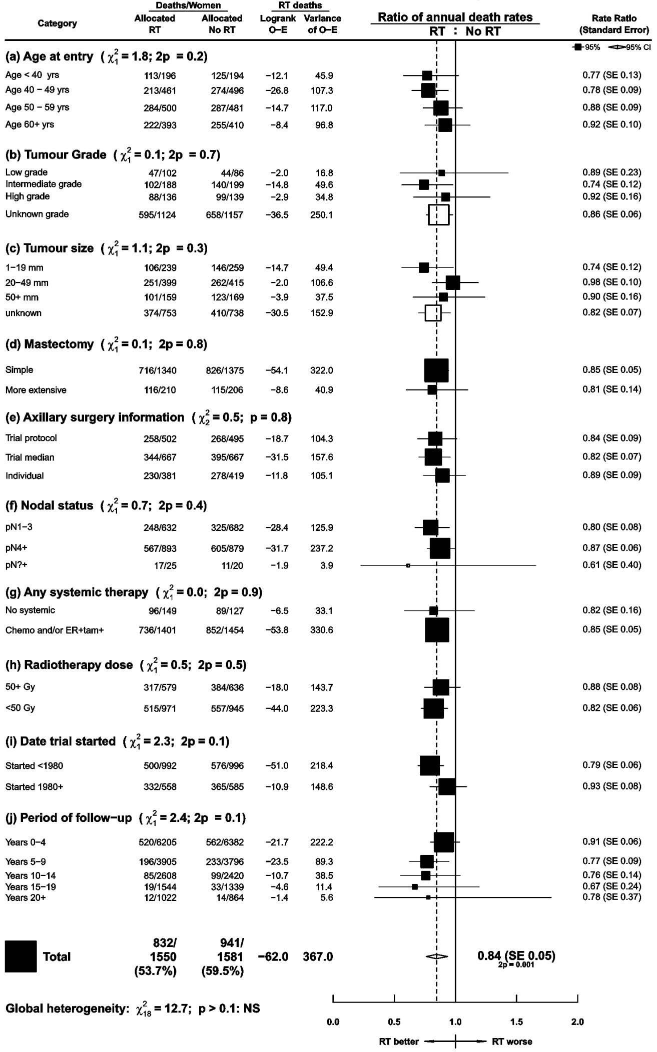

Note:In (g), 181 women who were ER positive with tamoxifen also had chemotherapy. In (h), trials that used orthovoltage irradiation are included in the <50 Gy category.

Webfigure 7. Effect of radiotherapy (RT) to the chest wall and regional lymph nodes versus not after mastectomy and axillary dissection (Mast+AD): 10-year risk of locoregional recurrence and recurrence of any type and 20-year risk of breast cancer and all-cause mortality in 1314 women with 1-3 pathologically positive nodes (pN1-3). See webfigure 1 for methodological note and also webfigure 8.

1314 pN1-3 women with Mast+AD

Locoregional recurrence first

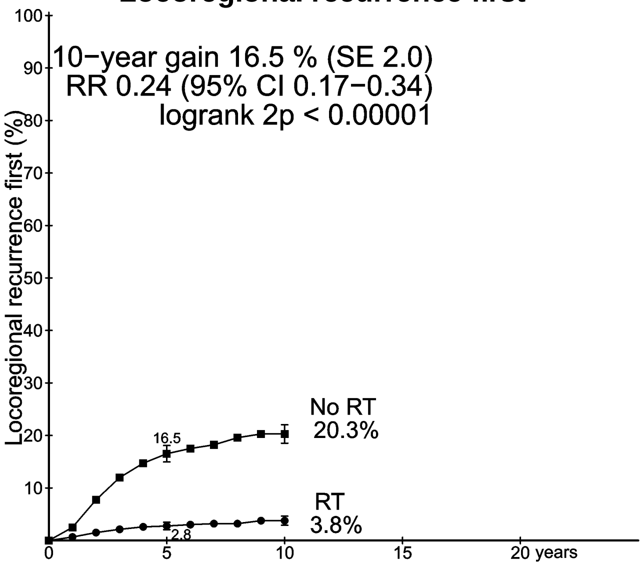

Locoregional recurrence first rates (%/year) and logrank analyses

	Years 0−4	Years 5−9
RT	0.58 (15/2588)	0.21 (4/1876)
No RT	3.61 (95/2633)	0.97 (17/1745)
Rate ratio, from	0.23 SE 0.10	0.28 SE 0.25
(O−E)/V	−38.1/25.8	−6.5/5.1

Any first recurrence

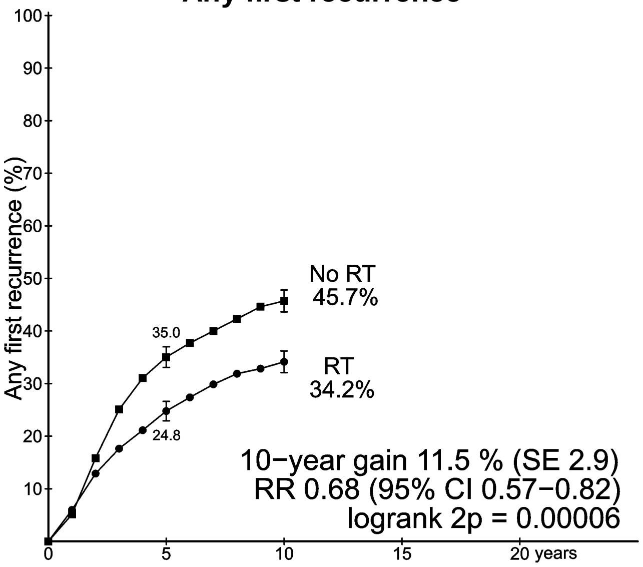

Any first recurrence rates (%/year) and logrank analyses

	Years 0−4	Years 5−9
RT	6.00 (157/2616)	2.85 (54/1898)
No RT	8.75 (235/2686)	3.87 (69/1782)
Rate ratio, from	0.67 SE 0.09	0.75 SE 0.17
(O−E)/V	−34.5/84.9	−7.8/26.7

Breast cancer mortality

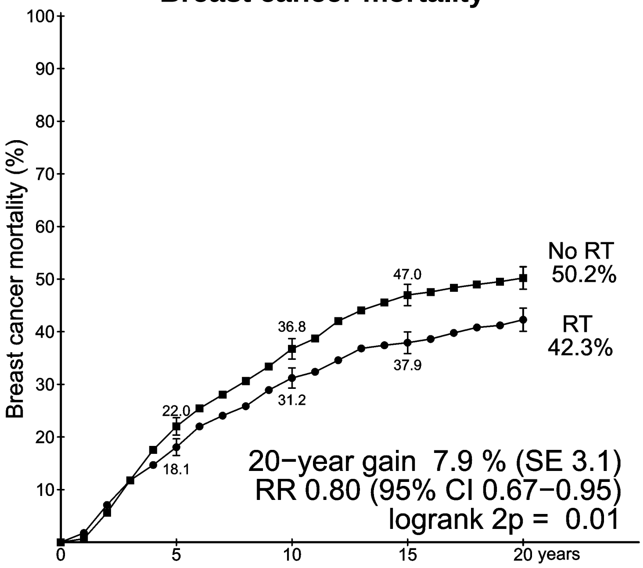

Breast cancer mortality rates (%/year) and logrank analyses

	Years 0−4	Years 5−9	Years 10−14	Years 15−19	Years 20+
RT	3.93 (111/2822)	3.50 (76/2172)	2.12 (35/1648)	1.39 (15/1080)	1.29 (11/850)
No RT	4.83 (148/3064)	4.17 (94/2255)	3.56 (58/1630)	1.32 (13/986)	1.59 (12/757)
Rate ratio, from	0.82 SE 0.12	0.84 SE 0.15	0.59 SE 0.17	1.17 SE 0.44	0.88 SE 0.42
(O−E)/V	−11.2/56.7	−6.5/37.4	−11.0/20.7	0.9/6.1	−0.6/5.0

Any death

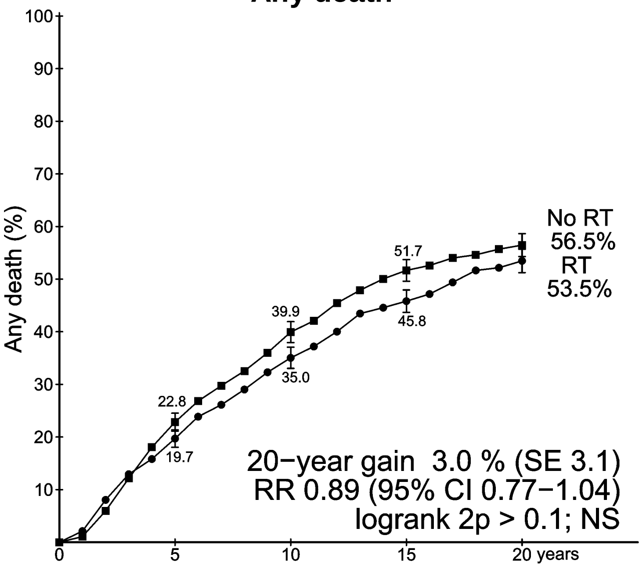

Any death rates (%/year) and logrank analyses

	Years 0−4	Years 5−9	Years 10−14	Years 15−19	Years 20+
RT	4.50 (127/2822)	4.37 (95/2172)	3.76 (62/1648)	3.06 (33/1080)	4.12 (35/850)
No RT	5.22 (160/3064)	5.06 (114/2255)	4.48 (73/1630)	2.64 (26/986)	4.49 (34/757)
Rate ratio, from	0.86 SE 0.12	0.85 SE 0.14	0.83 SE 0.17	1.51 SE 0.36	0.90 SE 0.25
(O−E)/V	−9.2/63.2	−7.5/46.4	−5.5/30.3	5.0/12.1	−1.4/13.9

Webfigure 8. Effect of radiotherapy (RT) to the chest wall and regional lymph nodes versus not after mastectomy and axillary dissection (Mast+AD): 10-year risk of recurrence and type of first recurrence, by allocated treatment, in 1314 women with 1-3 pathologically positive nodes (pN1-3). (r_L = number of women for whom first recurrence was locoregional, r_D = number women for whom distant recurrence was first.)

1314 pN1-3 women with Mast+AD

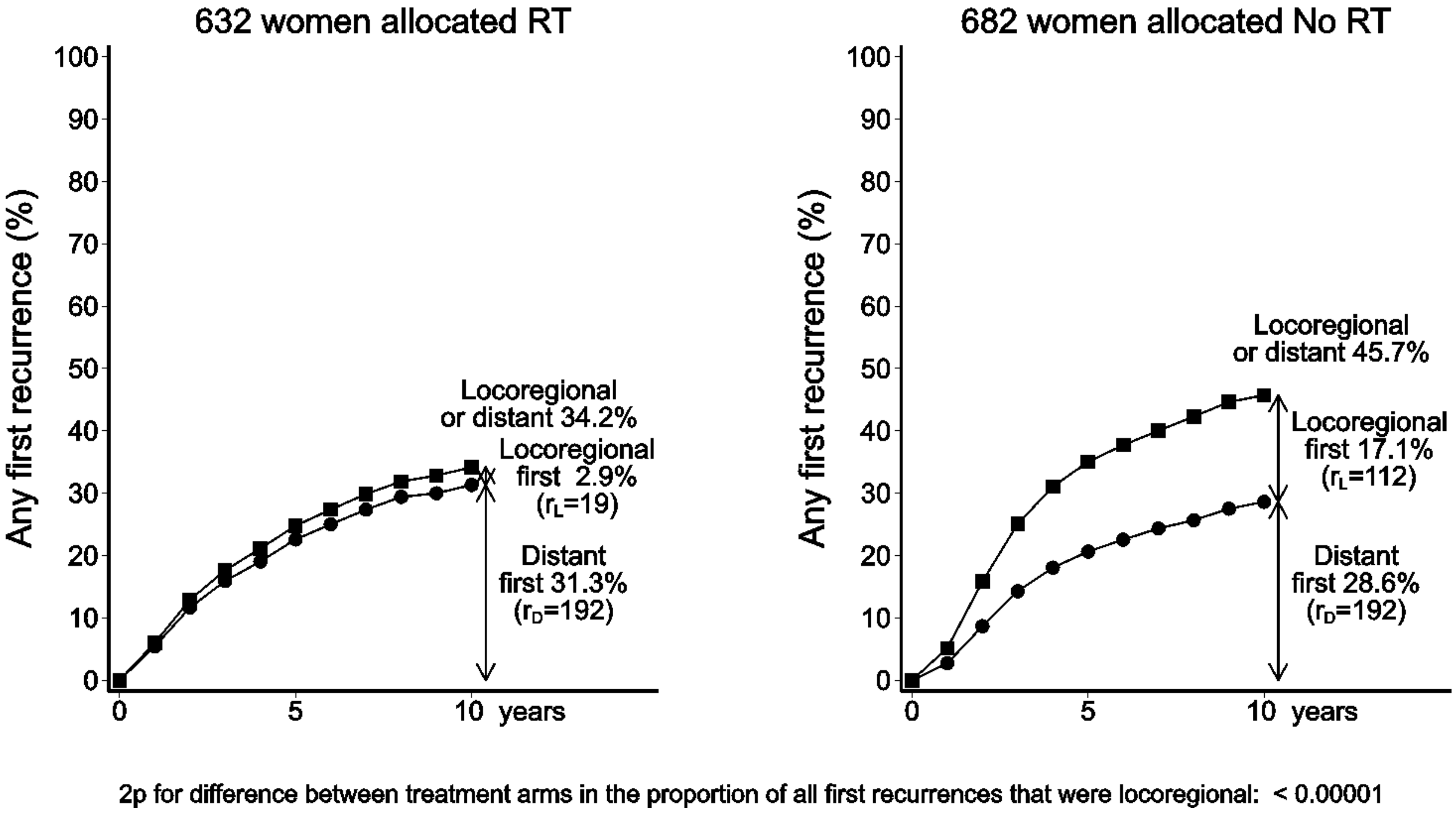

Webfigure 9. Effect of radiotherapy (RT) to the chest wall and regional lymph nodes versus not after mastectomy and axillary dissection (Mast+AD): Event rate ratios and 95% confidence intervals for locoregional recurrence and recurrence of any type during years 0-9 and for breast cancer mortality in 1314 women with 1-3 pathologically positive nodes (pN1-3) by prognostic and other factors. Categories with unknowns are excluded from the heterogeneity and trend tests.

1314 pN1-3 women with Mast+AD

Locoregional recurrence first (years 0-9)

Category	Events/Women: Allocated RT	Events/Women: Allocated No RT	RT events: Logrank O−E	RT events: Variance of O−E	Rate Ratio (Standard Error)
**(a) Age at entry** (χ^2_1 = 0.9; 2p = 0.3)					
Age < 40 yrs	5/75	20/74	−8.0	5.6	0.24 (SE 0.22)
Age 40 − 49 yrs	6/203	19/211	−6.3	6.1	0.36 (SE 0.25)
Age 50 − 59 yrs	7/200	40/199	−15.8	11.1	0.24 (SE 0.16)
Age 60+ yrs	1/147	33/185	−14.5	8.1	0.17 (SE 0.16)
**(b) Tumour Grade** (χ^2_1 = 0.0; 2p = 0.9)					
Low grade	4/64	7/48	−2.5	2.2	0.32 (SE 0.40)
Intermediate grade	4/81	21/95	−7.5	5.5	0.26 (SE 0.23)
High grade	1/50	9/57	−3.0	2.3	0.27 (SE 0.37)
Unknown grade	10/430	75/469	−30.5	19.7	0.21 (SE 0.11)
**(c) Tumour size** (χ^2_1 = 0.0; 2p = 0.9)					
1−19 mm	4/138	26/148	−10.4	7.0	0.23 (SE 0.20)
20−49 mm	5/148	37/187	−13.6	9.6	0.24 (SE 0.17)
50+ mm	2/32	5/28	−2.1	1.1	0.15 (SE 0.43)
unknown	8/307	44/306	−17.1	12.0	0.24 (SE 0.15)
**(d) Mastectomy** (χ^2_1 = 0.3; 2p = 0.6)					
Simple	18/507	95/544	−37.4	26.6	0.25 (SE 0.10)
More extensive	1/118	17/125	−7.2	4.3	0.19 (SE 0.23)
**(e) Axillary surgery information** (χ^2_2 = 1.1; p = 0.6)					
Trial protocol	8/277	42/281	−16.6	11.8	0.24 (SE 0.16)
Trial median	9/212	38/234	−14.4	11.4	0.28 (SE 0.17)
Individual	2/136	32/154	−13.6	7.8	0.17 (SE 0.17)
**(f) Number of positive nodes** (χ^2_1 = 0.2; 2p = 0.7)					
1 positive node	3/189	34/209	−14.2	8.8	0.20 (SE 0.17)
2−3 positive nodes	8/218	35/222	−13.1	9.2	0.24 (SE 0.18)
Unknown but pN1−3	8/218	43/238	−17.2	11.7	0.23 (SE 0.15)
**(g) Any systemic therapy** (χ^2_1 = 1.1; 2p = 0.3)					
No systemic	0/93	12/88	−5.7	2.[illegible]	0.00 (SE 0.26)
Chemo and/or ER+tam+	19/532	100/581	−38.9	28.0	0.25 (SE 0.10)
**(h) Radiotherapy dose** (χ^2_1 = 0.9; 2p = 0.4)					
50+ Gy	3/173	43/203	−17.7	10.6	0.19 (SE 0.15)
<50 Gy	16/452	69/466	−26.8	20.3	0.27 (SE 0.12)
**(i) Date trial started** (χ^2_1 = 0.4; 2p = 0.5)					
Started <1980	15/426	68/441	−26.8	19.8	0.26 (SE 0.12)
Started 1980+	4/199	44/228	−17.8	11.1	0.20 (SE 0.15)
**(j) Period of follow−up** (χ^2_1 = 0.2; 2p = 0.7)					
Years 0−4	15/2588	95/2633	−38.1	25.8	0.23 (SE 0.10)
Years 5−9	4/1876	17/1745	−6.5	5.1	0.28 (SE 0.25)
**Total**	19/625 (3.0%)	112/669 (16.7%)	−44.6	30.9	0.24 (SE 0.10) 2p < 0.00001

Global heterogeneity: χ^2_{15} = 6.4; p > 0.1: NS

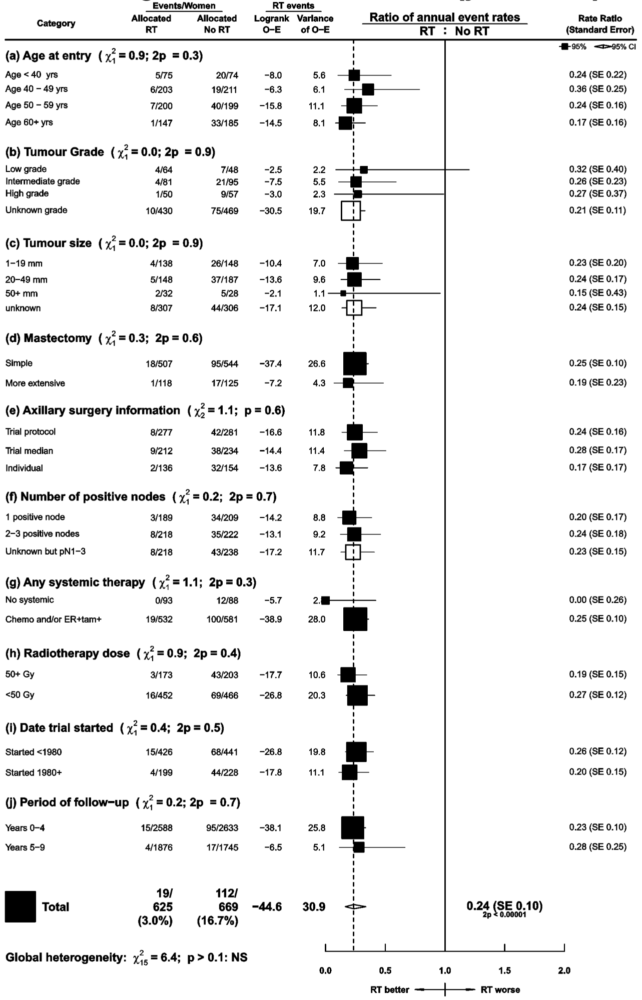

Any first recurrence (years 0-9)

Category	Events/Women: Allocated RT	Events/Women: Allocated No RT	RT events: Logrank O−E	RT events: Variance of O−E	Rate Ratio (Standard Error)
**(a) Age at entry** (χ^2_1 = 0.0; 2p = 0.9)					
Age < 40 yrs	27/75	43/76	−9.1	13.8	0.52 (SE 0.20)
Age 40 − 49 yrs	59/204	72/217	−3.5	29.1	0.89 (SE 0.17)
Age 50 − 59 yrs	74/205	99/203	−16.1	38.0	0.65 (SE 0.13)
Age 60+ yrs	51/148	90/186	−13.6	30.7	0.64 (SE 0.15)
**(b) Tumour Grade** (χ^2_1 = 0.2; 2p = 0.6)					
Low grade	17/64	14/48	−0.9	6.2	0.86 (SE 0.37)
Intermediate grade	26/81	45/95	−8.5	15.0	0.57 (SE 0.20)
High grade	18/50	29/57	−4.3	9.5	0.64 (SE 0.26)
Unknown grade	150/437	216/482	−28.5	77.0	0.69 (SE 0.10)
**(c) Tumour size** (χ^2_1 = 3.1; 2p = 0.08)					
1−19 mm	27/139	60/153	−15.9	18.4	0.42 (SE 0.16)
20−49 mm	63/151	88/192	−4.1	31.0	0.88 (SE 0.17)
50+ mm	12/35	17/31	−2.5	4.9	0.60 (SE 0.35)
unknown	109/307	139/306	−17.8	52.8	0.71 (SE 0.12)
**(d) Mastectomy** (χ^2_1 = 0.0; 2p = 0.9)					
Simple	172/514	245/557	−33.5	90.2	0.69 (SE 0.09)
More extensive	39/118	59/125	−8.2	21.1	0.68 (SE 0.18)
**(e) Axillary surgery information** (χ^2_2 = 0.2; p = 0.9)					
Trial protocol	97/277	127/281	−16.7	49.8	0.72 (SE 0.12)
Trial median	69/212	104/234	−15.8	37.3	0.65 (SE 0.13)
Individual	45/143	73/167	−9.8	24.5	0.67 (SE 0.17)
**(f) Number of positive nodes** (χ^2_1 = 1.1; 2p = 0.3)					
1 positive node	48/191	80/214	−13.8	27.8	0.61 (SE 0.15)
2−3 positive nodes	89/223	116/230	−9.8	41.3	0.79 (SE 0.14)
Unknown but pN1−3	74/218	108/238	−18.5	38.4	0.62 (SE 0.13)
**(g) Any systemic therapy** (χ^2_1 = 0.4; 2p = 0.5)					
No systemic	34/93	42/88	−4.1	16.8	0.78 (SE 0.22)
Chemo and/or ER+tam+	177/539	262/594	−38.2	94.5	0.67 (SE 0.08)
**(h) Radiotherapy dose** (χ^2_1 = 0.2; 2p = 0.6)					
50+ Gy	55/180	97/216	−14.1	31.2	0.64 (SE 0.14)
<50 Gy	156/452	207/466	−28.2	80.4	0.70 (SE 0.09)
**(i) Date trial started** (χ^2_1 = 1.1; 2p = 0.3)					
Started <1980	145/433	208/454	−34.1	76.6	0.64 (SE 0.09)
Started 1980+	66/199	96/228	−8.1	35.1	0.79 (SE 0.15)
**(j) Period of follow−up** (χ^2_1 = 0.3; 2p = 0.6)					
Years 0−4	157/2616	235/2686	−34.5	84.9	0.67 (SE 0.09)
Years 5−9	54/1898	69/1782	−7.8	26.7	0.75 (SE 0.17)
**Total**	211/632 (33.4%)	304/682 (44.6%)	−42.3	111.6	0.68 (SE 0.08) 2p = 0.00006

Global heterogeneity: χ^2_{15} = 13.5; p > 0.1: NS

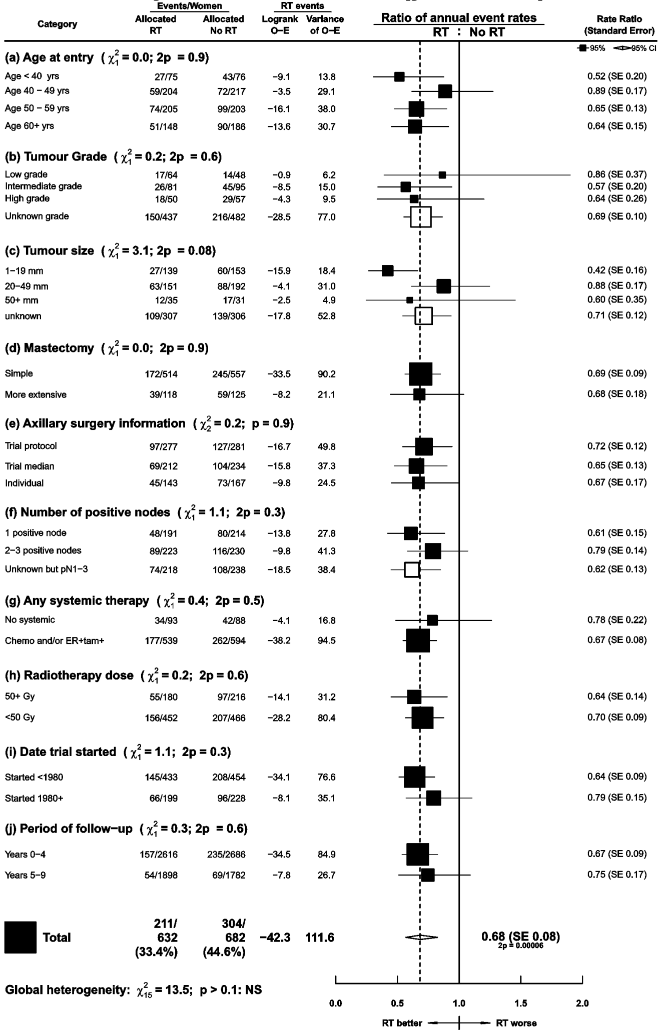

Breast cancer mortality

Category	Deaths/Women: Allocated RT	Deaths/Women: Allocated No RT	RT deaths: Logrank O−E	RT deaths: Variance of O−E	Rate Ratio (Standard Error)
**(a) Age at entry** (χ^2_1 = 1.1; 2p = 0.3)					
Age < 40 yrs	31/75	43/76	−8.6	15.1	0.57 (SE 0.20)
Age 40 − 49 yrs	65/204	81/217	−5.0	33.2	0.86 (SE 0.16)
Age 50 − 59 yrs	84/205	106/203	−11.0	43.6	0.78 (SE 0.13)
Age 60+ yrs	68/148	95/186	−3.8	34.0	0.89 (SE 0.16)
**(b) Tumour Grade** (χ^2_1 = 0.7; 2p = 0.4)					
Low grade	22/64	15/48	1.3	7.4	1.19 (SE 0.40)
Intermediate grade	27/81	48/95	−8.1	16.5	0.61 (SE 0.19)
High grade	20/50	29/57	−3.2	10.5	0.74 (SE 0.27)
Unknown grade	179/437	233/482	−18.1	86.9	0.81 (SE 0.10)
**(c) Tumour size** (χ^2_1 = 0.0; 2p = 0.9)					
1−19 mm	46/139	63/153	−6.4	23.2	0.76 (SE 0.18)
20−49 mm	65/151	90/192	−3.1	33.1	0.91 (SE 0.17)
50+ mm	12/35	17/31	−2.3	5.2	0.64 (SE 0.35)
unknown	125/307	155/306	−13.8	59.3	0.79 (SE 0.12)
**(d) Mastectomy** (χ^2_1 = 0.2; 2p = 0.7)					
Simple	195/514	258/557	−24.1	99.6	0.79 (SE 0.09)
More extensive	53/118	67/125	−3.8	25.9	0.86 (SE 0.18)
**(e) Axillary surgery information** (χ^2_2 = 0.1; p = 1.0)					
Trial protocol	113/277	140/281	−11.5	55.5	0.81 (SE 0.12)
Trial median	83/212	112/234	−11.6	43.9	0.77 (SE 0.13)
Individual	52/143	73/167	−5.3	26.5	0.82 (SE 0.18)
**(f) Number of positive nodes** (χ^2_1 = 0.2; 2p = 0.7)					
1 positive node	67/191	88/214	−7.7	32.4	0.79 (SE 0.16)
2−3 positive nodes	100/223	124/230	−6.5	47.8	0.87 (SE 0.14)
Unknown but pN1−3	81/218	113/238	−11.4	41.5	0.76 (SE 0.14)
**(g) Any systemic therapy** (χ^2_1 = 0.4; 2p = 0.5)					
No systemic	46/93	52/88	−2.1	21.8	0.91 (SE 0.20)
Chemo and/or ER+tam+	202/539	273/594	−25.9	103.7	0.78 (SE 0.09)
**(h) Radiotherapy dose** (χ^2_1 = 0.0; 2p = 1.0)					
50+ Gy	64/180	95/216	−7.5	33.1	0.80 (SE 0.16)
<50 Gy	184/452	230/466	−20.9	92.8	0.80 (SE 0.09)
**(i) Date trial started** (χ^2_1 = 2.0; 2p = 0.2)					
Started <1980	173/433	232/454	−27.2	88.6	0.74 (SE 0.09)
Started 1980+	75/199	93/228	−1.2	37.4	0.97 (SE 0.16)
**(j) Period of follow−up** (χ^2_1 = 0.0; 2p = 0.9)					
Years 0−4	111/2822	148/3064	−11.2	56.7	0.82 (SE 0.12)
Years 5−9	76/2172	94/2255	−6.5	37.4	0.84 (SE 0.15)
Years 10−14	35/1648	58/1630	−11.0	20.7	0.59 (SE 0.17)
Years 15−19	15/1080	13/986	0.9	6.1	1.16 (SE 0.44)
Years 20+	11/850	12/757	−0.6	5.0	0.89 (SE 0.42)
**Total**	248/632 (39.2%)	325/682 (47.7%)	−28.4	125.9	0.80 (SE 0.08) 2p = 0.01

Global heterogeneity: χ^2_{18} = 11.4; p > 0.1: NS

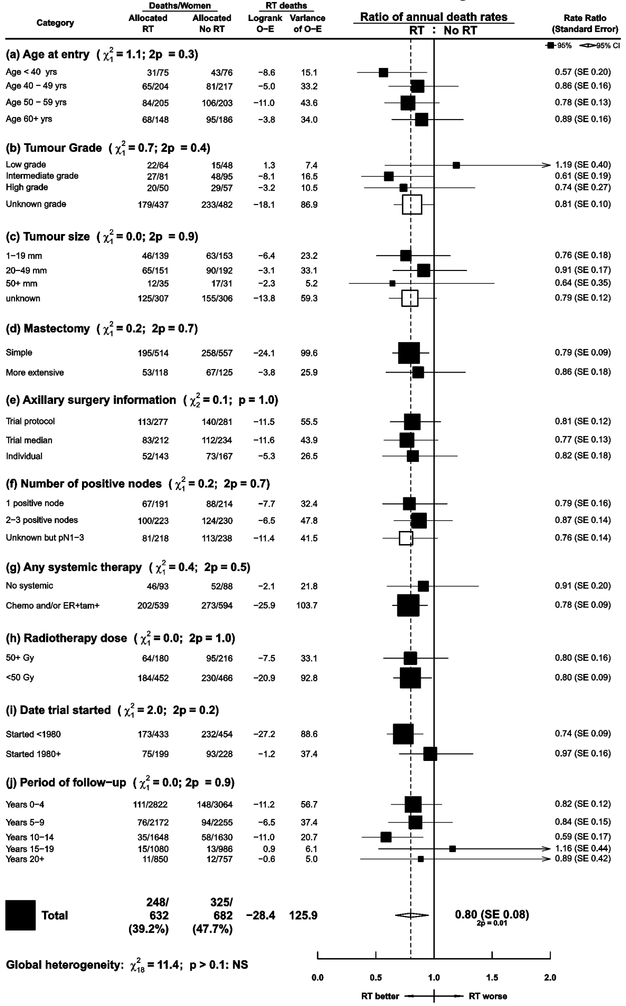

In (h), trials that used orthovoltage irradiation are included in the <50 Gy category.

Webfigure 10. Effect of radiotherapy (RT) to the chest wall and regional lymph nodes versus not after mastectomy and axillary dissection (Mast+AD): 10-year risk of locoregional recurrence and recurrence of any type and 20-year risk of breast cancer and all-cause mortality in 1133 women with 1-3 pathologically positive nodes (pN1-3) in trials where systemic therapy was given to both randomised treatment groups. See webfigure 1 for methodological note and also webfigure 11.

1133 pN1-3 women with Mast+AD and systemic therapy

Locoregional recurrence first

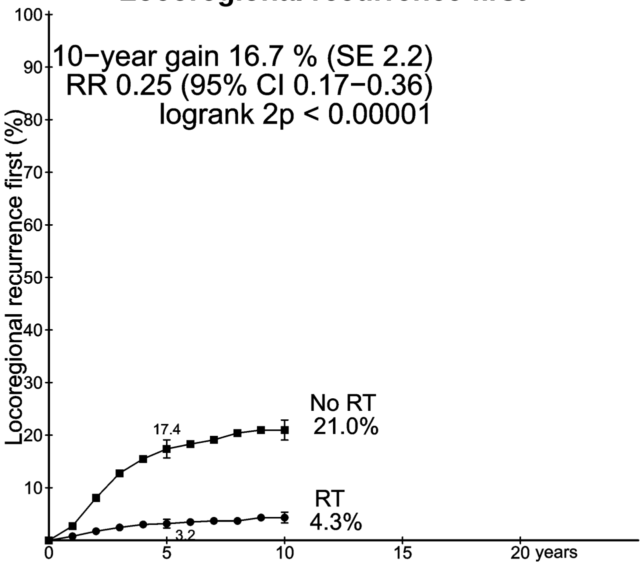

Locoregional recurrence first rates (%/year) and logrank analyses

	Years 0−4	Years 5−9
RT	0.68 (15/2213)	0.25 (4/1599)
No RT	3.80 (86/2264)	0.93 (14/1502)
Rate ratio, from	0.24 SE 0.11	0.31 SE 0.28
(O−E)/V	−33.9/23.7	−5.0/4.3

Any first recurrence

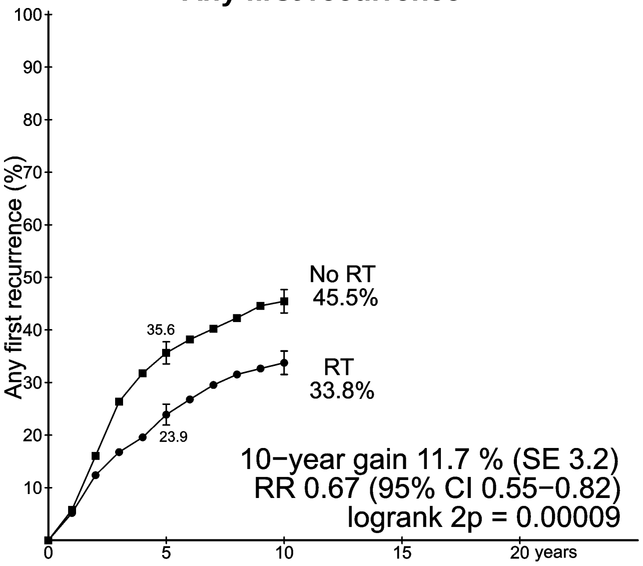

Any first recurrence rates (%/year) and logrank analyses

	Years 0−4	Years 5−9
RT	5.76 (129/2240)	2.96 (48/1621)
No RT	8.93 (207/2318)	3.58 (55/1538)
Rate ratio, from	0.62 SE 0.09	0.85 SE 0.20
(O−E)/V	−34.6/72.3	−3.6/22.2

Breast cancer mortality

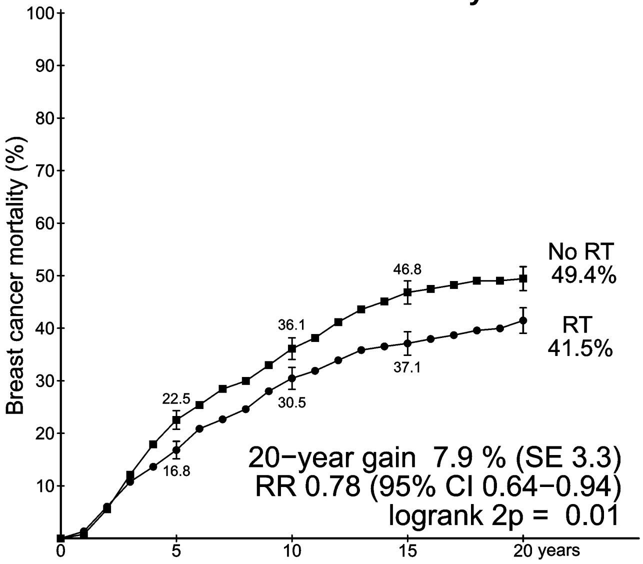

Breast cancer mortality rates (%/year) and logrank analyses

	Years 0−4	Years 5−9	Years 10−14	Years 15−19	Years 20+
RT	3.64 (88/2420)	3.58 (67/1873)	2.06 (29/1406)	1.35 (12/887)	1.22 (6/490)
No RT	4.92 (131/2661)	3.83 (75/1959)	3.70 (52/1406)	1.12 (9/800)	1.35 (6/446)
Rate ratio, from	0.74 SE 0.12	0.94 SE 0.17	0.56 SE 0.18	1.21 SE 0.52	1.16 SE 0.68
(O−E)/V	−14.6/47.5	−2.1/31.1	−10.5/18.1	0.9/4.6	0.4/2.5

Any death

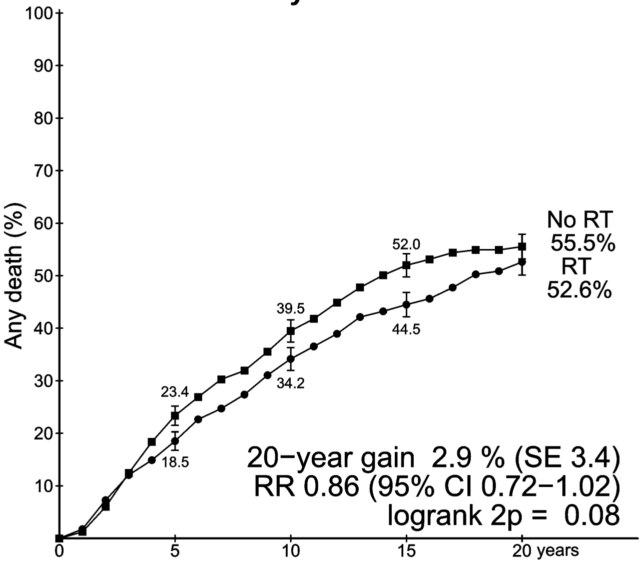

Any death rates (%/year) and logrank analyses

	Years 0−4	Years 5−9	Years 10−14	Years 15−19	Years 20+
RT	4.21 (102/2420)	4.38 (82/1873)	3.56 (50/1406)	3.16 (28/887)	2.45 (12/490)
No RT	5.34 (142/2661)	4.75 (93/1959)	4.77 (67/1406)	2.25 (18/800)	3.14 (14/446)
Rate ratio, from	0.78 SE 0.12	0.90 SE 0.15	0.74 SE 0.17	1.82 SE 0.45	0.84 SE 0.39
(O−E)/V	−13.0/53.2	−4.0/38.6	−7.8/26.3	5.5/9.2	−1.0/5.6

Webfigure 11. Effect of radiotherapy (RT) to the chest wall and regional lymph nodes versus not after mastectomy and axillary dissection (Mast+AD): 10-year risk of recurrence and type of first recurrence, by allocated treatment, in 1133 women with 1-3 pathologically positive nodes (pN1-3) in trials where systemic therapy was given to both randomised treatment groups. (r_L = number of women for whom first recurrence was locoregional, r_D = number women for whom distant recurrence was first.)

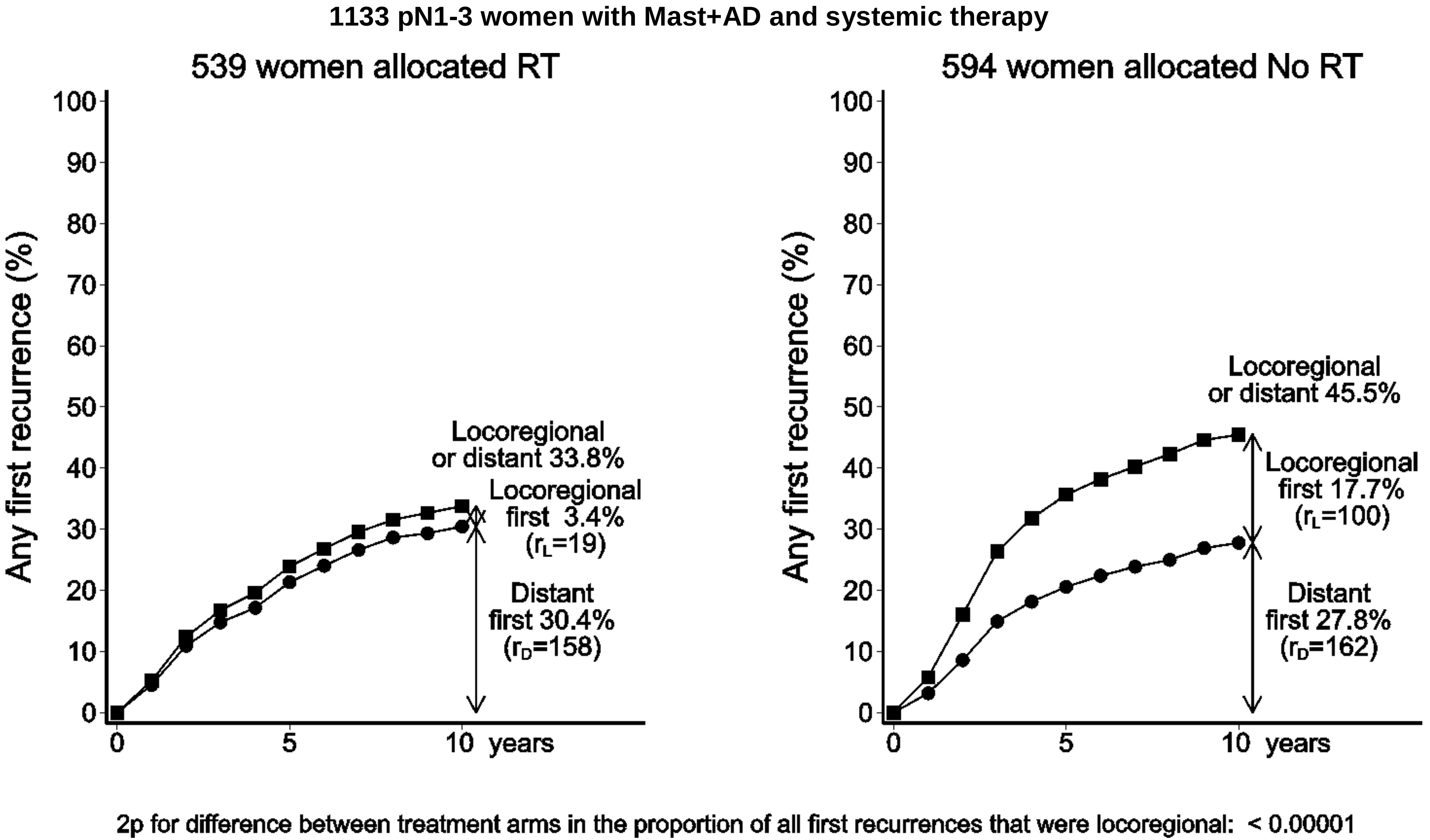

Webfigure 12. Effect of radiotherapy (RT) to the chest wall and regional lymph nodes versus not after mastectomy and axillary dissection (Mast+AD): 10-year risk of locoregional recurrence and recurrence of any type and 15-year risk of breast cancer mortality in 1133 women with 1-3 pathologically positive nodes (pN1-3) in trials where systemic therapy was given to both randomised treatment groups subdivided according to number of positive nodes. See webfigure 1 for methodological note and also webfigures 13-15.

Locoregional recurrence first | **Any first recurrence** | **Breast cancer mortality**

318 women with Mast+AD, systemic therapy and 1 positive node

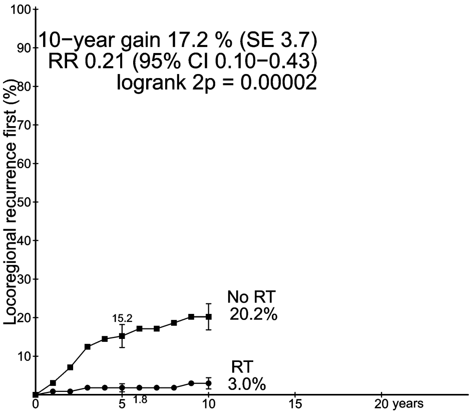

Locoregional recurrence first rates (%/year) and logrank analyses

	Years 0−4	Years 5−9
RT	0.31 (2/635)	0.20 (1/491)
No RT	3.36 (23/685)	1.18 (6/510)
Rate ratio, from	0.21 SE 0.21	0.23 SE 0.40
(O−E)/V	−9.2/5.8	−2.5/1.7

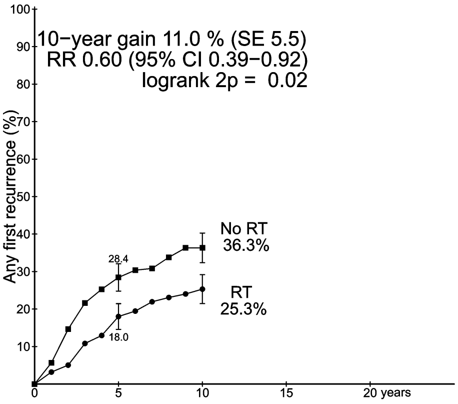

Any first recurrence rates (%/year) and logrank analyses

	Years 0−4	Years 5−9
RT	4.04 (26/643)	1.82 (9/496)
No RT	6.91 (49/709)	2.64 (14/530)
Rate ratio, from	0.56 SE 0.19	0.75 SE 0.39
(O−E)/V	−9.2/16.1	−1.4/5.0

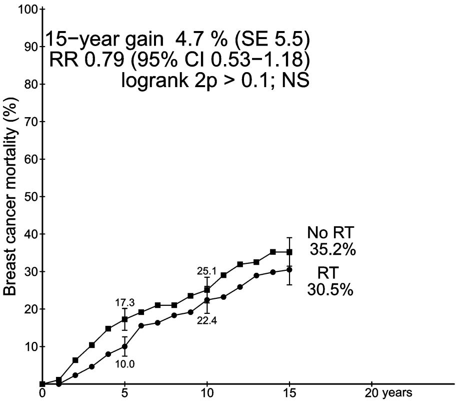

Breast cancer mortality rates (%/year) and logrank analyses

	Years 0−4	Years 5−9	Years 10−14	Years 15+
RT	2.06 (14/678)	2.91 (16/550)	2.16 (10/463)	1.46 (6/410)
No RT	3.67 (29/790)	2.14 (14/655)	3.03 (16/528)	1.64 (7/427)
Rate ratio, from	0.56 SE 0.25	1.55 SE 0.51	0.72 SE 0.35	0.67 SE 0.53
(O−E)/V	−5.5/9.4	2.7/6.1	−1.9/5.9	−1.0/2.4

365 women with Mast+AD, systemic therapy and 2-3 positive nodes

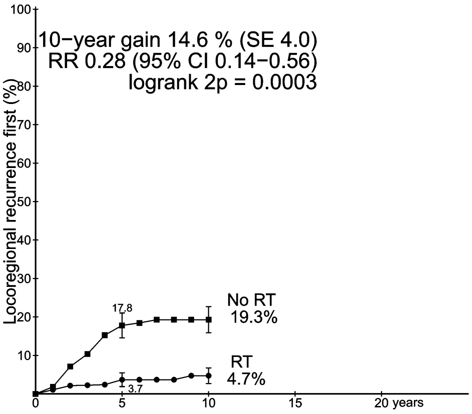

Locoregional recurrence first rates (%/year) and logrank analyses

	Years 0−4	Years 5−9
RT	1.00 (7/703)	0.19 (1/514)
No RT	3.76 (26/692)	0.46 (2/436)
Rate ratio, from	0.25 SE 0.21	0.64 SE 0.96
(O−E)/V	−9.6/7.1	−0.3/0.7

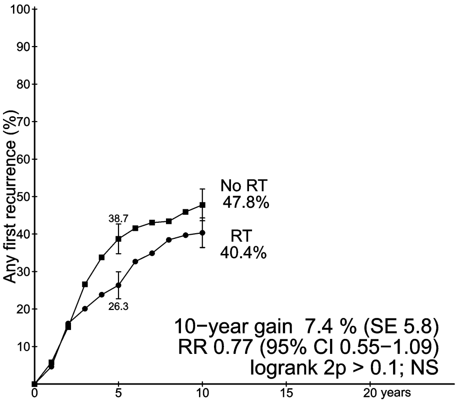

Any first recurrence rates (%/year) and logrank analyses

	Years 0−4	Years 5−9
RT	6.51 (47/722)	4.21 (22/522)
No RT	10.12 (73/721)	4.21 (19/451)
Rate ratio, from	0.64 SE 0.16	1.37 SE 0.41
(O−E)/V	−11.1/24.5	2.6/8.2

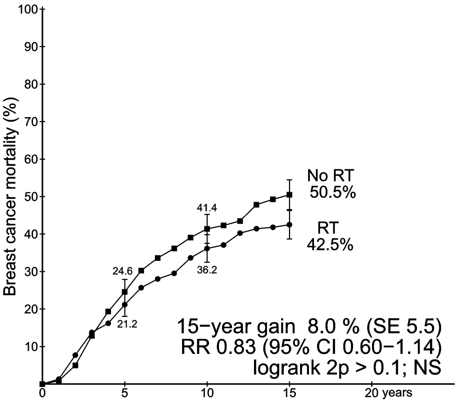

Breast cancer mortality rates (%/year) and logrank analyses

	Years 0−4	Years 5−9	Years 10−14	Years 15+
RT	4.55 (36/791)	4.12 (25/607)	2.07 (10/483)	1.24 (5/402)
No RT	5.56 (46/828)	5.29 (30/567)	3.47 (15/432)	1.48 (5/339)
Rate ratio, from	0.87 SE 0.22	0.81 SE 0.26	0.64 SE 0.35	1.18 SE 0.70
(O−E)/V	−2.5/17.4	−2.5/11.8	−2.4/5.5	0.4/2.4

450 pN1-3 women with exact number of positive nodes unknown, Mast+AD and systemic therapy

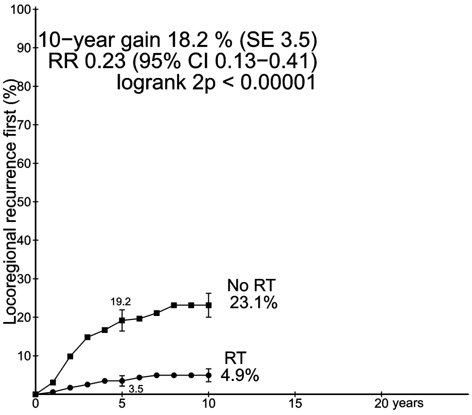

Locoregional recurrence first rates (%/year) and logrank analyses

	Years 0−4	Years 5−9
RT	0.69 (6/874)	0.34 (2/594)
No RT	4.17 (37/888)	1.08 (6/556)
Rate ratio, from	0.21 SE 0.16	0.36 SE 0.46
(O−E)/V	−15.1/9.8	−1.9/1.9

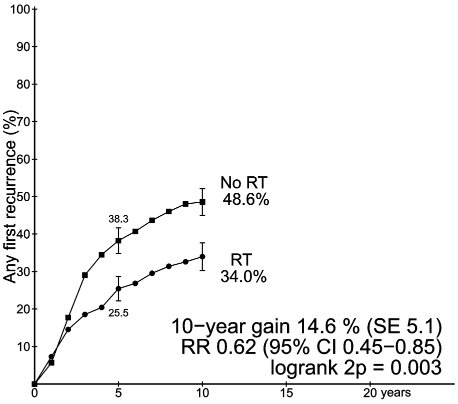

Any first recurrence rates (%/year) and logrank analyses

	Years 0−4	Years 5−9
RT	6.41 (56/874)	2.82 (17/603)
No RT	9.57 (85/888)	3.95 (22/558)
Rate ratio, from	0.61 SE 0.14	0.66 SE 0.28
(O−E)/V	−14.8/30.0	−3.5/8.3

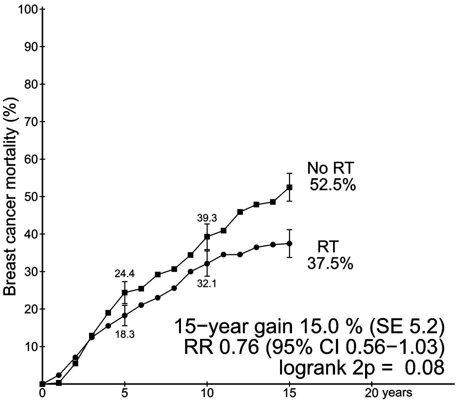

Breast cancer mortality rates (%/year) and logrank analyses

	Years 0−4	Years 5−9	Years 10−14	Years 15+
RT	4.00 (38/950)	3.63 (26/716)	1.95 (9/460)	1.24 (7/565)
No RT	5.37 (56/1043)	4.21 (31/736)	4.71 (21/446)	0.62 (3/480)
Rate ratio, from	0.74 SE 0.19	0.87 SE 0.26	0.40 SE 0.26	2.57 SE 1.08
(O−E)/V	−5.9/20.0	−1.8/12.4	−6.0/6.6	2.2/2.4

Webfigure 13. Effect of radiotherapy (RT) to the chest wall and regional lymph nodes versus not after mastectomy and axillary dissection (Mast+AD): 10-year risk of recurrence and type of first recurrence, by allocated treatment, in 318 women with 1 pathologically positive node (pN1) and where systemic therapy was given to both randomised treatment groups. (r_L = number of women for whom first recurrence was locoregional, r_D = number women for whom distant recurrence was first.)

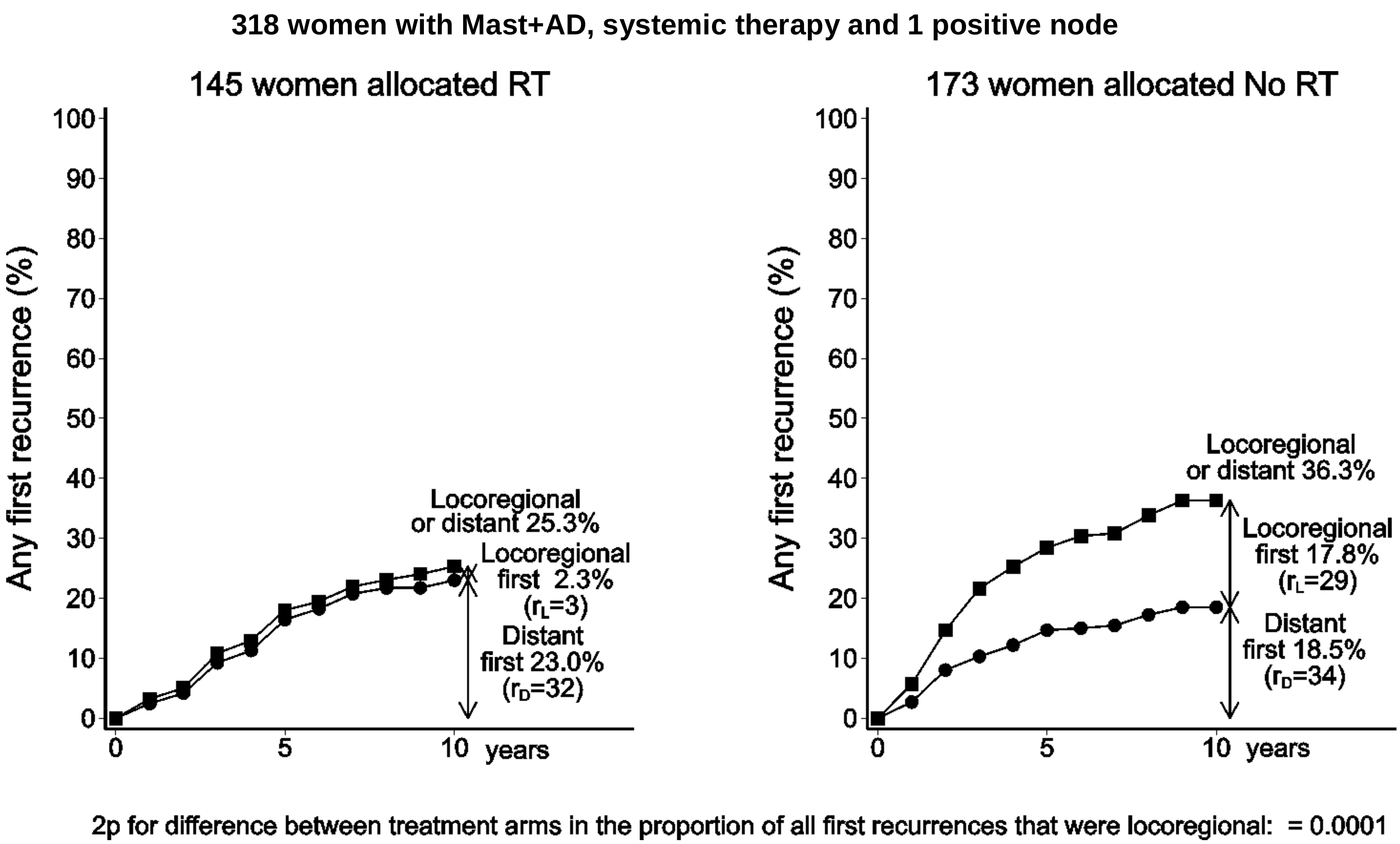

Webfigure 14. Effect of radiotherapy (RT) to the chest wall and regional lymph nodes versus not after mastectomy and axillary dissection (Mast+AD): 10-year risk of recurrence and type of first recurrence, by allocated treatment, in 365 women with 2-3 pathologically positive nodes (pN2-3) and where systemic therapy was given to both randomised treatment groups. (r_L = number of women for whom first recurrence was locoregional, r_D = number women for whom distant recurrence was first.)

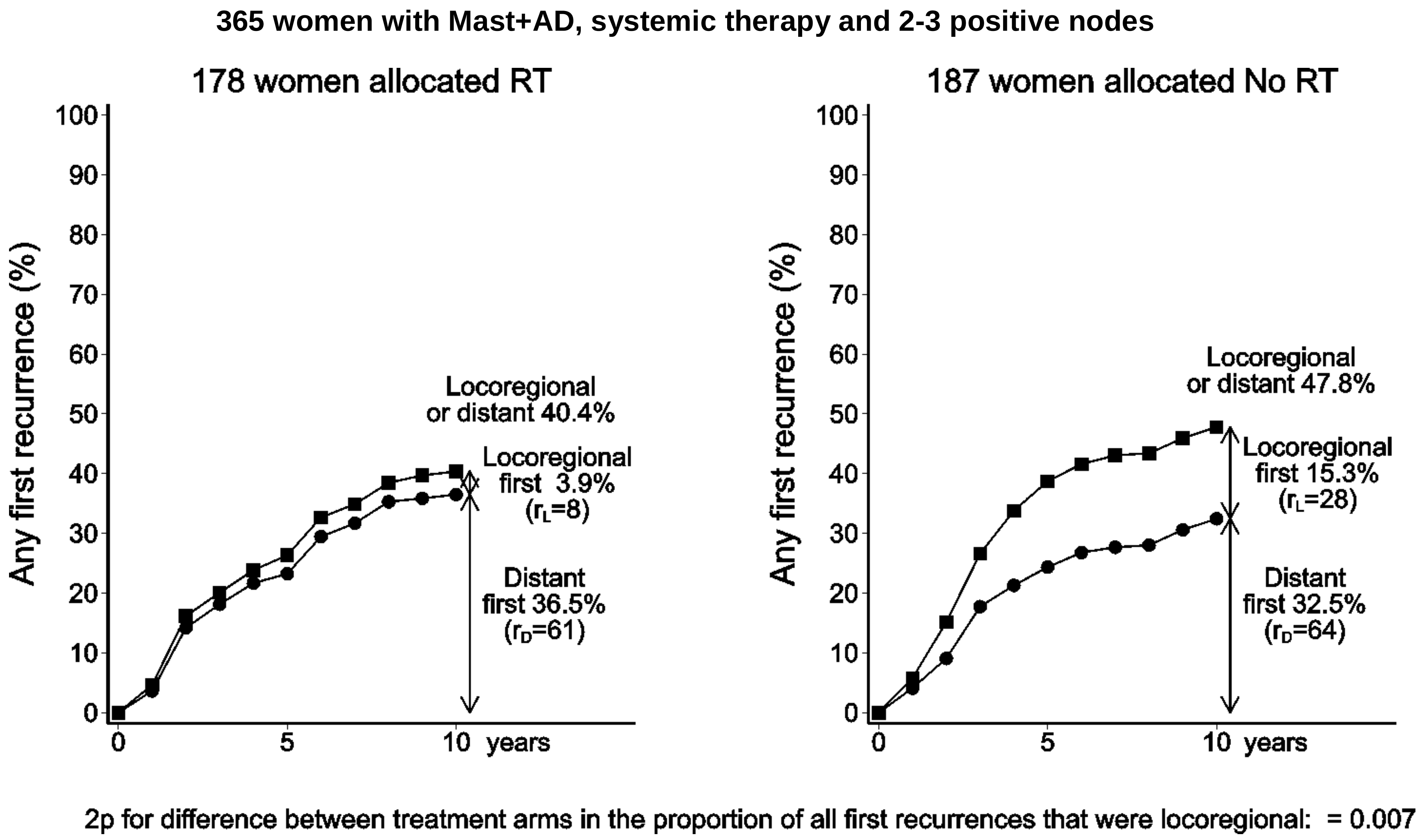

Webfigure 15. Effect of radiotherapy (RT) to the chest wall and regional lymph nodes versus not after mastectomy and axillary dissection (Mast+AD): 10-year risk of recurrence and type of first recurrence, by allocated treatment, in 450 women with 1-3 pathologically positive nodes (pN1-3) but the exact number of positive nodes unknown and where systemic therapy was given to both randomised treatment groups. (r_L = number of women for whom first recurrence was locoregional, r_D = number women for whom distant recurrence was first.)

450 pN1-3 women but exact number of positive nodes unknown, Mast+AD and systemic therapy

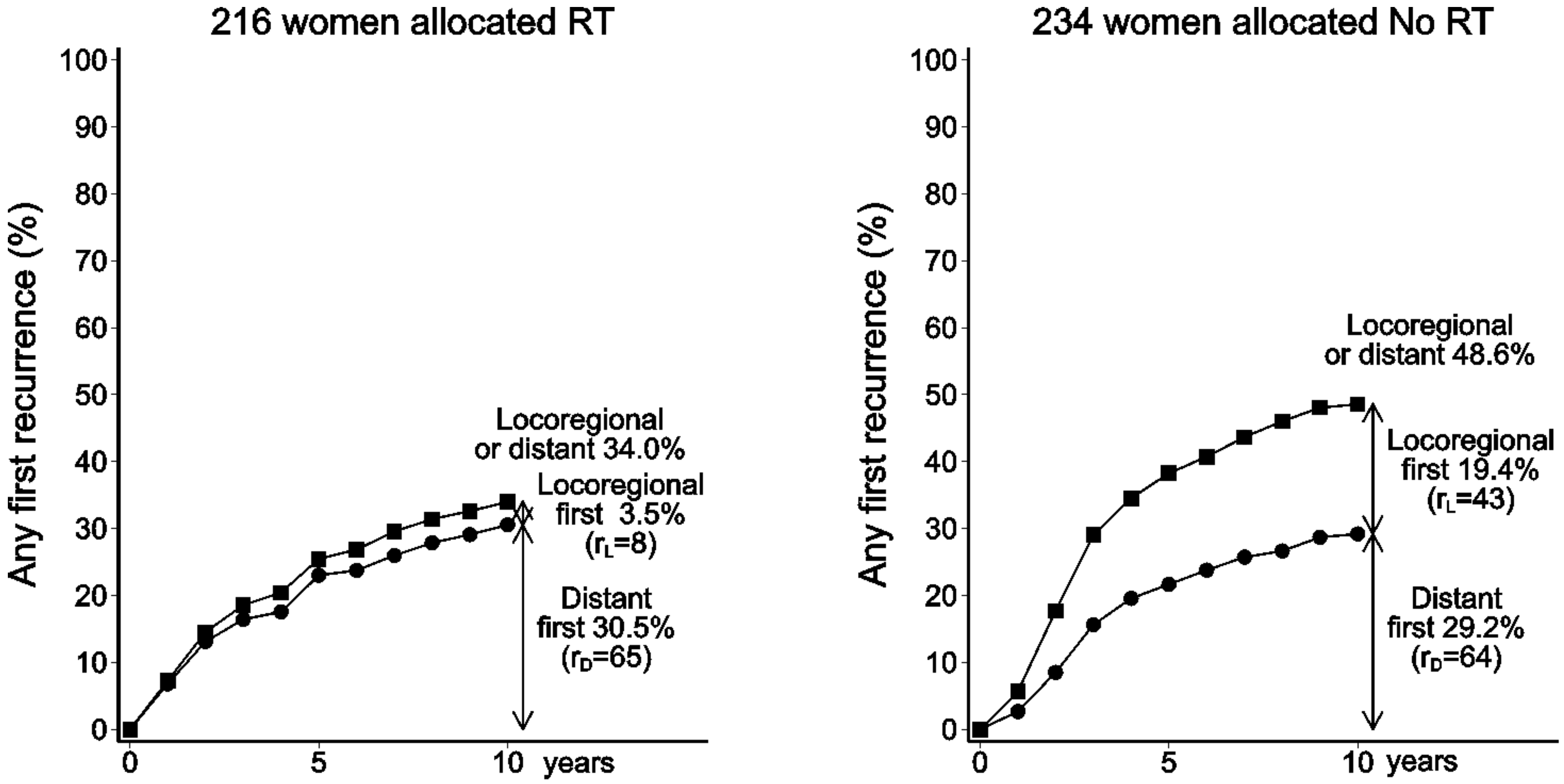

2p for difference between treatment arms in the proportion of all first recurrences that were locoregional: = 0.00002

Webfigure 16. Effect of radiotherapy (RT) to the chest wall and regional lymph nodes versus not after mastectomy and axillary dissection (Mast+AD): 10-year risk of locoregional recurrence and recurrence of any type and 20-year risk of breast cancer and all-cause mortality in 1772 women with 4+ pathologically positive nodes (pN4+). See webfigure 1 for methodological note and also webfigure 17.

1772 pN4+ women with Mast+AD

Locoregional recurrence first

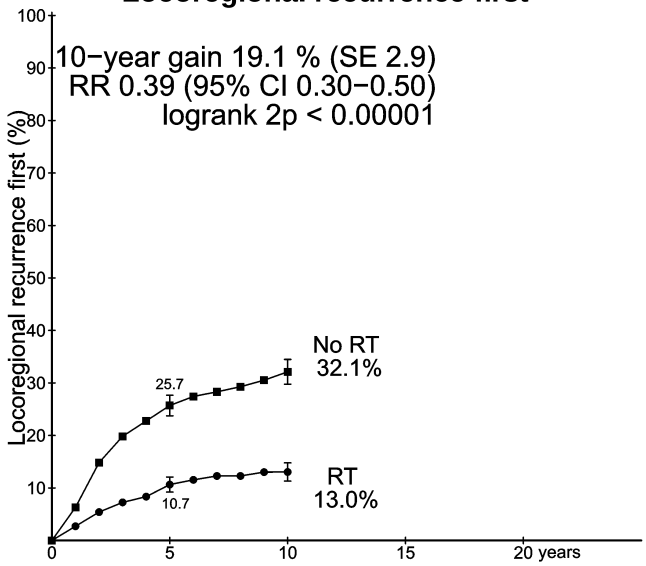

Locoregional recurrence first rates (%/year) and logrank analyses

	Years 0−4	Years 5−9
RT	2.59 (69/2663)	0.73 (9/1241)
No RT	6.53 (155/2375)	1.81 (17/937)
Rate ratio, from	0.39 SE 0.09	0.37 SE 0.27
(O−E)/V	−48.3/50.9	−5.5/5.5

Any first recurrence

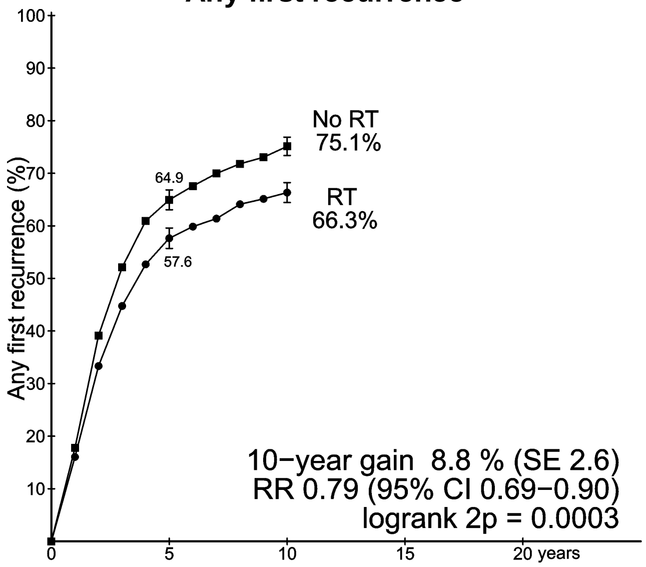

Any first recurrence rates (%/year) and logrank analyses

	Years 0−4	Years 5−9
RT	18.53 (508/2741)	5.21 (67/1286)
No RT	22.07 (549/2487)	7.43 (75/1010)
Rate ratio, from	0.81 SE 0.06	0.67 SE 0.15
(O−E)/V	−42.8/197.8	−11.3/28.6

Breast cancer mortality

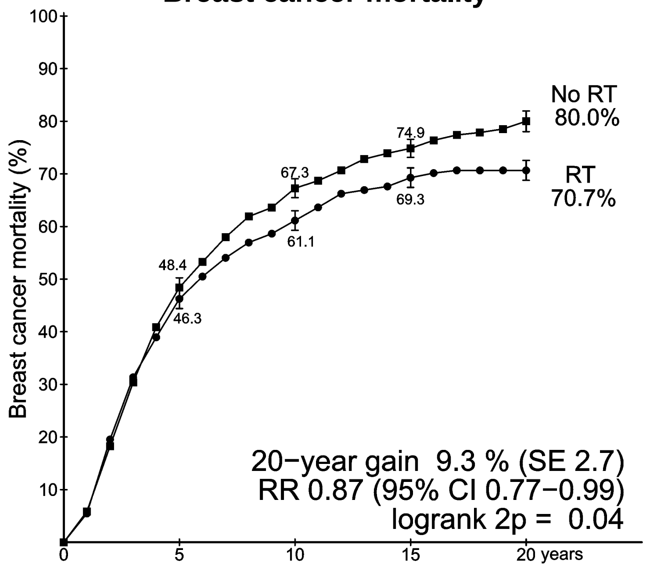

Breast cancer mortality rates (%/year) and logrank analyses

	Years 0−4	Years 5−9	Years 10−14	Years 15−19	Years 20+
RT	12.20 (400/3278)	6.85 (115/1678)	5.05 (47/931)	0.89 (4/450)	0.60 (1/166)
No RT	12.54 (406/3237)	9.14 (137/1499)	5.20 (40/770)	5.83 (20/343)	2.04 (2/98)
Rate ratio, from	0.95 SE 0.08	0.71 SE 0.12	0.99 SE 0.24	0.35 SE 0.27	0.24 SE 0.71
(O−E)/V	−7.9/163.1	−17.3/51.1	−0.1/17.1	−5.5/5.3	−0.8/0.6

Any death

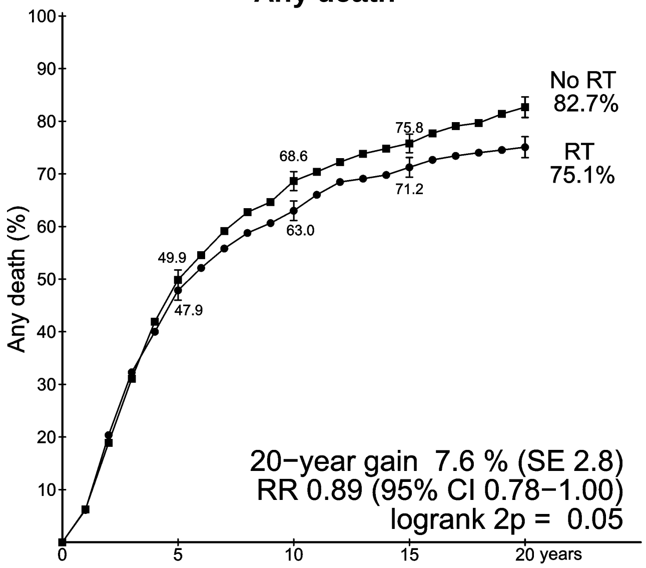

Any death rates (%/year) and logrank analyses

	Years 0−4	Years 5−9	Years 10−14	Years 15−19	Years 20+
RT	12.91 (423/3278)	7.69 (129/1678)	6.23 (58/931)	3.56 (16/450)	3.01 (5/166)
No RT	13.25 (429/3237)	9.87 (148/1499)	6.11 (47/770)	7.87 (27/343)	4.08 (4/98)
Rate ratio, from	0.96 SE 0.07	0.74 SE 0.11	1.01 SE 0.22	0.48 SE 0.24	0.54 SE 0.58
(O−E)/V	−7.3/173.3	−17.1/56.6	0.2/20.3	−6.3/8.7	−1.0/1.7

Webfigure 17. Effect of radiotherapy (RT) to the chest wall and regional lymph nodes versus not after mastectomy and axillary dissection (Mast+AD): 10-year risk of recurrence and type of first recurrence, by allocated treatment, in 1772 women with 4+ pathologically positive nodes (pN4+). (r_L = number of women for whom first recurrence was locoregional, r_D = number women for whom distant recurrence was first.)

1772 pN4+ women with Mast+AD

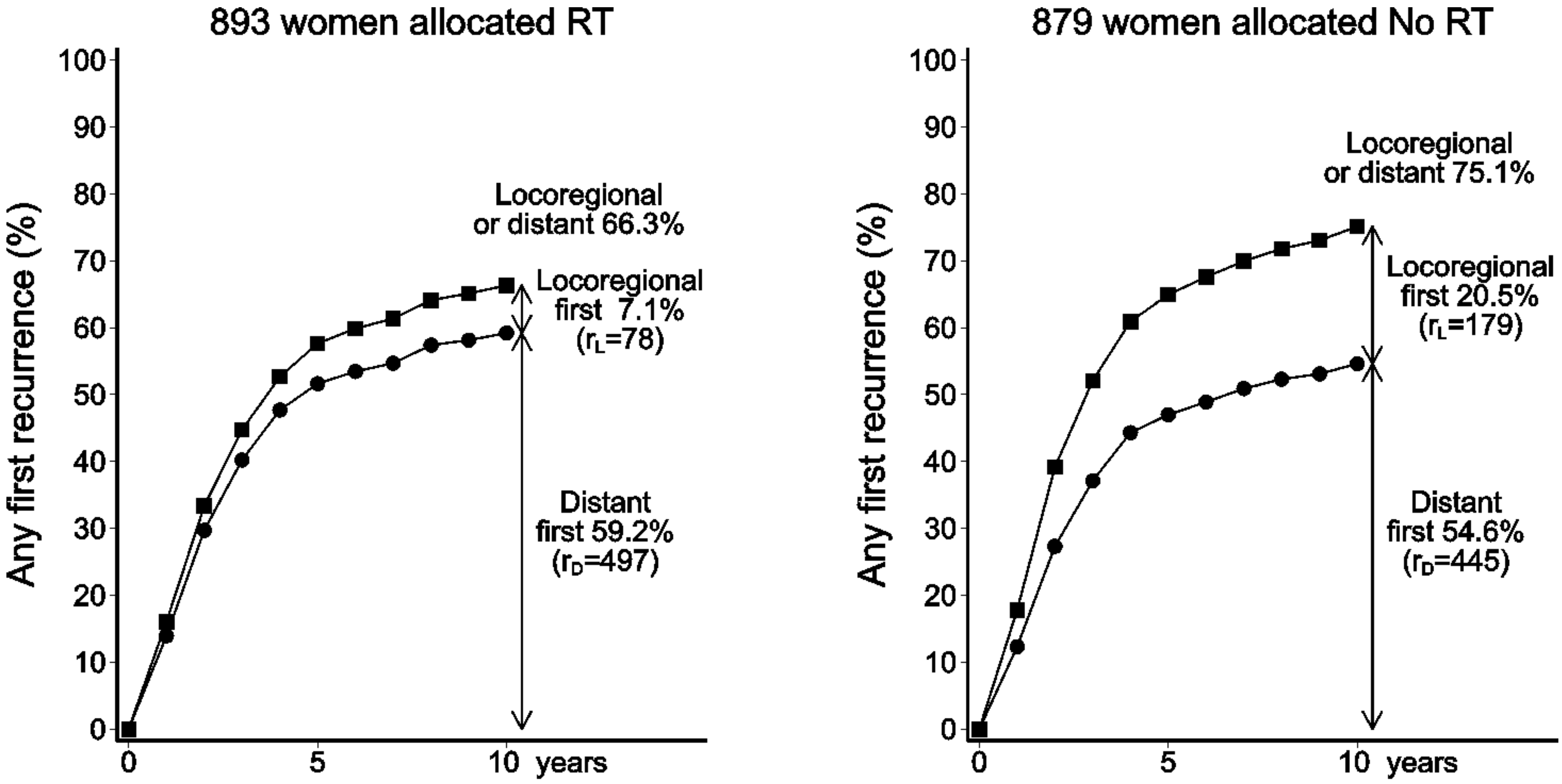

2p for difference between treatment arms in the proportion of all first recurrences that were locoregional: < 0.00001

Webfigure 18. Effect of radiotherapy (RT) to the chest wall and regional lymph nodes versus not after mastectomy and axillary dissection (Mast+AD): Event rate ratios and 95% confidence intervals for locoregional recurrence and recurrence of any type during years 0-9 and for breast cancer mortality in 1772 women with 4+ pathologically positive nodes (pN4+) by prognostic and other factors.
Categories with unknowns are excluded from the heterogeneity and trend tests.

1772 pN4+ women with Mast+AD

Locoregional recurrence first (years 0-9)

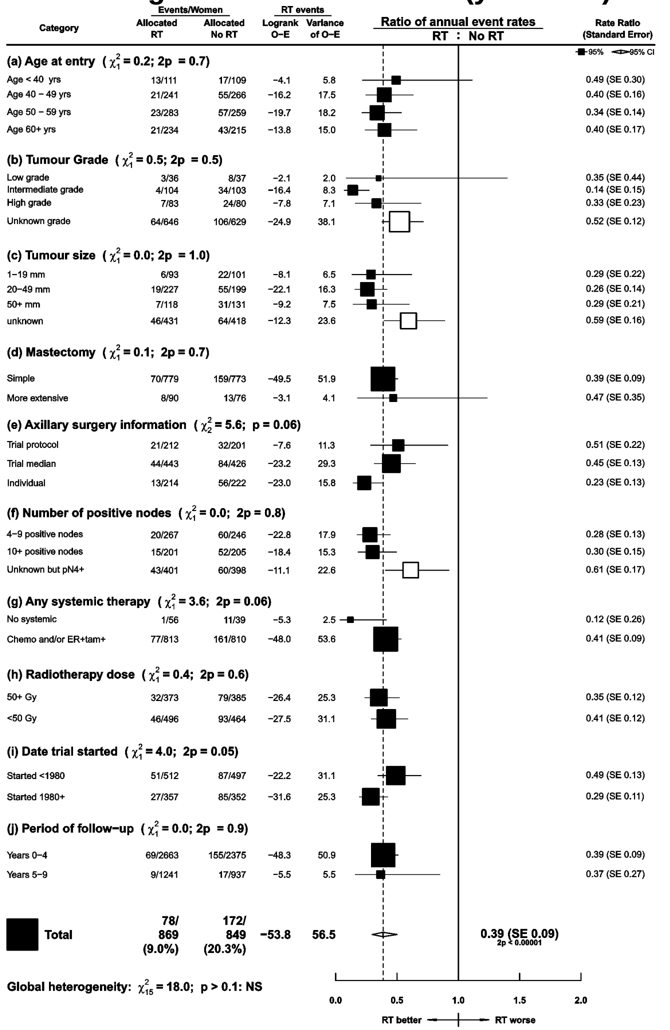

Any first recurrence (years 0-9)

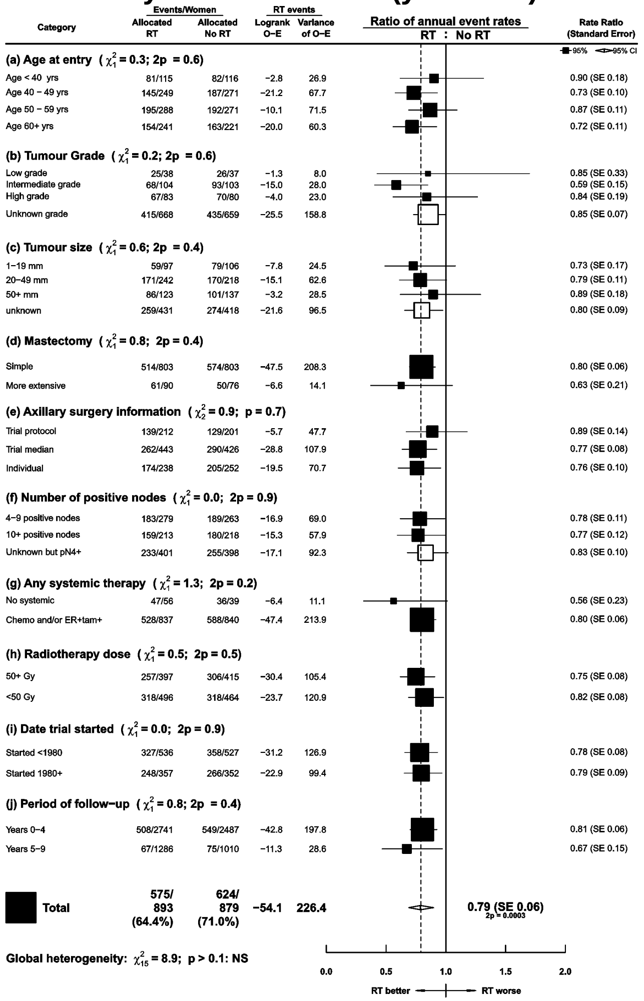

Breast cancer mortality

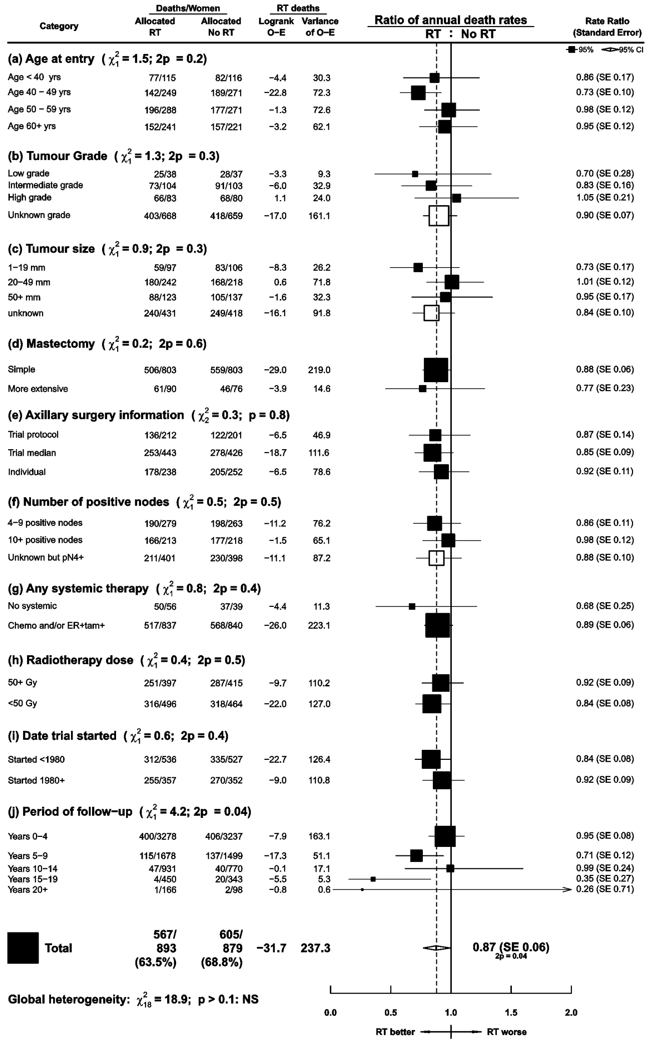

Note:In (g), 135 women who were ER positive with tamoxifen also had chemotherapy. In (h), trials that used orthovoltage irradiation are included in the <50 Gy category.

Webfigure 19. Effect of radiotherapy (RT) to the chest wall and regional lymph nodes versus not after mastectomy and axillary dissection (Mast+AD): 10-year risk of locoregional recurrence and recurrence of any type and 20-year risk of breast cancer and all-cause mortality in 1677 women with 4+ pathologically positive nodes (pN4+) in trials where systemic therapy was given to both randomised treatment groups. See webfigure 1 for methodological note and also webfigure 20.

1677 pN4+ women with Mast+AD and systemic therapy

Locoregional recurrence first

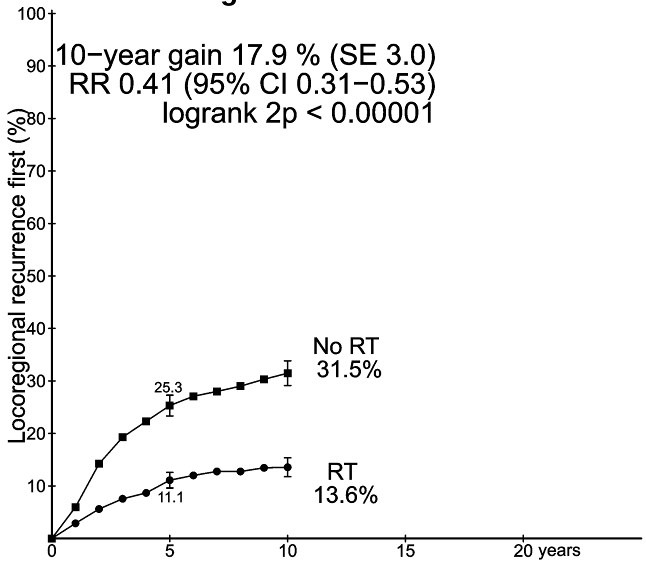

Locoregional recurrence first rates (%/year) and logrank analyses

	Years 0−4	Years 5−9
RT	2.70 (68/2522)	0.75 (9/1199)
No RT	6.30 (145/2301)	1.73 (16/927)
Rate ratio, from	0.41 SE 0.10	0.39 SE 0.28
(O−E)/V	−43.0/48.3	−5.0/5.3

Any first recurrence

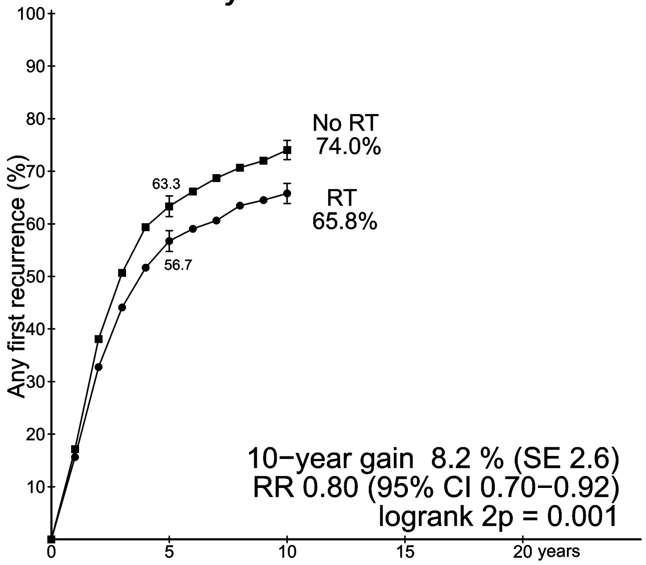

Any first recurrence rates (%/year) and logrank analyses

	Years 0−4	Years 5−9
RT	17.88 (465/2600)	5.06 (63/1244)
No RT	21.30 (514/2413)	7.41 (74/999)
Rate ratio, from	0.82 SE 0.07	0.68 SE 0.16
(O−E)/V	−36.6/185.7	−10.8/28.1

Breast cancer mortality

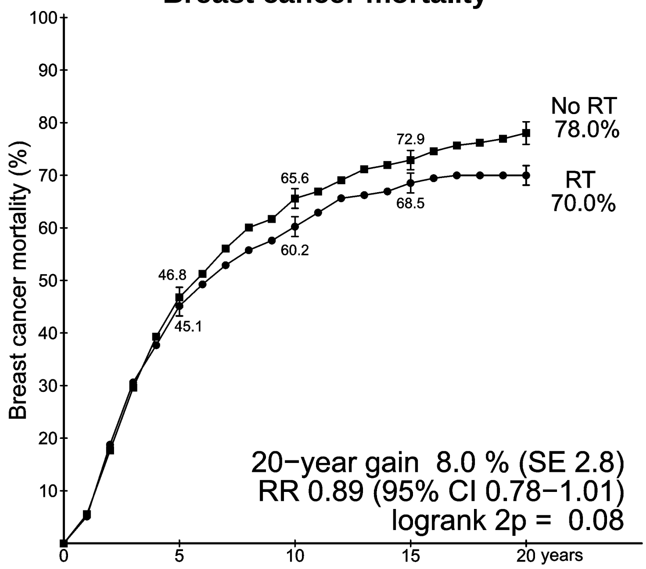

Breast cancer mortality rates (%/year) and logrank analyses

	Years 0−4	Years 5−9	Years 10−14	Years 15−19	Years 20+
RT	11.69 (363/3104)	6.59 (107/1624)	4.82 (43/893)	0.70 (3/431)	0.61 (1/164)
No RT	12.09 (377/3119)	8.85 (131/1481)	4.96 (38/765)	5.83 (20/343)	2.04 (2/98)
Rate ratio, from	0.96 SE 0.08	0.74 SE 0.12	1.06 SE 0.25	0.35 SE 0.27	0.24 SE 0.71
(O−E)/V	−5.9/151.2	−14.8/49.4	1.0/16.6	−5.5/5.3	−0.8/0.6

Any death

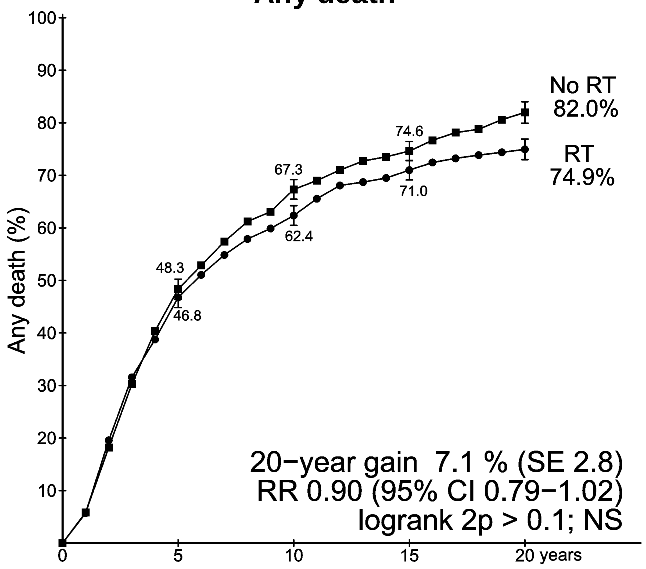

Any death rates (%/year) and logrank analyses

	Years 0−4	Years 5−9	Years 10−14	Years 15−19	Years 20+
RT	12.37 (384/3104)	7.45 (121/1624)	6.05 (54/893)	3.25 (14/431)	2.43 (4/164)
No RT	12.79 (399/3119)	9.52 (141/1481)	5.88 (45/765)	7.87 (27/343)	4.08 (4/98)
Rate ratio, from	0.97 SE 0.08	0.77 SE 0.12	1.07 SE 0.23	0.48 SE 0.24	0.54 SE 0.58
(O−E)/V	−5.3/160.7	−14.3/54.5	1.4/19.8	−6.3/8.7	−1.0/1.7

Webfigure 20. Effect of radiotherapy (RT) to the chest wall and regional lymph nodes versus not after mastectomy and axillary dissection (Mast+AD): 10-year risk of recurrence and type of first recurrence, by allocated treatment, in 1677 women with 4+ pathologically positive nodes (pN4+) in trials where systemic therapy was given to both randomised treatment groups. (r_L = number of women for whom first recurrence was locoregional, r_D = number women for whom distant recurrence was first.)

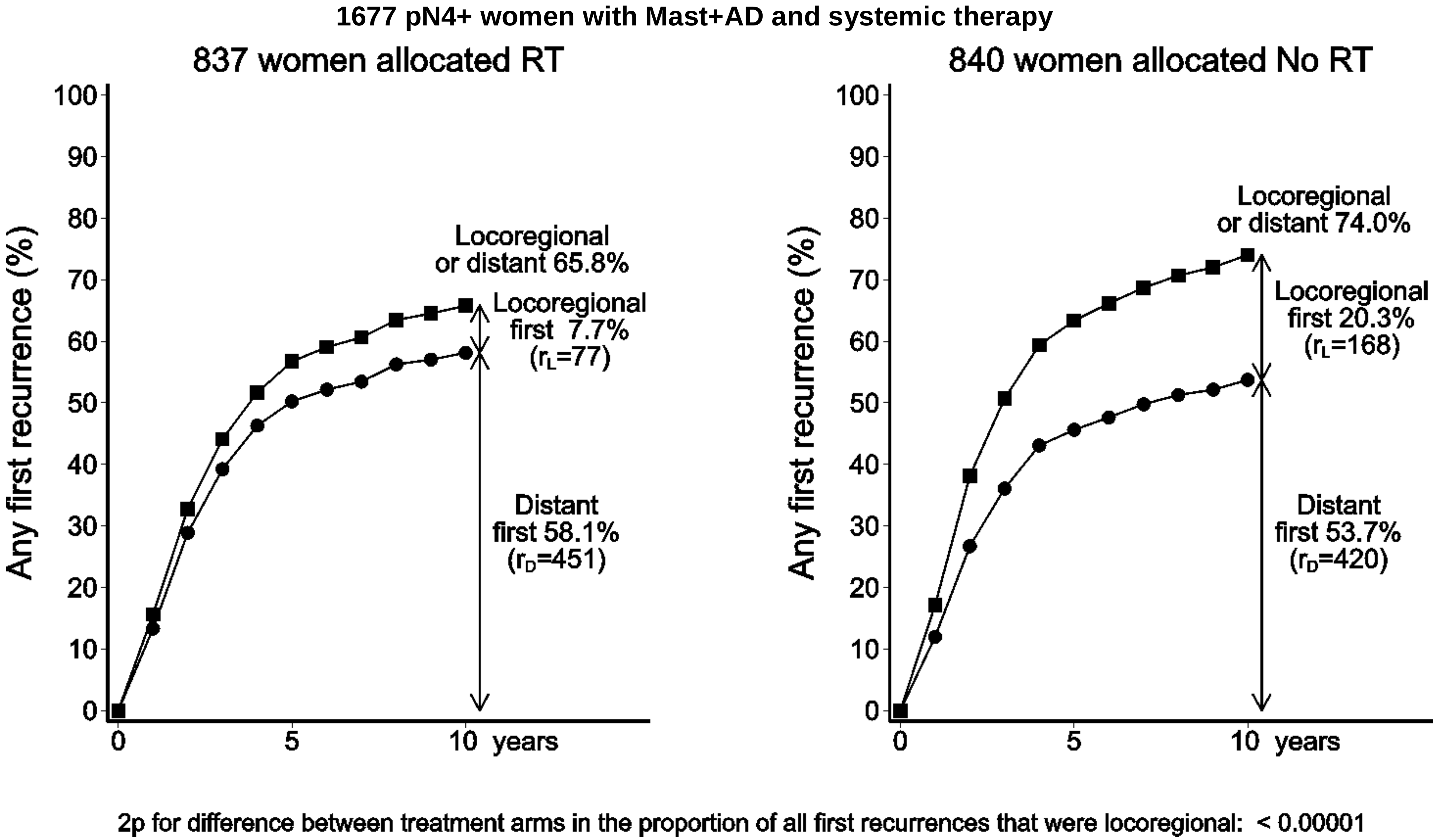

Webfigure 21. Effect of radiotherapy (RT) to the chest wall and regional lymph nodes versus not after mastectomy and axillary dissection (Mast+AD): 10-year risk of locoregional recurrence and recurrence of any type and 15-year risk of breast cancer mortality in 1677 women with 4+ pathologically positive nodes (pN4+) in trials where systemic therapy was given to both randomised treatment groups subdivided according to number of positive nodes. See webfigure 1 for methodological note and also webfigures 22-24.

Locoregional recurrence first | **Any first recurrence** | **Breast cancer mortality**

479 women with Mast+AD, systemic therapy and 4-9 positive nodes

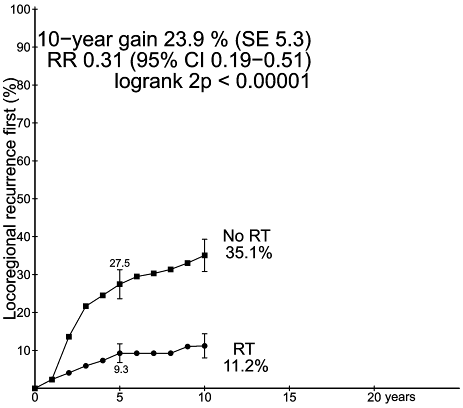

Locoregional recurrence first rates (%/year) and logrank analyses

	Years 0−4	Years 5−9
RT	2.16 (17/787)	0.68 (3/438)
No RT	6.67 (46/689)	1.88 (6/319)
Rate ratio, from	0.33 SE 0.16	0.18 SE 0.35
(O−E)/V	−15.5/14.2	−3.2/1.8

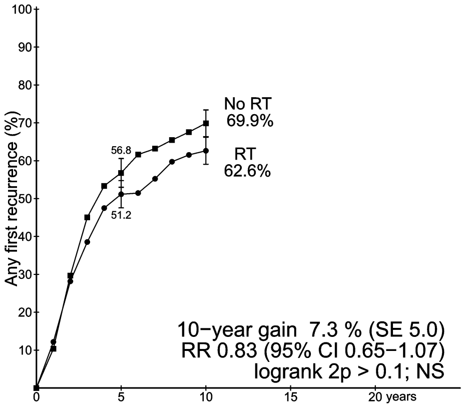

Any first recurrence rates (%/year) and logrank analyses

	Years 0−4	Years 5−9
RT	15.14 (126/832)	6.06 (28/462)
No RT	17.67 (134/758)	7.95 (29/365)
Rate ratio, from	0.86 SE 0.13	0.71 SE 0.26
(O−E)/V	−7.5/50.0	−3.8/10.9

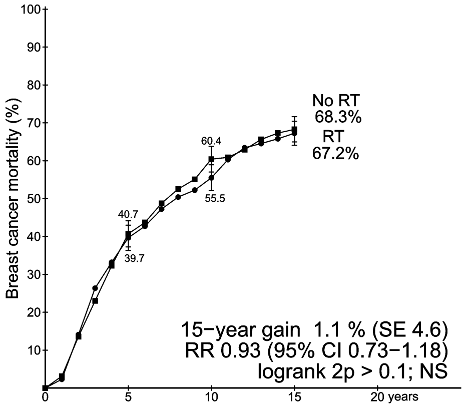

Breast cancer mortality rates (%/year) and logrank analyses

	Years 0−4	Years 5−9	Years 10−14	Years 15+
RT	9.78 (96/982)	6.31 (38/602)	5.54 (22/397)	0.75 (2/268)
No RT	10.01 (95/949)	7.72 (43/557)	5.15 (19/369)	6.02 (14/233)
Rate ratio, from	0.99 SE 0.16	0.78 SE 0.22	1.52 SE 0.43	0.23 SE 0.32
(O−E)/V	−0.5/39.9	−4.2/16.5	3.6/8.5	−4.0/2.7

403 women with Mast+AD, systemic therapy and 10+ positive nodes

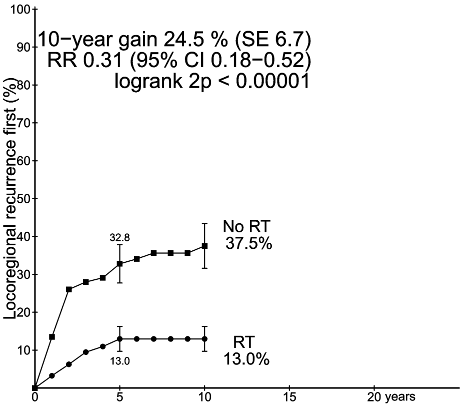

Locoregional recurrence first rates (%/year) and logrank analyses

	Years 0−4	Years 5−9
RT	2.83 (14/494)	0.00 (0/240)
No RT	10.55 (46/436)	1.93 (3/155)
Rate ratio, from	0.32 SE 0.16	0.16 SE 0.54
(O−E)/V	−15.4/13.6	−1.3/0.7

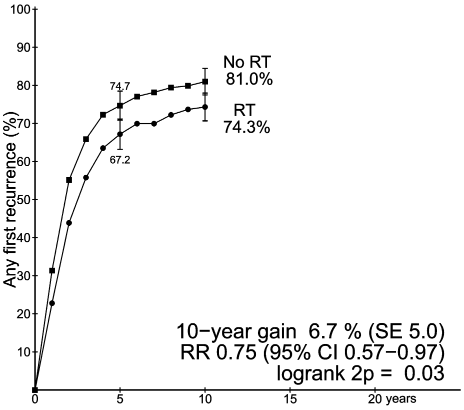

Any first recurrence rates (%/year) and logrank analyses

	Years 0−4	Years 5−9
RT	24.49 (129/527)	5.19 (13/250)
No RT	33.00 (158/479)	7.32 (13/178)
Rate ratio, from	0.74 SE 0.12	0.80 SE 0.40
(O−E)/V	−14.8/49.5	−1.1/4.9

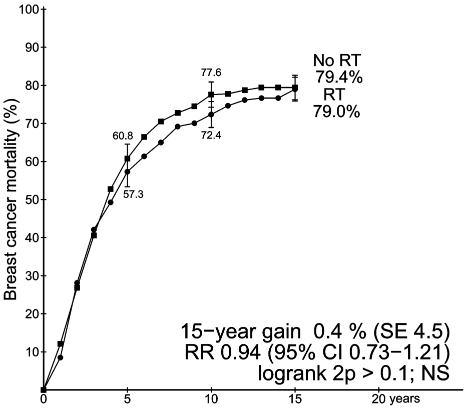

Breast cancer mortality rates (%/year) and logrank analyses

	Years 0−4	Years 5−9	Years 10−14	Years 15+
RT	16.59 (109/657)	8.28 (27/326)	5.22 (11/211)	1.68 (2/119)
No RT	18.01 (127/705)	12.41 (34/274)	1.86 (3/162)	5.19 (4/77)
Rate ratio, from	0.91 SE 0.14	0.77 SE 0.25	2.66 SE 0.99	1.85 SE 1.41
(O−E)/V	−4.0/44.9	−3.1/11.9	2.9/2.9	0.6/1.0

795 pN4+ women with exact number of positive nodes unknown, Mast+AD and systemic therapy

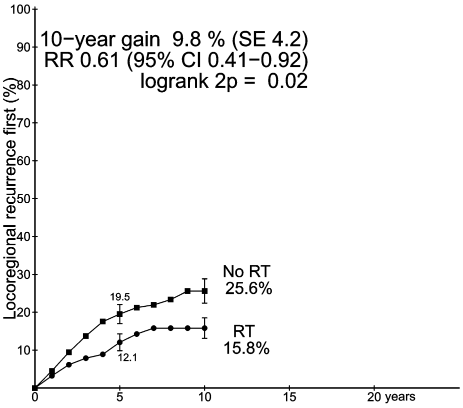

Locoregional recurrence first rates (%/year) and logrank analyses

	Years 0−4	Years 5−9
RT	2.98 (37/1242)	1.15 (6/521)
No RT	4.51 (53/1176)	1.55 (7/452)
Rate ratio, from	0.59 SE 0.17	0.83 SE 0.57
(O−E)/V	−10.6/20.1	−0.5/2.6

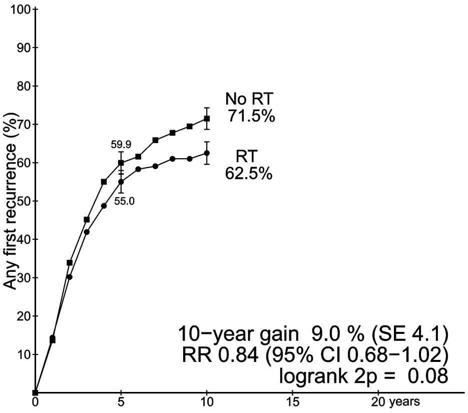

Any first recurrence rates (%/year) and logrank analyses

	Years 0−4	Years 5−9
RT	16.91 (210/1242)	4.14 (22/532)
No RT	18.88 (222/1176)	7.00 (32/457)
Rate ratio, from	0.88 SE 0.10	0.58 SE 0.23
(O−E)/V	−10.7/81.2	−5.9/10.9

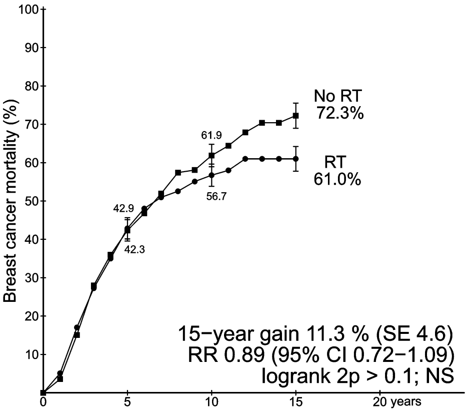

Breast cancer mortality rates (%/year) and logrank analyses

	Years 0−4	Years 5−9	Years 10−14	Years 15+
RT	10.78 (158/1466)	6.04 (42/695)	3.51 (10/285)	0.00 (0/209)
No RT	10.58 (155/1464)	8.31 (54/650)	6.81 (16/235)	3.05 (4/131)
Rate ratio, from	1.02 SE 0.13	0.68 SE 0.19	0.45 SE 0.33	0.47 SE 0.64
(O−E)/V	1.3/62.2	−7.5/19.1	−3.5/4.5	−0.9/1.2

Webfigure 22. Effect of radiotherapy (RT) to the chest wall and regional lymph nodes versus not after mastectomy and axillary dissection (Mast+AD): 10-year risk of recurrence and type of first recurrence, by allocated treatment, in 479 women with 4-9 pathologically positive nodes (pN4-9) in trials where systemic therapy was given to both randomised treatment groups. (r_L = number of women for whom first recurrence was locoregional, r_D = number women for whom distant recurrence was first.)

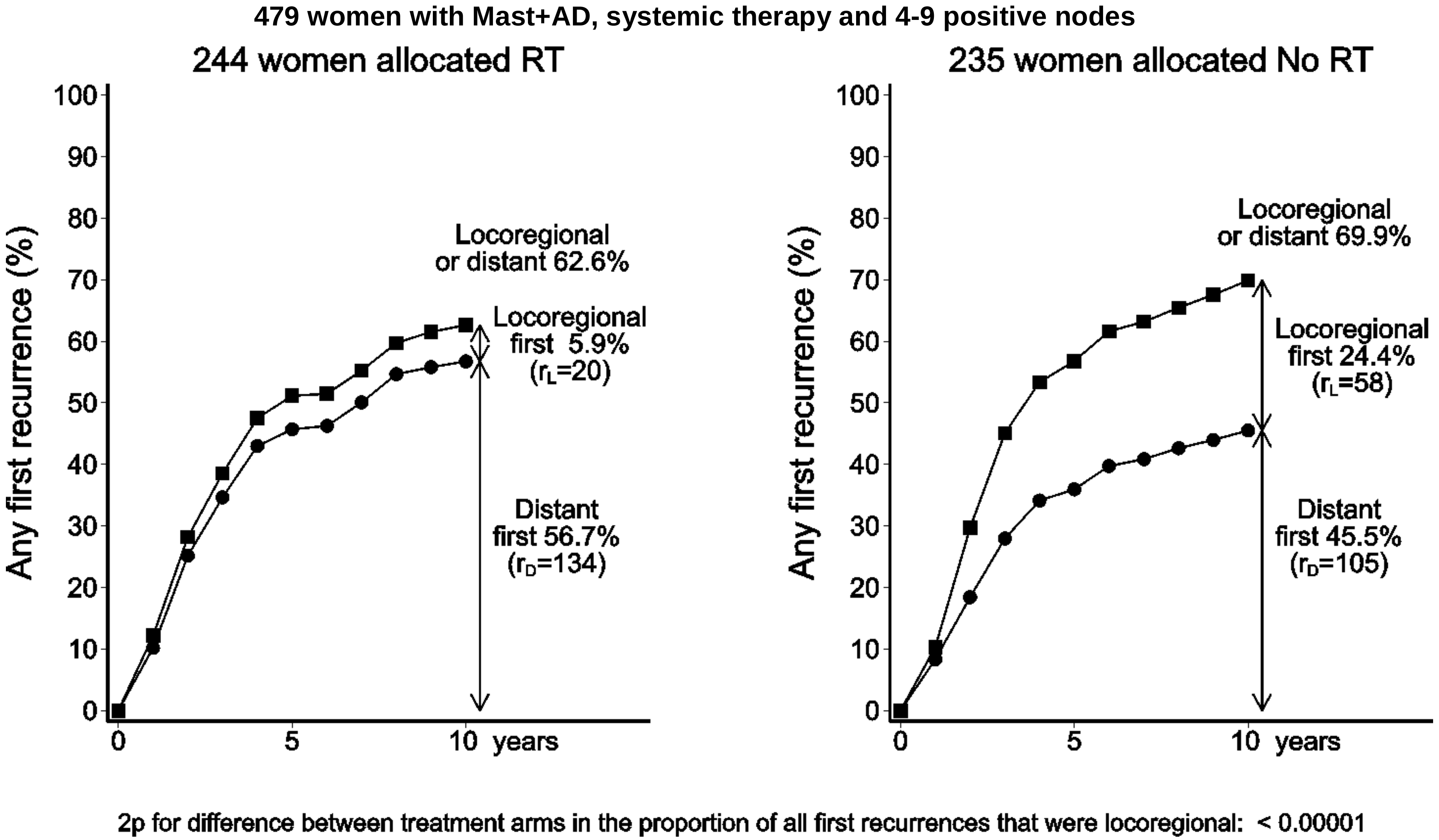

Webfigure 23. Effect of radiotherapy (RT) to the chest wall and regional lymph nodes versus not after mastectomy and axillary dissection (Mast+AD): 10-year risk of recurrence and type of first recurrence, by allocated treatment, in 403 women with 10+ pathologically positive nodes (pN10+) in trials where systemic therapy was given to both randomised treatment groups. (r_L = number of women for whom first recurrence was locoregional, r_D = number women for whom distant recurrence was first.)

403 women with Mast+AD, systemic therapy and 10+ positive nodes

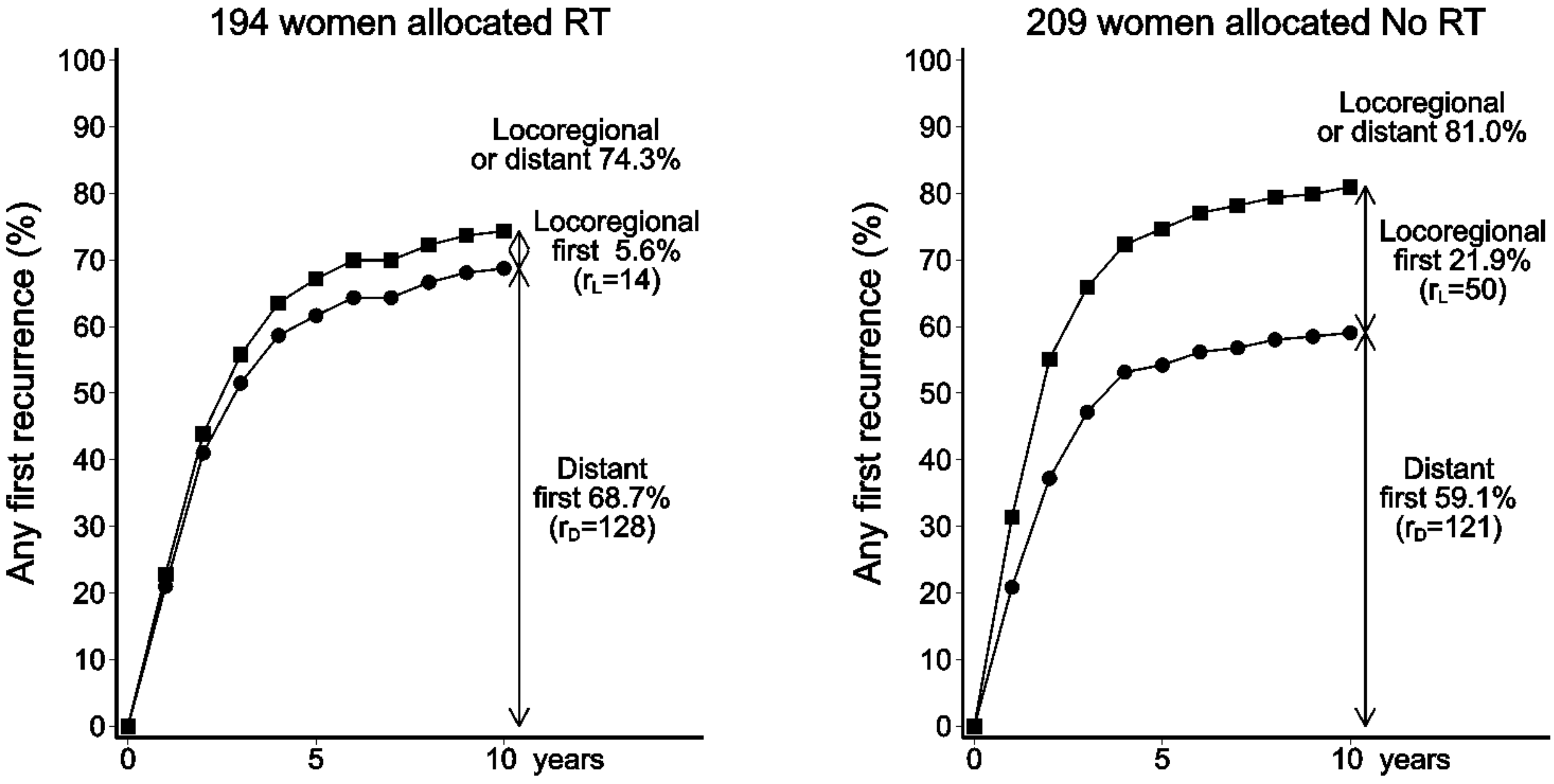

2p for difference between treatment arms in the proportion of all first recurrences that were locoregional: = 0.00002

Webfigure 24. Effect of radiotherapy (RT) to the chest wall and regional lymph nodes versus not after mastectomy and axillary dissection (Mast+AD): 10-year risk of recurrence and type of first recurrence, by allocated treatment, in 795 women with 4+ pathologically positive nodes but the exact number of positive nodes unknown in trials where systemic therapy was given to both randomised treatment groups. (r_L = number of women for whom first recurrence was locoregional, r_D = number women for whom distant recurrence was first.)

795 pN4+ women but exact number of positive nodes unknown, Mast+AD and systemic therapy

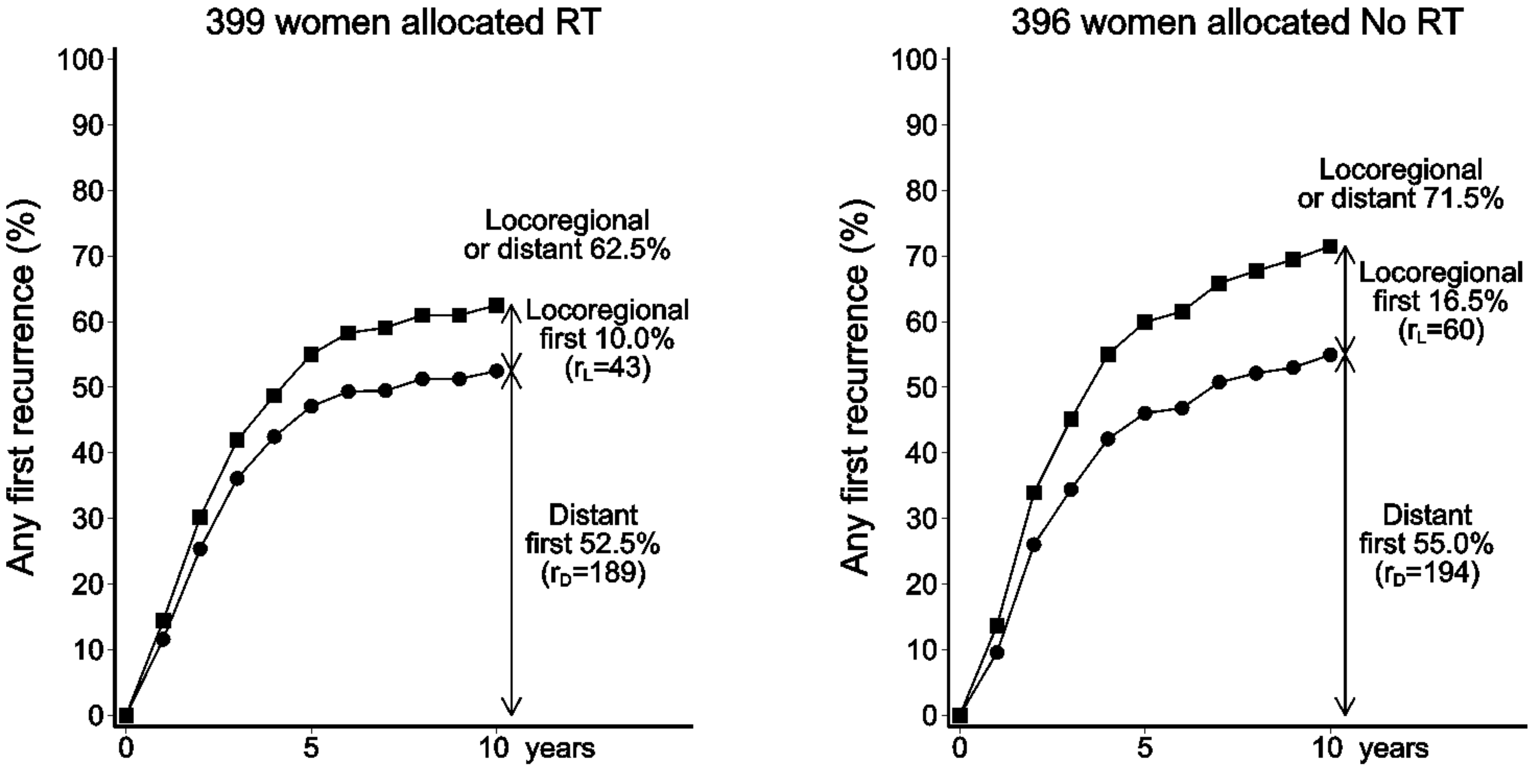

2p for difference between treatment arms in the proportion of all first recurrences that were locoregional: > 0.1; NS

Webfigure 25. Effect of radiotherapy (RT) to the chest wall and regional lymph nodes versus not after mastectomy and axillary sampling (Mast+AS): 10-year risk of locoregional recurrence and recurrence of any type and 20-year risk of breast cancer and all-cause mortality in 870 women with pathologically node-negative (pN0) disease. See webfigure 1 for methodological note and also webfigure 26. Note: 0 locoregional recurrences, 8 recurrences of any type and 10 breast cancer deaths were reported among the 36 pN0 women with tumours ⩾ 5 cm who were allocated to receive radiotherapy. 4 locoregional recurrences, 11 recurrences of any type and 9 breast cancer deaths were reported among the 36 pN0 women with tumours ⩾ 5 cm who were allocated to not to receive radiotherapy.

870 pN0 women with Mast+AS

Locoregional recurrence first

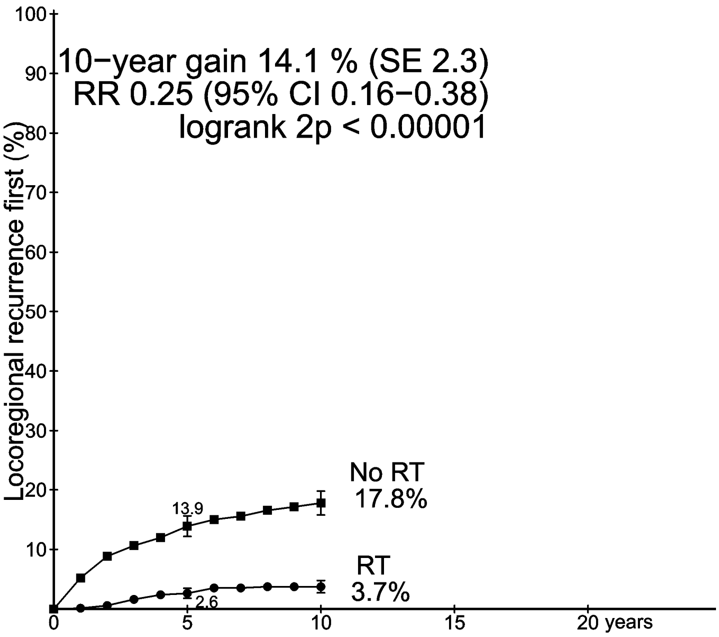

Locoregional recurrence first rates (%/year) and logrank analyses

	Years 0−4	Years 5−9
RT	0.54 (10/1849)	0.27 (4/1504)
No RT	3.11 (58/1862)	1.02 (14/1373)
Rate ratio, from	0.23 SE 0.13	0.32 SE 0.29
(O−E)/V	−23.0/15.8	−4.8/4.2

Any first recurrence

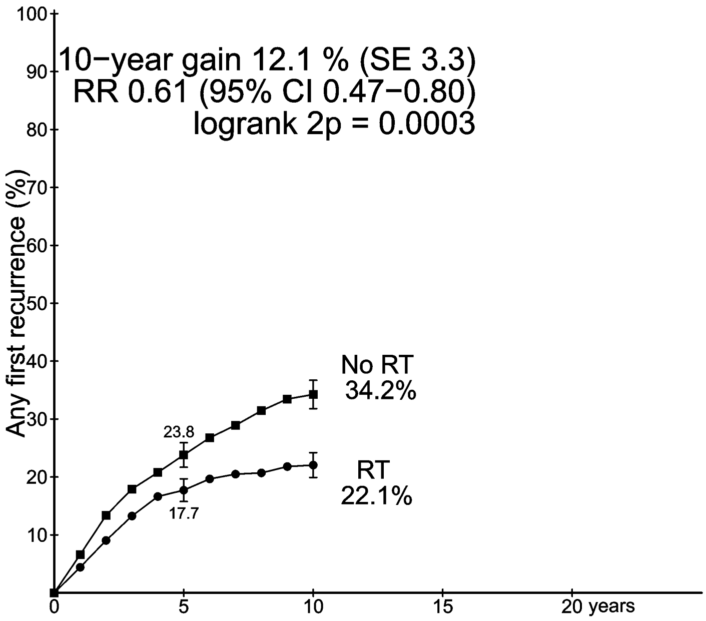

Any first recurrence rates (%/year) and logrank analyses

	Years 0−4	Years 5−9
RT	4.11 (76/1849)	1.20 (18/1504)
No RT	5.58 (104/1862)	3.35 (46/1373)
Rate ratio, from	0.71 SE 0.13	0.40 SE 0.17
(O−E)/V	−13.8/40.7	−12.9/14.0

Breast cancer mortality

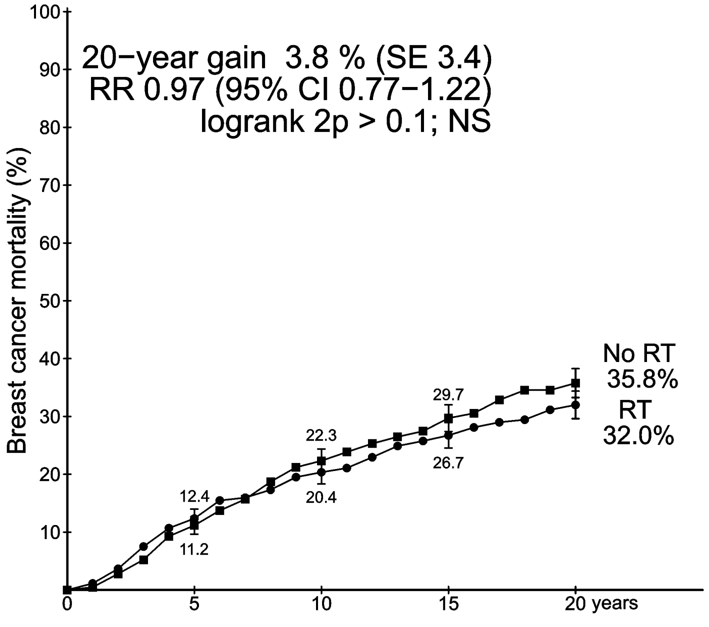

Breast cancer mortality rates (%/year) and logrank analyses

	Years 0−4	Years 5−9	Years 10−14	Years 15−19	Years 20+
RT	2.67 (53/1986)	1.91 (32/1679)	1.51 (22/1455)	1.47 (17/1155)	2.07 (30/1451)
No RT	2.30 (48/2089)	2.72 (47/1729)	2.14 (31/1450)	1.86 (20/1075)	1.85 (25/1349)
Rate ratio, from	1.13 SE 0.22	0.73 SE 0.20	0.84 SE 0.26	0.83 SE 0.31	1.46 SE 0.36
(O−E)/V	2.8/23.4	−5.6/17.7	−2.2/12.6	−1.6/8.5	4.3/11.3

Any death

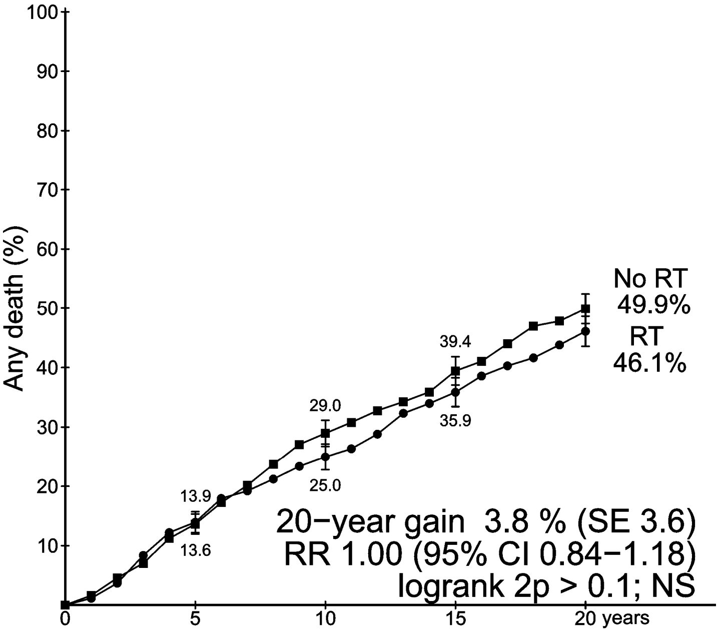

Any death rates (%/year) and logrank analyses

	Years 0−4	Years 5−9	Years 10−14	Years 15−19	Years 20+
RT	3.12 (62/1986)	2.86 (48/1679)	3.16 (46/1455)	3.55 (41/1155)	6.96 (101/1451
No RT	2.97 (62/2089)	3.93 (68/1729)	3.17 (46/1450)	3.91 (42/1075)	5.86 (79/1349)
Rate ratio, from	1.03 SE 0.19	0.71 SE 0.16	0.99 SE 0.21	0.92 SE 0.22	1.29 SE 0.18
(O−E)/V	0.8/28.5	−9.2/26.5	−0.2/21.6	−1.6/18.8	9.8/38.2

Webfigure 26. Effect of radiotherapy (RT) to the chest wall and regional lymph nodes versus not after mastectomy and axillary sampling (Mast+AS): 10-year risk of recurrence and type of first recurrence, by allocated treatment, in 870 women with pathologically node negative (pN0) disease. (r_L = number of women for whom first recurrence was locoregional, r_D = number women for whom distant recurrence was first.)

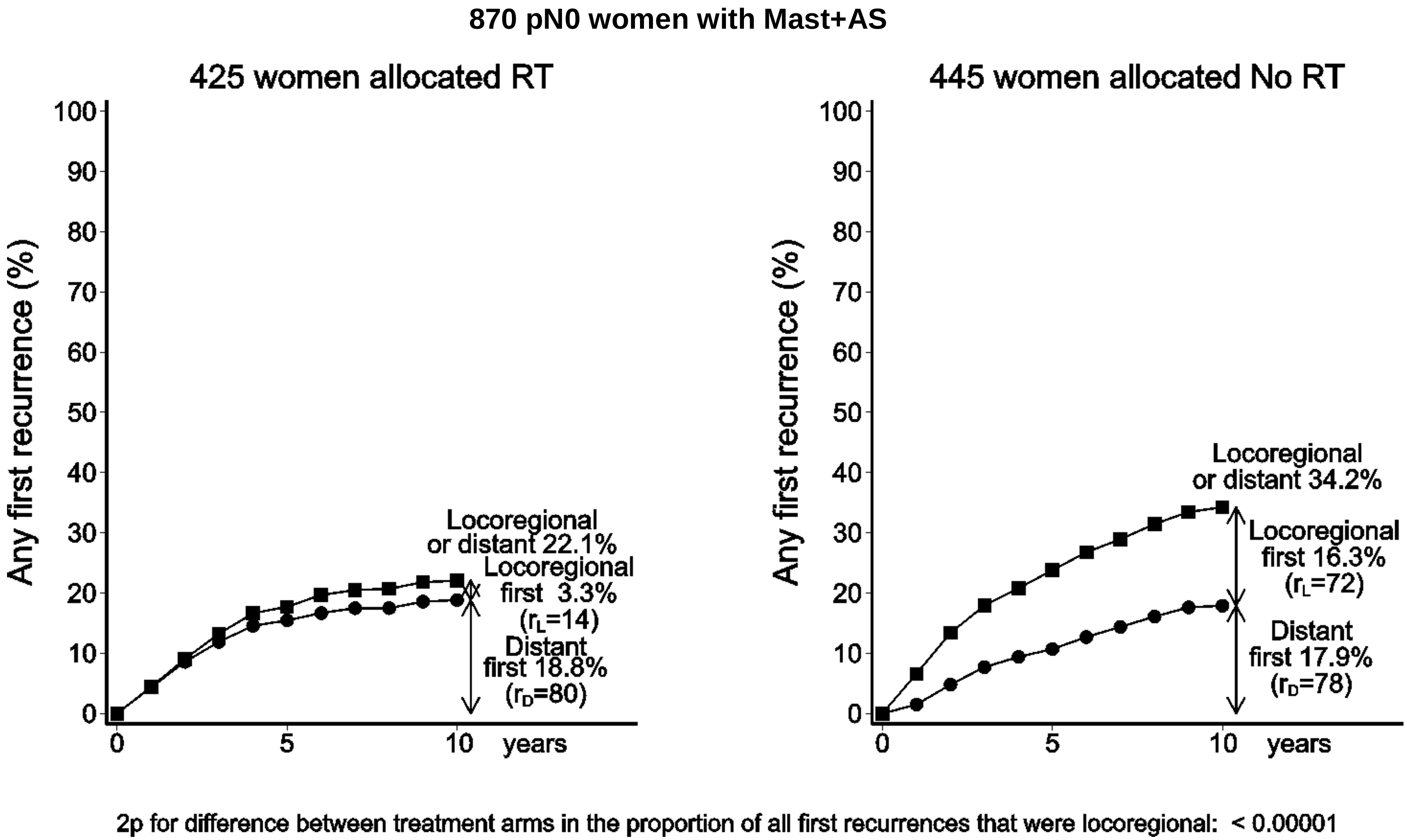

Webfigure 27. Effect of radiotherapy (RT) to the chest wall and regional lymph nodes versus not after mastectomy and axillary sampling (Mast+AS): 10-year risk of locoregional recurrence and recurrence of any type and 20-year risk of breast cancer and all-cause mortality in 2541 women with pathologically node-positive (pN+) disease. See webfigure 1 for methodological note and also webfigure 28.

2541 pN+ women with Mast+AS

Locoregional recurrence first

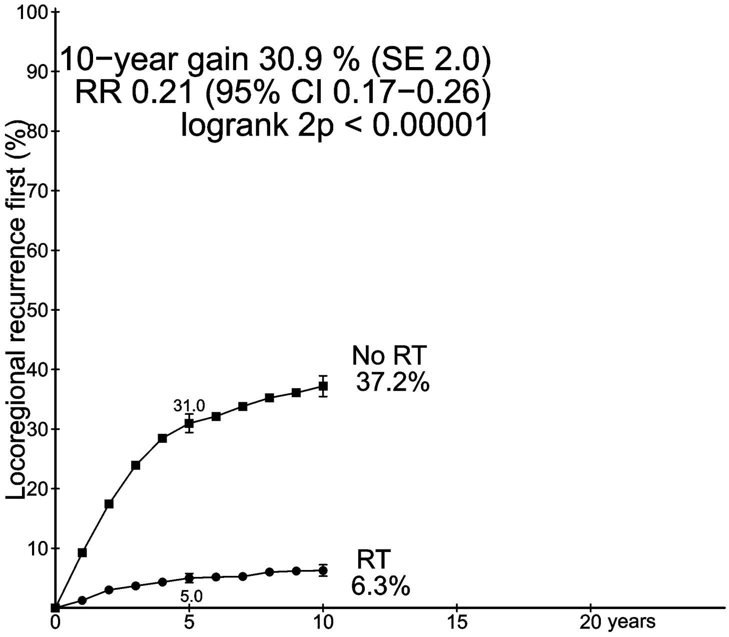

Locoregional recurrence first rates (%/year) and logrank analyses

	Years 0−4	Years 5−9
RT	1.31 (61/4667)	0.44 (13/2977)
No RT	8.10 (325/4011)	2.06 (43/2087)
Rate ratio, from	0.21 SE 0.05	0.22 SE 0.15
(O−E)/V	−139.0/88.6	−18.9/12.6

Any first recurrence

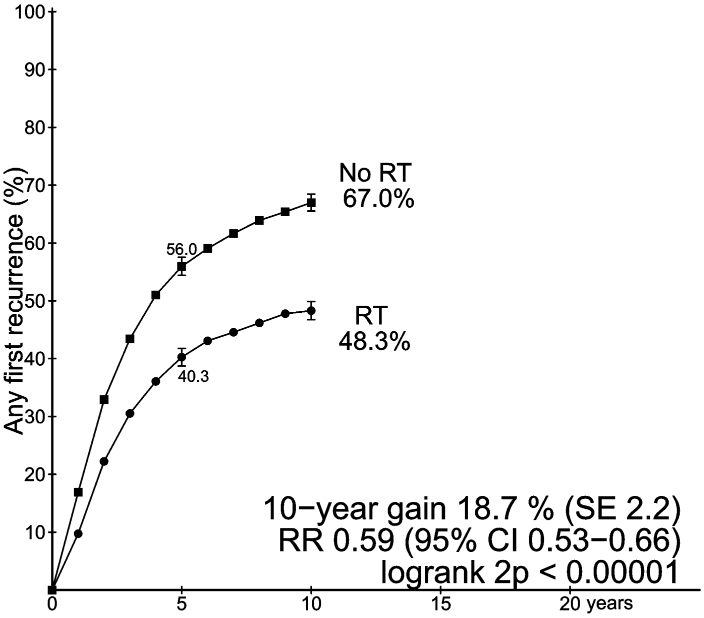

Any first recurrence rates (%/year) and logrank analyses

	Years 0−4	Years 5−9
RT	11.05 (519/4698)	3.41 (102/2996)
No RT	17.29 (700/4050)	6.15 (130/2114)
Rate ratio, from	0.61 SE 0.05	0.51 SE 0.10
(O−E)/V	−120.1/242.0	−33.4/50.0

Breast cancer mortality

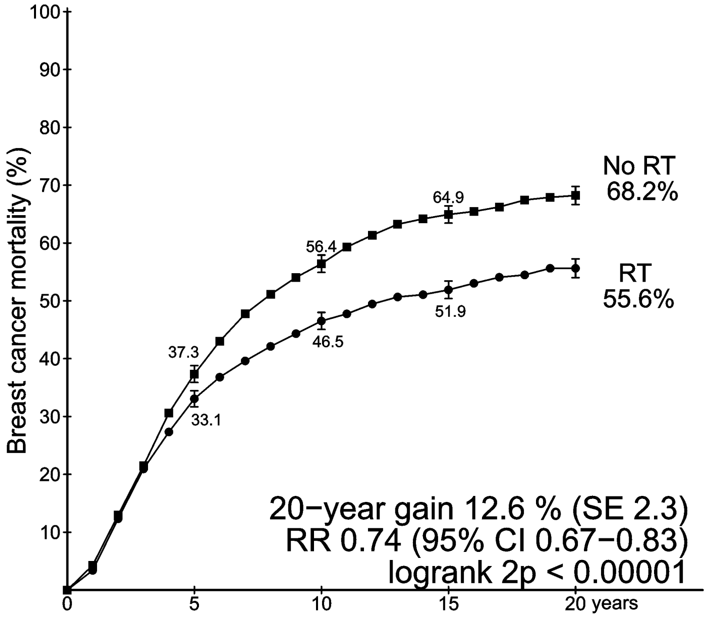

Breast cancer mortality rates (%/year) and logrank analyses

	Years 0−4	Years 5−9	Years 10−14	Years 15−19	Years 20+
RT	7.77 (410/5278)	4.63 (164/3539)	2.30 (57/2473)	1.78 (22/1239)	1.25 (6/480)
No RT	9.16 (473/5163)	7.55 (229/3032)	4.80 (87/1813)	2.14 (19/888)	2.35 (8/340)
Rate ratio, from	0.86 SE 0.07	0.61 SE 0.09	0.48 SE 0.13	0.93 SE 0.33	1.14 SE 0.63
(O−E)/V	−27.7/187.2	−42.2/85.3	−23.0/31.5	−0.6/8.3	0.4/2.9

Any death

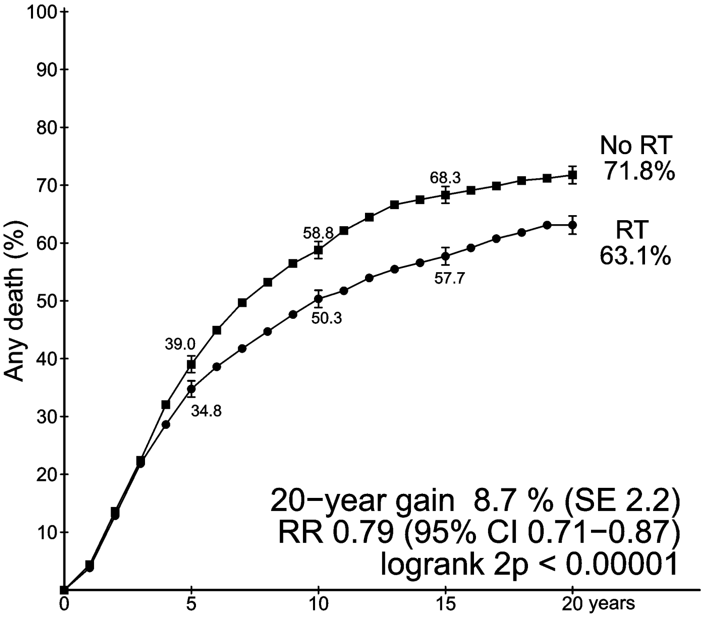

Any death rates (%/year) and logrank analyses

	Years 0−4	Years 5−9	Years 10−14	Years 15−19	Years 20+
RT	8.36 (441/5278)	5.77 (204/3539)	3.56 (88/2473)	3.23 (40/1239)	3.75 (18/480)
No RT	9.72 (502/5163)	8.24 (250/3032)	5.90 (107/1813)	2.70 (24/888)	4.41 (15/340)
Rate ratio, from	0.87 SE 0.07	0.68 SE 0.08	0.59 SE 0.12	1.28 SE 0.31	0.87 SE 0.39
(O−E)/V	−28.9/201.6	−38.0/98.7	−22.7/42.5	3.3/13.1	−0.8/5.7

Webfigure 28. Effect of radiotherapy (RT) to the chest wall and regional lymph nodes versus not after mastectomy and axillary sampling (Mast+AS): 10-year risk of recurrence and type of first recurrence, by allocated treatment, in 2541 women with pathologically node-positive (pN+) disease. (r_L = number of women for whom first recurrence was locoregional, r_D = number women for whom distant recurrence was first.)

2541 pN+ women with Mast+AS

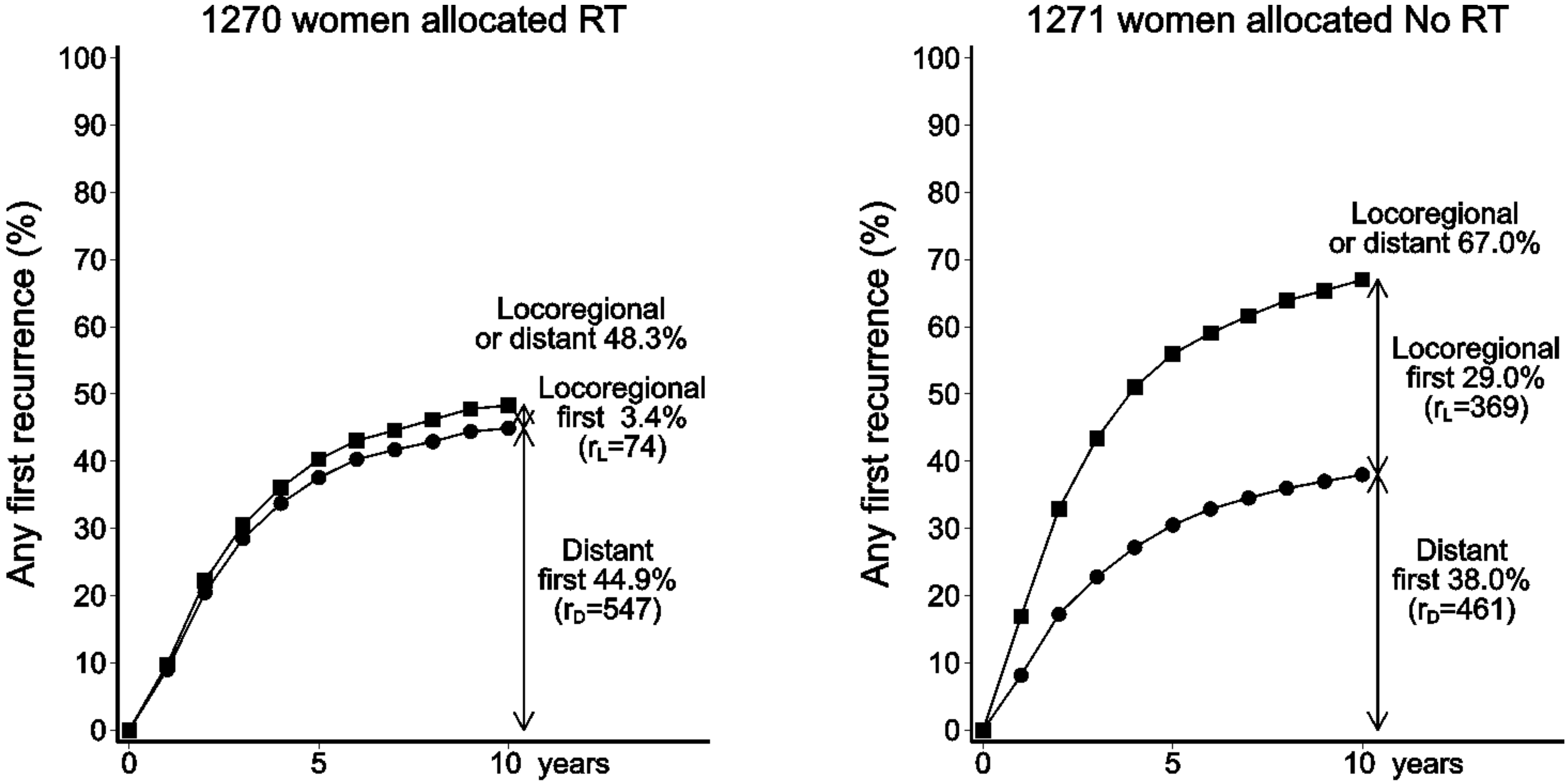

2p for difference between treatment arms in the proportion of all first recurrences that were locoregional: < 0.00001

Webfigure 29. Effect of radiotherapy (RT) to the chest wall and regional lymph nodes versus not after mastectomy and axillary dissection (Mast+AD) or axillary sampling (Mast+AS): Event rate ratios, one line per trial, for locoregional recurrence and recurrence of any type during years 0-9 and for breast cancer and all-cause mortality in 1594 women with pathologically node-negative (pN0) disease.

1594 pN0 women

Locoregional recurrence first (years 0-9)

Year code, and study name	Treatment Information	Events/Women: Allocated RT	Events/Women: Allocated No RT	RT events: Logrank O−E	RT events: Variance of O−E	Ratio of annual event rates RT : No RT
**(a) Axillary dissection**						
64B Oslo X−ray	CW+AF+IMC	2/175	2/174	0.0	1.0	
74D DFCI Boston	†CW+AF+(IMC)	0/8	0/2			
76C Glasgow	†CW+AF+IMC	0/1	0/1			
78A S Sweden II:1	†CW+AF+IMC	6/134	3/144	1.7	2.2	
79G Metaxas Athens	†CW+AF+IMC	0/5	0/5			
82B DBCG 82b ipremenop.	†CW+AF+IMC	1/8	0/10	0.4	0.2	
82C DBCG 82c ipostmenop.	†CW+AF+IMC	0/6	0/12			
82Q ECOG EST3181	†CW+AF+IMC	0/9	0/4			
**(a) Subtotal**		**9/ 346 (2.6%)**	**5/ 352 (1.4%)**	**2.1**	**3.5**	**1.81 (SE 0.73)** 2p > 0.1: NS
**(b) Axillary sampling**						
71B Stockholm A	CW+AF+IMC	4/203	30/196	−13.2	8.2	
73A Southampton UK	CW+AF+IMC	3/23	4/29	0.5	1.4	
74B Edinburgh I	CW+AF	5/114	24/114	−9.6	6.9	
82B DBCG 82b ipremenop.	†CW+AF+IMC	0/36	4/53	−1.9	0.9	
82C DBCG 82c ipostmenop.	†CW+AF+IMC	2/49	10/53	−3.5	2.5	
**(b) Subtotal**		**14/ 425 (3.3%)**	**72/ 445 (16.2%)**	**−27.8**	**19.9**	**0.25 (SE 0.12)** 2p < 0.00001
**(c) Extent of axillary surgery unknown**						
86C CRC, UK	†Various	0/14	1/10	−0.7	0.2	
**(c) Subtotal**		**0/ 14 (0.0%)**	**1/ 10 (10.0%)**	**−0.7**	**0.2**	**0.00 (SE 0.67)** 2p > 0.1: NS
**Total**		**23/ 785 (2.9%)**	**78/ 807 (9.7%)**	**−26.4**	**23.6**	**0.33 (SE 0.12)** 2p < 0.00001

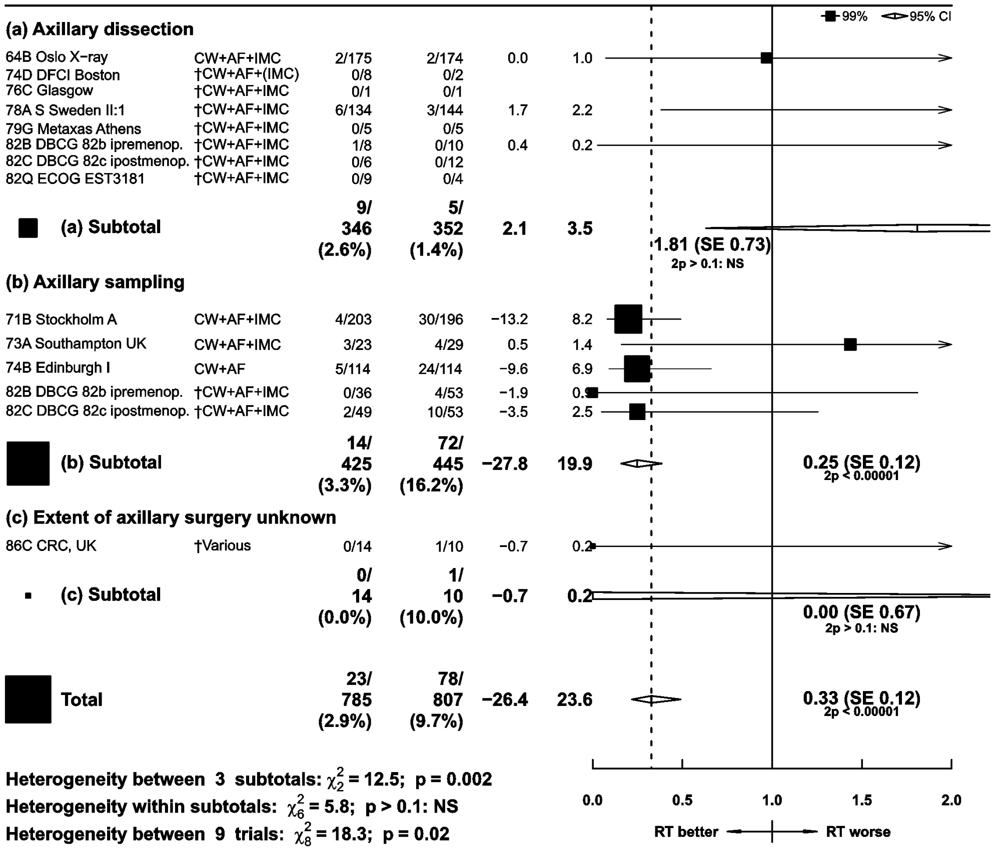

Heterogeneity between 3 subtotals: χ^2_2 = 12.5; p = 0.002
Heterogeneity within subtotals: χ^2_6 = 5.8; p > 0.1: NS
Heterogeneity between 9 trials: χ^2_8 = 18.3; p = 0.02

Any first recurrence (years 0-9)

Year code, and study name	Treatment Information	Events/Women: Allocated RT	Events/Women: Allocated No RT	RT events: Logrank O−E	RT events: Variance of O−E	Ratio of annual event rates RT : No RT
**(a) Axillary dissection**						
64B Oslo X−ray	CW+AF+IMC	28/175	38/174	−5.1	16.1	
74D DFCI Boston	†CW+AF+(IMC)	1/8	1/2	−0.3	0.2	
76C Glasgow	†CW+AF+IMC	1/1	0/1	0.5	0.2	
77J MD Ander. 7730B	†CW+AF+IMC	0/1	0/1			
78A S Sweden II:1	†CW+AF+IMC	34/134	27/144	4.7	14.7	
79G Metaxas Athens	†CW+AF+IMC	2/5	1/5	0.7	0.4	
82B DBCG 82b ipremenop.	†CW+AF+IMC	3/8	2/10	0.3	1.0	
82C DBCG 82c ipostmenop.	†CW+AF+IMC	4/6	3/12	1.7	1.4	
82Q ECOG EST3181	†CW+AF+IMC	2/9	1/4	−0.4	0.5	
**(a) Subtotal**		**75/ 347 (21.6%)**	**73/ 353 (20.7%)**	**2.0**	**34.5**	**1.06 (SE 0.18)** 2p > 0.1: NS
**(b) Axillary sampling**						
71B Stockholm A	CW+AF+IMC	39/203	61/196	−13.1	23.4	
73A Southampton UK	CW+AF+IMC	8/23	11/29	0.1	3.6	
74B Edinburgh I	CW+AF	27/114	40/114	−7.5	15.6	
82B DBCG 82b ipremenop.	†CW+AF+IMC	3/36	14/53	−3.9	3.2	
82C DBCG 82c ipostmenop.	†CW+AF+IMC	17/49	24/53	−2.3	8.7	
**(b) Subtotal**		**94/ 425 (22.1%)**	**150/ 445 (33.7%)**	**−26.8**	**54.6**	**0.61 (SE 0.11)** 2p = 0.0003
**(c) Extent of axillary surgery unknown**						
86C CRC, UK	†Various	2/14	4/10	−1.4	1.4	
**(c) Subtotal**		**2/ 14 (14.3%)**	**4/ 10 (40.0%)**	**−1.4**	**1.4**	**0.38 (SE 0.53)** 2p > 0.1: NS
**Total**		**171/ 786 (21.8%)**	**227/ 808 (28.1%)**	**−26.1**	**90.6**	**0.75 (SE 0.09)** 2p = 0.006

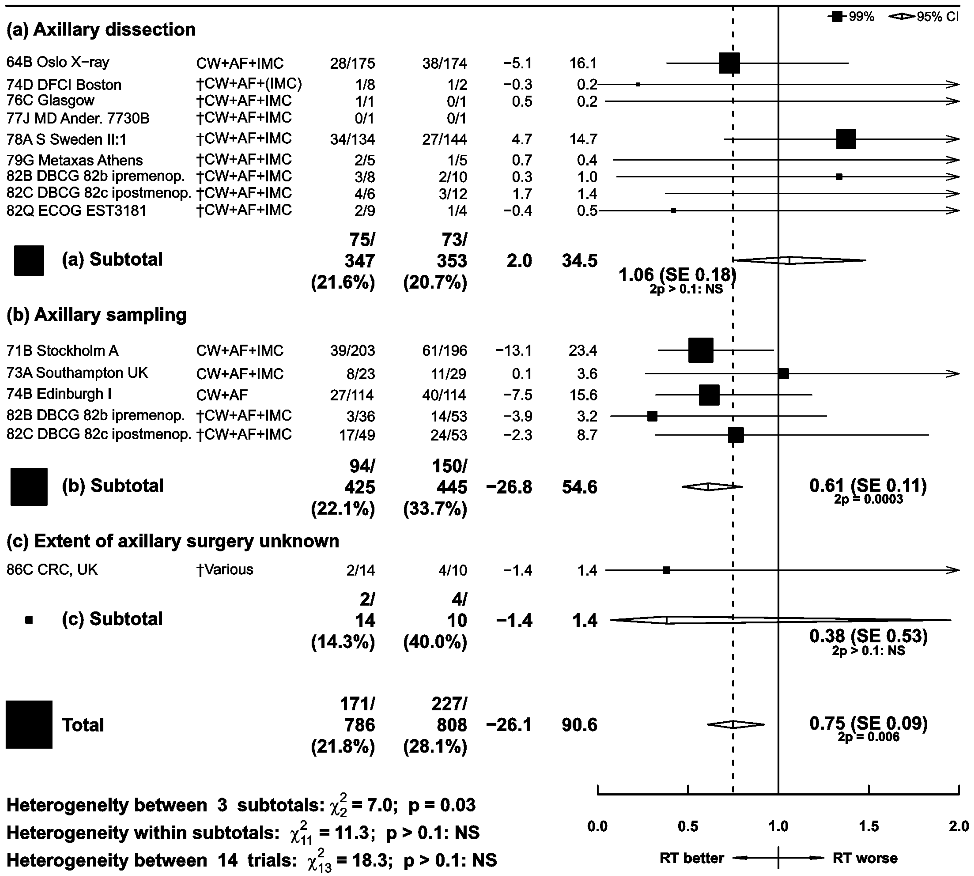

Heterogeneity between 3 subtotals: χ^2_2 = 7.0; p = 0.03
Heterogeneity within subtotals: χ^2_{11} = 11.3; p > 0.1: NS
Heterogeneity between 14 trials: χ^2_{13} = 18.3; p > 0.1: NS

† Same polychemotherapy (usually cyclophosphamide, methotrexate, and 5-fluorouracil), and/or tamoxifen in both groups.
Radiotherapy sites: CW=chest wall, AF=Axilla and/or supraclavicular fossa, IMC=Internal mammary chain. Site(s) in brackets were not always treated.

continued overleaf

Webfigure 29 cntd.

1594 pN0 women

Breast cancer mortality

Year code, and study name	Treatment Information	Deaths/Women: Allocated RT	Deaths/Women: Allocated No RT	RT deaths: Logrank O−E	RT deaths: Variance of O−E	Ratio of annual death rates RT : No RT
**(a) Axillary dissection**						
64B Oslo X−ray	CW+AF+IMC	57/175	62/174	−2.0	27.3	
74D DFCI Boston	†CW+AF+(IMC)	1/8	1/2	−0.3	0.2	
76C Glasgow	†CW+AF+IMC	1/1	0/1	0.5	0.2	
77J MD Ander. 7730B	†CW+AF+IMC	0/1	0/1			
78A S Sweden II:1	†CW+AF+IMC	42/134	34/144	8.5	18.2	
79G Metaxas Athens	†CW+AF+IMC	1/5	1/5	0.3	0.2	
82B DBCG 82b ipremenop.	†CW+AF+IMC	3/8	3/10	−0.2	1.3	
82C DBCG 82c ipostmenop.	†CW+AF+IMC	4/6	4/12	1.6	1.5	
82Q ECOG EST3181	†CW+AF+IMC	2/9	1/4	−0.4	0.5	
**(a) Subtotal**		**111/ 347 (32.0%)**	**106/ 353 (30.0%)**	**8.0**	**49.5**	**1.18 (SE 0.15)** 2p > 0.1: NS
**(b) Axillary sampling**						
71B Stockholm A	CW+AF+IMC	77/203	75/196	2.5	35.7	
73A Southampton UK	CW+AF+IMC	8/23	13/29	−0.6	4.0	
74B Edinburgh I	CW+AF	44/114	50/114	−1.5	20.7	
82B DBCG 82b ipremenop.	†CW+AF+IMC	6/36	14/53	−3.3	4.2	
82C DBCG 82c ipostmenop.	†CW+AF+IMC	19/49	19/53	0.6	8.9	
**(b) Subtotal**		**154/ 425 (36.2%)**	**171/ 445 (38.4%)**	**−2.3**	**73.4**	**0.97 (SE 0.11)** 2p > 0.1: NS
**(c) Extent of axillary surgery unknown**						
86C CRC, UK	†Various	2/14	5/10	−1.4	1.3	
**(c) Subtotal**		**2/ 14 (14.3%)**	**5/ 10 (50.0%)**	**−1.4**	**1.3**	**0.35 (SE 0.53)** 2p > 0.1: NS
**Total**		**267/ 786 (34.0%)**	**282/ 808 (34.9%)**	**4.3**	**124.2**	**1.04 (SE 0.09)** 2p > 0.1: NS

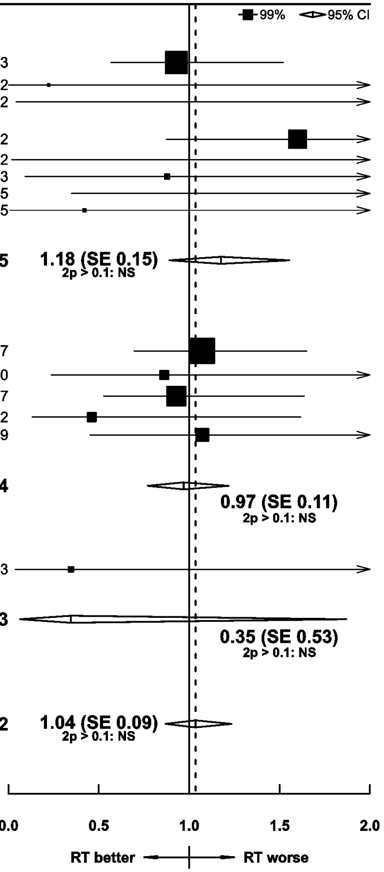

Heterogeneity between 3 subtotals: $\chi^2_2 = 2.7$; p > 0.1: NS
Heterogeneity within subtotals: $\chi^2_{11} = 9.8$; p > 0.1: NS
Heterogeneity between 14 trials: $\chi^2_{13} = 12.5$; p > 0.1: NS

Any death

Year code, and study name	Treatment Information	Deaths/Women: Allocated RT	Deaths/Women: Allocated No RT	RT deaths: Logrank O−E	RT deaths: Variance of O−E	Ratio of annual death rates RT : No RT
**(a) Axillary dissection**						
64B Oslo X−ray	CW+AF+IMC	148/175	150/174	11.3	64.7	
74D DFCI Boston	†CW+AF+(IMC)	1/8	1/2	−0.3	0.2	
76C Glasgow	†CW+AF+IMC	1/1	1/1	0.5	0.2	
77J MD Ander. 7730B	†CW+AF+IMC	0/1	0/1			
78A S Sweden II:1	†CW+AF+IMC	78/134	73/144	8.7	35.2	
79G Metaxas Athens	†CW+AF+IMC	2/5	1/5	0.3	0.2	
82B DBCG 82b ipremenop.	†CW+AF+IMC	3/8	4/10	−0.2	1.3	
82C DBCG 82c ipostmenop.	†CW+AF+IMC	6/6	7/12	1.8	2.6	
82Q ECOG EST3181	†CW+AF+IMC	3/9	1/4	−0.2	0.7	
**(a) Subtotal**		**242/ 347 (69.7%)**	**238/ 353 (67.4%)**	**21.9**	**105.1**	**1.23 (SE 0.11)** 2p = 0.03
**(b) Axillary sampling**						
71B Stockholm A	CW+AF+IMC	153/203	145/196	−0.6	68.3	
73A Southampton UK	CW+AF+IMC	16/23	20/29	1.7	6.8	
74B Edinburgh I	CW+AF	87/114	83/114	2.8	38.0	
82B DBCG 82b ipremenop.	†CW+AF+IMC	11/36	19/53	−2.9	6.4	
82C DBCG 82c ipostmenop.	†CW+AF+IMC	31/49	30/53	−1.3	14.1	
**(b) Subtotal**		**298/ 425 (70.1%)**	**297/ 445 (66.7%)**	**−0.3**	**133.6**	**1.00 (SE 0.09)** 2p > 0.1: NS
**(c) Extent of axillary surgery unknown**						
86C CRC, UK	†Various	4/14	8/10	−2.4	2.4	
**(c) Subtotal**		**4/ 14 (28.6%)**	**8/ 10 (80.0%)**	**−2.4**	**2.4**	**0.37 (SE 0.41)** 2p > 0.1: NS
**Total**		**544/ 786 (69.2%)**	**543/ 808 (67.2%)**	**19.3**	**241.1**	**1.08 (SE 0.07)** 2p > 0.1: NS

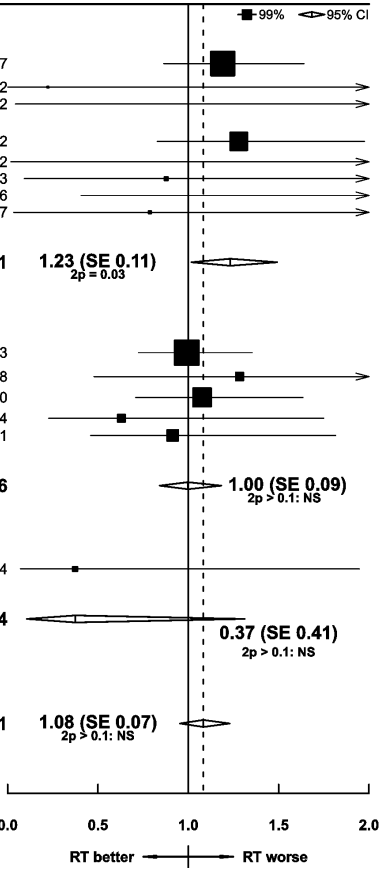

Heterogeneity between 3 subtotals: $\chi^2_2 = 5.4$; p = 0.07
Heterogeneity within subtotals: $\chi^2_{11} = 5.0$; p > 0.1: NS
Heterogeneity between 14 trials: $\chi^2_{13} = 10.4$; p > 0.1: NS

† Same polychemotherapy (usually cyclophosphamide, methotrexate, and 5-fluorouracil), and/or tamoxifen in both groups.
Radiotherapy sites: CW=chest wall, AF=Axilla and/or supraclavicular fossa, IMC=Internal mammary chain. Site(s) in brackets were not always treated.

Webfigure 30. Effect of radiotherapy (RT) to the chest wall and regional lymph nodes versus not after mastectomy and axillary dissection (Mast+AD) or axillary sampling (Mast+AS): Event rate ratios, one line per trial, for locoregional recurrence and recurrence of any type during years 0-9 and for breast cancer and all-cause mortality in 5821 women with pathologically node-positive (pN+) disease.

5821 pN+ women

Locoregional recurrence first (years 0-9)

Year code, and study name	Treatment Information	Events/Women Allocated RT	Events/Women Allocated No RT	RT events Logrank O−E	RT events Variance of O−E	Ratio of annual event rates RT : No RT
**(a) Axillary dissection**						
64B Oslo X−ray	CW+AF+IMC	0/110	10/93	−5.2	2.4	
74D DFCI Boston	†CW+AF+(IMC)	7/103	19/105	−5.2	5.6	
74Q Piedmont OA (pN4+)	†CW+AF+IMC	6/65	9/55	−1.6	2.9	
76A SECSG 1	†CW+AF+IMC	12/126	18/129	−3.5	7.1	
76C Glasgow	†CW+AF+IMC	14/110	29/100	−8.9	9.8	
78A S Sweden II:1	†CW+AF+IMC	9/225	36/228	−14.8	10.6	
78G BCCA Vancouver	†CW+AF+IMC	16/163	34/153	−11.6	11.4	
78Q Düsseldorf U.	†CW+AF+IMC	0/34	1/54	−0.4	0.2	
79G Metaxas Athens	†CW+AF+IMC	4/25	4/36	0.0	2.0	
80S Helsinki	†CW+AF+IMC	4/47	13/52	−4.0	3.2	
82B DBCG 82b ipremenop.	†CW+AF+IMC	9/193	42/207	−17.1	11.5	
82C DBCG 82c ipostmenop.	†CW+AF+IMC	6/157	46/169	−19.5	12.1	
82Q ECOG EST3181	†CW+AF+IMC	12/161	29/157	−8.8	9.5	
**(a) Subtotal**		**99/ 1519 (6.5%)**	**290/ 1538 (18.9%)**	**−100.5**	**88.4**	**0.32 (SE 0.06)** 2p < 0.00001
**(b) Axillary sampling**						
71B Stockholm A	CW+AF+IMC	11/118	44/121	−18.8	12.4	
73A Southampton UK	CW+AF+IMC	5/33	21/38	−8.7	5.3	
74B Edinburgh I	CW+AF	0/0	0/1			
79F Coimbra	†CW+AF+IMC	7/62	9/61	−0.4	3.3	
82B DBCG 82b ipremenop.	†CW+AF+IMC	24/517	140/493	−64.0	38.3	
82C DBCG 82c ipostmenop.	†CW+AF+IMC	19/401	123/406	−55.1	33.1	
84A GBSG 03 Germany	†CW+AF+IMC	2/96	11/100	−4.2	3.1	
85F Nottingham	†CW+AF	6/36	20/41	−6.9	5.7	
**(b) Subtotal**		**74/ 1263 (5.9%)**	**368/ 1261 (29.2%)**	**−157.9**	**101.2**	**0.21 (SE 0.05)** 2p < 0.00001
**(c) Extent of axillary surgery unknown**						
80W NSABC Israel	†CW+AF+IMC	3/54	8/58	−2.7	2.6	
86C CRC, UK	†Various	1/17	5/20	−2.0	0.9	
**(c) Subtotal**		**4/ 71 (5.6%)**	**13/ 78 (16.7%)**	**−4.7**	**3.5**	**0.26 (SE 0.30)** 2p = 0.01
**Total**		**177/ 2853 (6.2%)**	**671/ 2877 (23.3%)**	**−263.1**	**193.2**	**0.26 (SE 0.04)** 2p < 0.00001

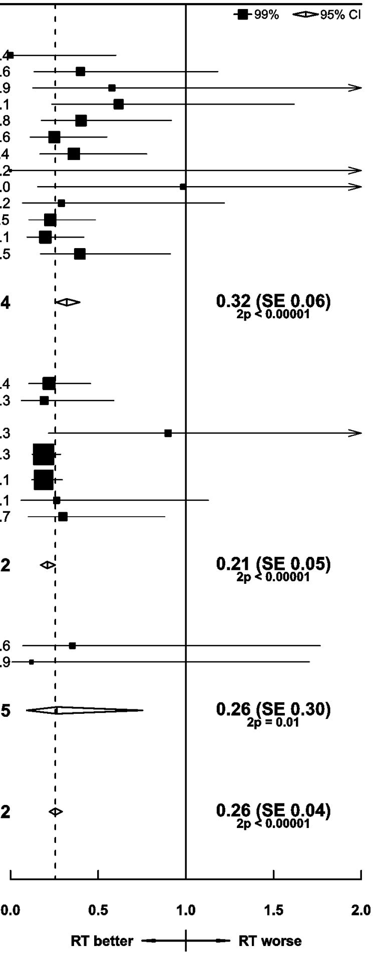

Heterogeneity between 3 subtotals: χ^2_2 = 8.5; p = 0.01
Heterogeneity within subtotals: χ^2_{19} = 24.9; p > 0.1: NS
Heterogeneity between 22 trials: χ^2_{21} = 33.3; p = 0.04

Any first recurrence (years 0-9)

Year code, and study name	Treatment Information	Events/Women Allocated RT	Events/Women Allocated No RT	RT events Logrank O−E	RT events Variance of O−E	Ratio of annual event rates RT : No RT
**(a) Axillary dissection**						
64B Oslo X−ray	CW+AF+IMC	53/110	53/93	−10.2	19.7	
74D DFCI Boston	†CW+AF+(IMC)	55/103	62/105	−1.6	23.7	
74Q Piedmont OA (pN4+)	†CW+AF+IMC	41/65	40/55	−2.6	14.9	
76A SECSG 1	†CW+AF+IMC	62/126	83/129	−10.8	28.6	
76C Glasgow	†CW+AF+IMC	61/110	63/100	−6.5	25.0	
77J MD Ander. 7730B	†CW+AF+IMC	22/31	24/43	6.4	6.5	
78A S Sweden II:1	†CW+AF+IMC	90/225	119/228	−25.1	43.0	
78G BCCA Vancouver	†CW+AF+IMC	71/163	88/153	−16.2	32.8	
78Q Düsseldorf U.	†CW+AF+IMC	17/34	21/54	3.6	7.4	
79G Metaxas Athens	†CW+AF+IMC	14/25	22/36	−3.5	5.6	
80S Helsinki	†CW+AF+IMC	19/47	23/52	−1.7	7.7	
82B DBCG 82b ipremenop.	†CW+AF+IMC	100/193	135/207	−21.9	45.7	
82C DBCG 82c ipostmenop.	†CW+AF+IMC	97/157	119/169	−13.8	43.0	
82Q ECOG EST3181	†CW+AF+IMC	100/161	88/157	5.6	38.5	
**(a) Subtotal**		**802/ 1550 (51.7%)**	**940/ 1581 (59.5%)**	**−98.4**	**342.1**	**0.75 (SE 0.05)** 2p < 0.00001
**(b) Axillary sampling**						
71B Stockholm A	CW+AF+IMC	60/118	84/121	−18.9	28.4	
73A Southampton UK	CW+AF+IMC	15/33	34/38	−12.3	8.3	
74B Edinburgh I	CW+AF	0/0	0/1			
77J MD Ander. 7730B	†CW+AF+IMC	4/7	5/10	0.5	0.2	
79F Coimbra	†CW+AF+IMC	27/62	38/61	−4.7	11.5	
82B DBCG 82b ipremenop.	†CW+AF+IMC	245/517	326/493	−71.4	117.1	
82C DBCG 82c ipostmenop.	†CW+AF+IMC	213/401	256/406	−33.3	97.6	
84A GBSG 03 Germany	†CW+AF+IMC	35/96	53/100	−7.0	18.2	
85F Nottingham	†CW+AF	22/36	34/41	−6.5	10.6	
**(b) Subtotal**		**621/ 1270 (48.9%)**	**830/ 1271 (65.3%)**	**−153.5**	**292.0**	**0.59 (SE 0.05)** 2p < 0.00001
**(c) Extent of axillary surgery unknown**						
80W NSABC Israel	†CW+AF+IMC	22/54	22/58	−0.9	9.5	
86C CRC, UK	†Various	11/17	10/20	−0.5	2.5	
**(c) Subtotal**		**33/ 71 (46.5%)**	**32/ 78 (41.0%)**	**−1.4**	**12.0**	**0.89 (SE 0.27)** 2p > 0.1: NS
**Total**		**1456/ 2891 (50.4%)**	**1802/ 2930 (61.5%)**	**−253.3**	**646.1**	**0.68 (SE 0.03)** 2p < 0.00001

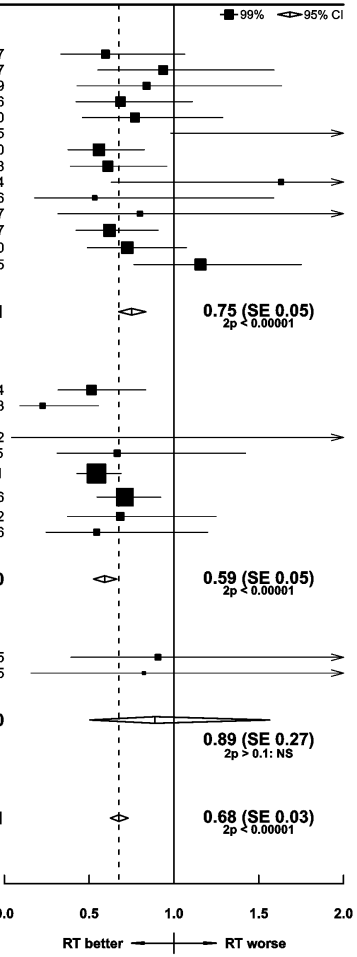

Heterogeneity between 3 subtotals: χ^2_2 = 9.9; p = 0.007
Heterogeneity within subtotals: χ^2_{21} = 47.1; p = 0.0009
Heterogeneity between 24 trials: χ^2_{23} = 56.9; p = 0.0001

† Same polychemotherapy (usually cyclophosphamide, methotrexate, and 5-fluorouracil), and/or tamoxifen in both groups.
Radiotherapy sites: CW=chest wall, AF=Axilla and/or supraclavicular fossa, IMC=Internal mammary chain. Site(s) in brackets were not always treated.

continued overleaf

Webfigure 30 cntd.

5821 pN+ women

Breast cancer mortality

Year code, and study name	Treatment Information	Deaths/Women: Allocated RT	Deaths/Women: Allocated No RT	RT deaths: Logrank O−E	RT deaths: Variance of O−E	Ratio of annual death rates RT : No RT
**(a) Axillary dissection**						
64B Oslo X−ray	CW+AF+IMC	68/110	63/93	−8.0	25.1	
74D DFCI Boston	†CW+AF+(IMC)	46/103	53/105	0.1	20.5	
74Q Piedmont OA (pN4+)	†CW+AF+IMC	36/65	40/55	−3.5	14.3	
76A SECSG 1	†CW+AF+IMC	54/126	65/129	−3.7	24.7	
76C Glasgow	†CW+AF+IMC	63/110	69/100	−8.0	25.6	
77J MD Ander. 7730B	†CW+AF+IMC	23/31	24/43	6.0	6.9	
78A S Sweden II:1	†CW+AF+IMC	106/225	131/228	−18.7	51.2	
78G BCCA Vancouver	†CW+AF+IMC	79/163	96/153	−16.8	39.1	
78Q Düsseldorf U.	†CW+AF+IMC	14/34	14/54	4.9	5.1	
79G Metaxas Athens	†CW+AF+IMC	11/25	21/36	−3.4	5.9	
80S Helsinki	†CW+AF+IMC	22/47	20/52	0.9	8.1	
82B DBCG 82b ipremenop.	†CW+AF+IMC	104/193	138/207	−16.8	51.6	
82C DBCG 82c ipostmenop.	†CW+AF+IMC	103/157	116/169	−1.0	46.6	
82Q ECOG EST3181	†CW+AF+IMC	103/161	91/157	6.0	42.4	
**(a) Subtotal**		**832/ 1550 (53.7%)**	**941/ 1581 (59.5%)**	**−62.0**	**367.0**	**0.84 (SE 0.05)** 2p = 0.001
**(b) Axillary sampling**						
71B Stockholm A	CW+AF+IMC	67/118	96/121	−12.8	36.7	
73A Southampton UK	CW+AF+IMC	23/33	32/38	−5.0	10.4	
74B Edinburgh I	CW+AF	0/0	0/1			
77J MD Ander. 7730B	†CW+AF+IMC	5/7	6/10	0.0	0.5	
79F Coimbra	†CW+AF+IMC	31/62	36/61	−0.1	11.4	
82B DBCG 82b ipremenop.	†CW+AF+IMC	253/517	320/493	−54.3	125.3	
82C DBCG 82c ipostmenop.	†CW+AF+IMC	224/401	254/406	−16.1	104.2	
84A GBSG 03 Germany	†CW+AF+IMC	34/96	44/100	−1.8	16.3	
85F Nottingham	†CW+AF	22/36	28/41	−2.9	10.3	
**(b) Subtotal**		**659/ 1270 (51.9%)**	**816/ 1271 (64.2%)**	**−93.1**	**315.3**	**0.74 (SE 0.05)** 2p < 0.00001
**(c) Extent of axillary surgery unknown**						
80W NSABC Israel	†CW+AF+IMC	21/54	20/58	0.6	8.4	
86C CRC, UK	†Various	11/17	9/20	0.3	2.7	
**(c) Subtotal**		**32/ 71 (45.1%)**	**29/ 78 (37.2%)**	**1.0**	**11.0**	**1.09 (SE 0.31)** 2p > 0.1: NS
**Total**		**1523/ 2891 (52.7%)**	**1786/ 2930 (61.0%)**	**−154.1**	**693.3**	**0.80 (SE 0.03)** 2p < 0.00001

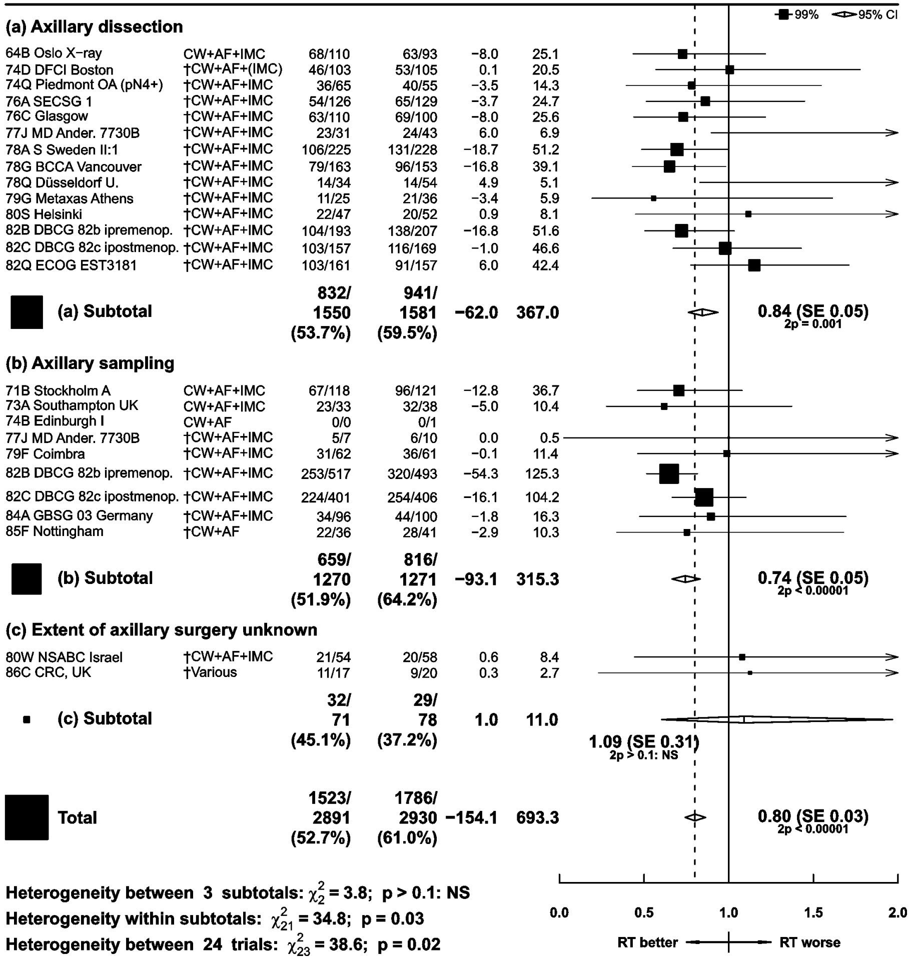

Heterogeneity between 3 subtotals: $\chi^2_2 = 3.8$; p > 0.1: NS
Heterogeneity within subtotals: $\chi^2_{21} = 34.8$; p = 0.03
Heterogeneity between 24 trials: $\chi^2_{23} = 38.6$; p = 0.02

Any death

Year code, and study name	Treatment Information	Deaths/Women: Allocated RT	Deaths/Women: Allocated No RT	RT deaths: Logrank O−E	RT deaths: Variance of O−E	Ratio of annual death rates RT : No RT
**(a) Axillary dissection**						
64B Oslo X−ray	CW+AF+IMC	101/110	85/93	−5.2	35.9	
74D DFCI Boston	†CW+AF+(IMC)	57/103	55/105	3.0	22.8	
74Q Piedmont OA (pN4+)	†CW+AF+IMC	41/65	41/55	−1.6	15.2	
76A SECSG 1	†CW+AF+IMC	60/126	69/129	−3.2	26.9	
76C Glasgow	†CW+AF+IMC	77/110	81/100	−7.4	31.4	
77J MD Ander. 7730B	†CW+AF+IMC	24/31	24/43	6.5	7.2	
78A S Sweden II:1	†CW+AF+IMC	149/225	161/228	−16.1	67.5	
78G BCCA Vancouver	†CW+AF+IMC	89/163	100/153	−15.5	42.1	
78Q Düsseldorf U.	†CW+AF+IMC	17/34	24/54	3.3	7.8	
79G Metaxas Athens	†CW+AF+IMC	11/25	21/36	−3.4	5.9	
80S Helsinki	†CW+AF+IMC	24/47	25/52	1.5	9.0	
82B DBCG 82b ipremenop.	†CW+AF+IMC	111/193	144/207	−17.0	54.7	
82C DBCG 82c ipostmenop.	†CW+AF+IMC	122/157	131/169	−1.1	54.1	
82Q ECOG EST3181	†CW+AF+IMC	118/161	112/157	4.2	50.0	
**(a) Subtotal**		**1001/ 1550 (64.6%)**	**1073/ 1581 (67.9%)**	**−52.1**	**430.4**	**0.89 (SE 0.05)** 2p = 0.01
**(b) Axillary sampling**						
71B Stockholm A	CW+AF+IMC	93/118	104/121	−11.2	42.6	
73A Southampton UK	CW+AF+IMC	28/33	32/38	−3.5	11.1	
74B Edinburgh I	CW+AF	0/0	1/1			
77J MD Ander. 7730B	†CW+AF+IMC	5/7	6/10	0.0	0.5	
79F Coimbra	†CW+AF+IMC	41/62	42/61	1.7	14.9	
82B DBCG 82b ipremenop.	†CW+AF+IMC	284/517	328/493	−47.2	134.4	
82C DBCG 82c ipostmenop.	†CW+AF+IMC	272/401	307/406	−24.7	127.2	
84A GBSG 03 Germany	†CW+AF+IMC	45/96	48/100	1.3	19.9	
85F Nottingham	†CW+AF	23/36	30/41	−3.6	11.0	
**(b) Subtotal**		**791/ 1270 (62.3%)**	**898/ 1271 (70.7%)**	**−87.1**	**361.6**	**0.79 (SE 0.05)** 2p < 0.00001
**(c) Extent of axillary surgery unknown**						
80W NSABC Israel	†CW+AF+IMC	23/54	20/58	1.6	8.9	
86C CRC, UK	†Various	13/17	13/20	0.0	3.4	
**(c) Subtotal**		**36/ 71 (50.7%)**	**33/ 78 (42.3%)**	**1.6**	**12.3**	**1.14 (SE 0.30)** 2p > 0.1: NS
**Total**		**1828/ 2891 (63.2%)**	**2004/ 2930 (68.4%)**	**−137.6**	**804.3**	**0.84 (SE 0.03)** 2p < 0.00001

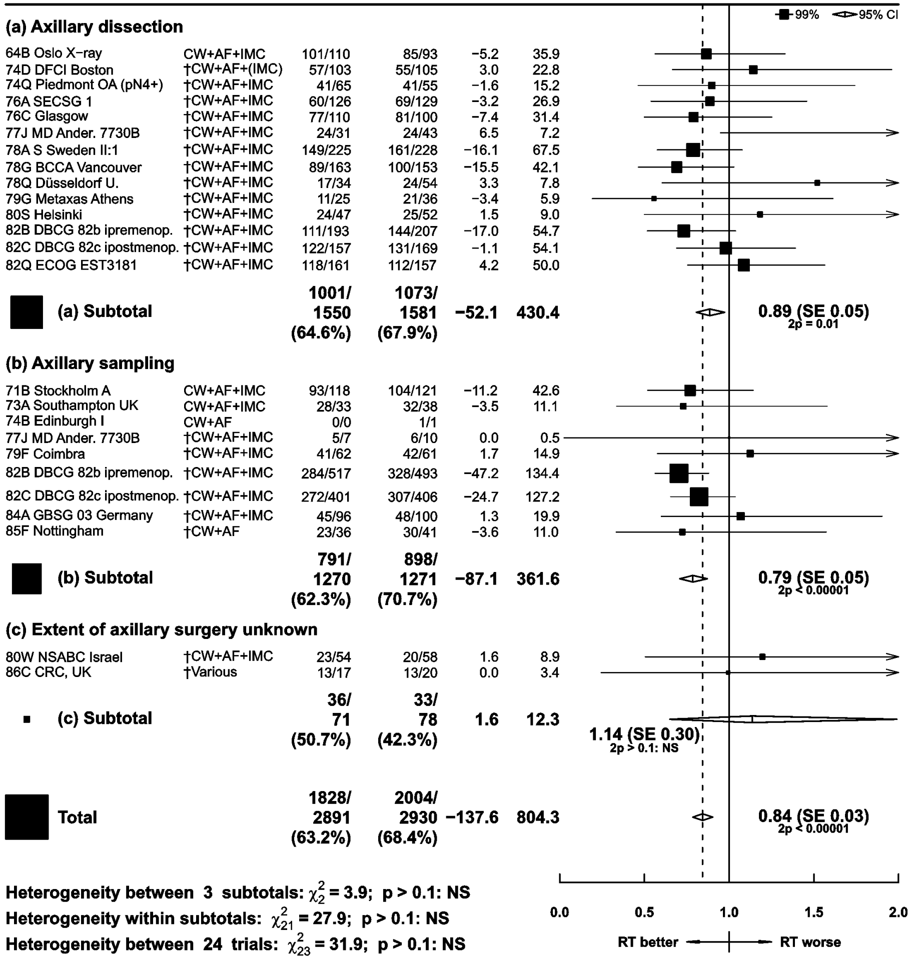

Heterogeneity between 3 subtotals: $\chi^2_2 = 3.9$; p > 0.1: NS
Heterogeneity within subtotals: $\chi^2_{21} = 27.9$; p > 0.1: NS
Heterogeneity between 24 trials: $\chi^2_{23} = 31.9$; p > 0.1: NS

† Same polychemotherapy (usually cyclophosphamide, methotrexate, and 5-fluorouracil), and/or tamoxifen in both groups.
Radiotherapy sites: CW=chest wall, AF=Axilla and/or supraclavicular fossa, IMC=Internal mammary chain. Site(s) in brackets were not always treated.

Webfigure 31. Effect of radiotherapy (RT) to the chest wall and regional lymph nodes versus not after mastectomy and axillary dissection (Mast+AD) or axillary sampling (Mast+AS): Event rate ratios, one line per trial, for locoregional recurrence and recurrence of any type during years 0-9 and for breast cancer and all-cause mortality in 2801 women with 1-3 pathologically positive nodes (pN1-3).

2801 pN1-3 women

Locoregional recurrence first (years 0-9)

Year code, and study name	Treatment Information	Events/Women: Allocated RT	Events/Women: Allocated No RT	RT events: Logrank O−E	RT events: Variance of O−E	Ratio of annual event rates RT : No RT
**(a) Axillary dissection**						
64B Oslo X−ray	CW+AF+IMC	0/80	6/73	−3.1	1.5	
74D DFCI Boston	†CW+AF+(IMC)	1/37	3/41	−0.9	1.0	
76A SECSG 1	†CW+AF+IMC	0/1	0/0			
76C Glasgow	†CW+AF+IMC	3/70	19/69	−8.1	5.2	
78A S Sweden II:1	†CW+AF+IMC	4/140	25/155	−10.6	6.9	
78G BCCA Vancouver	†CW+AF+IMC	7/91	14/92	−3.6	5.0	
79G Metaxas Athens	†CW+AF+IMC	0/7	1/11	−0.5	0.2	
80S Helsinki	†CW+AF+IMC	1/29	10/38	−3.6	2.6	
82B DBCG 82b ipremenop.	†CW+AF+IMC	1/83	13/79	−6.3	3.1	
82C DBCG 82c ipostmenop.	†CW+AF+IMC	1/53	19/75	−7.3	4.7	
82Q ECOG EST3181	†CW+AF+IMC	1/34	2/36	−0.6	0.7	
**(a) Subtotal**		**19/ 625 (3.0%)**	**112/ 669 (16.7%)**	**−44.5**	**30.9**	**0.24 (SE 0.10)** 2p < 0.00001
**(b) Axillary sampling**						
71B Stockholm A	CW+AF+IMC	5/43	12/42	−3.7	3.8	
79F Coimbra	†CW+AF+IMC	1/28	4/29	−1.4	1.2	
82B DBCG 82b ipremenop.	†CW+AF+IMC	12/344	82/322	−38.3	22.4	
82C DBCG 82c ipostmenop.	†CW+AF+IMC	11/245	59/240	−25.6	16.9	
84A GBSG 03 Germany	†CW+AF+IMC	1/62	5/57	−2.3	1.5	
**(b) Subtotal**		**30/ 722 (4.2%)**	**162/ 690 (23.5%)**	**−71.3**	**45.8**	**0.21 (SE 0.07)** 2p < 0.00001
**(c) Extent of axillary surgery unknown**						
80W NSABC Israel	†CW+AF+IMC	1/20	5/19	−2.4	1.5	
86C CRC, UK	†Various	0/10	4/18	−1.3	0.7	
**(c) Subtotal**		**1/ 30 (3.3%)**	**9/ 37 (24.3%)**	**−3.7**	**2.2**	**0.18 (SE 0.32)** 2p = 0.01
**Total**		**50/ 1377 (3.6%)**	**283/ 1396 (20.3%)**	**−119.6**	**78.8**	**0.22 (SE 0.06)** 2p < 0.00001

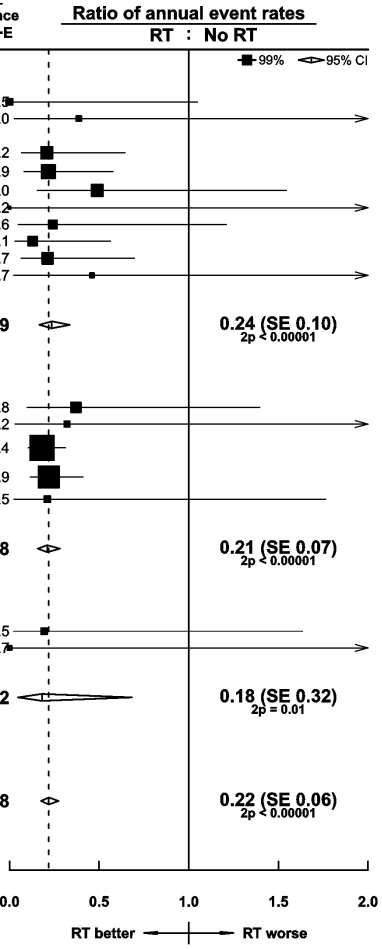

Heterogeneity between 3 subtotals: $\chi^2_2 = 0.3$; p > 0.1: NS
Heterogeneity within subtotals: $\chi^2_{14} = 7.1$; p > 0.1: NS
Heterogeneity between 17 trials: $\chi^2_{16} = 7.5$; p > 0.1: NS

Any first recurrence (years 0-9)

Year code, and study name	Treatment Information	Events/Women: Allocated RT	Events/Women: Allocated No RT	RT events: Logrank O−E	RT events: Variance of O−E	Ratio of annual event rates RT : No RT
**(a) Axillary dissection**						
64B Oslo X−ray	CW+AF+IMC	29/80	35/73	−4.2	14.6	
74D DFCI Boston	†CW+AF+(IMC)	12/37	15/41	−1.1	6.0	
76A SECSG 1	†CW+AF+IMC	0/1	0/0			
76C Glasgow	†CW+AF+IMC	32/70	39/69	−5.2	15.5	
77J MD Ander. 7730B	†CW+AF+IMC	5/7	7/13	1.6	1.3	
78A S Sweden II:1	†CW+AF+IMC	34/140	65/155	−17.7	21.8	
78G BCCA Vancouver	†CW+AF+IMC	30/91	41/92	−6.3	16.2	
79G Metaxas Athens	†CW+AF+IMC	3/7	6/11	−1.2	1.2	
80S Helsinki	†CW+AF+IMC	7/29	18/38	−3.1	5.5	
82B DBCG 82b ipremenop.	†CW+AF+IMC	22/83	30/79	−6.9	11.0	
82C DBCG 82c ipostmenop.	†CW+AF+IMC	18/53	36/75	−4.5	12.2	
82Q ECOG EST3181	†CW+AF+IMC	19/34	12/36	6.4	6.3	
**(a) Subtotal**		**211/ 632 (33.4%)**	**304/ 682 (44.6%)**	**−42.3**	**111.7**	**0.68 (SE 0.08)** 2p = 0.00006
**(b) Axillary sampling**						
71B Stockholm A	CW+AF+IMC	21/43	25/42	−3.3	9.7	
77J MD Ander. 7730B	†CW+AF+IMC	3/4	3/4	0.5	0.2	
79F Coimbra	†CW+AF+IMC	7/28	15/29	−3.5	4.9	
82B DBCG 82b ipremenop.	†CW+AF+IMC	143/344	190/322	−39.1	73.8	
82C DBCG 82c ipostmenop.	†CW+AF+IMC	113/245	133/240	−17.7	55.5	
84A GBSG 03 Germany	†CW+AF+IMC	16/62	24/57	−4.6	8.5	
**(b) Subtotal**		**303/ 726 (41.7%)**	**390/ 694 (56.2%)**	**−67.7**	**152.8**	**0.64 (SE 0.07)** 2p < 0.00001
**(c) Extent of axillary surgery unknown**						
80W NSABC Israel	†CW+AF+IMC	12/20	15/19	−2.4	5.6	
86C CRC, UK	†Various	5/10	8/18	0.2	2.2	
**(c) Subtotal**		**17/ 30 (56.7%)**	**23/ 37 (62.2%)**	**−2.2**	**7.9**	**0.76 (SE 0.31)** 2p > 0.1: NS
**Total**		**531/ 1388 (38.3%)**	**717/ 1413 (50.7%)**	**−112.2**	**272.3**	**0.66 (SE 0.05)** 2p < 0.00001

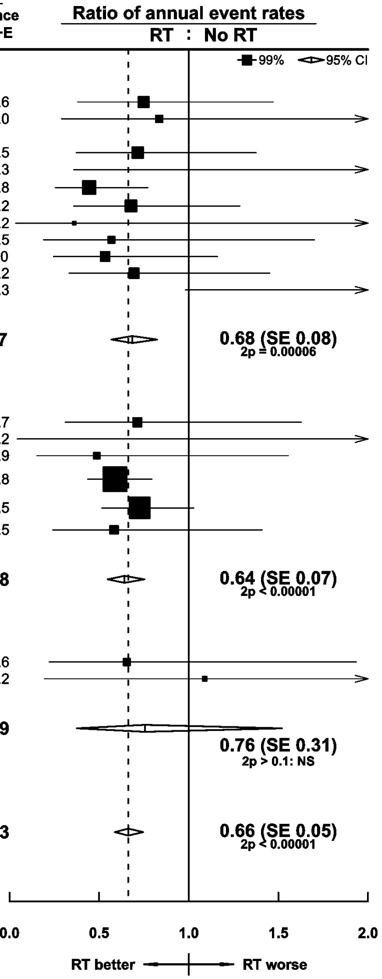

Heterogeneity between 3 subtotals: $\chi^2_2 = 0.4$; p > 0.1: NS
Heterogeneity within subtotals: $\chi^2_{16} = 25.3$; p = 0.07
Heterogeneity between 19 trials: $\chi^2_{18} = 25.7$; p > 0.1: NS

† Same polychemotherapy (usually cyclophosphamide, methotrexate, and 5-fluorouracil), and/or tamoxifen in both groups.
Radiotherapy sites: CW=chest wall, AF=Axilla and/or supraclavicular fossa, IMC=Internal mammary chain. Site(s) in brackets were not always treated.

continued overleaf

Webfigure 31 cntd.

2801 pN1-3 women

Breast cancer mortality

Year code, and study name	Treatment Information	Deaths/Women: Allocated RT	Deaths/Women: Allocated No RT	RT deaths: Logrank O−E	RT deaths: Variance of O−E	Ratio of annual death rates RT : No RT
**(a) Axillary dissection**						
64B Oslo X-ray	CW+AF+IMC	41/80	45/73	−2.0	19.5	
74D DFCI Boston	†CW+AF+(IMC)	9/37	12/41	0.2	4.6	
76A SECSG 1	†CW+AF+IMC	0/1	0/0			
76C Glasgow	†CW+AF+IMC	33/70	42/69	−4.1	15.8	
77J MD Ander. 7730B	†CW+AF+IMC	5/7	7/13	0.6	1.3	
78A S Sweden II:1	†CW+AF+IMC	48/140	75/155	−14.0	27.3	
78G BCCA Vancouver	†CW+AF+IMC	34/91	45/92	−6.8	19.0	
79G Metaxas Athens	†CW+AF+IMC	3/7	6/11	−1.1	1.2	
80S Helsinki	†CW+AF+IMC	9/29	16/38	−1.1	5.4	
82B DBCG 82b ipremenop.	†CW+AF+IMC	25/83	31/79	−5.3	12.5	
82C DBCG 82c ipostmenop.	†CW+AF+IMC	22/53	35/75	−0.6	12.7	
82Q ECOG EST3181	†CW+AF+IMC	19/34	11/36	5.8	6.7	
**(a) Subtotal**		**248/ 632 (39.2%)**	**325/ 682 (47.7%)**	**−28.4**	**125.9**	**0.80 (SE 0.08)** 2p = 0.01
**(b) Axillary sampling**						
71B Stockholm A	CW+AF+IMC	23/43	32/42	−1.6	12.8	
77J MD Ander. 7730B	†CW+AF+IMC	4/4	3/4	0.0	0.5	
79F Coimbra	†CW+AF+IMC	8/28	13/29	−1.7	4.5	
82B DBCG 82b ipremenop.	†CW+AF+IMC	152/344	188/322	−28.6	78.4	
82C DBCG 82c ipostmenop.	†CW+AF+IMC	126/245	138/240	−12.1	59.6	
84A GBSG 03 Germany	†CW+AF+IMC	16/62	20/57	−1.6	7.8	
**(b) Subtotal**		**329/ 726 (45.3%)**	**394/ 694 (56.8%)**	**−45.6**	**163.5**	**0.76 (SE 0.07)** 2p = 0.0004
**(c) Extent of axillary surgery unknown**						
80W NSABC Israel	†CW+AF+IMC	12/20	13/19	−1.1	4.9	
86C CRC, UK	†Various	6/10	7/18	0.5	2.2	
**(c) Subtotal**		**18/ 30 (60.0%)**	**20/ 37 (54.1%)**	**−0.6**	**7.1**	**0.92 (SE 0.36)** 2p > 0.1: NS
**Total**		**595/ 1388 (42.9%)**	**739/ 1413 (52.3%)**	**−74.6**	**296.5**	**0.78 (SE 0.05)** 2p = 0.00001

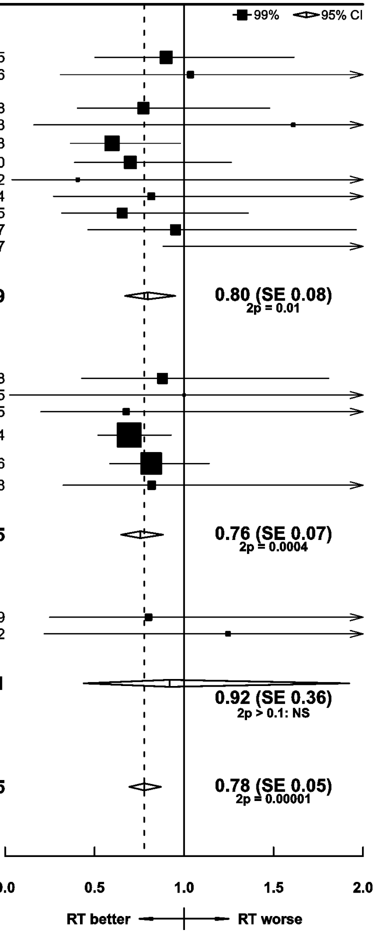

Heterogeneity between 3 subtotals: $\chi^2_2 = 0.4$; p > 0.1: NS
Heterogeneity within subtotals: $\chi^2_{16} = 15.0$; p > 0.1: NS
Heterogeneity between 19 trials: $\chi^2_{18} = 15.4$; p > 0.1: NS

Any death

Year code, and study name	Treatment Information	Deaths/Women: Allocated RT	Deaths/Women: Allocated No RT	RT deaths: Logrank O−E	RT deaths: Variance of O−E	Ratio of annual death rates RT : No RT
**(a) Axillary dissection**						
64B Oslo X-ray	CW+AF+IMC	71/80	65/73	1.4	29.6	
74D DFCI Boston	†CW+AF+(IMC)	14/37	12/41	2.0	5.4	
76A SECSG 1	†CW+AF+IMC	0/1	0/0			
76C Glasgow	†CW+AF+IMC	45/70	52/69	−3.2	20.6	
77J MD Ander. 7730B	†CW+AF+IMC	5/7	7/13	0.6	1.3	
78A S Sweden II:1	†CW+AF+IMC	80/140	99/155	−11.2	40.1	
78G BCCA Vancouver	†CW+AF+IMC	41/91	49/92	−6.4	21.4	
79G Metaxas Athens	†CW+AF+IMC	3/7	6/11	−1.1	1.2	
80S Helsinki	†CW+AF+IMC	10/29	20/38	−0.6	5.9	
82B DBCG 82b ipremenop.	†CW+AF+IMC	26/83	36/79	−7.8	13.9	
82C DBCG 82c ipostmenop.	†CW+AF+IMC	33/53	45/75	0.5	17.8	
82Q ECOG EST3181	†CW+AF+IMC	24/34	16/36	7.1	8.8	
**(a) Subtotal**		**352/ 632 (55.7%)**	**407/ 682 (59.7%)**	**−18.7**	**166.0**	**0.89 (SE 0.07)** 2p > 0.1: NS
**(b) Axillary sampling**						
71B Stockholm A	CW+AF+IMC	32/43	35/42	−0.9	15.1	
77J MD Ander. 7730B	†CW+AF+IMC	4/4	3/4	0.0	0.5	
79F Coimbra	†CW+AF+IMC	15/28	18/29	−1.0	7.1	
82B DBCG 82b ipremenop.	†CW+AF+IMC	175/344	194/322	−23.2	85.2	
82C DBCG 82c ipostmenop.	†CW+AF+IMC	165/245	176/240	−14.5	77.9	
84A GBSG 03 Germany	†CW+AF+IMC	22/62	21/57	0.4	9.4	
**(b) Subtotal**		**413/ 726 (56.9%)**	**447/ 694 (64.4%)**	**−39.2**	**195.3**	**0.82 (SE 0.06)** 2p = 0.005
**(c) Extent of axillary surgery unknown**						
80W NSABC Israel	†CW+AF+IMC	12/20	13/19	−1.1	4.9	
86C CRC, UK	†Various	7/10	11/18	0.1	2.9	
**(c) Subtotal**		**19/ 30 (63.3%)**	**24/ 37 (64.9%)**	**−0.9**	**7.8**	**0.89 (SE 0.34)** 2p > 0.1: NS
**Total**		**784/ 1388 (56.5%)**	**878/ 1413 (62.1%)**	**−58.8**	**369.0**	**0.85 (SE 0.05)** 2p = 0.002

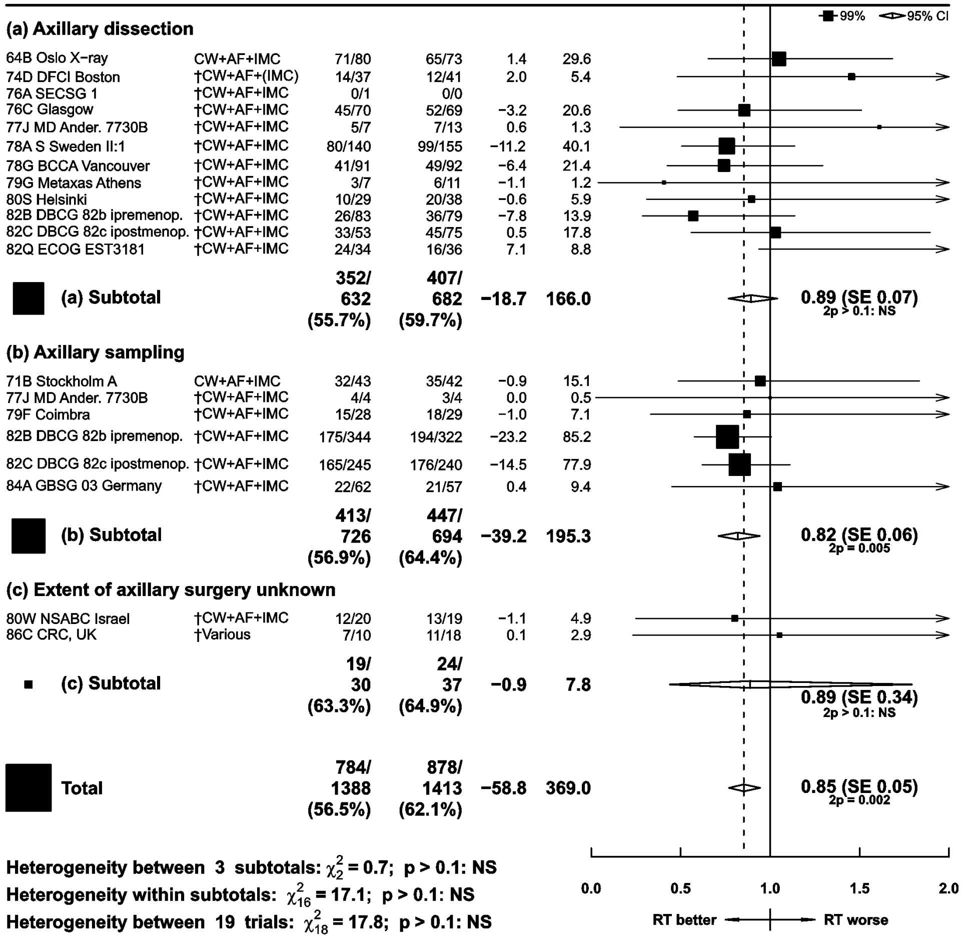

Heterogeneity between 3 subtotals: $\chi^2_2 = 0.7$; p > 0.1: NS
Heterogeneity within subtotals: $\chi^2_{16} = 17.1$; p > 0.1: NS
Heterogeneity between 19 trials: $\chi^2_{18} = 17.8$; p > 0.1: NS

† Same polychemotherapy (usually cyclophosphamide, methotrexate, and 5-fluorouracil), and/or tamoxifen in both groups.
Radiotherapy sites: CW=chest wall, AF=Axilla and/or supraclavicular fossa, IMC=Internal mammary chain. Site(s) in brackets were not always treated.

Webfigure 32. Effect of radiotherapy (RT) to the chest wall and regional lymph nodes versus not after mastectomy and axillary dissection (Mast+AD) or axillary sampling (Mast+AS): Event rate ratios, one line per trial, for locoregional recurrence and recurrence of any type during years 0-9 and for breast cancer and all-cause mortality in 2557 women with 4+ pathologically positive nodes (pN4+).

2557 pN4+ women

Locoregional recurrence first (years 0-9)

Year code, and study name	Treatment Information	Events/Women Allocated RT	Events/Women Allocated No RT	RT events Logrank O−E	RT events Variance of O−E	Ratio of annual event rates RT : No RT
**(a) Axillary dissection**						
64B Oslo X−ray	CW+AF+IMC	0/30	4/20	−2.2	0.9	
74D DFCI Boston	†CW+AF+(IMC)	5/55	14/56	−4.0	4.2	
74Q Piedmont OA (pN4+)	†CW+AF+IMC	6/65	9/55	−1.6	2.9	
76A SECSG 1	†CW+AF+IMC	12/125	18/129	−3.5	7.1	
76C Glasgow	†CW+AF+IMC	11/40	10/31	−0.8	4.6	
78A S Sweden II:1	†CW+AF+IMC	5/85	11/73	−4.2	3.7	
78G BCCA Vancouver	†CW+AF+IMC	8/60	17/54	−6.1	5.7	
78Q Düsseldorf U.	†CW+AF+IMC	0/34	1/54	−0.4	0.2	
79G Metaxas Athens	†CW+AF+IMC	4/18	3/25	0.5	1.7	
80S Helsinki	†CW+AF+IMC	3/16	2/9	−0.3	0.7	
82B DBCG 82b ipremenop.	†CW+AF+IMC	8/110	29/128	−10.8	8.4	
82C DBCG 82c ipostmenop.	†CW+AF+IMC	5/104	27/94	−12.3	7.4	
82Q ECOG EST3181	†CW+AF+IMC	11/127	27/121	−8.3	8.8	
**(a) Subtotal**		**78/ 869 (9.0%)**	**172/ 849 (20.3%)**	**−53.8**	**56.4**	**0.39 (SE 0.09)** 2p < 0.00001
**(b) Axillary sampling**						
79F Coimbra	†CW+AF+IMC	5/32	4/29	0.5	1.8	
82B DBCG 82b ipremenop.	†CW+AF+IMC	10/146	50/143	−22.4	13.6	
82C DBCG 82c ipostmenop.	†CW+AF+IMC	6/127	60/140	−28.8	15.0	
84A GBSG 03 Germany	†CW+AF+IMC	1/34	6/43	−1.9	1.7	
**(b) Subtotal**		**22/ 339 (6.5%)**	**120/ 355 (33.8%)**	**−52.6**	**32.1**	**0.19 (SE 0.09)** 2p < 0.00001
**(c) Extent of axillary surgery unknown**						
80W NSABC Israel	†CW+AF+IMC	2/34	3/39	−0.3	1.1	
86C CRC, UK	†Various	1/7	1/2	−0.7	0.2	
**(c) Subtotal**		**3/ 41 (7.3%)**	**4/ 41 (9.8%)**	**−0.9**	**1.3**	**0.49 (SE 0.62)** 2p > 0.1: NS
**Total**		**103/ 1249 (8.2%)**	**296/ 1245 (23.8%)**	**−107.4**	**89.8**	**0.30 (SE 0.06)** 2p < 0.00001

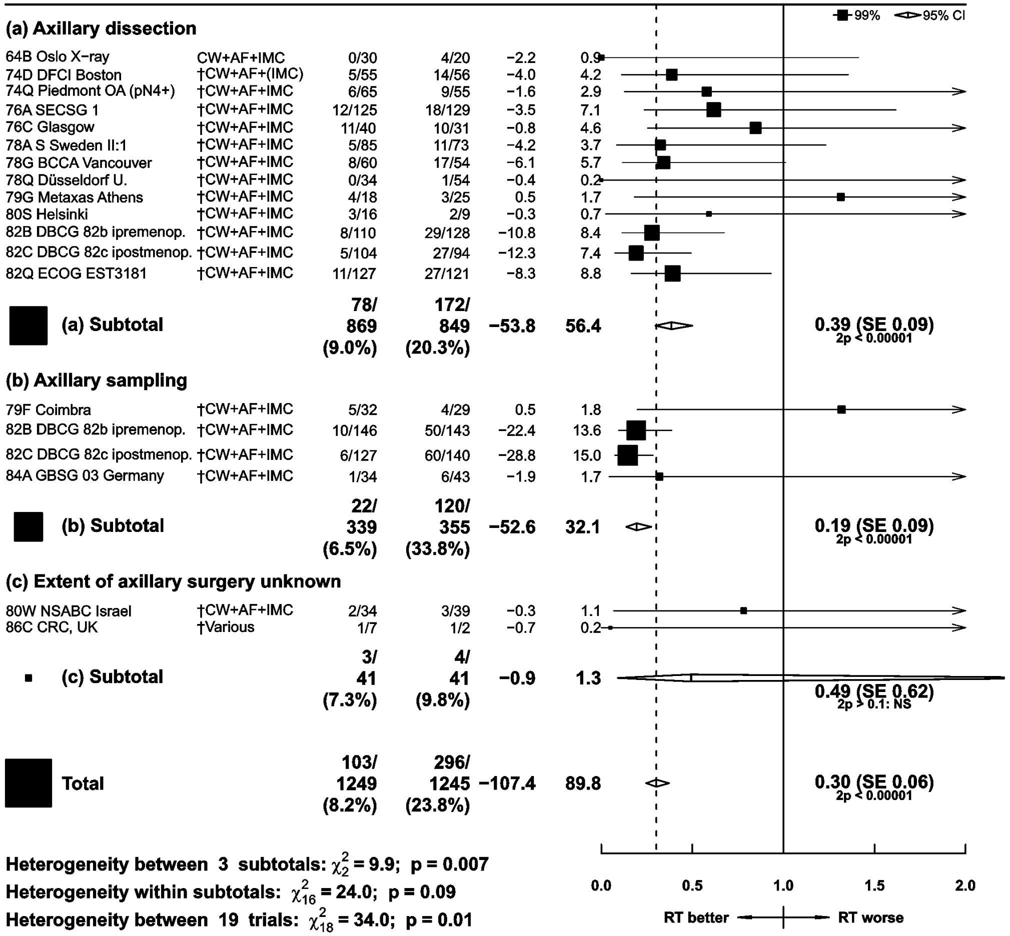

Heterogeneity between 3 subtotals: χ^2_2 = 9.9; p = 0.007
Heterogeneity within subtotals: χ^2_{16} = 24.0; p = 0.09
Heterogeneity between 19 trials: χ^2_{18} = 34.0; p = 0.01

Any first recurrence (years 0-9)

Year code, and study name	Treatment Information	Events/Women Allocated RT	Events/Women Allocated No RT	RT events Logrank O−E	RT events Variance of O−E	Ratio of annual event rates RT : No RT
**(a) Axillary dissection**						
64B Oslo X−ray	CW+AF+IMC	24/30	18/20	−6.0	5.2	
74D DFCI Boston	†CW+AF+(IMC)	36/55	42/56	−0.6	15.7	
74Q Piedmont OA (pN4+)	†CW+AF+IMC	41/65	40/55	−2.6	14.9	
76A SECSG 1	†CW+AF+IMC	62/125	83/129	−10.8	28.6	
76C Glasgow	†CW+AF+IMC	29/40	24/31	−1.4	9.5	
77J MD Ander. 7730B	†CW+AF+IMC	17/24	17/30	4.8	5.2	
78A S Sweden II:1	†CW+AF+IMC	56/85	54/73	−7.4	21.2	
78G BCCA Vancouver	†CW+AF+IMC	34/60	43/54	−8.5	15.0	
78Q Düsseldorf U.	†CW+AF+IMC	17/34	21/54	3.6	7.4	
79G Metaxas Athens	†CW+AF+IMC	11/18	16/25	−2.3	4.4	
80S Helsinki	†CW+AF+IMC	10/16	2/9	2.2	1.7	
82B DBCG 82b ipremenop.	†CW+AF+IMC	78/110	105/128	−15.0	34.8	
82C DBCG 82c ipostmenop.	†CW+AF+IMC	79/104	83/94	−9.4	30.7	
82Q ECOG EST3181	†CW+AF+IMC	81/127	76/121	−0.8	32.2	
**(a) Subtotal**		**575/ 893 (64.4%)**	**624/ 879 (71.0%)**	**−54.1**	**226.4**	**0.79 (SE 0.06)** 2p = 0.0003
**(b) Axillary sampling**						
77J MD Ander. 7730B	†CW+AF+IMC	1/3	2/6			
79F Coimbra	†CW+AF+IMC	18/32	20/29	−1.1	6.6	
82B DBCG 82b ipremenop.	†CW+AF+IMC	99/146	126/143	−28.3	40.3	
82C DBCG 82c ipostmenop.	†CW+AF+IMC	94/127	119/140	−16.8	39.8	
84A GBSG 03 Germany	†CW+AF+IMC	19/34	29/43	−2.4	9.7	
**(b) Subtotal**		**231/ 342 (67.5%)**	**296/ 361 (82.0%)**	**−48.6**	**96.4**	**0.60 (SE 0.08)** 2p < 0.00001
**(c) Extent of axillary surgery unknown**						
80W NSABC Israel	†CW+AF+IMC	10/34	7/39	1.5	3.9	
86C CRC, UK	†Various	6/7	2/2	−0.7	0.2	
**(c) Subtotal**		**16/ 41 (39.0%)**	**9/ 41 (22.0%)**	**0.8**	**4.1**	**1.21 (SE 0.54)** 2p > 0.1: NS
**Total**		**822/ 1276 (64.4%)**	**929/ 1281 (72.5%)**	**−101.9**	**326.9**	**0.73 (SE 0.05)** 2p < 0.00001

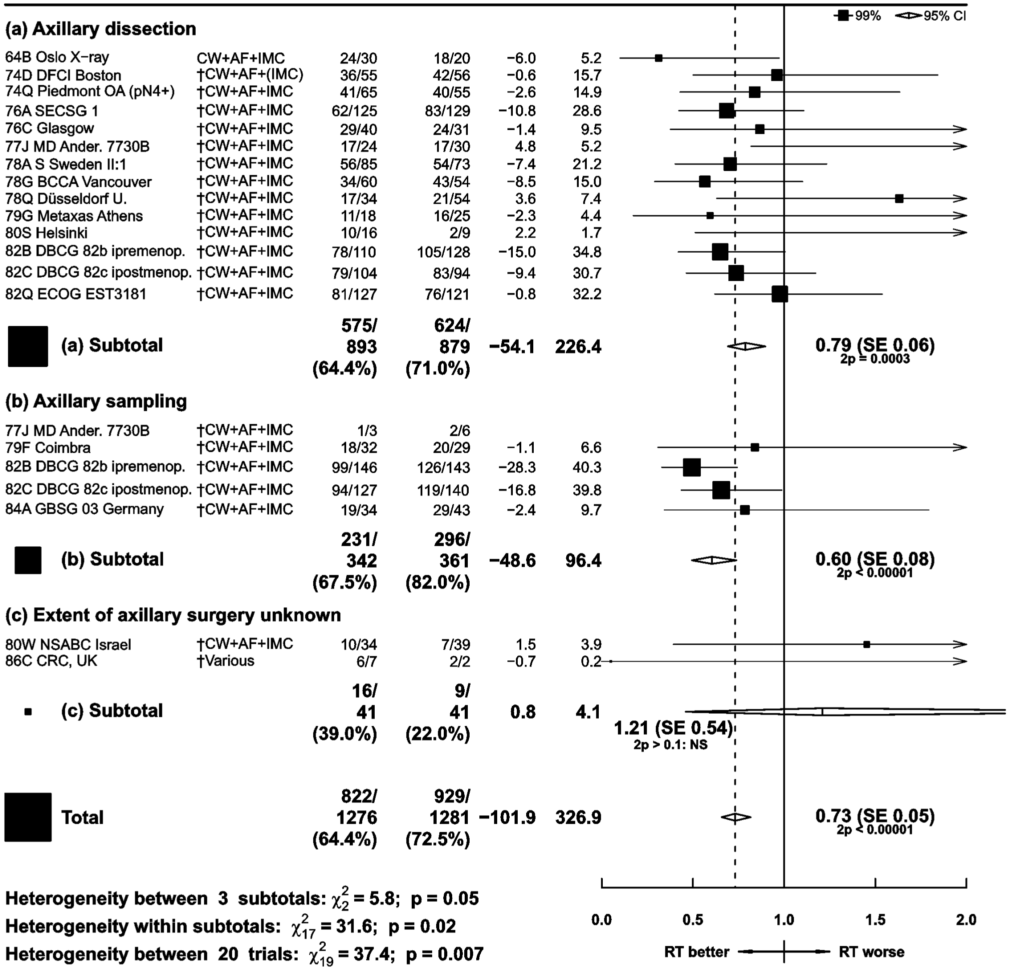

Heterogeneity between 3 subtotals: χ^2_2 = 5.8; p = 0.05
Heterogeneity within subtotals: χ^2_{17} = 31.6; p = 0.02
Heterogeneity between 20 trials: χ^2_{19} = 37.4; p = 0.007

† Same polychemotherapy (usually cyclophosphamide, methotrexate, and 5-fluorouracil), and/or tamoxifen in both groups.
Radiotherapy sites: CW=chest wall, AF=Axilla and/or supraclavicular fossa, IMC=Internal mammary chain. Site(s) in brackets were not always treated.

continued overleaf

2557 pN4+ women

Breast cancer mortality

Year code, and study name	Treatment Information	Deaths/Women Allocated RT	Deaths/Women Allocated No RT	RT deaths Logrank O−E	RT deaths Variance of O−E	Ratio of annual death rates RT : No RT
**(a) Axillary dissection**						
64B Oslo X-ray	CW+AF+IMC	27/30	18/20	−5.9	5.6	
74D DFCI Boston	†CW+AF+(IMC)	30/55	37/56	−0.2	14.6	
74Q Piedmont OA (pN4+)	†CW+AF+IMC	36/65	40/55	−3.5	14.3	
76A SECSG 1	†CW+AF+IMC	54/125	65/129	−3.7	24.7	
76C Glasgow	†CW+AF+IMC	30/40	27/31	−3.9	9.8	
77J MD Ander. 7730B	†CW+AF+IMC	18/24	17/30	5.4	5.7	
78A S Sweden II:1	†CW+AF+IMC	58/85	56/73	−4.6	23.9	
78G BCCA Vancouver	†CW+AF+IMC	37/60	46/54	−8.8	18.0	
78Q Düsseldorf U.	†CW+AF+IMC	14/34	14/54	4.9	5.1	
79G Metaxas Athens	†CW+AF+IMC	8/18	15/25	−2.4	4.7	
80S Helsinki	†CW+AF+IMC	11/16	2/9	2.8	2.1	
82B DBCG 82b ipremenop.	†CW+AF+IMC	79/110	107/128	−11.5	39.1	
82C DBCG 82c ipostmenop.	†CW+AF+IMC	81/104	81/94	−0.4	33.9	
82Q ECOG EST3181	†CW+AF+IMC	84/127	80/121	0.1	35.7	
**(a) Subtotal**		**567/ 893 (63.5%)**	**605/ 879 (68.8%)**	**−31.7**	**237.2**	**0.87 (SE 0.06)** 2p = 0.04
**(b) Axillary sampling**						
77J MD Ander. 7730B	†CW+AF+IMC	1/3	3/6			
79F Coimbra	†CW+AF+IMC	21/32	20/29	2.1	6.7	
82B DBCG 82b ipremenop.	†CW+AF+IMC	101/146	130/143	−24.8	46.4	
82C DBCG 82c ipostmenop.	†CW+AF+IMC	98/127	116/140	−4.1	44.7	
84A GBSG 03 Germany	†CW+AF+IMC	18/34	24/43	−0.3	8.5	
**(b) Subtotal**		**239/ 342 (69.9%)**	**293/ 361 (81.2%)**	**−27.0**	**106.3**	**0.78 (SE 0.09)** 2p = 0.009
**(c) Extent of axillary surgery unknown**						
80W NSABC Israel	†CW+AF+IMC	9/34	7/39	1.7	3.5	
86C CRC, UK	†Various	5/7	2/2	−0.2	0.5	
**(c) Subtotal**		**14/ 41 (34.1%)**	**9/ 41 (22.0%)**	**1.5**	**4.0**	**1.47 (SE 0.61)** 2p > 0.1: NS
**Total**		**820/ 1276 (64.3%)**	**907/ 1281 (70.8%)**	**−57.2**	**347.5**	**0.85 (SE 0.05)** 2p = 0.002

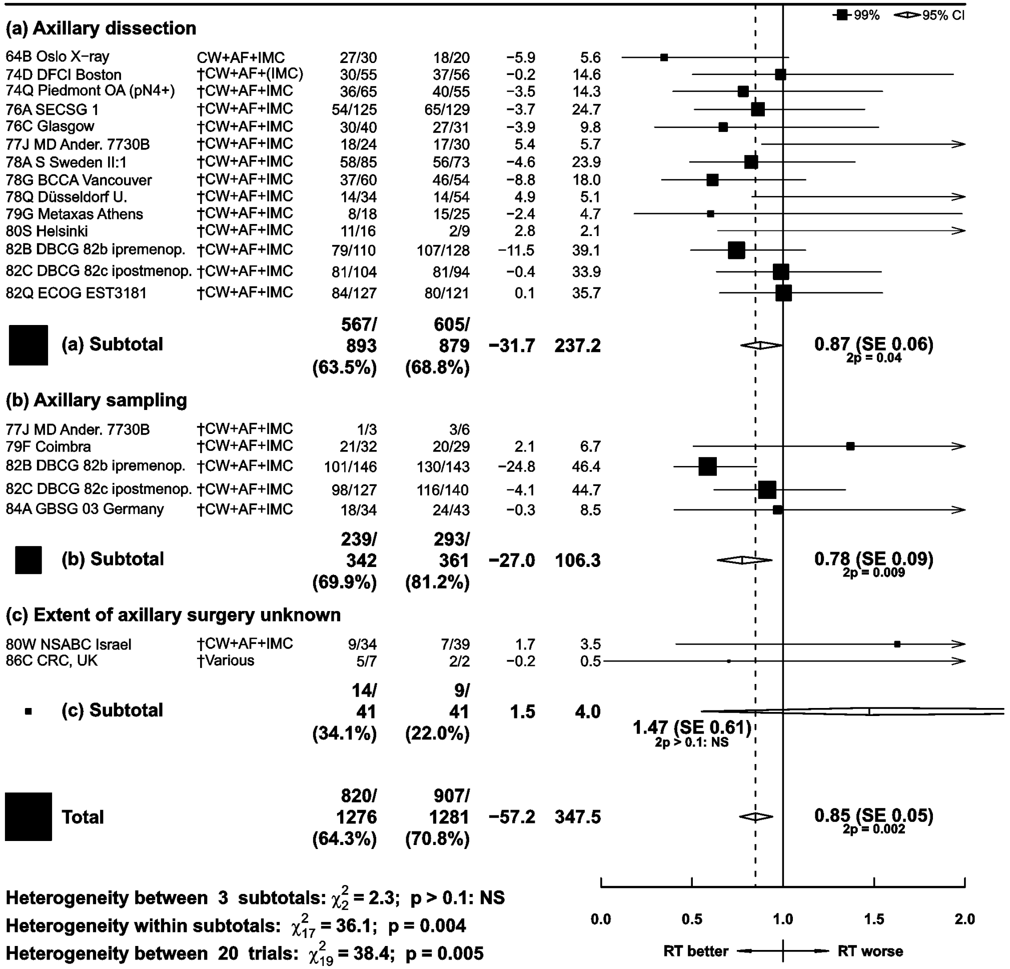

Heterogeneity between 3 subtotals: $\chi^2_2 = 2.3$; p > 0.1: NS
Heterogeneity within subtotals: $\chi^2_{17} = 36.1$; p = 0.004
Heterogeneity between 20 trials: $\chi^2_{19} = 38.4$; p = 0.005

Any death

Year code, and study name	Treatment Information	Deaths/Women Allocated RT	Deaths/Women Allocated No RT	RT deaths Logrank O−E	RT deaths Variance of O−E	Ratio of annual death rates RT : No RT
**(a) Axillary dissection**						
64B Oslo X-ray	CW+AF+IMC	30/30	20/20	−6.6	6.3	
74D DFCI Boston	†CW+AF+(IMC)	35/55	39/56	0.9	16.0	
74Q Piedmont OA (pN4+)	†CW+AF+IMC	41/65	41/55	−1.6	15.2	
76A SECSG 1	†CW+AF+IMC	60/125	69/129	−3.2	26.9	
76C Glasgow	†CW+AF+IMC	32/40	29/31	−4.2	10.8	
77J MD Ander. 7730B	†CW+AF+IMC	19/24	17/30	5.9	5.9	
78A S Sweden II:1	†CW+AF+IMC	69/85	62/73	−5.0	27.4	
78G BCCA Vancouver	†CW+AF+IMC	40/60	46/54	−7.9	18.6	
78Q Düsseldorf U.	†CW+AF+IMC	17/34	24/54	3.3	7.8	
79G Metaxas Athens	†CW+AF+IMC	8/18	15/25	−2.4	4.7	
80S Helsinki	†CW+AF+IMC	12/16	3/9	3.0	2.6	
82B DBCG 82b ipremenop.	†CW+AF+IMC	85/110	108/128	−9.2	40.8	
82C DBCG 82c ipostmenop.	†CW+AF+IMC	89/104	86/94	−1.6	36.3	
82Q ECOG EST3181	†CW+AF+IMC	94/127	96/121	−2.9	41.3	
**(a) Subtotal**		**631/ 893 (70.7%)**	**655/ 879 (74.5%)**	**−31.5**	**260.5**	**0.89 (SE 0.06)** 2p = 0.05
**(b) Axillary sampling**						
77J MD Ander. 7730B	†CW+AF+IMC	1/3	3/6			
79F Coimbra	†CW+AF+IMC	24/32	21/29	3.2	7.5	
82B DBCG 82b ipremenop.	†CW+AF+IMC	109/146	132/143	−23.2	48.7	
82C DBCG 82c ipostmenop.	†CW+AF+IMC	107/127	131/140	−10.2	49.3	
84A GBSG 03 Germany	†CW+AF+IMC	23/34	27/43	0.9	10.5	
**(b) Subtotal**		**264/ 342 (77.2%)**	**314/ 361 (87.0%)**	**−29.2**	**115.9**	**0.78 (SE 0.08)** 2p = 0.007
**(c) Extent of axillary surgery unknown**						
80W NSABC Israel	†CW+AF+IMC	11/34	7/39	2.7	4.0	
86C CRC, UK	†Various	6/7	2/2	−0.2	0.5	
**(c) Subtotal**		**17/ 41 (41.5%)**	**9/ 41 (22.0%)**	**2.5**	**4.5**	**1.75 (SE 0.63)** 2p > 0.1: NS
**Total**		**912/ 1276 (71.5%)**	**978/ 1281 (76.3%)**	**−58.2**	**380.9**	**0.86 (SE 0.05)** 2p = 0.003

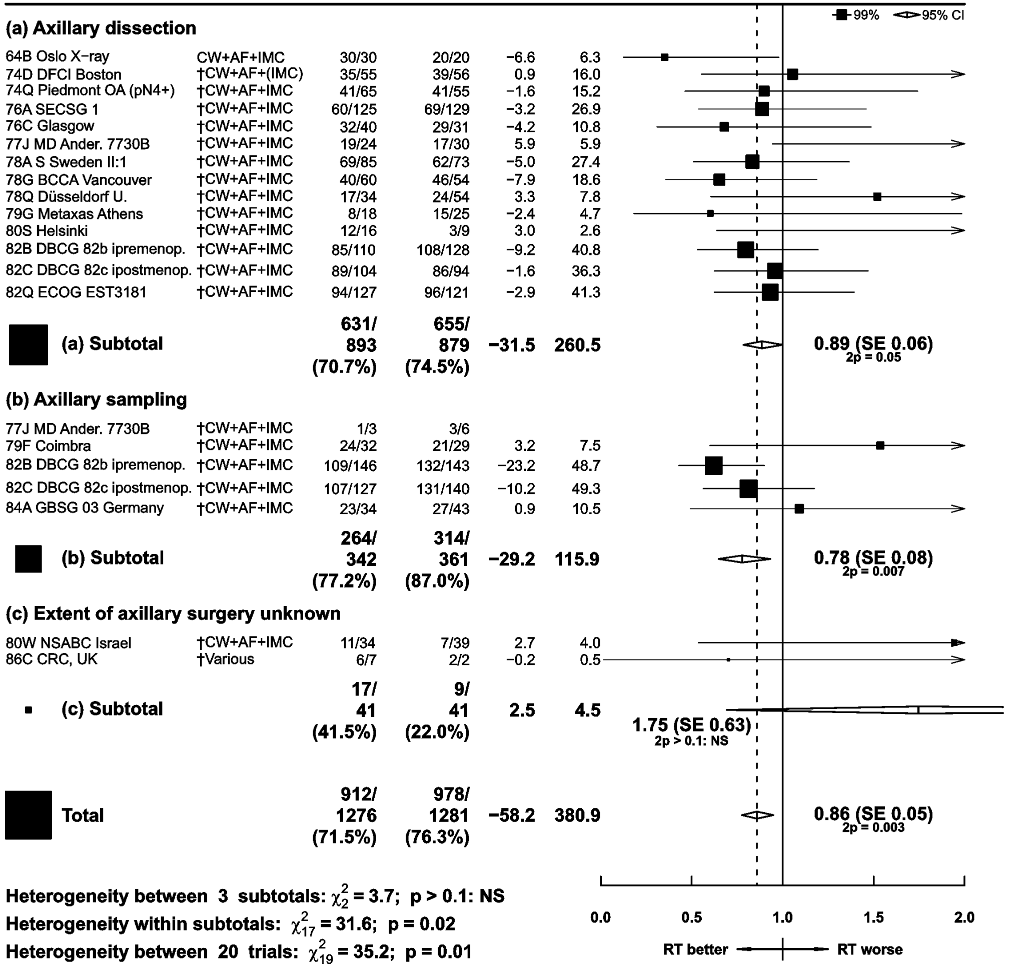

Heterogeneity between 3 subtotals: $\chi^2_2 = 3.7$; p > 0.1: NS
Heterogeneity within subtotals: $\chi^2_{17} = 31.6$; p = 0.02
Heterogeneity between 20 trials: $\chi^2_{19} = 35.2$; p = 0.01

† Same polychemotherapy (usually cyclophosphamide, methotrexate, and 5-fluorouracil), and/or tamoxifen in both groups.
Radiotherapy sites: CW=chest wall, AF=Axilla and/or supraclavicular fossa, IMC=Internal mammary chain. Site(s) in brackets were not always treated.

continued overleaf

Webfigure 33. Effect of radiotherapy (RT) to the chest wall and regional lymph nodes versus not after mastectomy and axillary dissection (Mast+AD) or axillary sampling (Mast+AS): Event rate ratios, one line per trial, for locoregional recurrence and recurrence of any type during years 0-9 and for breast cancer and all-cause mortality in 463 women with pathologically positive nodes (pN?+) but unknown if they were 1-3 or 4+ positive.

463 pN?+ women

Locoregional recurrence first (years 0-9)

Year code, and study name	Treatment Information	Events/Women: Allocated RT	Events/Women: Allocated No RT	RT events: Logrank O−E	RT events: Variance of O−E	Ratio of annual event rates RT : No RT
**(a) Axillary dissection**						
74D DFCI Boston	†CW+AF+(IMC)	1/11	2/8	−0.3	0.5	
78G BCCA Vancouver	†CW+AF+IMC	1/12	3/7	−1.9	0.7	
80S Helsinki	†CW+AF+IMC	0/2	1/5			
**(a) Subtotal**		**2/ 25 (8.0%)**	**6/ 20 (30.0%)**	**−2.2**	**1.1**	**0.15 (SE 0.42)** 2p = 0.04
**(b) Axillary sampling**						
71B Stockholm A	CW+AF+IMC	6/75	32/79	−15.0	8.6	
73A Southampton UK	CW+AF+IMC	5/33	21/38	−8.7	5.3	
74B Edinburgh I	CW+AF	0/0	0/1			
79F Coimbra	†CW+AF+IMC	1/2	1/3	0.5	0.2	
82B DBCG 82b ipremenop.	†CW+AF+IMC	2/27	8/28	−3.3	2.3	
82C DBCG 82c ipostmenop.	†CW+AF+IMC	2/29	4/26	−0.7	1.2	
85F Nottingham	†CW+AF	6/36	20/41	−6.9	5.7	
**(b) Subtotal**		**22/ 202 (10.9%)**	**86/ 216 (39.8%)**	**−34.0**	**23.4**	**0.23 (SE 0.11)** 2p < 0.00001
**Total**		**24/ 227 (10.6%)**	**92/ 236 (39.0%)**	**−36.2**	**24.5**	**0.23 (SE 0.11)** 2p < 0.00001

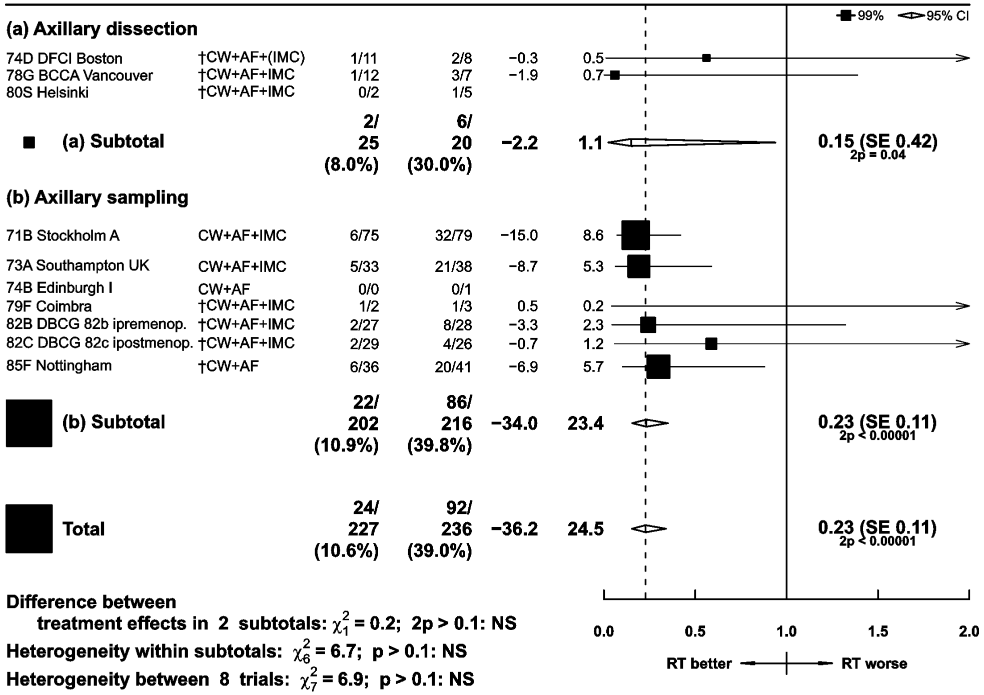

Difference between treatment effects in 2 subtotals: $\chi^2_1 = 0.2$; 2p > 0.1: NS
Heterogeneity within subtotals: $\chi^2_6 = 6.7$; p > 0.1: NS
Heterogeneity between 8 trials: $\chi^2_7 = 6.9$; p > 0.1: NS

Any first recurrence (years 0-9)

Year code, and study name	Treatment Information	Events/Women: Allocated RT	Events/Women: Allocated No RT	RT events: Logrank O−E	RT events: Variance of O−E	Ratio of annual event rates RT : No RT
**(a) Axillary dissection**						
74D DFCI Boston	†CW+AF+(IMC)	7/11	5/8	0.1	2.0	
78G BCCA Vancouver	†CW+AF+IMC	7/12	4/7	−1.4	1.6	
80S Helsinki	†CW+AF+IMC	2/2	3/5	−0.8	0.5	
**(a) Subtotal**		**16/ 25 (64.0%)**	**12/ 20 (60.0%)**	**−2.1**	**4.1**	**0.61 (SE 0.39)** 2p > 0.1: NS
**(b) Axillary sampling**						
71B Stockholm A	CW+AF+IMC	39/75	59/79	−15.6	18.7	
73A Southampton UK	CW+AF+IMC	15/33	34/38	−12.3	8.3	
74B Edinburgh I	CW+AF	0/0	0/1			
79F Coimbra	†CW+AF+IMC	2/2	3/3			
82B DBCG 82b ipremenop.	†CW+AF+IMC	3/27	10/28	−4.0	3.0	
82C DBCG 82c ipostmenop.	†CW+AF+IMC	6/29	4/26	1.1	2.2	
85F Nottingham	†CW+AF	22/36	34/41	−6.5	10.6	
**(b) Subtotal**		**87/ 202 (43.1%)**	**144/ 216 (66.7%)**	**−37.2**	**42.8**	**0.42 (SE 0.10)** 2p < 0.00001
**Total**		**103/ 227 (45.4%)**	**156/ 236 (66.1%)**	**−39.2**	**46.9**	**0.43 (SE 0.10)** 2p < 0.00001

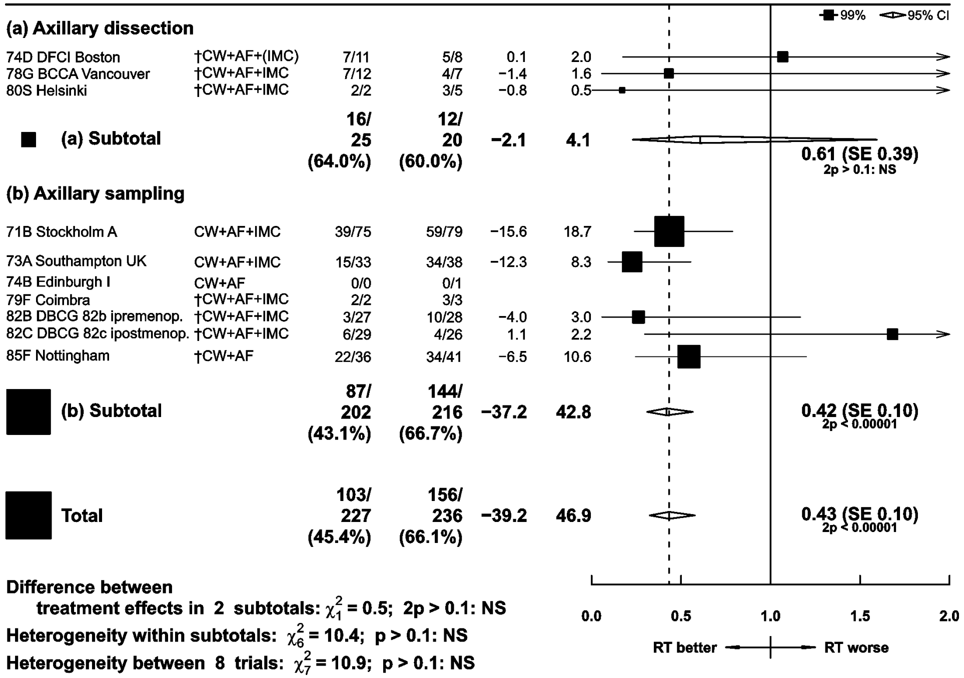

Difference between treatment effects in 2 subtotals: $\chi^2_1 = 0.5$; 2p > 0.1: NS
Heterogeneity within subtotals: $\chi^2_6 = 10.4$; p > 0.1: NS
Heterogeneity between 8 trials: $\chi^2_7 = 10.9$; p > 0.1: NS

† Same polychemotherapy (usually cyclophosphamide, methotrexate, and 5-fluorouracil), and/or tamoxifen in both groups.
Radiotherapy sites: CW=chest wall, AF=Axilla and/or supraclavicular fossa, IMC=Internal mammary chain. Site(s) in brackets were not always treated.

continued overleaf

463 pN?+ women

Breast cancer mortality

Year code, and study name	Treatment Information	Deaths/Women Allocated RT	Deaths/Women Allocated No RT	RT deaths Logrank O−E	RT deaths Variance of O−E	Ratio of annual death rates RT : No RT
**(a) Axillary dissection**						
74D DFCI Boston	†CW+AF+(IMC)	7/11	4/8	0.2	1.3	
78G BCCA Vancouver	†CW+AF+IMC	8/12	5/7	−1.2	2.1	
80S Helsinki	†CW+AF+IMC	2/2	2/5	−0.8	0.5	
**(a) Subtotal**		**17/ 25 (68.0%)**	**11/ 20 (55.0%)**	**−1.9**	**3.9**	**0.61 (SE 0.40)** 2p > 0.1: NS
**(b) Axillary sampling**						
71B Stockholm A	CW+AF+IMC	44/75	64/79	−11.1	24.0	
73A Southampton UK	CW+AF+IMC	23/33	32/38	−5.0	10.4	
74B Edinburgh I	CW+AF	0/0	0/1			
79F Coimbra	†CW+AF+IMC	2/2	3/3	−0.5	0.2	
82B DBCG 82b ipremenop.	†CW+AF+IMC	0/27	2/28	−0.9	0.5	
82C DBCG 82c ipostmenop.	†CW+AF+IMC	0/29	0/26			
85F Nottingham	†CW+AF	22/36	28/41	−2.9	10.3	
**(b) Subtotal**		**91/ 202 (45.0%)**	**129/ 216 (59.7%)**	**−20.5**	**45.5**	**0.64 (SE 0.12)** 2p = 0.002
**Total**		**108/ 227 (47.6%)**	**140/ 236 (59.3%)**	**−22.4**	**49.4**	**0.64 (SE 0.11)** 2p = 0.001

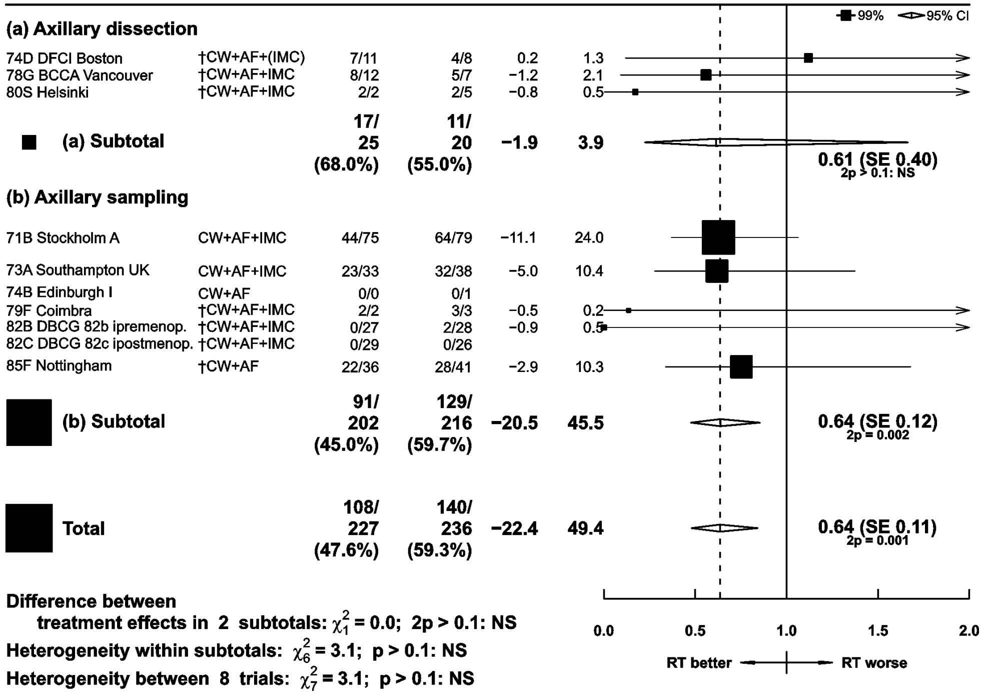

Difference between treatment effects in 2 subtotals: $\chi^2_1 = 0.0$; 2p > 0.1: NS
Heterogeneity within subtotals: $\chi^2_6 = 3.1$; p > 0.1: NS
Heterogeneity between 8 trials: $\chi^2_7 = 3.1$; p > 0.1: NS

Any death

Year code, and study name	Treatment Information	Deaths/Women Allocated RT	Deaths/Women Allocated No RT	RT deaths Logrank O−E	RT deaths Variance of O−E	Ratio of annual death rates RT : No RT
**(a) Axillary dissection**						
74D DFCI Boston	†CW+AF+(IMC)	8/11	4/8	0.2	1.3	
78G BCCA Vancouver	†CW+AF+IMC	8/12	5/7	−1.2	2.1	
80S Helsinki	†CW+AF+IMC	2/2	2/5	−0.8	0.5	
**(a) Subtotal**		**18/ 25 (72.0%)**	**11/ 20 (55.0%)**	**−1.9**	**3.9**	**0.61 (SE 0.40)** 2p > 0.1: NS
**(b) Axillary sampling**						
71B Stockholm A	CW+AF+IMC	61/75	69/79	−10.3	27.5	
73A Southampton UK	CW+AF+IMC	28/33	32/38	−3.5	11.1	
74B Edinburgh I	CW+AF	0/0	1/1			
79F Coimbra	†CW+AF+IMC	2/2	3/3	−0.5	0.2	
82B DBCG 82b ipremenop.	†CW+AF+IMC	0/27	2/28	−0.9	0.5	
82C DBCG 82c ipostmenop.	†CW+AF+IMC	0/29	0/26			
85F Nottingham	†CW+AF	23/36	30/41	−3.6	11.0	
**(b) Subtotal**		**114/ 202 (56.4%)**	**137/ 216 (63.4%)**	**−18.8**	**50.5**	**0.69 (SE 0.12)** 2p = 0.008
**Total**		**132/ 227 (58.1%)**	**148/ 236 (62.7%)**	**−20.6**	**54.3**	**0.68 (SE 0.11)** 2p = 0.005

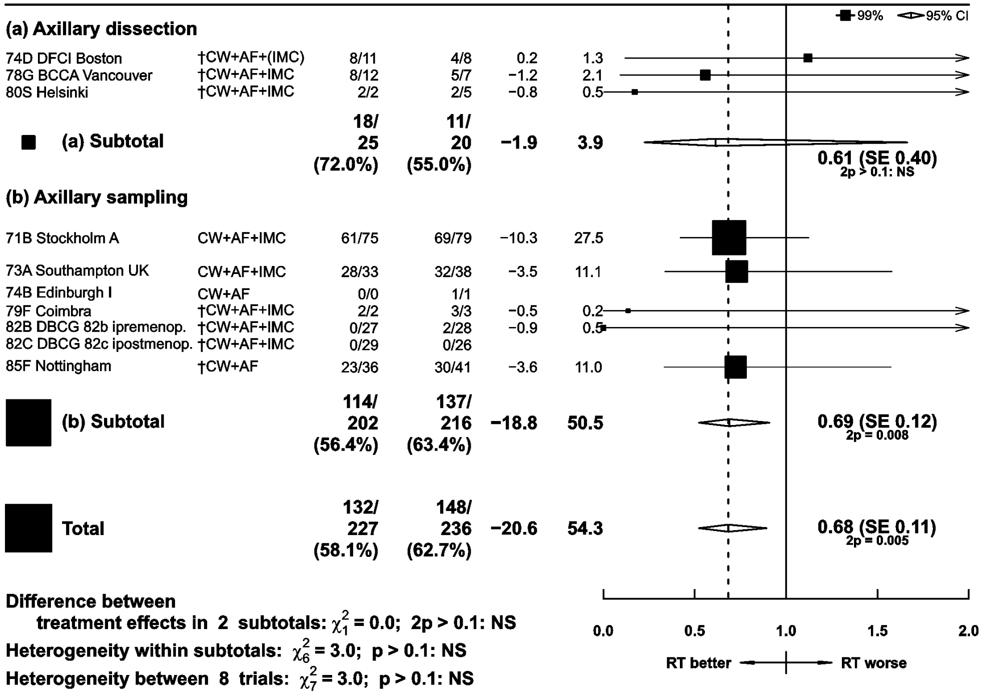

Difference between treatment effects in 2 subtotals: $\chi^2_1 = 0.0$; 2p > 0.1: NS
Heterogeneity within subtotals: $\chi^2_6 = 3.0$; p > 0.1: NS
Heterogeneity between 8 trials: $\chi^2_7 = 3.0$; p > 0.1: NS

† Same polychemotherapy (usually cyclophosphamide, methotrexate, and 5-fluorouracil), and/or tamoxifen in both groups.
Radiotherapy sites: CW=chest wall, AF=Axilla and/or supraclavicular fossa, IMC=Internal mammary chain. Site(s) in brackets were not always treated.

Webfigure 34. Effect of radiotherapy (RT) to the chest wall and regional lymph nodes versus not after mastectomy and axillary dissection (Mast+AD) or axillary sampling (Mast+AS): Event rate ratios, one line per trial, for locoregional recurrence and recurrence of any type during years 0-9 and for breast cancer and all-cause mortality in 720 women with unknown pathological nodal status (pN?).

720 pN? women

Locoregional recurrence first (years 0-9)

Year code, and study name	Treatment Information	Events/Women Allocated RT	Events/Women Allocated No RT	RT events Logrank O−E	RT events Variance of O−E	Ratio of annual event rates RT : No RT
**(a) Axillary dissection**						
76A SECSG 1	†CW+AF+IMC	0/1	0/1			
76C Glasgow	†CW+AF+IMC	1/1	0/6	0.7	0.2	
78A S Sweden II:1	†CW+AF+IMC	0/22	2/18	−0.7	0.5	
78G BCCA Vancouver	†CW+AF+IMC	0/1	0/1			
82Q ECOG EST3181	†CW+AF+IMC	0/1	0/0			
**(a) Subtotal**		**1/ 26 (3.8%)**	**2/ 26 (7.7%)**	**−0.1**	**0.7**	**0.93 (SE 1.17)** 2p > 0.1: NS
**(b) Axillary sampling**						
71B Stockholm A	CW+AF+IMC	1/2	2/4	−0.5	0.2	
73A Southampton UK	CW+AF+IMC	3/18	3/10	−1.1	1.1	
74B Edinburgh I	CW+AF	6/59	25/60	−9.3	7.0	
79F Coimbra	†CW+AF+IMC	0/0	0/1			
82B DBCG 82b ipremenop.	†CW+AF+IMC	12/137	46/150	−17.1	13.1	
82C DBCG 82c ipostmenop.	†CW+AF+IMC	5/113	29/97	−13.9	8.0	
84A GBSG 03 Germany	†CW+AF+IMC	0/2	0/1			
**(b) Subtotal**		**27/ 331 (8.2%)**	**105/ 323 (32.5%)**	**−41.9**	**29.4**	**0.24 (SE 0.10)** 2p < 0.00001
**(c) Extent of axillary surgery unknown**						
86C CRC, UK	†Various	0/6	0/4			
**(c) Subtotal**		**0/ 6 (0.0%)**	**0/ 4 (0.0%)**			
**Total**		**28/ 363 (7.7%)**	**107/ 353 (30.3%)**	**−42.0**	**30.1**	**0.25 (SE 0.10)** 2p < 0.00001

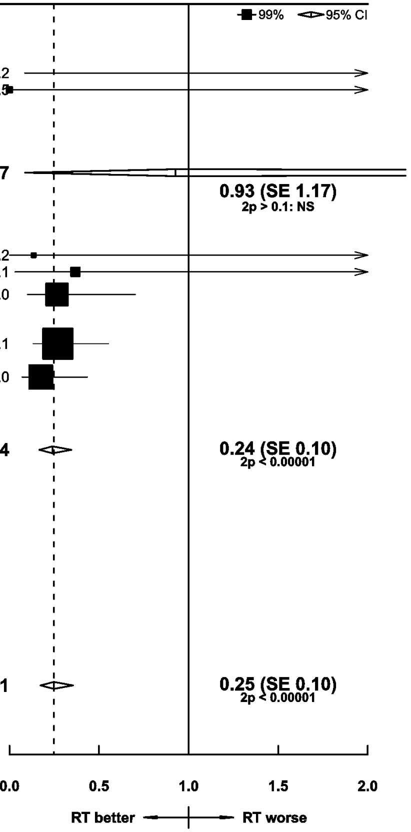

Difference between treatment effects in 2 subtotals: $\chi^2_1 = 1.2$; $2p > 0.1$: NS
Heterogeneity within subtotals: $\chi^2_5 = 4.5$; $p > 0.1$: NS
Heterogeneity between 7 trials: $\chi^2_6 = 5.7$; $p > 0.1$: NS

Any first recurrence (years 0-9)

Year code, and study name	Treatment Information	Events/Women Allocated RT	Events/Women Allocated No RT	RT events Logrank O−E	RT events Variance of O−E	Ratio of annual event rates RT : No RT
**(a) Axillary dissection**						
76A SECSG 1	†CW+AF+IMC	0/1	0/1			
76C Glasgow	†CW+AF+IMC	1/1	4/6	0.1	0.4	
77J MD Ander. 7730B	†CW+AF+IMC	3/4	0/0			
78A S Sweden II:1	†CW+AF+IMC	7/22	5/18	1.0	2.8	
78G BCCA Vancouver	†CW+AF+IMC	0/1	0/1			
82Q ECOG EST3181	†CW+AF+IMC	0/1	0/0			
**(a) Subtotal**		**11/ 30 (36.7%)**	**9/ 26 (34.6%)**	**1.1**	**3.2**	**1.41 (SE 0.67)** 2p > 0.1: NS
**(b) Axillary sampling**						
71B Stockholm A	CW+AF+IMC	1/2	3/4	−0.5	0.2	
73A Southampton UK	CW+AF+IMC	8/18	4/10	−0.9	1.6	
74B Edinburgh I	CW+AF	24/59	33/60	−5.8	11.3	
79F Coimbra	†CW+AF+IMC	0/0	1/1			
82B DBCG 82b ipremenop.	†CW+AF+IMC	69/137	87/150	−9.0	32.8	
82C DBCG 82c ipostmenop.	†CW+AF+IMC	61/113	62/97	−10.9	25.7	
84A GBSG 03 Germany	†CW+AF+IMC	1/2	0/1			
**(b) Subtotal**		**164/ 331 (49.5%)**	**190/ 323 (58.8%)**	**−27.1**	**71.6**	**0.68 (SE 0.10)** 2p = 0.001
**(c) Extent of axillary surgery unknown**						
86C CRC, UK	†Various	2/6	1/4	0.1	0.7	
**(c) Subtotal**		**2/ 6 (33.3%)**	**1/ 4 (25.0%)**	**0.1**	**0.7**	**1.19 (SE 1.33)** 2p > 0.1: NS
**Total**		**177/ 367 (48.2%)**	**200/ 353 (56.7%)**	**−25.9**	**75.5**	**0.71 (SE 0.10)** 2p = 0.003

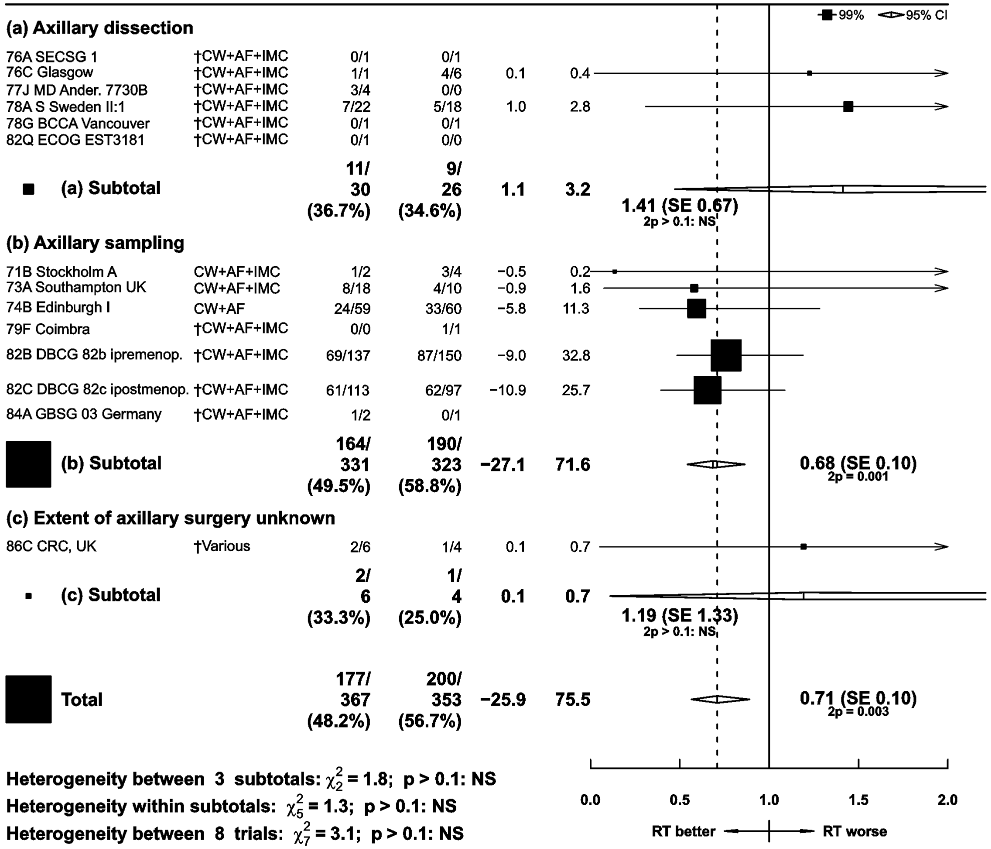

Heterogeneity between 3 subtotals: $\chi^2_2 = 1.8$; $p > 0.1$: NS
Heterogeneity within subtotals: $\chi^2_5 = 1.3$; $p > 0.1$: NS
Heterogeneity between 8 trials: $\chi^2_7 = 3.1$; $p > 0.1$: NS

† Same polychemotherapy (usually cyclophosphamide, methotrexate, and 5-fluorouracil), and/or tamoxifen in both groups.
Radiotherapy sites: CW=chest wall, AF=Axilla and/or supraclavicular fossa, IMC=Internal mammary chain. Site(s) in brackets were not always treated.

continued overleaf

720 pN? women

Breast cancer mortality

Year code, and study name	Treatment Information	Deaths/Women Allocated RT	Deaths/Women Allocated No RT	RT deaths Logrank O−E	RT deaths Variance of O−E
**(a) Axillary dissection**					
76A SECSG 1	†CW+AF+IMC	0/1	0/1		
76C Glasgow	†CW+AF+IMC	0/1	4/6	−0.6	0.4
77J MD Ander. 7730B	†CW+AF+IMC	3/4	0/0		
78A S Sweden II:1	†CW+AF+IMC	11/22	6/18	3.3	3.6
78G BCCA Vancouver	†CW+AF+IMC	0/1	1/1	−0.5	0.2
82Q ECOG EST3181	†CW+AF+IMC	0/1	0/0		
**(a) Subtotal**		**14/ 30 (46.7%)**	**11/ 26 (42.3%)**	**2.2**	**4.3**
**(b) Axillary sampling**					
71B Stockholm A	CW+AF+IMC	1/2	4/4	−0.5	0.2
73A Southampton UK	CW+AF+IMC	10/18	4/10	−0.7	1.7
74B Edinburgh I	CW+AF	33/59	38/60	0.0	15.6
79F Coimbra	†CW+AF+IMC	0/0	1/1		
82B DBCG 82b ipremenop.	†CW+AF+IMC	67/137	83/150	−4.4	32.5
82C DBCG 82c ipostmenop.	†CW+AF+IMC	63/113	64/97	−4.1	27.7
84A GBSG 03 Germany	†CW+AF+IMC	1/2	0/1		
**(b) Subtotal**		**175/ 331 (52.9%)**	**194/ 323 (60.1%)**	**−9.8**	**77.8**
**(c) Extent of axillary surgery unknown**					
86C CRC, UK	†Various	2/6	3/4	−0.2	0.9
**(c) Subtotal**		**2/ 6 (33.3%)**	**3/ 4 (75.0%)**	**−0.2**	**0.9**
**Total**		**191/ 367 (52.0%)**	**208/ 353 (58.9%)**	**−7.8**	**82.9**

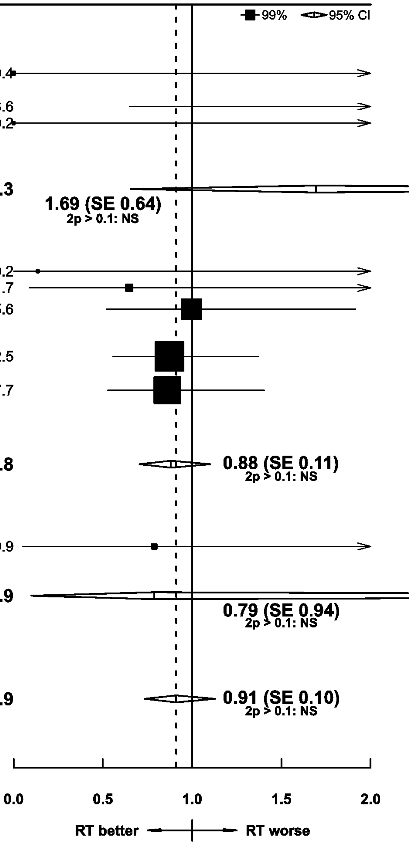

Heterogeneity between 3 subtotals: $\chi^2_2 = 1.7$; p > 0.1: NS
Heterogeneity within subtotals: $\chi^2_6 = 5.0$; p > 0.1: NS
Heterogeneity between 9 trials: $\chi^2_8 = 6.8$; p > 0.1: NS

Any death

Year code, and study name	Treatment Information	Deaths/Women Allocated RT	Deaths/Women Allocated No RT	RT deaths Logrank O−E	RT deaths Variance of O−E
**(a) Axillary dissection**					
76A SECSG 1	†CW+AF+IMC	0/1	0/1		
76C Glasgow	†CW+AF+IMC	0/1	5/6	−0.6	0.4
77J MD Ander. 7730B	†CW+AF+IMC	3/4	0/0		
78A S Sweden II:1	†CW+AF+IMC	19/22	11/18	4.9	6.7
78G BCCA Vancouver	†CW+AF+IMC	0/1	1/1	−0.5	0.2
82Q ECOG EST3181	†CW+AF+IMC	0/1	0/0		
**(a) Subtotal**		**22/ 30 (73.3%)**	**17/ 26 (65.4%)**	**3.9**	**7.3**
**(b) Axillary sampling**					
71B Stockholm A	CW+AF+IMC	2/2	4/4	−0.5	0.2
73A Southampton UK	CW+AF+IMC	15/18	7/10	−0.8	2.6
74B Edinburgh I	CW+AF	52/59	49/60	−0.6	20.9
79F Coimbra	†CW+AF+IMC	0/0	1/1		
82B DBCG 82b ipremenop.	†CW+AF+IMC	76/137	95/150	−5.8	37.5
82C DBCG 82c ipostmenop.	†CW+AF+IMC	80/113	78/97	−6.4	34.1
84A GBSG 03 Germany	†CW+AF+IMC	1/2	0/1		
**(b) Subtotal**		**226/ 331 (68.3%)**	**234/ 323 (72.4%)**	**−14.1**	**95.4**
**(c) Extent of axillary surgery unknown**					
86C CRC, UK	†Various	3/6	3/4	0.2	1.1
**(c) Subtotal**		**3/ 6 (50.0%)**	**3/ 4 (75.0%)**	**0.2**	**1.1**
**Total**		**251/ 367 (68.4%)**	**254/ 353 (72.0%)**	**−10.0**	**103.8**

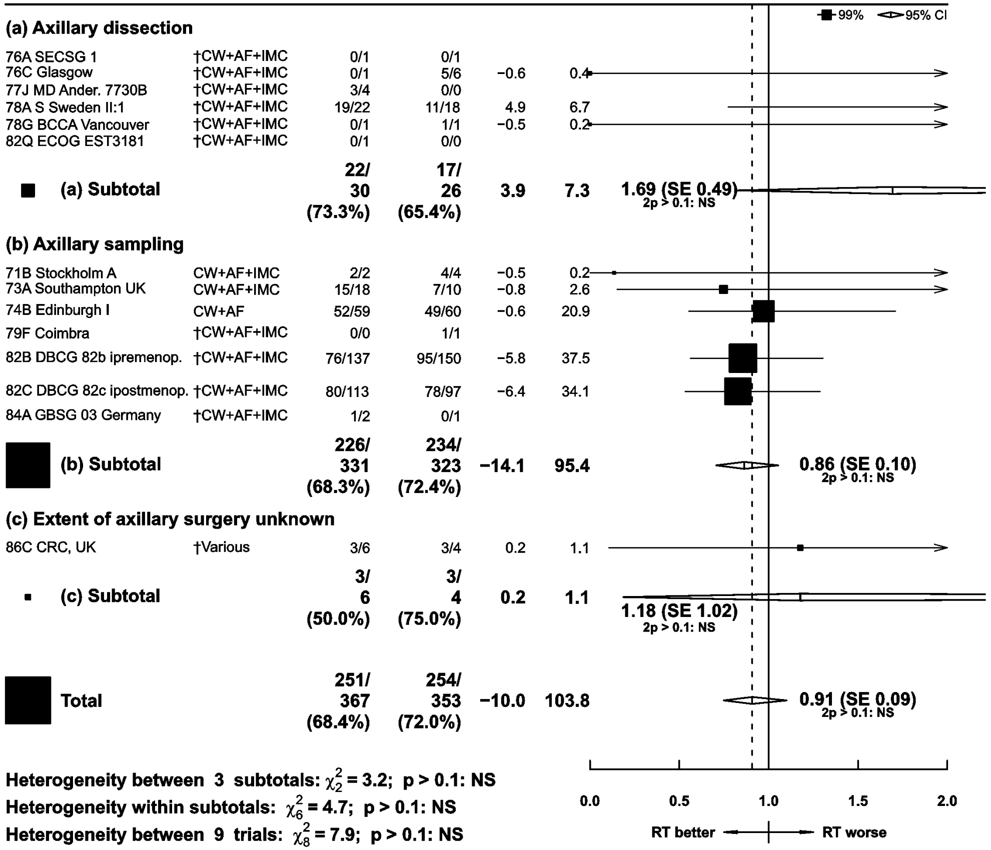

Heterogeneity between 3 subtotals: $\chi^2_2 = 3.2$; p > 0.1: NS
Heterogeneity within subtotals: $\chi^2_6 = 4.7$; p > 0.1: NS
Heterogeneity between 9 trials: $\chi^2_8 = 7.9$; p > 0.1: NS

† Same polychemotherapy (usually cyclophosphamide, methotrexate, and 5-fluorouracil), and/or tamoxifen in both groups.
Radiotherapy sites: CW=chest wall, AF=Axilla and/or supraclavicular fossa, IMC=Internal mammary chain. Site(s) in brackets were not always treated.

Webtable 2. **Availability of data from randomised trials beginning before the year 2000 and comparing radiotherapy to the regional lymph nodes alone versus not after mastectomy and axillary dissection (Mast+AD) or axillary sampling (Mast+AS)*.**

Nodal status†	Women	Deaths	Woman-years since diagnosis					% women given systemic therapy	
			Median/ woman	Total ('000s)	Distribution by years ('000s) <10	10-	20+	Chemotherapy‡	ER+ & Tamoxifen
**Axillary dissection**									
pN0	465	355	17.3	8.0	3.9	2.6	1.5	3	0
pN+	1029	678	6.5	10.1	6.6	2.4	1.1	39	0
pN unknown	810	499	5.6	6.4	4.7	1.5	0.2	2	0
**Total**	2304	1532	7.2	24.5	15.2	6.5	2.8	19	0

*Data available for 8 trials, start dates 1961 to 1978. In all trials radiotherapy was given to the axilla/supraclavicular fossa and the internal mammary chain. In 3 of the 8 trials radiotherapy to the chest wall was occasionally given. Full details of the trials are given in webtable 3.

† pN0: pathologically node negative, pN+: pathologically node positive, pN unknown: status not reported or staging method was clinical or unknown.

‡ Chemotherapy was cyclophosphamide, methotrexate, 5-fluorouracil [CMF], cyclophosphamide, 5-fluorouracil, prednisone [CFP], or melphalan.

Webtable 3. Randomised trials beginning before the year 2000 and comparing radiotherapy to the regional lymph nodes alone versus not after mastectomy and axillary dissection (Mast+AD) or axillary sampling (Mast+AS) – treatment details.

Year code and study name	Breast surgery	Axillary Surgery* (number of patients)	Chest wall RT	Supraclavicular and axillary fossa RT	Internal mammary chain RT	Boost RT to scar	Common systemic chemoendocrine therapy
61H NSABP B-03	RM	Axillary dissection (748)	None	35-45 Gy (1.8-2.3 Gy/f) o or c	35-45 Gy (1.8-2.3 Gy/f) o or c	None	None
64E Oslo Co-60	RM	Axillary dissection (563)	None	50 Gy de (2.5 Gy/f) c	50 Gy de (2.5 Gy/f) c	None	Ovarian irradiation
69A Heidelberg XRT	MRM	Axillary dissection (143)	None	65 Gy (2.2-2.7 Gy/f) c	65 Gy (2.2-2.7 Gy/f) c	None	None
71D SASIB	MRM, RM	Axillary dissection (377)	None for over half, others 45 Gy (4.5 Gy/f) o or c	45-60 Gy (2-4.5 Gy/f) c	40-60 Gy (2-4 Gy/f) c or e	None	None
73C Mayo 70-56-32	MRM, RM	Axillary dissection (241)	None or if skin involvement 50 Gy (2.1 Gy/f) m	50 Gy de (2.1 Gy/f) m	50 Gy de (2.1 Gy/f) m	None	CFP or not
73E INT Milan 1	RM	Axillary dissection (22)	None	40-45 Gy (1.8-2 Gy/f) c or m	40-45 Gy (1.8-2 Gy/f) c or m	None	None^
74Q Piedmont OA (excl pN4+)	MRM or RM	Axillary dissection (160)	None	45 Gy (1.5-2.8 Gy/f) u	45 Gy (1.5-2.8 Gy/f) u	None	Mel or CMF
78B Toronto-Edmont	MRM	Axillary dissection (50)	None	40 Gy de (2.5 Gy/f) c	40 Gy de (2.5 Gy/f) c	None	CMF+ovarian irradiation+P±bCG

* Based on the description of axillary surgery in the trial protocol or publications or on information on individual women. Women were classified as having axillary dissection if they were in a trial where the protocol required removal of axillary lymph nodes in at least Levels I & II or, if individual information was available, resection of ≥10 nodes. In other trials, women were classified as having axillary dissection if the trial publication indicated that the median number of nodes removed was ≥ 10. bCG=bacillus Calmette-Guérin, C=cyclophosphamide, c=cobalt-60, de=dose at depth (of nodes), F=fluorouracil, f=fraction, Gy=Gray (intended dose), m=megavoltage, M=methotrexate, Mel=melphalan, RM=modified radical mastectomy, NS=surgery not specified in detail (Patey mastectomy, or modified radical mastectomy), o=orthovoltage, P=prednisone, Patey= Patey mastectomy, RM=radical mastectomy (Halsted), RT=radiotherapy, u=unknown, ^After 1976 all patients in this trial with positive nodes received CMF chemotherapy.

References for Webtable 3

Year code and study name	Reference
61H NSABP B-03	Fisher B, Slack NH, Cavanaugh PJ, Gardner B, Ravdin RG. Postoperative radiotherapy in the treatment of breast cancer: results of the NSABP clinical trial. *Ann Surg* 1970; **172**: 711–32.
64E Oslo Co-60	Host H, Brennhovd IO, Loeb M. Postoperative radiotherapy in breast cancer-long-term results from the Oslo study. *Int J Radiat Oncol Biol Phys* 1986; **12**: 727–32.
69A Heidelberg XRT	Friedl W, Scheurlen, H.R., Amberger, H., Henningsen, B. Radiotherapy in operable breast cancer- 10 year results of a prospective randomized trial. *J Exp Clin Cancer Res* 1984; **3**: 71–7.
71D SASIB	Personal Correspondence from Dr A Hacking
73C Mayo 70-56-32	Ahmann DL, O'Fallon JR, Scanlon PW, Payne WS, Bisel HF, Edmonson JH, et al. A preliminary assessment of factors associated with recurrent disease in a surgical adjuvant clinical trial for patients with breast cancer with special emphasis on the aggressiveness of therapy. *Am J Clin Oncol* 1982; **5**: 371–81.
73E INT Milan 1	Veronesi U, Cascinelli N, Mariani L, Greco M, Saccozzi R, Luini A, et al. Twenty-year follow-up of a randomized study comparing breast-conserving surgery with radical mastectomy for early breast cancer. *N Engl J Med* 2002; **347**: 1227–32.
74Q Piedmont OA	Muss HB, Cooper MR, Brockschmidt JK, Ferree C, Richards F, 2nd, White DR, et al. A randomized trial of chemotherapy (L-PAM vs CMF) and irradiation for node positive breast cancer. Eleven year follow-up of a Piedmont Oncology Association trial. *Breast Cancer Res Treat* 1991; **19**: 77–84.
78B Toronto-Edmont	Personal Correspondence from Dr K Pritchard

Webfigure 35. Effect of radiotherapy (RT) to the regional lymph nodes alone versus not after mastectomy and axillary dissection (Mast+AD): 10-year risk of locoregional recurrence and recurrence of any type and 20-year risk of breast cancer and all-cause mortality in 465 women with pathologically node-negative (pN0) disease. See webfigure 1 for methodological note and also webfigure 36.

465 pN0 women with Mast+AD

Locoregional recurrence first

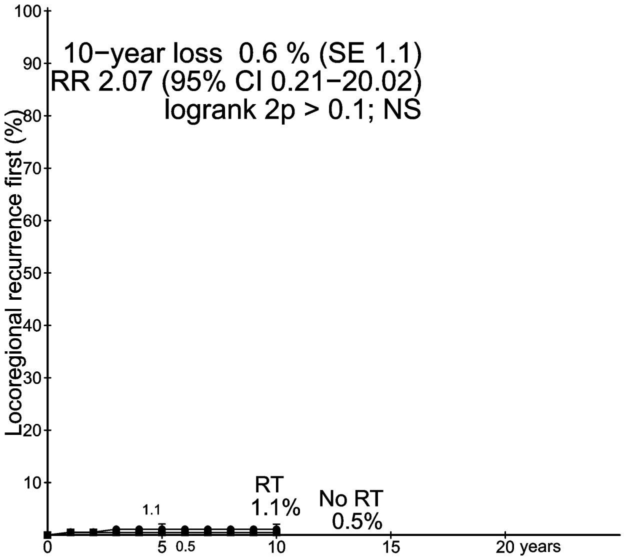

Locoregional recurrence first rates (%/year) and logrank analyses

	Years 0−4	Years 5−9
RT	0.35 (3/855)	0.00 (0/686)
No RT	0.11 (1/926)	0.00 (0/774)
Rate ratio, from	2.07 SE 1.70	. SE .
(O−E)/V	0.5/0.7	0.0/0.0

Any first recurrence

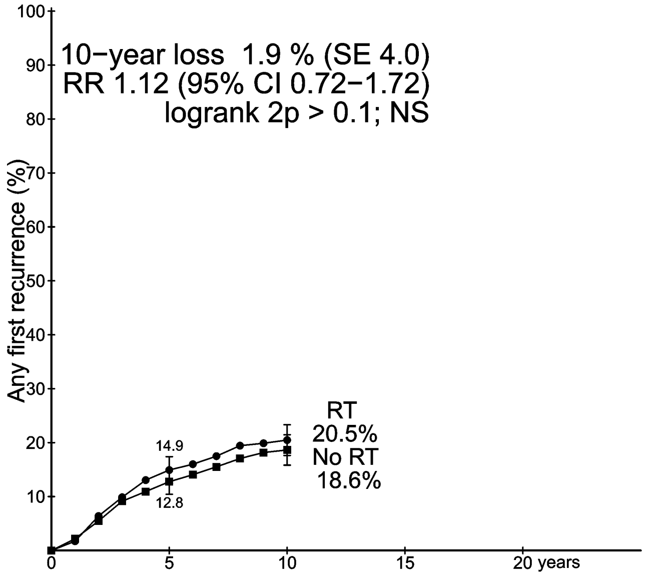

Any first recurrence rates (%/year) and logrank analyses

	Years 0−4	Years 5−9
RT	3.33 (34/1022)	1.39 (11/793)
No RT	2.94 (31/1054)	1.63 (14/858)
Rate ratio, from	1.18 SE 0.28	0.97 SE 0.42
(O−E)/V	2.4/14.8	−0.2/5.6

Breast cancer mortality

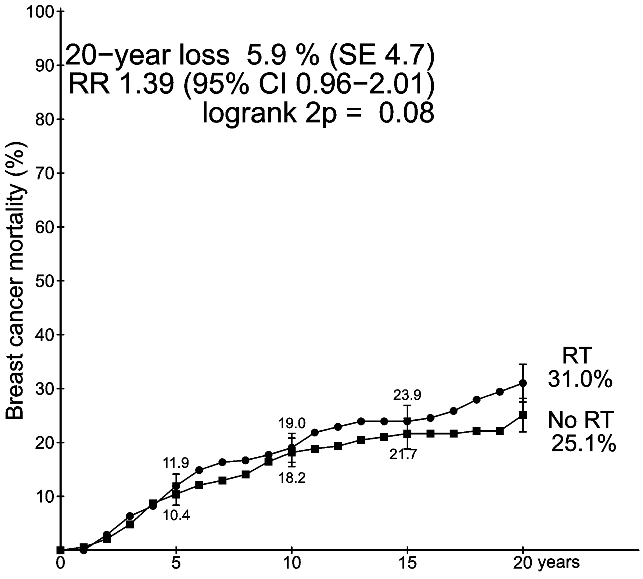

Breast cancer mortality rates (%/year) and logrank analyses

	Years 0−4	Years 5−9	Years 10−14	Years 15−19	Years 20+
RT	2.44 (26/1066)	1.65 (14/850)	1.33 (9/679)	1.98 (10/505)	2.40 (13/541)
No RT	2.19 (24/1098)	1.84 (17/924)	0.90 (7/775)	0.79 (5/631)	0.87 (8/924)
Rate ratio, from	1.17 SE 0.33	0.97 SE 0.37	1.30 SE 0.63	2.61 SE 0.92	2.79 SE 0.90
(O−E)/V	1.7/10.8	−0.3/7.1	0.9/3.2	3.2/3.4	3.9/3.8

Any death

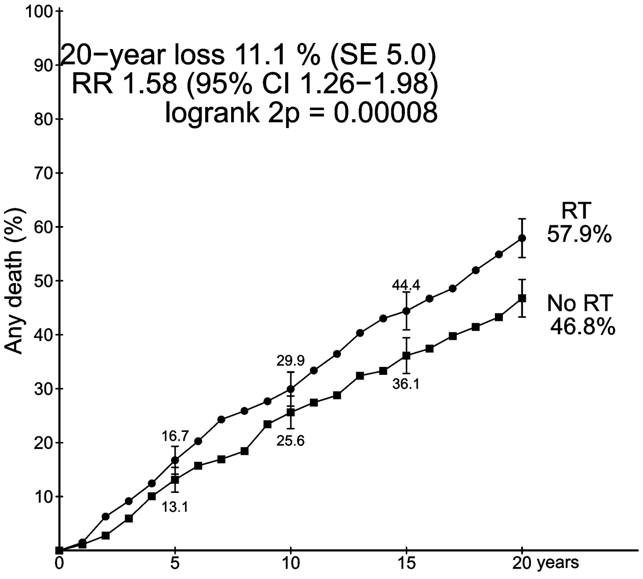

Any death rates (%/year) and logrank analyses

	Years 0−4	Years 5−9	Years 10−14	Years 15−19	Years 20+
RT	3.85 (41/1066)	3.53 (30/850)	5.15 (35/679)	5.75 (29/505)	10.91 (59/541)
No RT	2.82 (31/1098)	3.35 (31/924)	3.10 (24/775)	3.65 (23/631)	5.63 (52/924)
Rate ratio, from	1.31 SE 0.29	1.13 SE 0.29	1.56 SE 0.35	1.55 SE 0.36	2.37 SE 0.36
(O−E)/V	4.3/16.0	1.7/13.8	5.9/13.2	5.2/11.9	17.1/19.9

Webfigure 36. Effect of radiotherapy (RT) to the regional lymph nodes alone versus not after mastectomy and axillary dissection (Mast+AD): 10-year risk of recurrence and type of first recurrence, by allocated treatment, in 465 women with pathologically node-negative (pN0) disease. (r_L = number of women for whom first recurrence was locoregional, r_D = number women for whom distant recurrence was first.)

465 pN0 women with Mast+AD

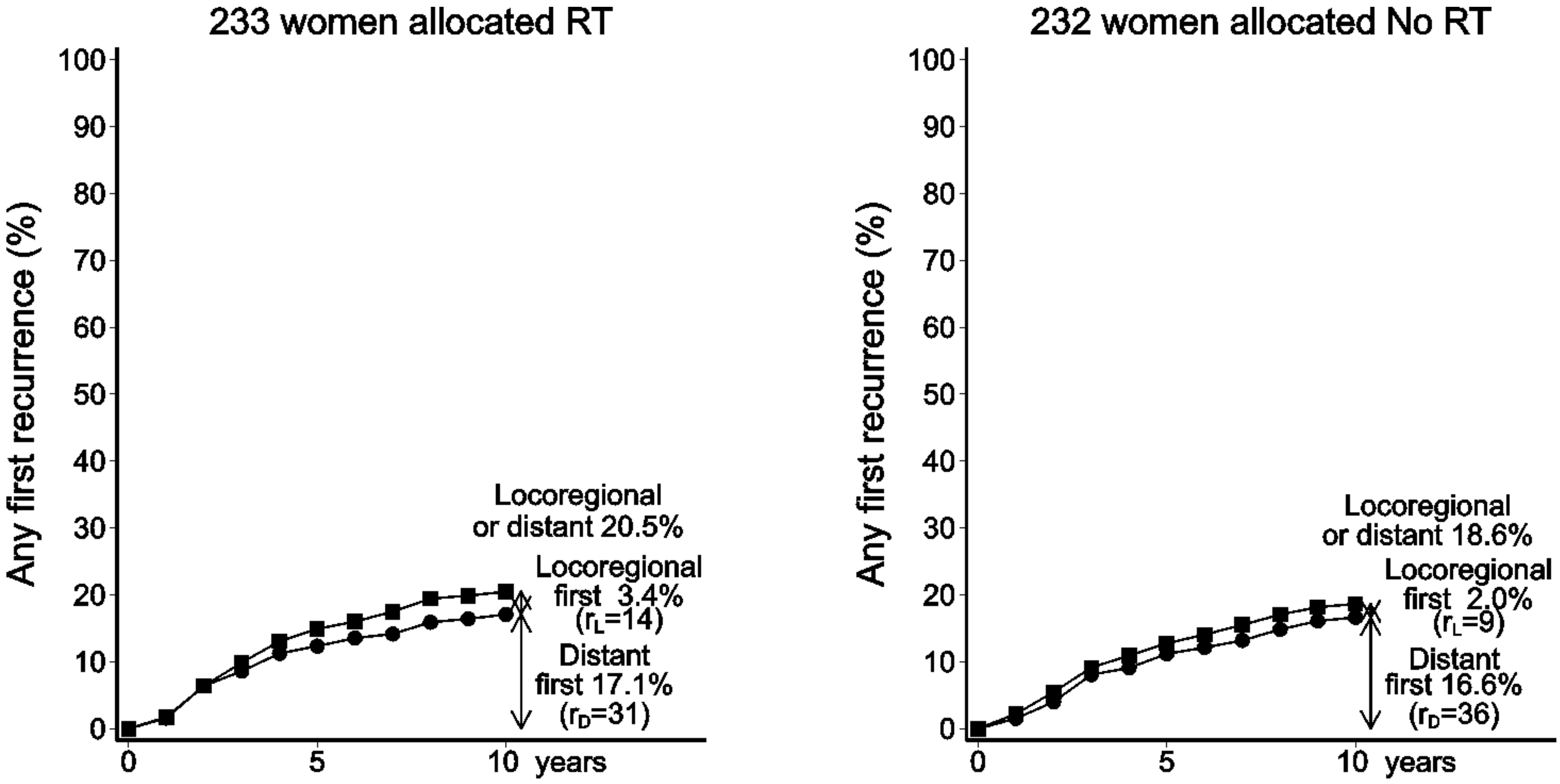

2p for difference between treatment arms in the proportion of all first recurrences that were locoregional: > 0.1; NS

Webfigure 37. Effect of radiotherapy (RT) to the regional lymph nodes alone versus not after mastectomy and axillary dissection (Mast+AD): 10-year risk of locoregional recurrence and recurrence of any type and 20-year risk of breast cancer and all-cause mortality in 1029 women with pathologically node-positive (pN+) disease. See webfigure 1 for methodological note and also webfigure 38.

1029 pN+ women with Mast+AD

Locoregional recurrence first

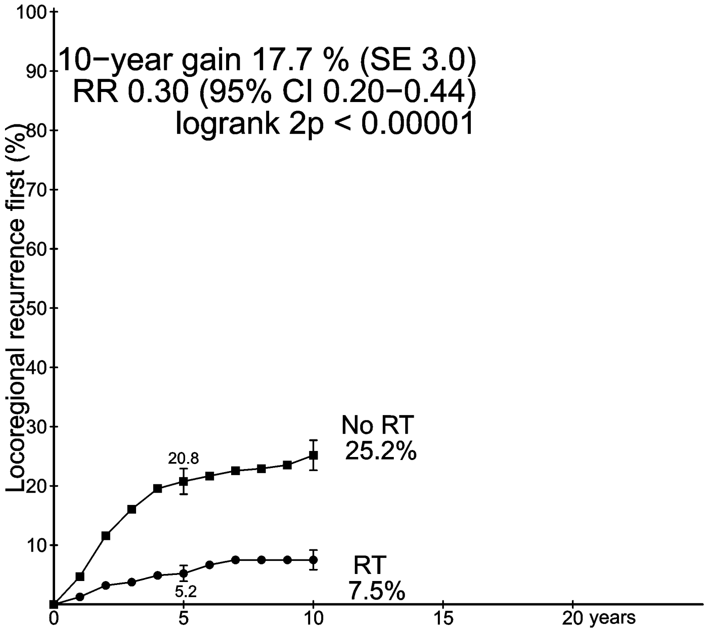

Locoregional recurrence first rates (%/year) and logrank analyses

	Years 0−4	Years 5−9
RT	1.53 (28/1827)	0.62 (6/962)
No RT	5.01 (84/1678)	1.31 (11/843)
Rate ratio, from	0.28 SE 0.12	0.47 SE 0.39
(O−E)/V	−30.0/23.6	−2.5/3.3

Any first recurrence

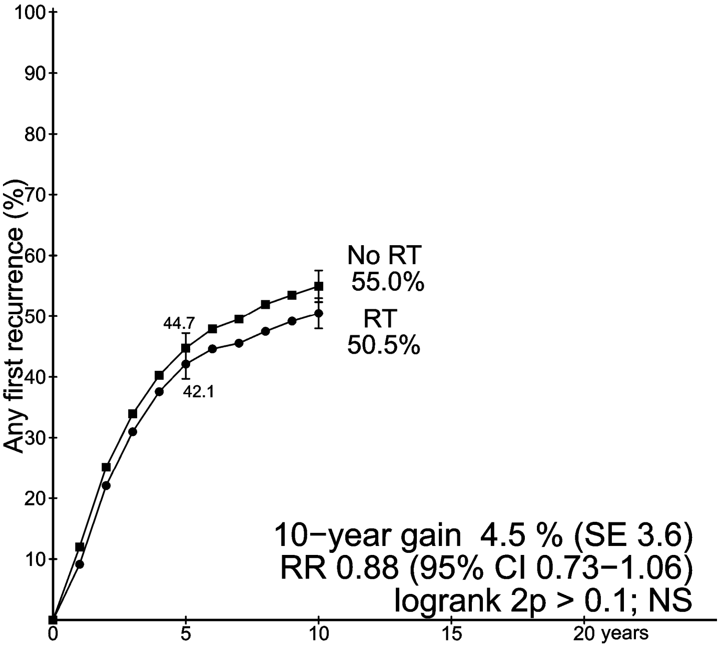

Any first recurrence rates (%/year) and logrank analyses

	Years 0−4	Years 5−9
RT	11.84 (230/1943)	3.43 (35/1020)
No RT	12.56 (222/1767)	4.55 (40/879)
Rate ratio, from	0.90 SE 0.10	0.75 SE 0.22
(O−E)/V	−9.3/90.0	−4.4/15.5

Breast cancer mortality

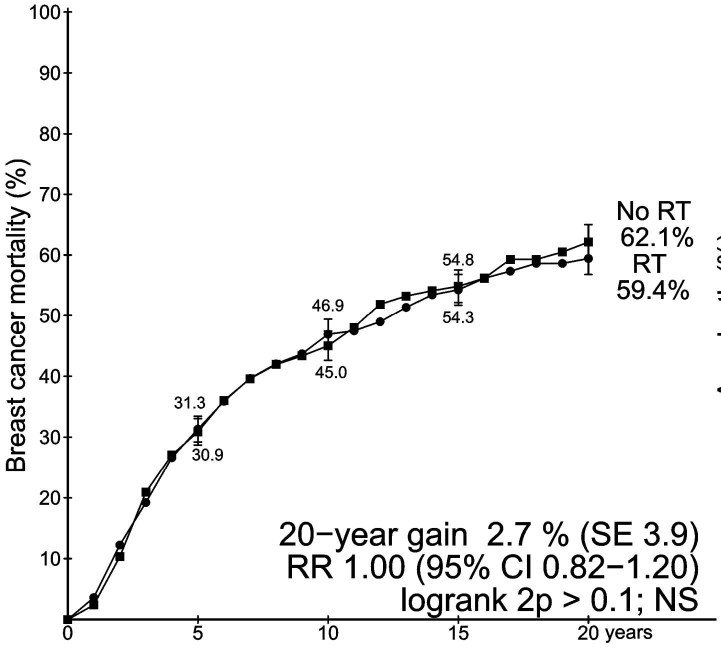

Breast cancer mortality rates (%/year) and logrank analyses

	Years 0−4	Years 5−9	Years 10−14	Years 15−19	Years 20+
RT	7.48 (165/2206)	5.69 (69/1212)	2.48 (19/767)	2.64 (13/493)	2.69 (15/558)
No RT	7.17 (149/2078)	4.42 (50/1132)	4.74 (33/696)	3.56 (16/449)	1.87 (10/536)
Rate ratio, from	1.02 SE 0.13	1.09 SE 0.21	0.69 SE 0.26	0.72 SE 0.35	1.85 SE 0.86
(O−E)/V	1.6/65.0	2.1/24.0	−3.8/10.2	−2.0/5.9	1.6/2.6

Any death

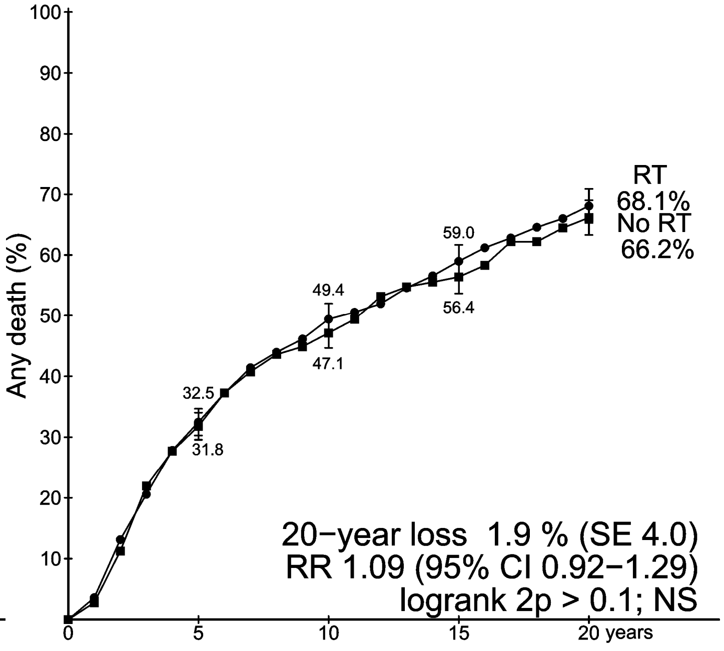

Any death rates (%/year) and logrank analyses

	Years 0−4	Years 5−9	Years 10−14	Years 15−19	Years 20+
RT	8.07 (178/2206)	7.01 (85/1212)	4.43 (34/767)	6.29 (31/493)	7.34 (41/558)
No RT	7.75 (161/2078)	5.13 (58/1132)	5.17 (36/696)	5.34 (24/449)	5.60 (30/536)
Rate ratio, from	1.03 SE 0.12	1.10 SE 0.20	0.99 SE 0.28	0.94 SE 0.30	2.23 SE 0.52
(O−E)/V	2.0/70.4	2.7/28.5	−0.2/12.8	−0.7/10.4	7.0/8.8

Webfigure 38. Effect of radiotherapy (RT) to the regional lymph nodes alone versus not after mastectomy and axillary dissection (Mast+AD): 10-year risk of recurrence and type of first recurrence, by allocated treatment, in 1029 women with pathologically node positive (pN+) disease. (r_L = number of women for whom first recurrence was locoregional, r_D = number women for whom distant recurrence was first.)

1029 pN+ women with Mast+AD

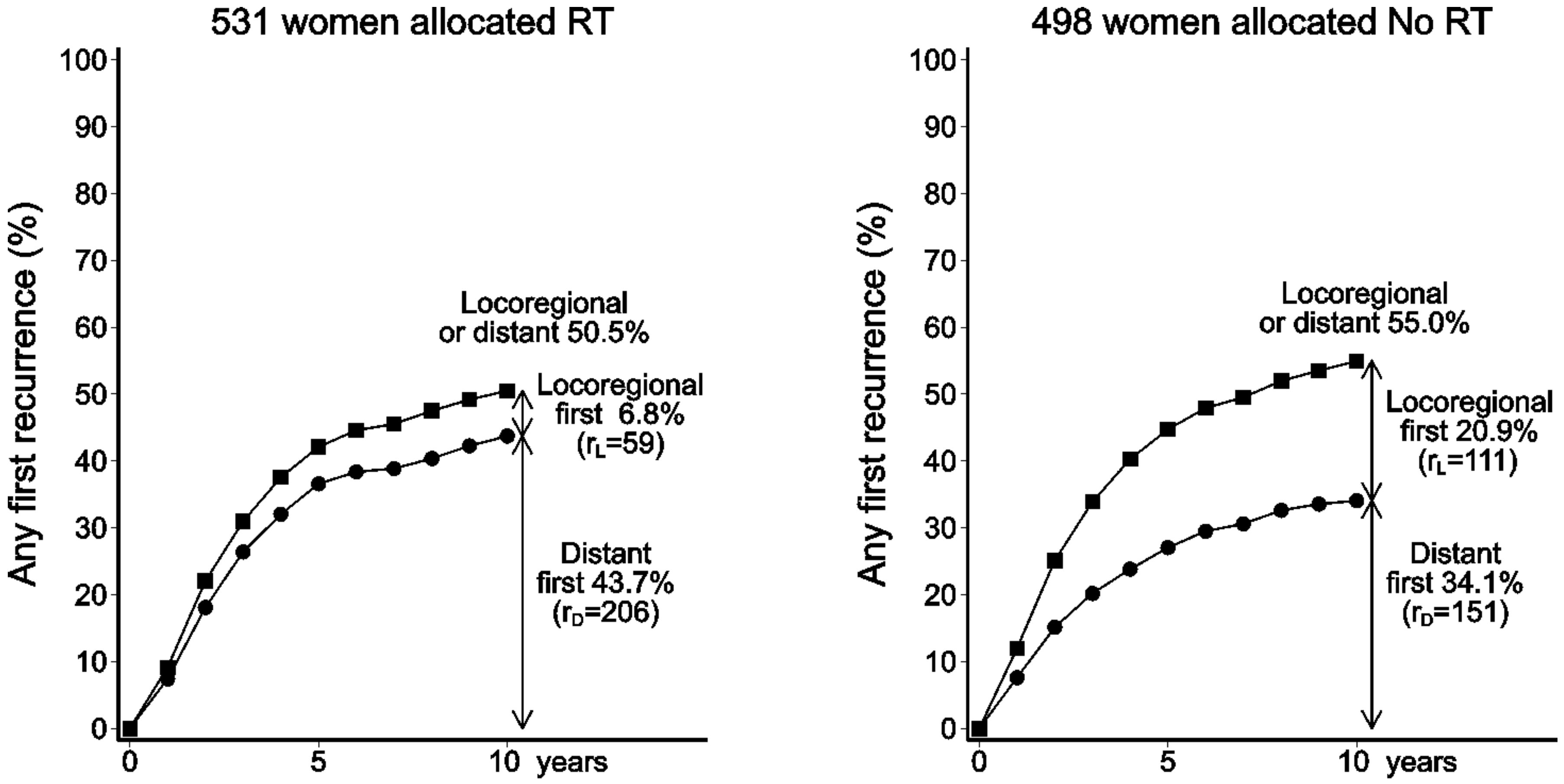

2p for difference between treatment arms in the proportion of all first recurrences that were locoregional: < 0.00001

Webfigure 39. Effect of radiotherapy (RT) to the regional lymph nodes alone versus not after mastectomy and axillary dissection (Mast+AD) or axillary sampling (Mast+AS): Event rate ratios, one line per trial, for locoregional recurrence and recurrence of any type during years 0-9 and for breast cancer and all-cause mortality in 465 women with pathologically node-negative (pN0) disease.

465 pN0 women

Locoregional recurrence first (years 0-9)

Year code, and study name	Treatment Information	Events/Women Allocated RT	Events/Women Allocated No RT	RT events Logrank O−E	RT events Variance of O−E
**(a) Nodal radiotherapy only, dissection**					
64E Oslo Co−60	AF+IMC	2/179	1/187	0.5	0.7
74Q Piedmont OA (!pN4+)	†AF+IMC	0/4	0/9		
**(a) Subtotal**		**2/ 183 (1.1%)**	**1/ 196 (0.5%)**	**0.5**	**0.7**
**(b) Some with chest wall radiotherapy, dissection**					
71D SASIB	(CW)+AF+IMC	1/6	0/5		
**(b) Subtotal**		**1/ 6 (16.7%)**	**0/ 5 (0.0%)**		
**Total**		**3/ 189 (1.6%)**	**1/ 201 (0.5%)**	**0.5**	**0.7**

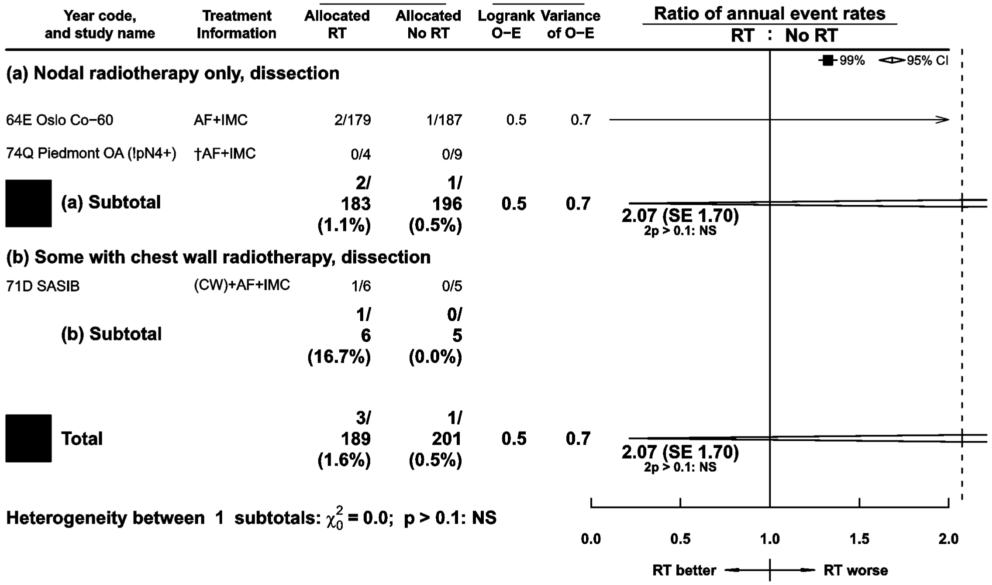

Heterogeneity between 1 subtotals: $\chi^2_0 = 0.0$; $p > 0.1$: NS

Any first recurrence (years 0-9)

Year code, and study name	Treatment Information	Events/Women Allocated RT	Events/Women Allocated No RT	RT events Logrank O−E	RT events Variance of O−E
**(a) Nodal radiotherapy only, dissection**					
64E Oslo Co−60	AF+IMC	31/179	31/187	1.3	15.1
69A Heidelberg XRT	AF+IMC	11/44	8/31	1.0	4.4
74Q Piedmont OA (!pN4+)	†AF+IMC	1/4	4/9	0.0	0.4
**(a) Subtotal**		**43/ 227 (18.9%)**	**43/ 227 (18.9%)**	**2.3**	**19.9**
**(b) Some with chest wall radiotherapy, dissection**					
71D SASIB	(CW)+AF+IMC	2/6	2/5	0.0	0.5
**(b) Subtotal**		**2/ 6 (33.3%)**	**2/ 5 (40.0%)**	**0.0**	**0.5**
**Total**		**45/ 233 (19.3%)**	**45/ 232 (19.4%)**	**2.3**	**20.4**

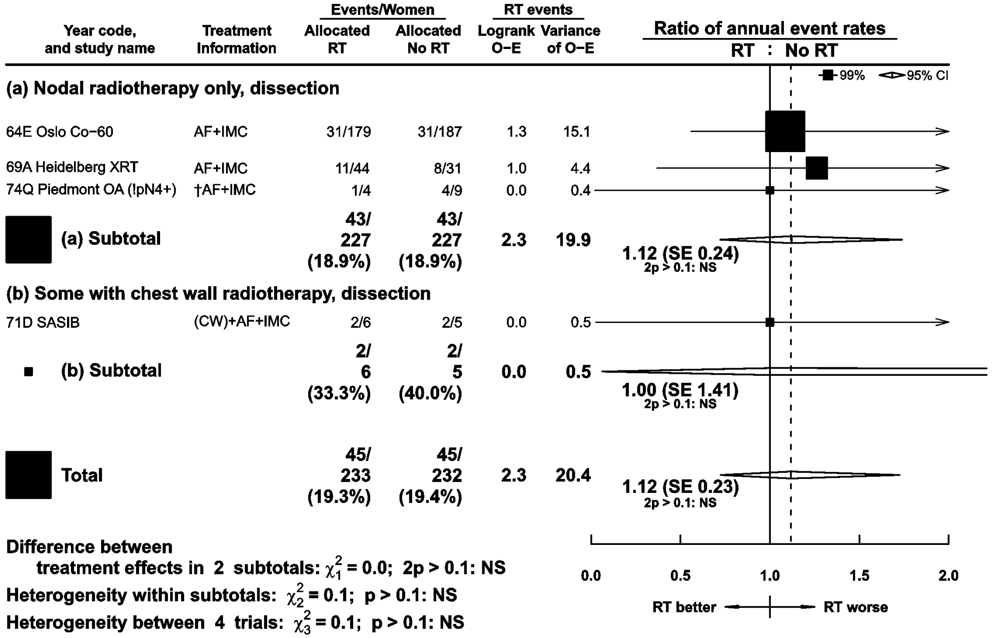

Difference between treatment effects in 2 subtotals: $\chi^2_1 = 0.0$; $2p > 0.1$: NS
Heterogeneity within subtotals: $\chi^2_2 = 0.1$; $p > 0.1$: NS
Heterogeneity between 4 trials: $\chi^2_3 = 0.1$; $p > 0.1$: NS

† Same polychemotherapy and/or tamoxifen in both groups.
Radiotherapy sites: CW=chest wall, AF=Axilla and/or supraclavicular fossa, IMC=Internal mammary chain. Site(s) in brackets were not always treated.

continued overleaf

465 pN0 women

Breast cancer mortality

Year code, and study name	Treatment Information	Deaths/Women Allocated RT	Deaths/Women Allocated No RT	RT deaths Logrank O−E	RT deaths Variance of O−E	Ratio of annual death rates RT : No RT
**(a) Nodal radiotherapy only, dissection**						
64E Oslo Co−60	AF+IMC	55/179	47/187	7.9	22.9	
69A Heidelberg XRT	AF+IMC	14/44	8/31	1.8	4.6	
74Q Piedmont OA (!pN4+)	†AF+IMC	1/4	4/9	−0.3	0.2	
**(a) Subtotal**		**70/ 227 (30.8%)**	**59/ 227 (26.0%)**	**9.4**	**27.8**	**1.40 (SE 0.23)** 2p = 0.08
**(b) Some with chest wall radiotherapy, dissection**						
71D SASIB	(CW)+AF+IMC	2/6	2/5	0.0	0.5	
**(b) Subtotal**		**2/ 6 (33.3%)**	**2/ 5 (40.0%)**	**0.0**	**0.5**	**1.00 (SE 1.41)** 2p > 0.1: NS
**Total**		**72/ 233 (30.9%)**	**61/ 232 (26.3%)**	**9.4**	**28.3**	**1.39 (SE 0.22)** 2p = 0.08

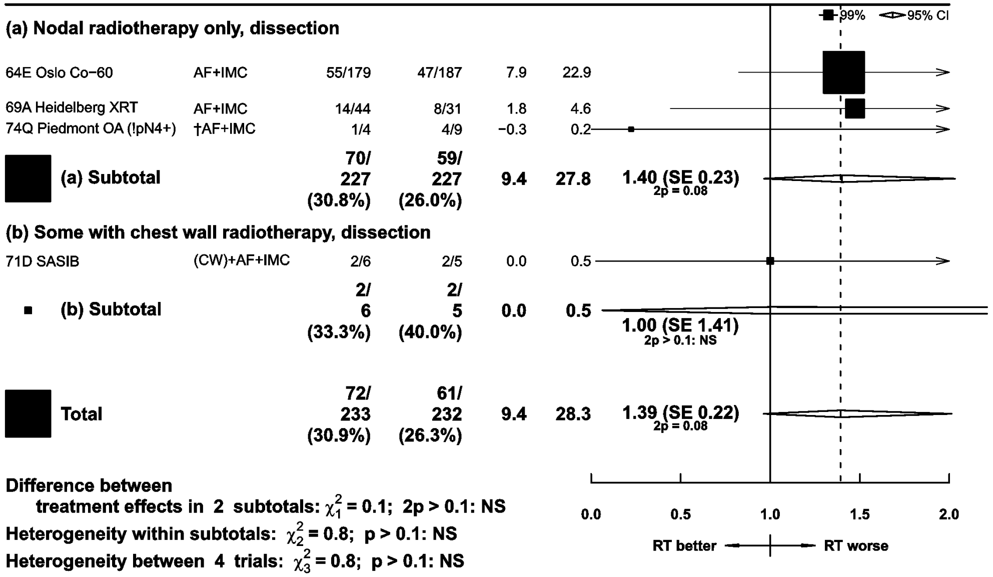

Difference between treatment effects in 2 subtotals: $\chi^2_1 = 0.1$; 2p > 0.1: NS
Heterogeneity within subtotals: $\chi^2_2 = 0.8$; p > 0.1: NS
Heterogeneity between 4 trials: $\chi^2_3 = 0.8$; p > 0.1: NS

Any death

Year code, and study name	Treatment Information	Deaths/Women Allocated RT	Deaths/Women Allocated No RT	RT deaths Logrank O−E	RT deaths Variance of O−E	Ratio of annual death rates RT : No RT
**(a) Nodal radiotherapy only, dissection**						
64E Oslo Co−60	AF+IMC	154/179	134/187	28.7	61.9	
69A Heidelberg XRT	AF+IMC	36/44	19/31	5.8	12.1	
74Q Piedmont OA (!pN4+)	†AF+IMC	1/4	6/9	−0.3	0.2	
**(a) Subtotal**		**191/ 227 (84.1%)**	**159/ 227 (70.0%)**	**34.2**	**74.3**	**1.59 (SE 0.15)** 2p = 0.00007
**(b) Some with chest wall radiotherapy, dissection**						
71D SASIB	(CW)+AF+IMC	3/6	2/5	0.0	0.5	
**(b) Subtotal**		**3/ 6 (50.0%)**	**2/ 5 (40.0%)**	**0.0**	**0.5**	**1.00 (SE 1.41)** 2p > 0.1: NS
**Total**		**194/ 233 (83.3%)**	**161/ 232 (69.4%)**	**34.2**	**74.8**	**1.58 (SE 0.15)** 2p = 0.00008

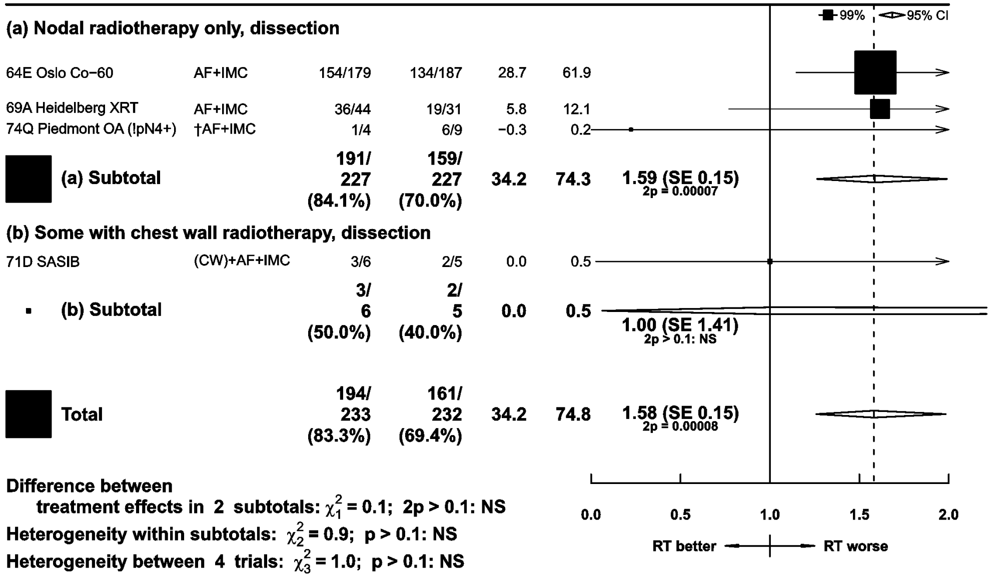

Difference between treatment effects in 2 subtotals: $\chi^2_1 = 0.1$; 2p > 0.1: NS
Heterogeneity within subtotals: $\chi^2_2 = 0.9$; p > 0.1: NS
Heterogeneity between 4 trials: $\chi^2_3 = 1.0$; p > 0.1: NS

† Same polychemotherapy and/or tamoxifen in both groups.
Radiotherapy sites: CW=chest wall, AF=Axilla and/or supraclavicular fossa, IMC=Internal mammary chain. Site(s) in brackets were not always treated.

Webfigure 40. Effect of radiotherapy (RT) to the regional lymph nodes alone versus not after mastectomy and axillary dissection (Mast+AD) or axillary sampling (Mast+AS): Event rate ratios, one line per trial, for locoregional recurrence and recurrence of any type during years 0-9 and for breast cancer and all-cause mortality in 1029 women with pathologically node-positive (pN+) disease.

1029 pN+ women

Locoregional recurrence first (years 0-9)

Year code, and study name	Treatment Information	Events/Women: Allocated RT	Events/Women: Allocated No RT	RT events: Logrank O−E	RT events: Variance of O−E	Ratio of annual event rates RT : No RT
**(a) Nodal radiotherapy only, dissection**						
64E Oslo Co−60	AF+IMC	1/99	6/98	−2.4	1.6	
73E INT Milan 1	AF+IMC	0/15	1/7			
74Q Piedmont OA (!pN4+)	†AF+IMC	0/67	8/61	−4.2	1.[illegible]	
78B Toronto−Edmont.	†AF+IMC	6/28	4/21	−0.5	1.6	
**(a) Subtotal**		**7/ 209 (3.3%)**	**19/ 187 (10.2%)**	**−7.0**	**5.1**	**0.25 (SE 0.24)** 2p = 0.002
**(b) Some with chest wall radiotherapy, dissection**						
71D SASIB	(CW)+AF+IMC	13/162	38/164	−13.3	11.6	
73C Mayo 70−56−32	†(CW)+AF+IMC	14/121	38/120	−12.2	10.2	
**(b) Subtotal**		**27/ 283 (9.5%)**	**76/ 284 (26.8%)**	**−25.5**	**21.8**	**0.31 (SE 0.13)** 2p < 0.00001
**Total**		**34/ 492 (6.9%)**	**95/ 471 (20.2%)**	**−32.5**	**26.9**	**0.30 (SE 0.11)** 2p < 0.00001

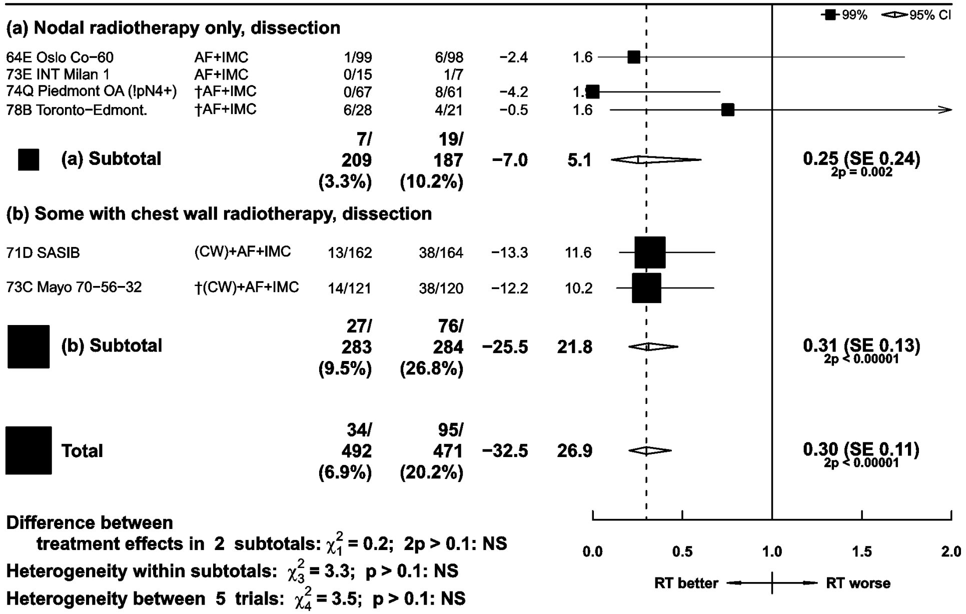

Difference between treatment effects in 2 subtotals: $\chi^2_1 = 0.2$; 2p > 0.1: NS
Heterogeneity within subtotals: $\chi^2_3 = 3.3$; p > 0.1: NS
Heterogeneity between 5 trials: $\chi^2_4 = 3.5$; p > 0.1: NS

Any first recurrence (years 0-9)

Year code, and study name	Treatment Information	Events/Women: Allocated RT	Events/Women: Allocated No RT	RT events: Logrank O−E	RT events: Variance of O−E	Ratio of annual event rates RT : No RT
**(a) Nodal radiotherapy only, dissection**						
64E Oslo Co−60	AF+IMC	33/99	47/98	−7.6	17.2	
69A Heidelberg XRT	AF+IMC	25/39	16/27	1.0	8.2	
73E INT Milan 1	AF+IMC	8/15	2/7	1.8	1.4	
74Q Piedmont OA (!pN4+)	†AF+IMC	32/67	19/61	4.5	11.0	
78B Toronto−Edmont.	†AF+IMC	15/28	10/21	1.7	3.9	
**(a) Subtotal**		**113/ 248 (45.6%)**	**94/ 214 (43.9%)**	**1.3**	**41.7**	**1.03 (SE 0.16)** 2p > 0.1: NS
**(b) Some with chest wall radiotherapy, dissection**						
71D SASIB	(CW)+AF+IMC	85/162	93/164	−6.7	36.9	
73C Mayo 70−56−32	†(CW)+AF+IMC	67/121	75/120	−8.3	26.9	
**(b) Subtotal**		**152/ 283 (53.7%)**	**168/ 284 (59.2%)**	**−15.0**	**63.8**	**0.79 (SE 0.11)** 2p = 0.06
**Total**		**265/ 531 (49.9%)**	**262/ 498 (52.6%)**	**−13.7**	**105.5**	**0.88 (SE 0.09)** 2p > 0.1: NS

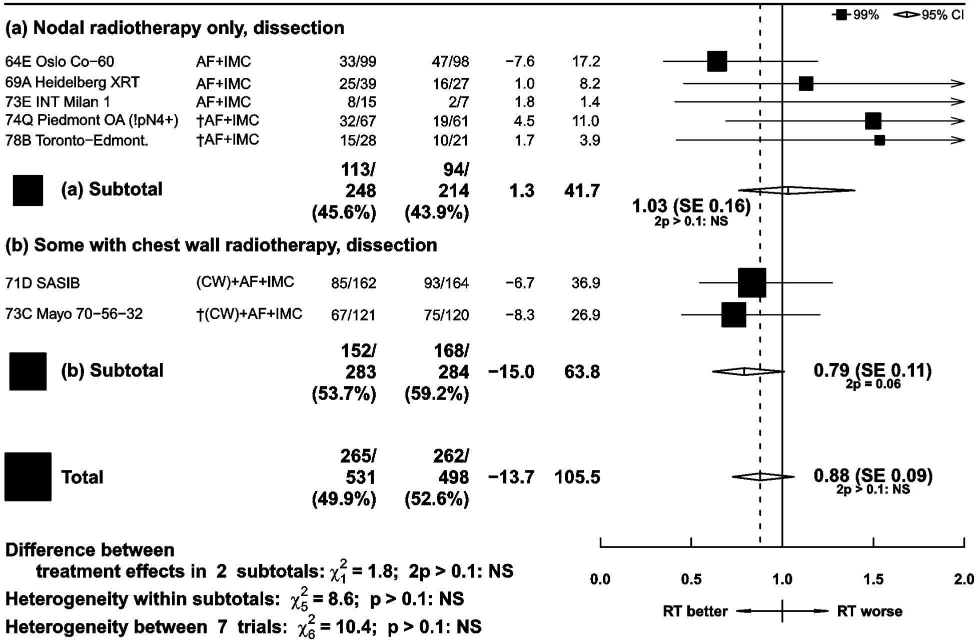

Difference between treatment effects in 2 subtotals: $\chi^2_1 = 1.8$; 2p > 0.1: NS
Heterogeneity within subtotals: $\chi^2_5 = 8.6$; p > 0.1: NS
Heterogeneity between 7 trials: $\chi^2_6 = 10.4$; p > 0.1: NS

† Same polychemotherapy and/or tamoxifen in both groups.
Radiotherapy sites: CW=chest wall, AF=Axilla and/or supraclavicular fossa, IMC=Internal mammary chain. Site(s) in brackets were not always treated.

continued overleaf

1029 pN+ women

Breast cancer mortality

Year code, and study name	Treatment Information	Deaths/Women Allocated RT	Deaths/Women Allocated No RT	RT deaths Logrank O−E	RT deaths Variance of O−E	Ratio of annual death rates RT : No RT
**(a) Nodal radiotherapy only, dissection**						
64E Oslo Co−60	AF+IMC	47/99	58/98	−8.7	22.1	
69A Heidelberg XRT	AF+IMC	27/39	18/27	0.9	8.6	
73E INT Milan 1	AF+IMC	9/15	4/7	1.5	2.1	
74Q Piedmont OA (!pN4+)	†AF+IMC	32/67	18/61	6.5	11.0	
78B Toronto−Edmont.	†AF+IMC	14/28	13/21	0.0	4.2	
**(a) Subtotal**		**129/ 248 (52.0%)**	**111/ 214 (51.9%)**	**0.2**	**48.0**	**1.00 (SE 0.14)** 2p > 0.1: NS
**(b) Some with chest wall radiotherapy, dissection**						
71D SASIB	(CW)+AF+IMC	68/162	62/164	2.8	27.4	
73C Mayo 70−56−32	†(CW)+AF+IMC	84/121	85/120	−3.5	32.3	
**(b) Subtotal**		**152/ 283 (53.7%)**	**147/ 284 (51.8%)**	**−0.7**	**59.6**	**0.99 (SE 0.13)** 2p > 0.1: NS
**Total**		**281/ 531 (52.9%)**	**258/ 498 (51.8%)**	**−0.5**	**107.6**	**1.00 (SE 0.10)** 2p > 0.1: NS

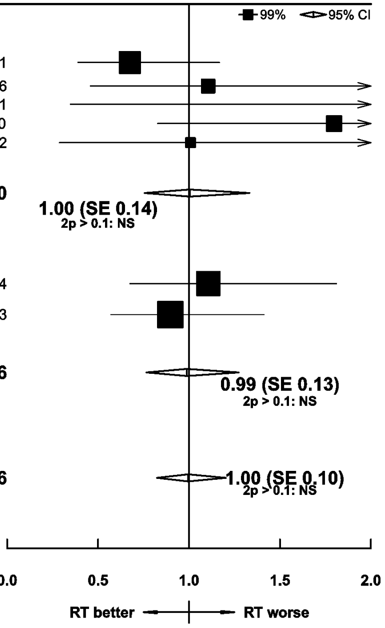

Difference between treatment effects in 2 subtotals: $\chi^2_1 = 0.0$; 2p > 0.1: NS
Heterogeneity within subtotals: $\chi^2_5 = 9.0$; p > 0.1: NS
Heterogeneity between 7 trials: $\chi^2_6 = 9.0$; p > 0.1: NS

Any death

Year code, and study name	Treatment Information	Deaths/Women Allocated RT	Deaths/Women Allocated No RT	RT deaths Logrank O−E	RT deaths Variance of O−E	Ratio of annual death rates RT : No RT
**(a) Nodal radiotherapy only, dissection**						
64E Oslo Co−60	AF+IMC	88/99	79/98	−0.6	32.4	
69A Heidelberg XRT	AF+IMC	37/39	24/27	1.3	11.9	
73E INT Milan 1	AF+IMC	11/15	5/7	1.5	2.1	
74Q Piedmont OA (!pN4+)	†AF+IMC	38/67	23/61	6.8	12.8	
78B Toronto−Edmont.	†AF+IMC	15/28	13/21	0.5	4.4	
**(a) Subtotal**		**189/ 248 (76.2%)**	**144/ 214 (67.3%)**	**9.4**	**63.7**	**1.16 (SE 0.14)** 2p > 0.1: NS
**(b) Some with chest wall radiotherapy, dissection**						
71D SASIB	(CW)+AF+IMC	76/162	65/164	4.7	29.8	
73C Mayo 70−56−32	†(CW)+AF+IMC	104/121	100/120	−3.2	37.3	
**(b) Subtotal**		**180/ 283 (63.6%)**	**165/ 284 (58.1%)**	**1.5**	**67.1**	**1.02 (SE 0.12)** 2p > 0.1: NS
**Total**		**369/ 531 (69.5%)**	**309/ 498 (62.0%)**	**10.9**	**130.8**	**1.09 (SE 0.09)** 2p > 0.1: NS

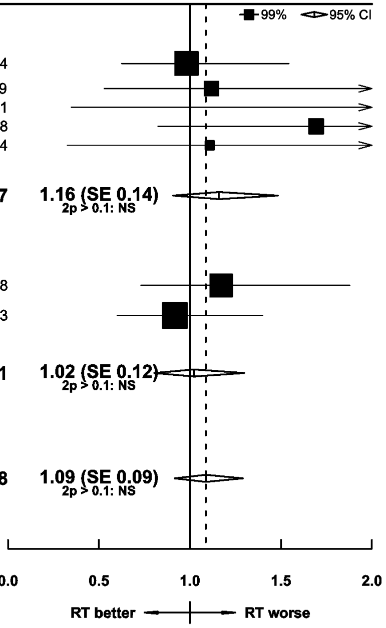

Difference between treatment effects in 2 subtotals: $\chi^2_1 = 0.5$; 2p > 0.1: NS
Heterogeneity within subtotals: $\chi^2_5 = 4.4$; p > 0.1: NS
Heterogeneity between 7 trials: $\chi^2_6 = 4.9$; p > 0.1: NS

† Same polychemotherapy and/or tamoxifen in both groups.
Radiotherapy sites: CW=chest wall, AF=Axilla and/or supraclavicular fossa, IMC=Internal mammary chain. Site(s) in brackets were not always treated.

Webfigure 41. Effect of radiotherapy (RT) to the regional lymph nodes alone versus not after mastectomy and axillary dissection (Mast+AD) or axillary sampling (Mast+AS): Event rate ratios, one line per trial, for locoregional recurrence and recurrence of any type during years 0-9 and for breast cancer and all-cause mortality in 810 women with unknown pathological nodal status (pN?).

810 pN? women

Locoregional recurrence first (years 0-9)

Year code, and study name	Treatment Information	Events/Women Allocated RT	Events/Women Allocated No RT	RT events Logrank O−E	RT events Variance of O−E	Ratio of annual event rates RT : No RT
**(a) Nodal radiotherapy only, dissection**						
74Q Piedmont OA (!pN4+)	†AF+IMC	0/8	0/11			
78B Toronto−Edmont.	†AF+IMC	0/0	0/1			
**(a) Subtotal**		**0/ 8 (0.0%)**	**0/ 12 (0.0%)**			
**(b) Some with chest wall radiotherapy, dissection**						
71D SASIB	(CW)+AF+IMC	4/18	4/22	0.3	1.8	
**(b) Subtotal**		**4/ 18 (22.2%)**	**4/ 22 (18.2%)**	**0.3**	**1.8**	**1.20 (SE 0.81)** 2p > 0.1: NS
**Total**		**4/ 26 (15.4%)**	**4/ 34 (11.8%)**	**0.3**	**1.8**	**1.20 (SE 0.81)** 2p > 0.1: NS

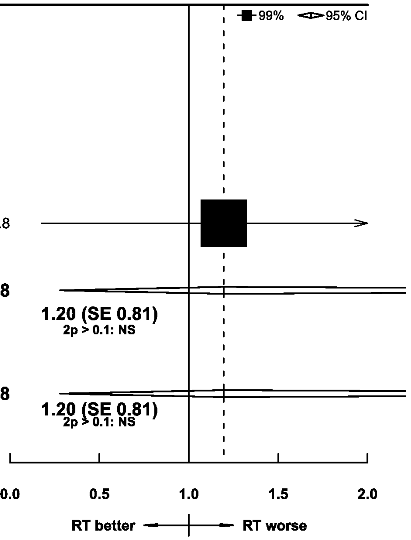

Heterogeneity between 1 subtotals: $\chi^2_0 = 0.0$; p > 0.1: NS

Any first recurrence (years 0-9)

Year code, and study name	Treatment Information	Events/Women Allocated RT	Events/Women Allocated No RT	RT events Logrank O−E	RT events Variance of O−E	Ratio of annual event rates RT : No RT
**(a) Nodal radiotherapy only, dissection**						
61H NSABP B−03*	AF+IMC	196/476	216/544	−0.3	57.2	
69A Heidelberg XRT	AF+IMC	0/2	0/0			
74Q Piedmont OA (!pN4+)	†AF+IMC	4/8	5/11	−0.1	1.6	
78B Toronto−Edmont.	†AF+IMC	0/0	1/1			
**(a) Subtotal**		**200/ 486 (41.2%)**	**222/ 556 (39.9%)**	**−0.4**	**58.8**	**0.99 (SE 0.13)** 2p > 0.1: NS
**(b) Some with chest wall radiotherapy, dissection**						
71D SASIB	(CW)+AF+IMC	15/18	11/22	4.3	4.8	
**(b) Subtotal**		**15/ 18 (83.3%)**	**11/ 22 (50.0%)**	**4.3**	**4.8**	**2.44 (SE 0.74)** 2p = 0.05
**Total**		**215/ 504 (42.7%)**	**233/ 578 (40.3%)**	**3.8**	**63.5**	**1.06 (SE 0.13)** 2p > 0.1: NS

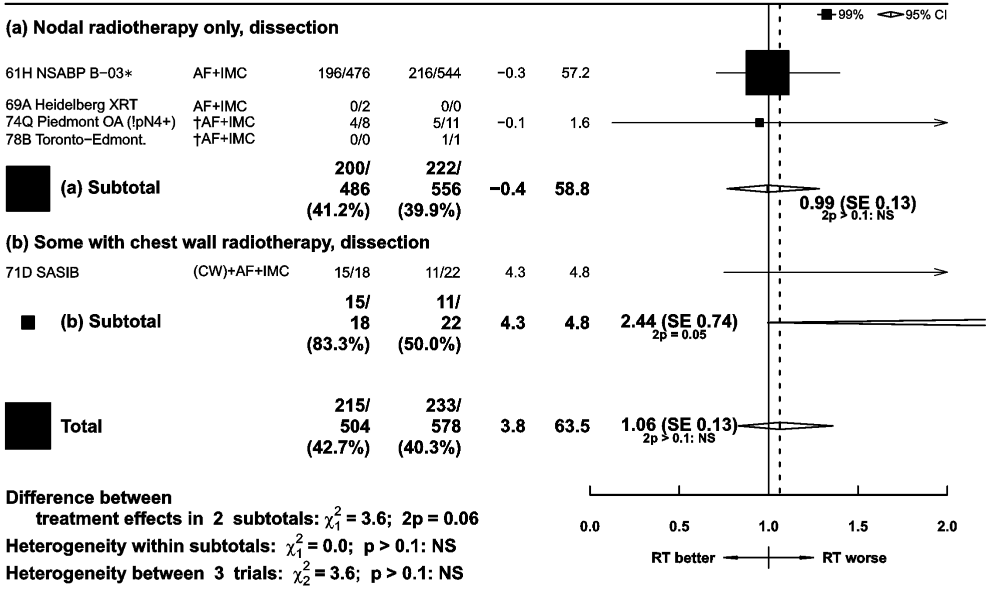

Difference between treatment effects in 2 subtotals: $\chi^2_1 = 3.6$; 2p = 0.06
Heterogeneity within subtotals: $\chi^2_1 = 0.0$; p > 0.1: NS
Heterogeneity between 3 trials: $\chi^2_2 = 3.6$; p > 0.1: NS

* For balance, control patients in NSABP B-03 count twice in subtotal and final total of events/deaths/women.
† Same polychemotherapy and/or tamoxifen in both groups.
Radiotherapy sites: CW=chest wall, AF=Axilla and/or supraclavicular fossa, IMC=Internal mammary chain. Site(s) in brackets were not always treated.

continued overleaf

Webfigure 41 cntd.

810 pN? women

Breast cancer mortality

Year code, and study name	Treatment Information	Deaths/Women Allocated RT	Deaths/Women Allocated No RT	RT deaths Logrank O−E	RT deaths Variance of O−E	Ratio of annual death rates RT : No RT
**(a) Nodal radiotherapy only, dissection**						
61H NSABP B−03*	AF+IMC	185/476	198/544	2.7	54.6	
69A Heidelberg XRT	AF+IMC	0/2	0/0			
74Q Piedmont OA (!pN4+)	†AF+IMC	5/8	5/11	0.6	1.8	
78B Toronto−Edmont.	†AF+IMC	0/0	1/1			
**(a) Subtotal**		**190/ 486 (39.1%)**	**204/ 556 (36.7%)**	**3.3**	**56.4**	**1.06 (SE 0.14)** 2p > 0.1: NS
**(b) Some with chest wall radiotherapy, dissection**						
71D SASIB	(CW)+AF+IMC	9/18	7/22	2.6	3.3	
**(b) Subtotal**		**9/ 18 (50.0%)**	**7/ 22 (31.8%)**	**2.6**	**3.3**	**2.22 (SE 0.84)** 2p > 0.1: NS
**Total**		**199/ 504 (39.5%)**	**211/ 578 (36.5%)**	**5.9**	**59.8**	**1.10 (SE 0.14)** 2p > 0.1: NS

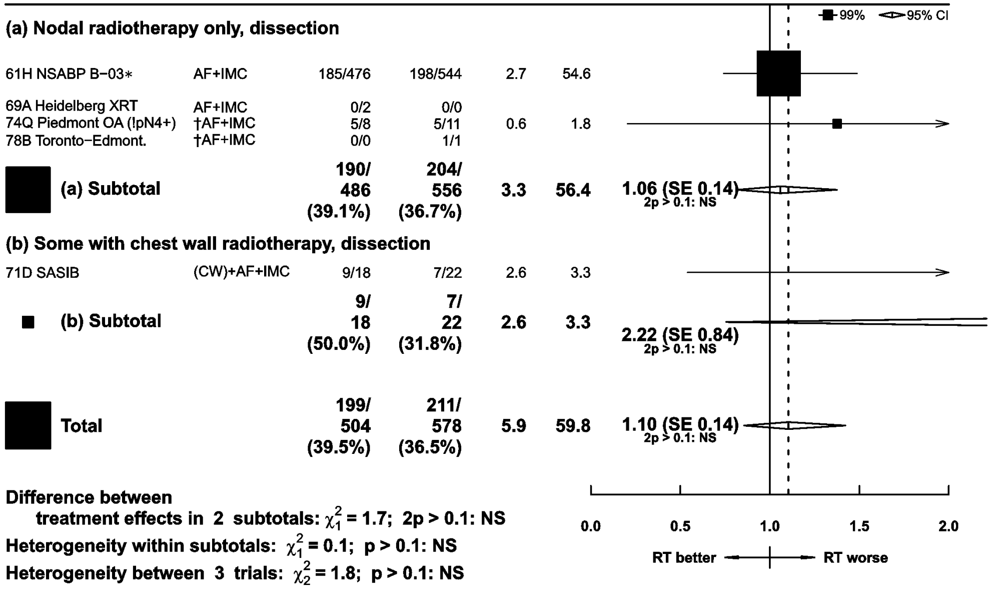

Difference between treatment effects in 2 subtotals: $\chi^2_1 = 1.7$; 2p > 0.1: NS
Heterogeneity within subtotals: $\chi^2_1 = 0.1$; p > 0.1: NS
Heterogeneity between 3 trials: $\chi^2_2 = 1.8$; p > 0.1: NS

Any death

Year code, and study name	Treatment Information	Deaths/Women Allocated RT	Deaths/Women Allocated No RT	RT deaths Logrank O−E	RT deaths Variance of O−E	Ratio of annual death rates RT : No RT
**(a) Nodal radiotherapy only, dissection**						
61H NSABP B−03*	AF+IMC	303/476	334/544	1.2	89.8	
69A Heidelberg XRT	AF+IMC	1/2	0/0			
74Q Piedmont OA (!pN4+)	†AF+IMC	5/8	5/11	0.6	1.8	
78B Toronto−Edmont.	†AF+IMC	0/0	1/1			
**(a) Subtotal**		**309/ 486 (63.6%)**	**340/ 556 (61.2%)**	**1.8**	**91.7**	**1.02 (SE 0.11)** 2p > 0.1: NS
**(b) Some with chest wall radiotherapy, dissection**						
71D SASIB	(CW)+AF+IMC	10/18	7/22	3.3	3.5	
**(b) Subtotal**		**10/ 18 (55.6%)**	**7/ 22 (31.8%)**	**3.3**	**3.5**	**2.55 (SE 0.88)** 2p = 0.08
**Total**		**319/ 504 (63.3%)**	**347/ 578 (60.0%)**	**5.1**	**95.2**	**1.05 (SE 0.11)** 2p > 0.1: NS

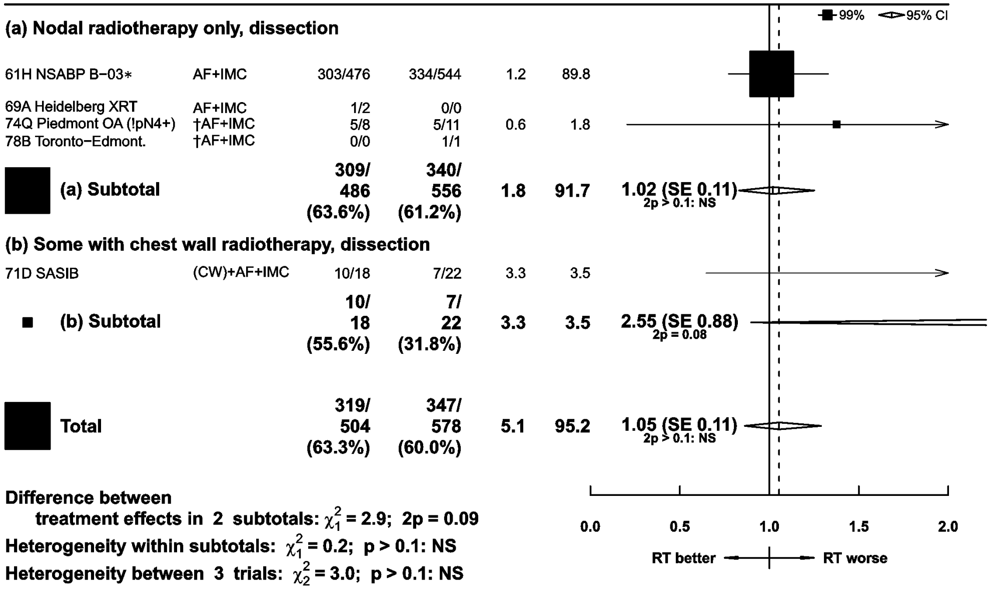

Difference between treatment effects in 2 subtotals: $\chi^2_1 = 2.9$; 2p = 0.09
Heterogeneity within subtotals: $\chi^2_1 = 0.2$; p > 0.1: NS
Heterogeneity between 3 trials: $\chi^2_2 = 3.0$; p > 0.1: NS

* For balance, control patients in NSABP B-03 count twice in subtotal and final total of events/deaths/women.
† Same polychemotherapy and/or tamoxifen in both groups.
Radiotherapy sites: CW=chest wall, AF=Axilla and/or supraclavicular fossa, IMC=Internal mammary chain. Site(s) in brackets were not always treated.

Webtable 4. Availability of data from randomised trials beginning before the year 2000 and comparing radiotherapy to the regional lymph nodes alone versus not after mastectomy but no axillary surgery (Mast)*.

Nodal status†	Women	Deaths	Woman-years since diagnosis					% women given systemic therapy		
			Median/ woman	Total ('000s)	Distribution by years ('000s) <10	10-	20+	Chemotherapy	ER+ & Tamoxifen	Any
**Axillary dissection**										
**cN-**	**2896**	**2098**	**12.4**	**45.3**	**21.7**	**12.8**	**10.8**	**0**	**2**	**2**
**cN+**	**1481**	**1188**	**9.6**	**21.5**	**10.5**	**5.9**	**5.1**	**0**	**1**	**1**
**Total**	**4377**	**3286**	**11.5**	**66.8**	**32.2**	**18.7**	**15.9**	**0**	**2**	**2**

*Data available for 4 trials, start dates 1970 to 1978. In all trials radiotherapy was given to the axilla/supraclavicular fossa and the internal mammary chain. Full details of the trials are given in webtable 5.

† cN-: negative clinical nodal status, cN+: positive clinical nodal status.

Webtable 5: Randomised trials beginning before the year 2000 and comparing radiotherapy to the chest wall and regional lymph nodes versus not after mastectomy but no axillary surgery (Mast) – treatment details.

Year code and study name	Breast surgery	Axillary Surgery* (number of patients)	Chest wall RT	Supraclavicular and axillary fossa RT	Internal mammary chain RT	Boost RT to scar	Common systemic chemoendocrine therapy
70A Manchester RBS1	SM	Axillary sampling (714)	30-37 Gy (2-2.5 Gy/f) o	37-40 Gy (2.5-2.7 Gy/f) o or m	37-40 Gy (2.5-2.7 Gy/f) o or m	None	Ovarian ablation
70B Kings/Cambridge	SM	Axillary sampling (2,800)	28.5-46 Gy (1.5-3.2 Gy/f) o or s	28.5-46 Gy (1.5-3.2 Gy/f) o or s	28.5-46 Gy (1.5-3.2 Gy/f) o or s	None	None
71C NSABP B-04	SM	Axillary sampling (770)	50 Gy (2 Gy/f) s	45-50 Gy de (1.8-2.0 Gy/f) s	45 Gy de (1.8 Gy/f) s	None	None
78D Scottish D	SM	Axillary sampling (93)	37-45 Gy (2.3-3.7 Gy/f) o or m	38.4-45.9 Gy (2.3-3.8 Gy/f) o or m	40-45 Gy (2.3-2.7 Gy/f) o or m	None	Tamoxifen or not

* Based on the description of axillary surgery in the trial protocol or publications or on lymph node information on individual women. Women were classified as having axillary sampling if they were in a trial where the protocol specified axillary sampling or, if individual information was available, resection of <10 nodes. In other trials, women were classified as having axillary sampling if the trial publication indicated that the median number of nodes removed was < 10, f=fraction, Gy=Gray (intended dose), m=megavoltage, RM=modified radical mastectomy, o=orthovoltage, RM=radical mastectomy (Halsted), RT=radiotherapy, SM=simple (total) mastectomy.

References for Webtable 5

Year code and study name	Reference
70A Manchester RBS1	Lythgoe JP, Palmer MK. Manchester regional breast study-5 and 10 year results. *Br J Surg* 1982; **69**: 693–6.
70B Kings/Cambridge	Houghton J, Baum M, Haybittle JL. Role of radiotherapy following total mastectomy in patients with early breast cancer. The Closed Trials Working Party of the CRC Breast Cancer Trials Group. *World J Surg* 1994; **18**: 117–22.
71C NSABP B-04	Fisher B, Montague E, Redmond C, Deutsch M, Brown GR, Zauber A, et al. Findings from NSABP protocol no. B-04-comparison of radical mastectomy with alternative treatments for primary breast cancer. I. Radiation compliance and its relation to treatment outcome. *Cancer* 1980; **46**: 1–13.
	Deutsch M, Land S, Begovic M, Sharif S. The incidence of arm edema in women with breast cancer randomized on the National Surgical Adjuvant Breast and Bowel Project study B-04 to radical mastectomy versus total mastectomy and radiotherapy versus total mastectomy alone. *Int J Radiat Oncol Biol Phys* 2008; **70**: 1020–4.
78D Scottish D	Stewart HJ, Prescott RJ, Forrest AP. Scottish adjuvant tamoxifen trial: a randomized study updated to 15 years. *J Natl Cancer Inst* 2001; **93**: 456–62.

Webfigure 42. Effect of radiotherapy (RT) to the chest wall and regional lymph nodes versus not after mastectomy but no axillary surgery (Mast): 10-year risk of locoregional recurrence and recurrence of any type and 20-year risks of breast cancer and all-cause mortality in 2896 women with clinically node-negative (cN-) disease. See webfigure 1 for methodological note and also webfigure 43
.

2896 cN- women with Mast

Locoregional recurrence first

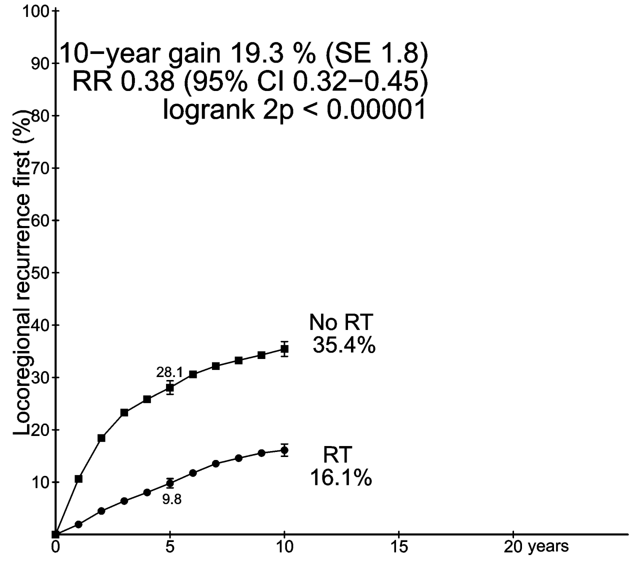

Locoregional recurrence first rates (%/year) and logrank analyses

	Years 0−4	Years 5−9
RT	2.12 (119/5612)	1.51 (56/3714)
No RT	7.11 (374/5259)	2.25 (77/3422)
Rate ratio, from	0.32 SE 0.06	0.68 SE 0.15
(O−E)/V	−130.5/115.8	−12.5/32.2

Any first recurrence

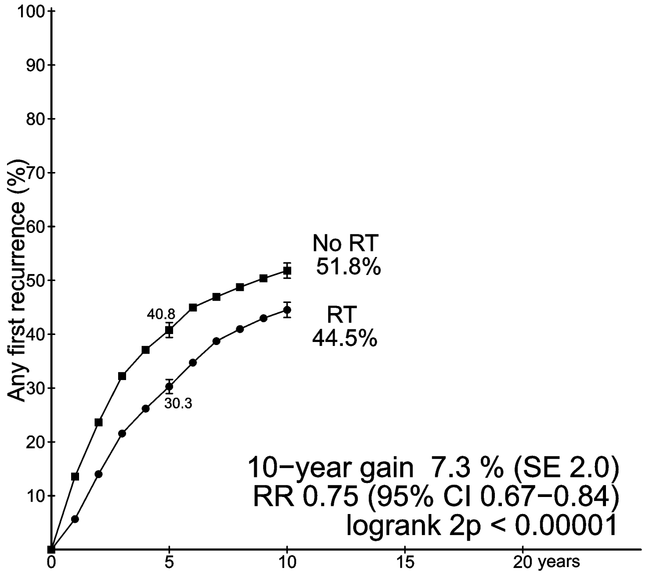

Any first recurrence rates (%/year) and logrank analyses

	Years 0−4	Years 5−9
RT	7.35 (415/5647)	4.67 (178/3810)
No RT	10.94 (577/5274)	4.24 (148/3492)
Rate ratio, from	0.66 SE 0.05	1.11 SE 0.12
(O−E)/V	−93.9/222.5	8.0/76.4

Breast cancer mortality

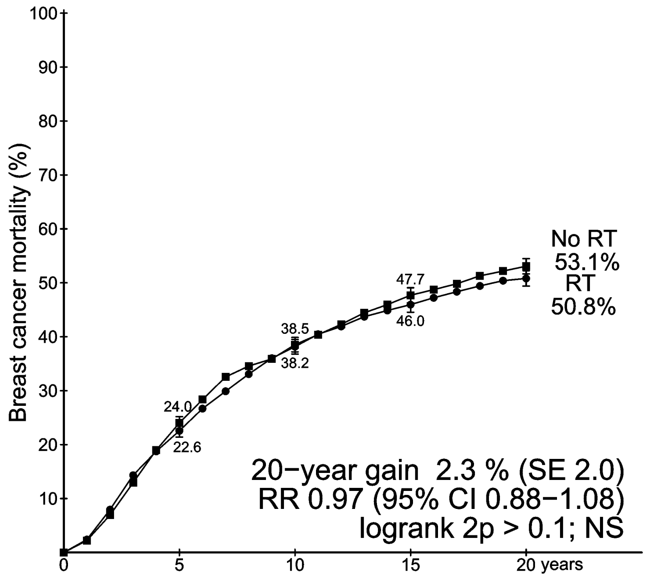

Breast cancer mortality rates (%/year) and logrank analyses

	Years 0−4	Years 5−9	Years 10−14	Years 15−19	Years 20+
RT	5.04 (308/6111)	4.51 (204/4523)	2.67 (93/3489)	1.87 (51/2733)	1.05 (54/5157)
No RT	5.37 (345/6428)	4.35 (203/4669)	3.28 (121/3688)	2.22 (63/2842)	0.98 (56/5686)
Rate ratio, from	0.94 SE 0.08	1.05 SE 0.11	0.84 SE 0.13	0.88 SE 0.18	1.16 SE 0.18
(O−E)/V	−8.6/151.6	4.9/94.3	−8.5/50.2	−3.6/27.2	5.3/35.6

Any death

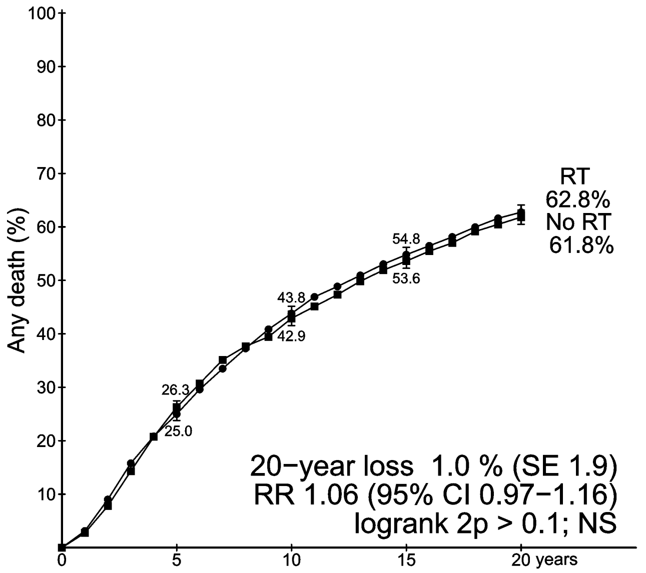

Any death rates (%/year) and logrank analyses

	Years 0−4	Years 5−9	Years 10−14	Years 15−19	Years 20+
RT	5.69 (348/6111)	5.77 (261/4523)	4.36 (152/3489)	3.84 (105/2733)	3.43 (177/5157)
No RT	5.94 (382/6428)	5.20 (243/4669)	4.28 (158/3688)	3.98 (113/2842)	2.80 (159/5686)
Rate ratio, from	0.96 SE 0.07	1.13 SE 0.10	1.05 SE 0.12	0.99 SE 0.14	1.26 SE 0.13
(O−E)/V	−7.5/170.3	14.5/117.3	3.8/72.3	−0.5/51.1	17.9/78.6

Webfigure 43. Effect of radiotherapy (RT) to the chest wall and regional lymph nodes versus not after mastectomy but no axillary surgery (Mast): 10-year risk of recurrence and type of first recurrence in 2896 women with clinically node-negative (cN-) disease. (r_L = number of women for whom first recurrence was locoregional, r_D = number women for whom distant recurrence was first.)

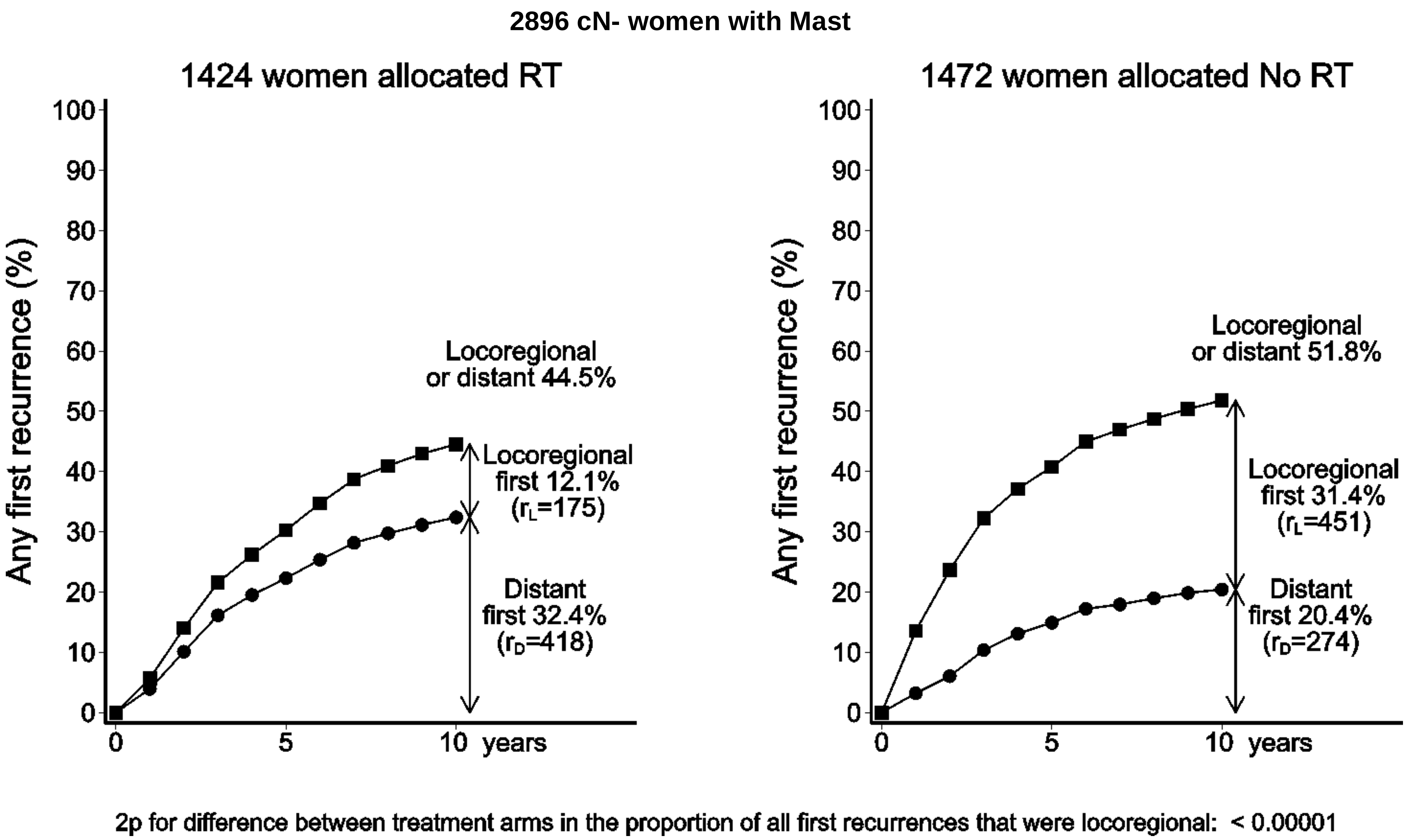

Webfigure 44. Effect of radiotherapy (RT) to the chest wall and regional lymph nodes versus not after mastectomy but no axillary surgery (Mast): 10-year risk of locoregional recurrence and recurrence of any type and 20-year risks of breast cancer and all-cause mortality in 1481 women with clinically node-positive (cN+) disease. See webfigure 1 for methodological note and also webfigure 45
.

1481 cN+ women with Mast

Locoregional recurrence first

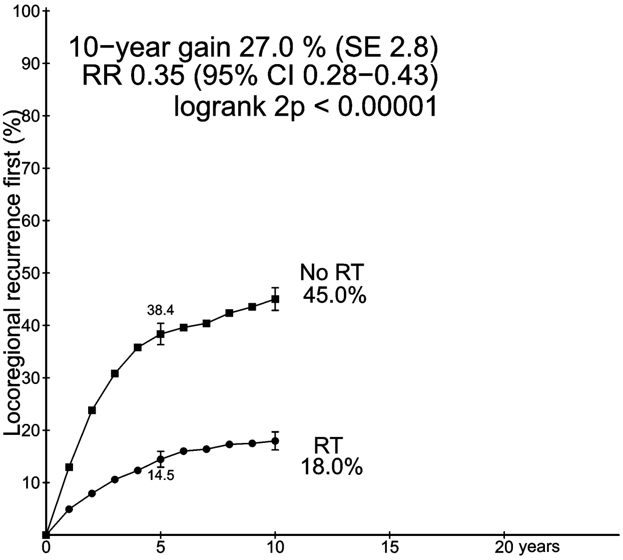

Locoregional recurrence first rates (%/year) and logrank analyses

	Years 0−4	Years 5−9
RT	3.55 (99/2787)	0.96 (17/1765)
No RT	10.55 (256/2425)	2.44 (35/1433)
Rate ratio, from	0.34 SE 0.07	0.40 SE 0.19
(O−E)/V	−87.5/81.0	−10.8/11.8

Any first recurrence

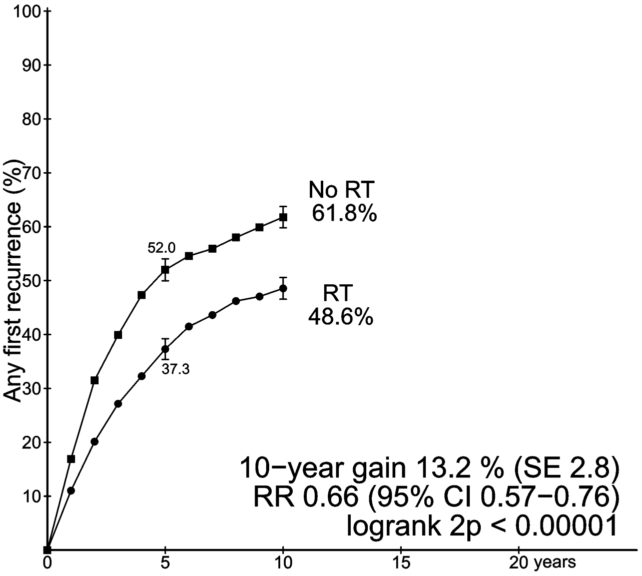

Any first recurrence rates (%/year) and logrank analyses

	Years 0−4	Years 5−9
RT	9.90 (277/2798)	4.16 (75/1802)
No RT	15.57 (378/2428)	4.63 (67/1448)
Rate ratio, from	0.61 SE 0.07	0.89 SE 0.17
(O−E)/V	−68.5/140.8	−3.7/32.2

Breast cancer mortality

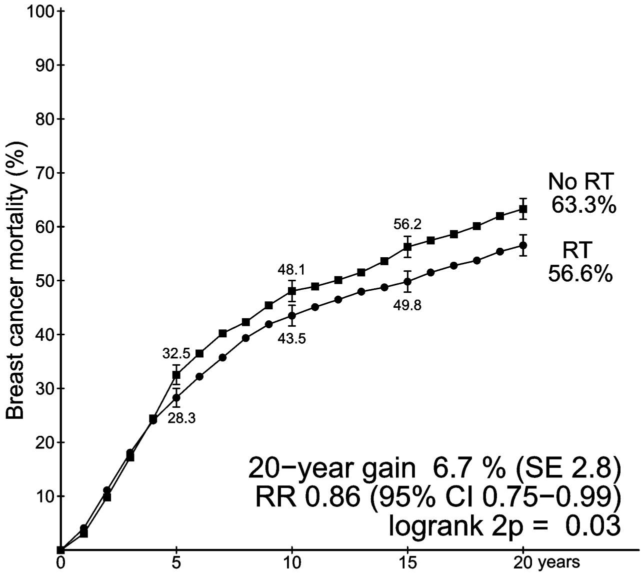

Breast cancer mortality rates (%/year) and logrank analyses

	Years 0−4	Years 5−9	Years 10−14	Years 15−19	Years 20+
RT	6.60 (206/3123)	4.79 (104/2169)	2.34 (40/1709)	2.76 (38/1379)	1.02 (28/2740)
No RT	7.54 (235/3116)	5.34 (110/2059)	3.41 (55/1611)	3.66 (45/1229)	1.23 (29/2358)
Rate ratio, from	0.87 SE 0.09	0.91 SE 0.14	0.72 SE 0.18	0.83 SE 0.21	0.89 SE 0.21
(O−E)/V	−13.8/99.7	−4.4/48.3	−7.1/21.9	−3.5/18.5	−2.4/20.1

Any death

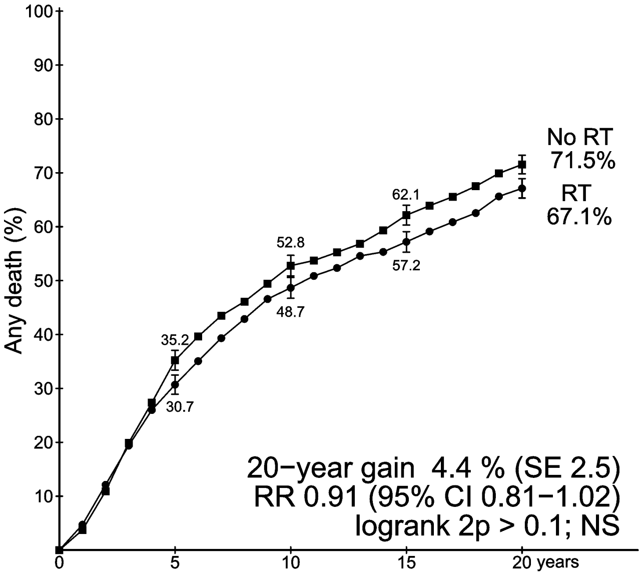

Any death rates (%/year) and logrank analyses

	Years 0−4	Years 5−9	Years 10−14	Years 15−19	Years 20+
RT	7.36 (230/3123)	6.22 (135/2169)	3.69 (63/1709)	5.29 (73/1379)	2.96 (81/2740)
No RT	8.34 (260/3116)	6.31 (130/2059)	4.41 (71/1611)	5.86 (72/1229)	3.10 (73/2358)
Rate ratio, from	0.86 SE 0.09	0.96 SE 0.13	0.84 SE 0.17	0.92 SE 0.17	1.04 SE 0.17
(O−E)/V	−16.4/111.7	−2.8/60.2	−5.3/30.9	−2.6/32.3	1.3/35.7

Webfigure 45. Effect of radiotherapy (RT) to the chest wall and regional lymph nodes versus not after mastectomy but no axillary surgery (Mast): 10-year risk of recurrence and type of first recurrence in 1481 women with clinically node-positive (cN+) disease. (r_L = number of women for whom first recurrence was locoregional, r_D = number women for whom distant recurrence was first.)

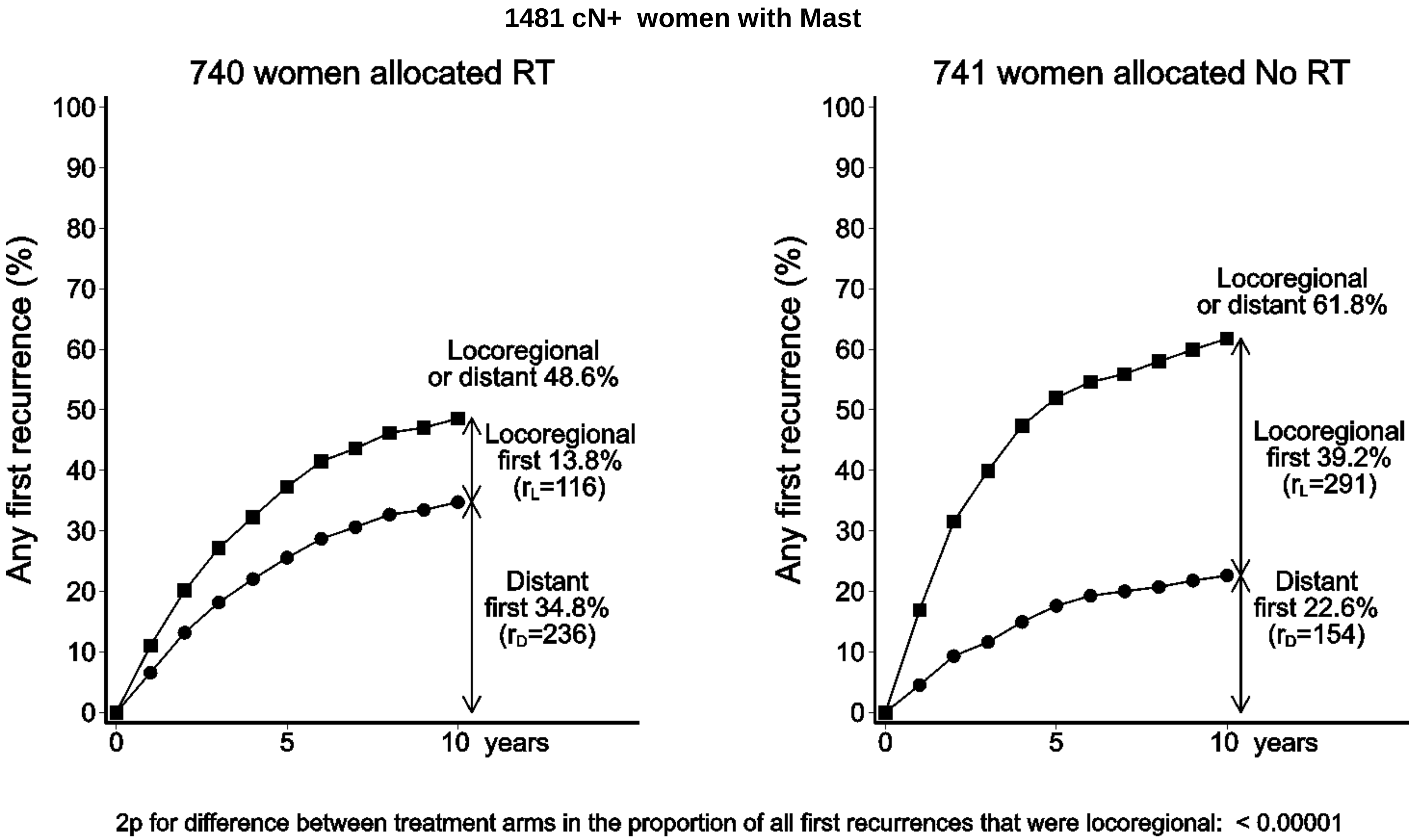

Webtable 6. **Availability of data from randomised trials beginning before the year 2000 and comparing radiotherapy to the regional lymph nodes alone versus not after mastectomy but no axillary surgery (Mast)*.**

Nodal status†	Women	Deaths	Woman-years since diagnosis					% women given systemic therapy‡		
			Median/ woman	Total (‘000s)	Distribution by years (‘000s) <10	10-	20+	Chemotherapy	ER+ & Tamoxifen	Any
**Axillary dissection**										
cN-	8	6	3.5	<0.1	<0.1			100	0	100
cN+	192	97	6.8	1.2	1.2	<0.1		100	0	100
**Total**	200	103	6.5	1.3	1.2	<0.1		100	0	100

*Data available for 2 trials, start dates 1985 to 1988. In all trials radiotherapy was given to the axilla/supraclavicular fossa and the internal mammary chain. Full details of the trials are given in webtable 7.

† cN-: negative clinical nodal status, cN+: positive clinical nodal status.

‡ Chemotherapy was cyclophosphamide, methotrexate, 5-fluorouracil [CMF].

Webtable 7. Randomised trials beginning before the year 2000 and comparing radiotherapy to the regional lymph nodes alone versus not after mastectomy but no axillary surgery (Mast) – treatment details.

Year code and study name	Breast surgery	Axillary dissection* (number of patients)	Chest wall RT	Supraclavicular and axillary fossa RT	Internal mammary chain RT	Boost RT to scar	Common systemic chemoendocrine therapy
85Z Tokyo CIH PS	EM	Axillary sampling (100)	None	42-48 Gy (2-3 Gy/f)	42-48 Gy (2-3 Gy/f)	None	CMF
88U Tokyo CIH CZ	EM	Axillary sampling (100)	None	42-48 Gy (2-3 Gy/f)	42-48 Gy (2-3 Gy/f)	None	CMF

*Based on the description of axillary surgery in the trial protocol or publications or on information on individual women. Women were classified as having axillary sampling if they were in a trial where the protocol specified no axillary dissection or, if individual information was available, resection of <10 nodes. In other trials, women were classified as having axillary sampling if the trial publication indicated that the median number of nodes removed was < 10, C=cyclophosphamide, EM=Extended mastectomy (ipsilateral parasternal and supraclavicular lymph node dissection), F=fluorouracil, f=fraction, Gy=Gray (intended dose), M=methotrexate, RT=radiotherapy.

References for Webtable 7

Year code and study name	Reference
85Z Tokyo CIH PS	Yamashita TH, Masahiko; Sekiguchi, Kenji; Kobayashi, Masao; Tanaka, Emiko; Uki, Akiyoshi; Kasumi, Fujio; Yoshimoto, Masataka. Efficacy of loco-regional lymphnodes irradiation after mastectomy for breast cancer with biopsy proven parasternal lymphnodes metastases — A randomized study. *Int J Radiat Oncol Biol Phys* 1996; **36**: 277.
88U Tokyo CIH CZ	Personal Correspondence from Dr M Yoshimoto

Webfigure 46. Effect of radiotherapy (RT) to the regional lymph nodes alone versus not after mastectomy but no axillary surgery (Mast): 10-year risks of recurrence, breast cancer and all-cause mortality in 192 clinically node-positive (cN+) women. See webfigure 1 for methodological note and also webfigure 47. Note, due to the very small number (8) of clinically node-negative women in this set of trials they are shown only in webfigure 48.

192 cN+ women with Mast

Locoregional recurrence first

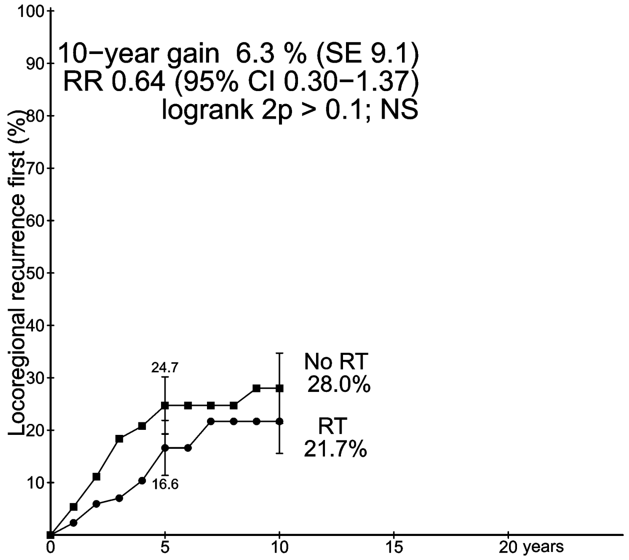

Locoregional recurrence first rates (%/year) and logrank analyses

	Years 0−4	Years 5−9
RT	3.60 (11/305)	1.46 (2/137)
No RT	5.54 (16/289)	1.64 (2/122)
Rate ratio, from	0.57 SE 0.31	1.86 SE 1.61
(O−E)/V	−3.4/6.1	0.5/0.7

Any first recurrence

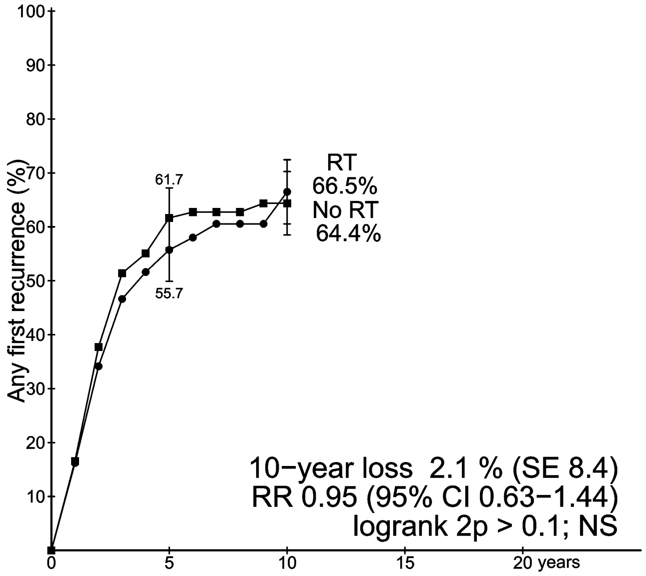

Any first recurrence rates (%/year) and logrank analyses

	Years 0−4	Years 5−9
RT	17.68 (54/305)	5.12 (7/137)
No RT	19.73 (57/289)	3.28 (4/122)
Rate ratio, from	0.86 SE 0.21	3.02 SE 1.35
(O−E)/V	−3.1/20.4	2.0/1.8

Breast cancer mortality

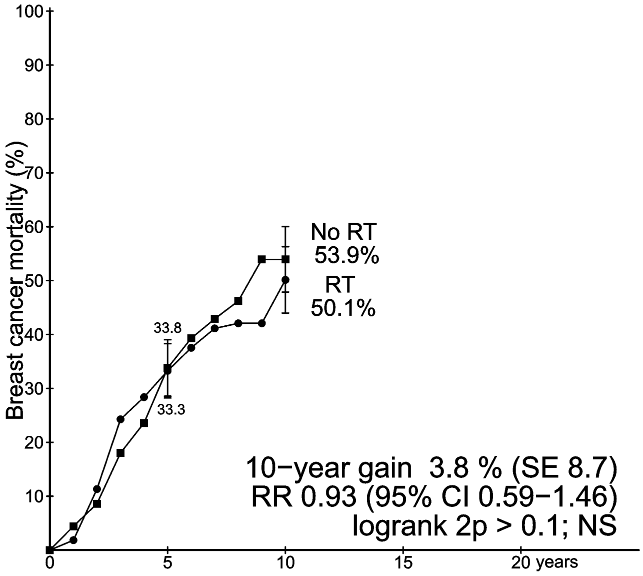

Breast cancer mortality rates (%/year) and logrank analyses

	Years 0−4	Years 5−9	Years 10+
RT	7.75 (31/400)	5.33 (11/206)	3.71 (1/27)
No RT	8.01 (32/399)	7.71 (15/195)	5.18 (1/19)
Rate ratio, from	1.01 SE 0.28	0.71 SE 0.36	2.10 SE 2.26
(O−E)/V	0.1/13.0	−1.8/5.4	0.3/0.4

Any death

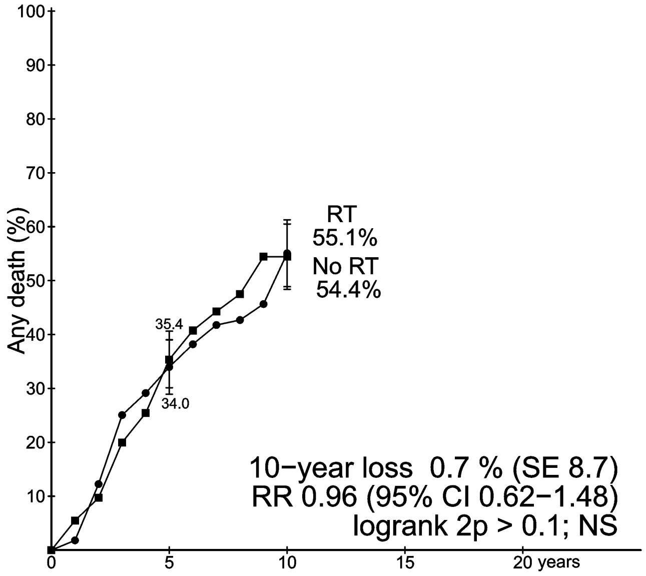

Any death rates (%/year) and logrank analyses

	Years 0−4	Years 5−9	Years 10+
RT	8.00 (32/400)	6.30 (13/206)	3.71 (1/27)
No RT	8.51 (34/399)	7.71 (15/195)	10.36 (2/19)
Rate ratio, from	0.98 SE 0.27	0.86 SE 0.38	2.10 SE 2.26
(O−E)/V	−0.3/13.8	−0.9/5.8	0.3/0.4

Webfigure 47. Effect of radiotherapy (RT) to the regional lymph nodes versus not after mastectomy but no axillary surgery (Mast): 10-year risk of recurrence and type of first recurrence in 192 women with clinically node-positive (cN+) disease. (r_L = number of women for whom first recurrence was locoregional, r_D = number women for whom distant recurrence was first.)

192 cN+ women with Mast

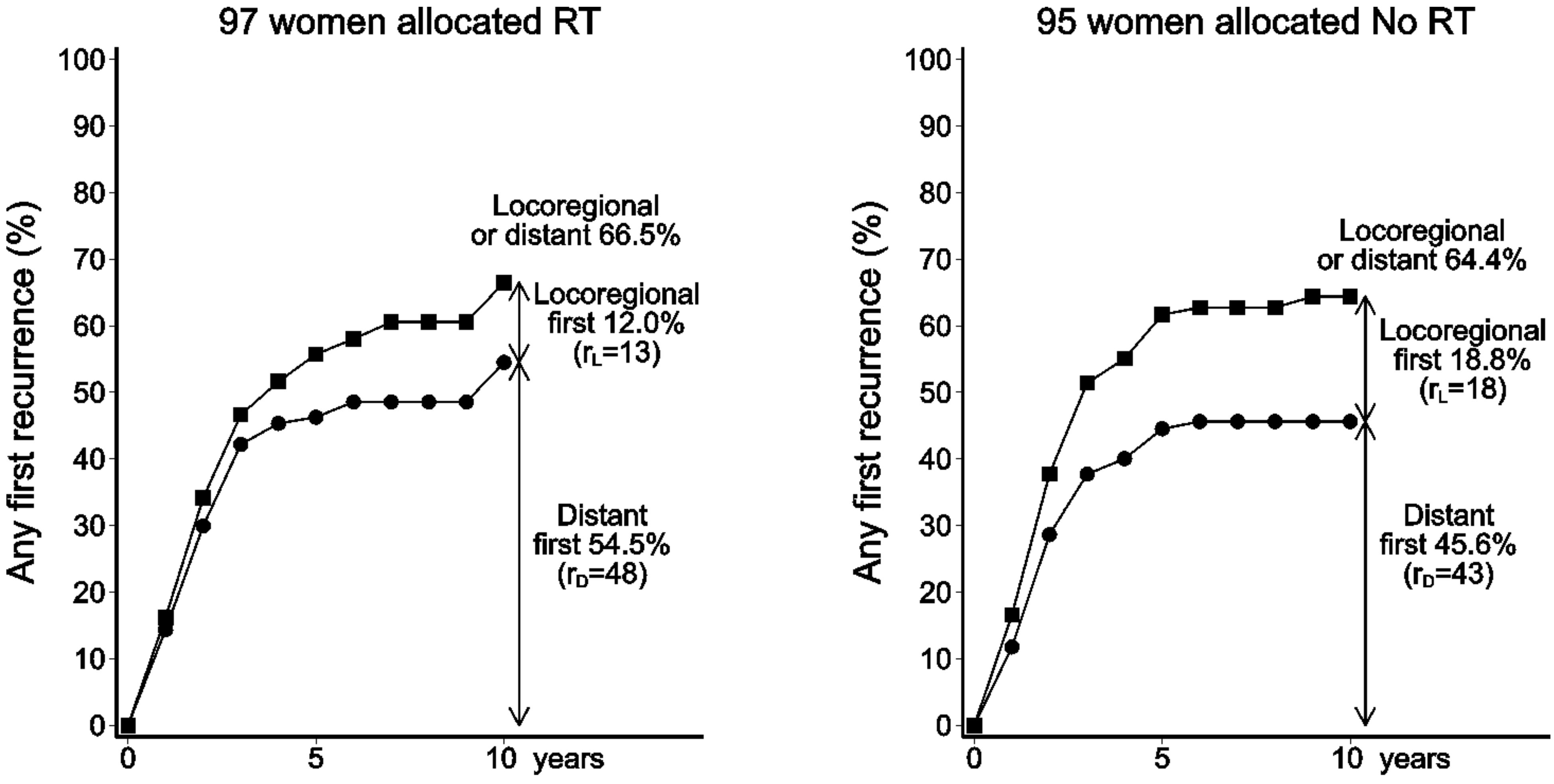

2p for difference between treatment arms in the proportion of all first recurrences that were locoregional: > 0.1; NS

Webfigure 48. Effect of radiotherapy (RT) versus not after mastectomy but no axillary surgery (Mast): 10 year risks of recurrence during years 0-9, breast cancer mortality, and all-cause mortality in 2904 women with clinically node-negative (cN-) disease. Event rate ratios, one line per trial, trial subdivided according to whether or not radiotherapy was given to the chest wall.

2904 cN- women

Locoregional recurrence first (years 0-9)

Year code, and study name	Treatment Information	Events/Women: Allocated RT	Events/Women: Allocated No RT	RT events: Logrank O−E	RT events: Variance of O−E
**(a) Mastectomy without axillary surgery but with CW radiotherapy**					
70B Kings/Cambridge	CW+AF+IMC	153/996	348/1049	−100.0	119.7
71C NSABP B−04	CW+AF+IMC	16/386	92/384	−40.1	24.4
78D Scottish D	†CW+AF+IMC	6/42	11/39	−2.9	3.8
**(a) Subtotal**		**175/ 1424 (12.3%)**	**451/ 1472 (30.6%)**	**−143.0**	**148.0**
**(b) Mastectomy without axillary surgery and no CW radiotherapy**					
85Z Tokyo CIH PS	†AF+IMC	0/3	1/5	−0.2	0.2
**(b) Subtotal**		**0/ 3 (0.0%)**	**1/ 5 (20.0%)**	**−0.2**	**0.2**
**Total**		**175/ 1427 (12.3%)**	**452/ 1477 (30.6%)**	**−143.2**	**148.2**

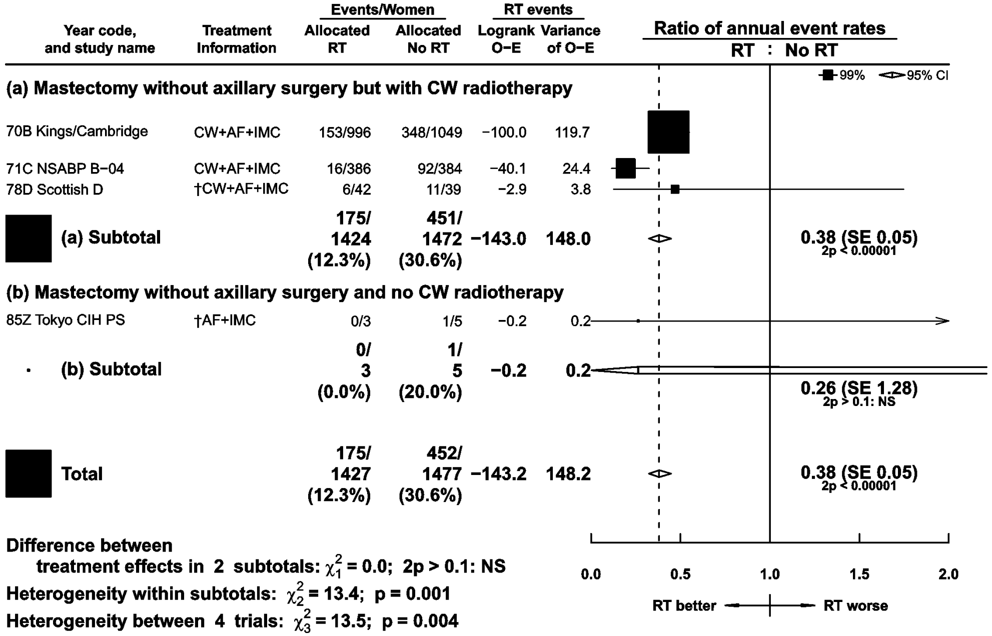

Difference between treatment effects in 2 subtotals: $\chi^2_1 = 0.0$; $2p > 0.1$: NS
Heterogeneity within subtotals: $\chi^2_2 = 13.4$; $p = 0.001$
Heterogeneity between 4 trials: $\chi^2_3 = 13.5$; $p = 0.004$

Any first recurrence (years 0-9)

Year code, and study name	Treatment Information	Events/Women: Allocated RT	Events/Women: Allocated No RT	RT events: Logrank O−E	RT events: Variance of O−E
**(a) Mastectomy without axillary surgery but with CW radiotherapy**					
70B Kings/Cambridge	CW+AF+IMC	435/996	532/1049	−55.7	222.2
71C NSABP B−04	CW+AF+IMC	139/386	178/384	−30.4	69.6
78D Scottish D	CW+AF+IMC	19/42	15/39	0.3	7.1
**(a) Subtotal**		**593/ 1424 (41.6%)**	**725/ 1472 (49.3%)**	**−85.9**	**298.9**
**(b) Mastectomy without axillary surgery and no CW radiotherapy**					
85Z Tokyo CIH PS	†AF+IMC	3/3	3/5	1.2	0.9
**(b) Subtotal**		**3/ 3 (100.0%)**	**3/ 5 (60.0%)**	**1.2**	**0.9**
**Total**		**596/ 1427 (41.8%)**	**728/ 1477 (49.3%)**	**−84.6**	**299.8**

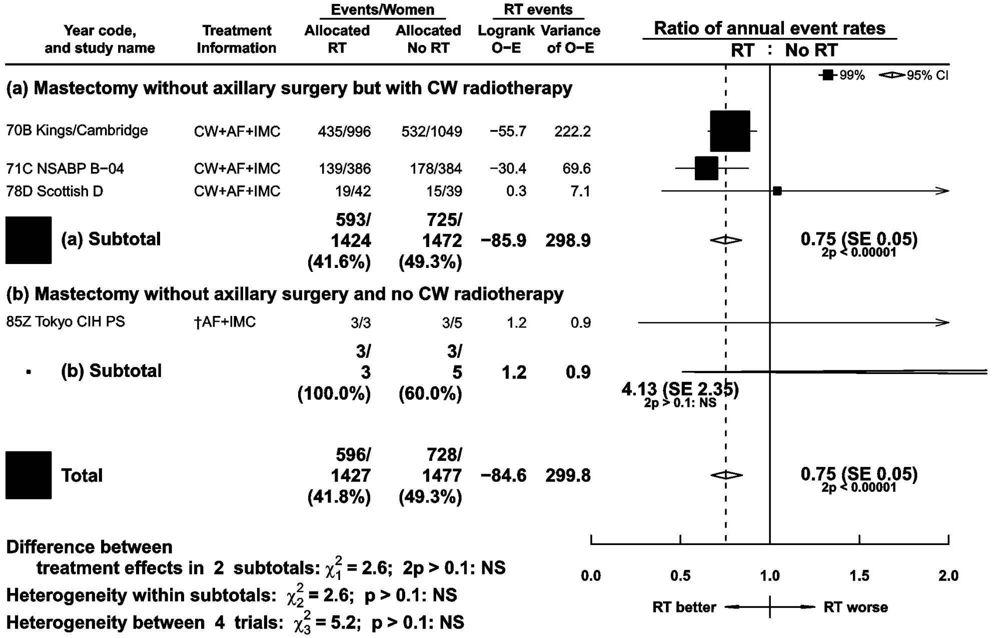

Difference between treatment effects in 2 subtotals: $\chi^2_1 = 2.6$; $2p > 0.1$: NS
Heterogeneity within subtotals: $\chi^2_2 = 2.6$; $p > 0.1$: NS
Heterogeneity between 4 trials: $\chi^2_3 = 5.2$; $p > 0.1$: NS

† Same polychemotherapy (cyclophosphamide, methotrexate, and 5-fluorouracil), and/or tamoxifen in both groups.
Radiotherapy sites: CW=chest wall, AF=Axilla and/or supraclavicular fossa, IMC=Internal mammary chain. Site(s) in brackets were not always treated.

continued overleaf

2904 cN- women

Breast cancer mortality

Year code, and study name	Treatment Information	Deaths/Women Allocated RT	Deaths/Women Allocated No RT	RT deaths Logrank O−E	RT deaths Variance of O−E	Ratio of annual death rates RT : No RT
**(a) Mastectomy without axillary surgery but with CW radiotherapy**						
70B Kings/Cambridge	CW+AF+IMC	523/996	590/1049	−3.7	270.0	
71C NSABP B−04	CW+AF+IMC	169/386	181/384	−6.5	81.3	
78D Scottish D	†CW+AF+IMC	18/42	17/39	−0.2	7.6	
**(a) Subtotal**		**710/ 1424 (49.9%)**	**788/ 1472 (53.5%)**	**−10.5**	**358.8**	**0.97 (SE 0.05)** 2p > 0.1: NS
**(b) Mastectomy without axillary surgery and no CW radiotherapy**						
85Z Tokyo CIH PS	†AF+IMC	3/3	3/5	0.9	0.9	
**(b) Subtotal**		**3/ 3 (100.0%)**	**3/ 5 (60.0%)**	**0.9**	**0.9**	**2.83 (SE 1.87)** 2p > 0.1: NS
**Total**		**713/ 1427 (50.0%)**	**791/ 1477 (53.6%)**	**−9.6**	**359.7**	**0.97 (SE 0.05)** 2p > 0.1: NS

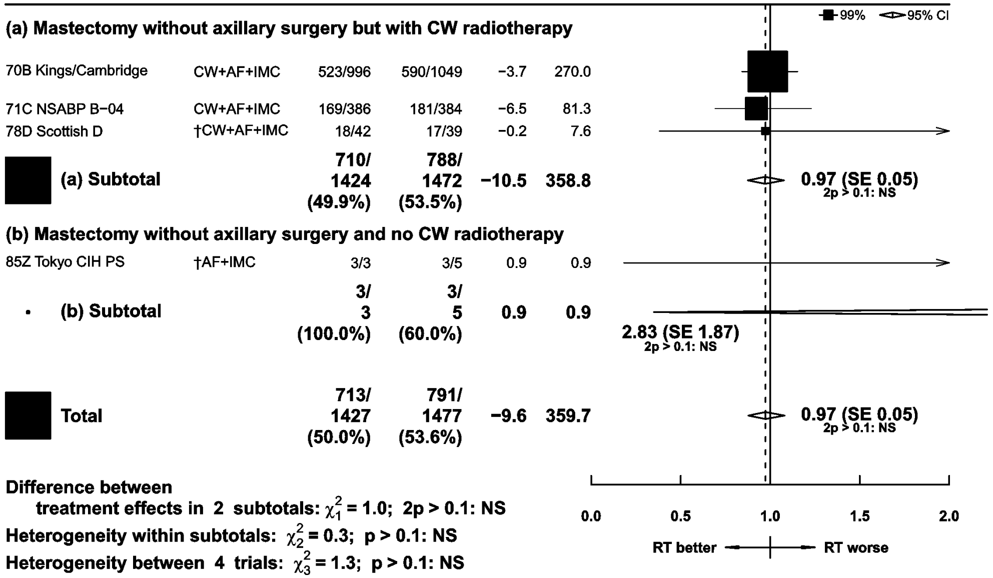

Difference between treatment effects in 2 subtotals: $\chi^2_1 = 1.0$; 2p > 0.1: NS
Heterogeneity within subtotals: $\chi^2_2 = 0.3$; p > 0.1: NS
Heterogeneity between 4 trials: $\chi^2_3 = 1.3$; p > 0.1: NS

Any death

Year code, and study name	Treatment Information	Deaths/Women Allocated RT	Deaths/Women Allocated No RT	RT deaths Logrank O−E	RT deaths Variance of O−E	Ratio of annual death rates RT : No RT
**(a) Mastectomy without axillary surgery but with CW radiotherapy**						
70B Kings/Cambridge	CW+AF+IMC	740/996	762/1049	15.3	355.4	
71C NSABP B−04	CW+AF+IMC	279/386	266/384	11.9	124.1	
78D Scottish D	†CW+AF+IMC	24/42	27/39	1.0	10.2	
**(a) Subtotal**		**1043/ 1424 (73.2%)**	**1055/ 1472 (71.7%)**	**28.2**	**489.6**	**1.06 (SE 0.05)** 2p > 0.1: NS
**(b) Mastectomy without axillary surgery and no CW radiotherapy**						
85Z Tokyo CIH PS	†AF+IMC	3/3	3/5	0.9	0.9	
**(b) Subtotal**		**3/ 3 (100.0%)**	**3/ 5 (60.0%)**	**0.9**	**0.9**	**2.83 (SE 1.87)** 2p > 0.1: NS
**Total**		**1046/ 1427 (73.3%)**	**1058/ 1477 (71.6%)**	**29.1**	**490.5**	**1.06 (SE 0.05)** 2p > 0.1: NS

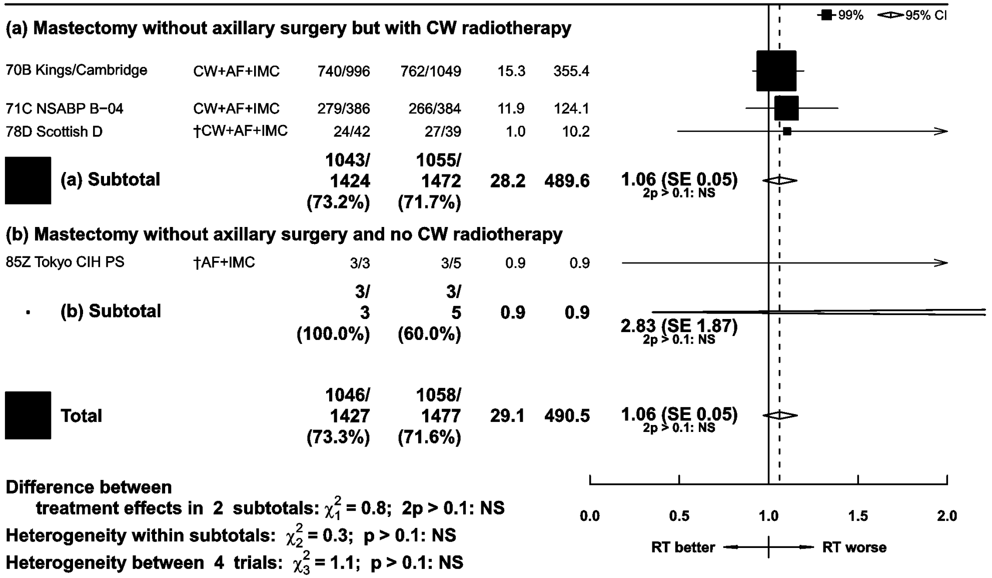

Difference between treatment effects in 2 subtotals: $\chi^2_1 = 0.8$; 2p > 0.1: NS
Heterogeneity within subtotals: $\chi^2_2 = 0.3$; p > 0.1: NS
Heterogeneity between 4 trials: $\chi^2_3 = 1.1$; p > 0.1: NS

† Same polychemotherapy (cyclophosphamide, methotrexate, and 5-fluorouracil), and/or tamoxifen in both groups.
Radiotherapy sites: CW=chest wall, AF=Axilla and/or supraclavicular fossa, IMC=Internal mammary chain. Site(s) in brackets were not always treated.

Webfigure 49. Effect of radiotherapy (RT) versus not after mastectomy but no axillary surgery (Mast): 10 year risks of recurrence during years 0-9, breast cancer mortality, and all-cause mortality in 1673 women with clinically node-positive (cN+) disease. Event rate ratios, one line per trial, trial subdivided according to whether or not radiotherapy was given to the chest wall.

1673 cN+ women

Locoregional recurrence first (years 0-9)

Year code, and study name	Treatment Information	Events/Women Allocated RT	Events/Women Allocated No RT	RT events Logrank O−E	RT events Variance of O−E	Ratio of annual event rates RT : No RT
**(a) Mastectomy without axillary surgery but with CW radiotherapy**						
70A Manchester RBS1	CW+AF+IMC	49/355	120/359	−39.7	39.5	
70B Kings/Cambridge	CW+AF+IMC	66/380	168/375	−58.7	53.4	
78D Scottish D	†CW+AF+IMC	1/5	3/7			
**(a) Subtotal**		**116/ 740 (15.7%)**	**291/ 741 (39.3%)**	**−98.3**	**92.9**	**0.35 (SE 0.06)** 2p < 0.00001
**(b) Mastectomy without axillary surgery and no CW radiotherapy**						
85Z Tokyo CIH PS	†AF+IMC	7/47	11/45	−2.3	3.7	
88U Tokyo CIH N2	†AF+IMC	6/50	7/50	−0.6	3.1	
**(b) Subtotal**		**13/ 97 (13.4%)**	**18/ 95 (18.9%)**	**−3.0**	**6.8**	**0.64 (SE 0.31)** 2p > 0.1: NS
**Total**		**129/ 837 (15.4%)**	**309/ 836 (37.0%)**	**−101.3**	**99.7**	**0.36 (SE 0.06)** 2p < 0.00001

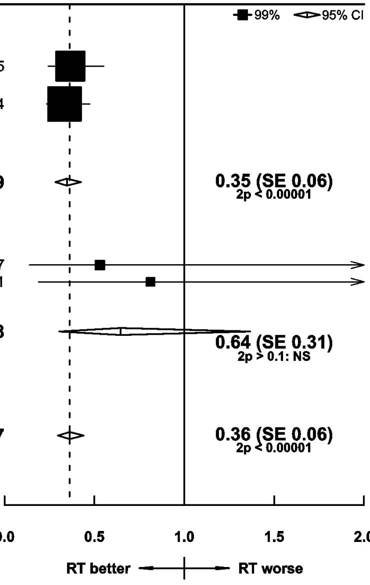

Difference between treatment effects in 2 subtotals: $\chi^2_1 = 2.4$; 2p > 0.1: NS
Heterogeneity within subtotals: $\chi^2_2 = 0.5$; p > 0.1: NS
Heterogeneity between 4 trials: $\chi^2_3 = 2.9$; p > 0.1: NS

Any first recurrence (years 0-9)

Year code, and study name	Treatment Information	Events/Women Allocated RT	Events/Women Allocated No RT	RT events Logrank O−E	RT events Variance of O−E	Ratio of annual event rates RT : No RT
**(a) Mastectomy without axillary surgery but with CW radiotherapy**						
70A Manchester RBS1	CW+AF+IMC	140/355	192/359	−36.1	74.3	
70B Kings/Cambridge	CW+AF+IMC	209/380	249/375	−36.7	98.5	
78D Scottish D	CW+AF+IMC	3/5	4/7	0.5	0.2	
**(a) Subtotal**		**352/ 740 (47.6%)**	**445/ 741 (60.1%)**	**−72.3**	**173.0**	**0.66 (SE 0.06)** 2p < 0.00001
**(b) Mastectomy without axillary surgery and no CW radiotherapy**						
85Z Tokyo CIH PS	†AF+IMC	27/47	26/45	1.3	9.8	
88U Tokyo CIH N2	†AF+IMC	34/50	35/50	−2.4	12.4	
**(b) Subtotal**		**61/ 97 (62.9%)**	**61/ 95 (64.2%)**	**−1.1**	**22.2**	**0.95 (SE 0.21)** 2p > 0.1: NS
**Total**		**413/ 837 (49.3%)**	**506/ 836 (60.5%)**	**−73.4**	**195.3**	**0.69 (SE 0.06)** 2p < 0.00001

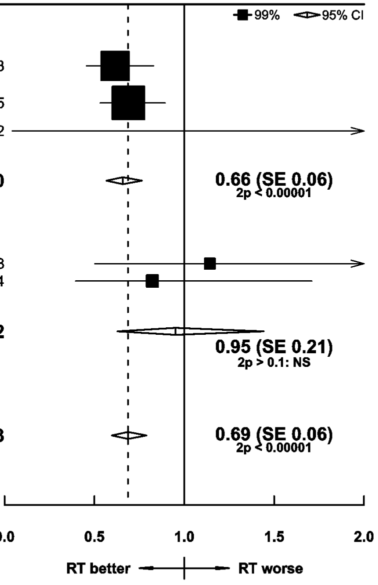

Difference between treatment effects in 2 subtotals: $\chi^2_1 = 2.7$; 2p > 0.1: NS
Heterogeneity within subtotals: $\chi^2_3 = 2.6$; p > 0.1: NS
Heterogeneity between 5 trials: $\chi^2_4 = 5.3$; p > 0.1: NS

† Same polychemotherapy (cyclophosphamide, methotrexate, and 5-fluorouracil), and/or tamoxifen in both groups.
Radiotherapy sites: CW=chest wall, AF=Axilla and/or supraclavicular fossa, IMC=Internal mammary chain. Site(s) in brackets were not always treated.

continued overleaf

1673 cN+ women

Breast cancer mortality

Year code, and study name	Treatment Information	Deaths/Women Allocated RT	Deaths/Women Allocated No RT	RT deaths Logrank O−E	RT deaths Variance of O−E	Ratio of annual death rates RT : No RT
**(a) Mastectomy without axillary surgery but with CW radiotherapy**						
70A Manchester RBS1	CW+AF+IMC	178/355	215/359	−14.5	93.7	
70B Kings/Cambridge	CW+AF+IMC	235/380	255/375	−17.3	114.6	
78D Scottish D	†CW+AF+IMC	3/5	4/7	0.5	0.2	
**(a) Subtotal**		**416/ 740 (56.2%)**	**474/ 741 (64.0%)**	**−31.2**	**208.6**	**0.86 (SE 0.06)** 2p = 0.03
**(b) Mastectomy without axillary surgery and no CW radiotherapy**						
85Z Tokyo CIH PS	†AF+IMC	18/47	21/45	−0.1	7.8	
88U Tokyo CIH N2	†AF+IMC	25/50	27/50	−1.2	11.1	
**(b) Subtotal**		**43/ 97 (44.3%)**	**48/ 95 (50.5%)**	**−1.4**	**18.9**	**0.93 (SE 0.22)** 2p > 0.1: NS
**Total**		**459/ 837 (54.8%)**	**522/ 836 (62.4%)**	**−32.6**	**227.5**	**0.87 (SE 0.06)** 2p = 0.03

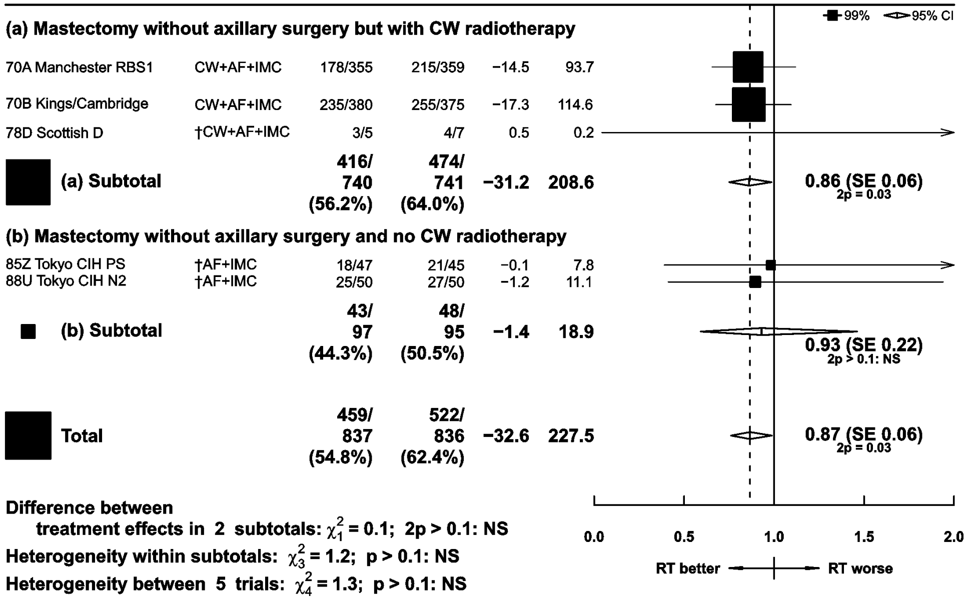

Difference between treatment effects in 2 subtotals: $\chi^2_1 = 0.1$; 2p > 0.1: NS
Heterogeneity within subtotals: $\chi^2_3 = 1.2$; p > 0.1: NS
Heterogeneity between 5 trials: $\chi^2_4 = 1.3$; p > 0.1: NS

Any death

Year code, and study name	Treatment Information	Deaths/Women Allocated RT	Deaths/Women Allocated No RT	RT deaths Logrank O−E	RT deaths Variance of O−E	Ratio of annual death rates RT : No RT
**(a) Mastectomy without axillary surgery but with CW radiotherapy**						
70A Manchester RBS1	CW+AF+IMC	274/355	286/359	−11.9	130.0	
70B Kings/Cambridge	CW+AF+IMC	303/380	316/375	−14.4	140.5	
78D Scottish D	†CW+AF+IMC	5/5	4/7	0.5	0.2	
**(a) Subtotal**		**582/ 740 (78.6%)**	**606/ 741 (81.8%)**	**−25.8**	**270.8**	**0.91 (SE 0.06)** 2p > 0.1: NS
**(b) Mastectomy without axillary surgery and no CW radiotherapy**						
85Z Tokyo CIH PS	†AF+IMC	20/47	23/45	0.4	8.5	
88U Tokyo CIH N2	†AF+IMC	26/50	28/50	−1.3	11.6	
**(b) Subtotal**		**46/ 97 (47.4%)**	**51/ 95 (53.7%)**	**−0.9**	**20.0**	**0.96 (SE 0.22)** 2p > 0.1: NS
**Total**		**628/ 837 (75.0%)**	**657/ 836 (78.6%)**	**−26.7**	**290.8**	**0.91 (SE 0.06)** 2p > 0.1: NS

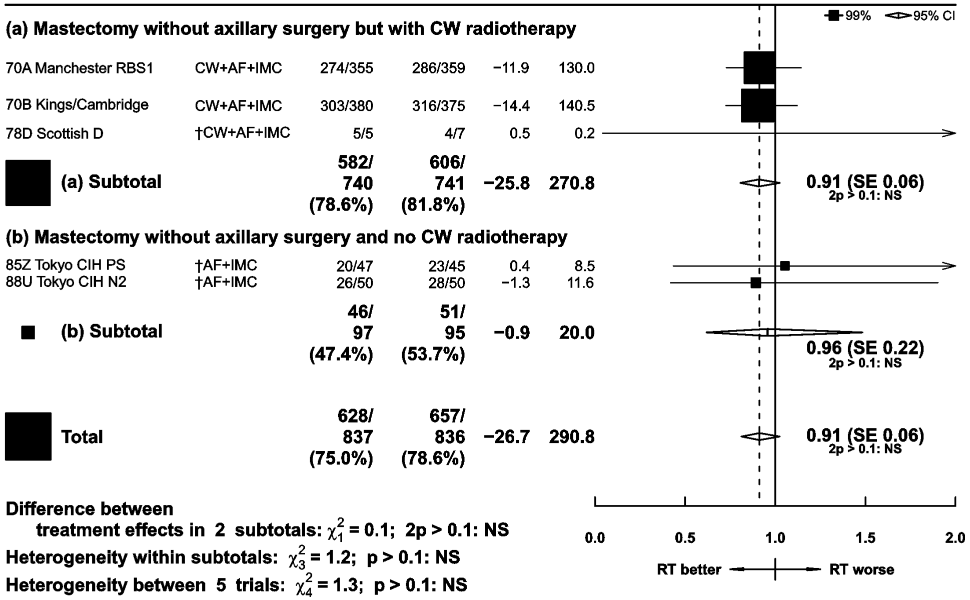

Difference between treatment effects in 2 subtotals: $\chi^2_1 = 0.1$; 2p > 0.1: NS
Heterogeneity within subtotals: $\chi^2_3 = 1.2$; p > 0.1: NS
Heterogeneity between 5 trials: $\chi^2_4 = 1.3$; p > 0.1: NS

† Same polychemotherapy (cyclophosphamide, methotrexate, and 5-fluorouracil), and/or tamoxifen in both groups.
Radiotherapy sites: CW=chest wall, AF=Axilla and/or supraclavicular fossa, IMC=Internal mammary chain. Site(s) in brackets were not always treated.

Webtable 8. **Availability of data from randomised trials beginning before the year 2000 and comparing radiotherapy to the chest wall and regional lymph nodes versus not before mastectomy and axillary dissection (Mast+AD) or axillary sampling (Mast+AS) *.**

Nodal status†	Women	Deaths	Woman-years since diagnosis					% women given systemic therapy	
			Median/ woman	Total ('000s)	Distribution by years ('000s) <10	10-	20+	Chemotherapy‡	ER+ & Tamoxifen
**Axillary dissection**									
**pN unknown**	**255**	**201**	**6.6**	**2.0**	**1.6**	**0.4**	**<0.1**	**0**	**0**
**Axillary sampling**									
**pN unknown**	**637**	**497**	**16.6**	**10.7**	**5.1**	**3.4**	**2.2**	**0**	**0**
**Total**	**892**	**698**	**12.1**	**12.7**	**6.7**	**3.8**	**2.2**	**0**	**0**

*Data available for 2 trials, start dates 1962 to 1971. In all trials radiotherapy was given to the axilla/supraclavicular fossa and the internal mammary chain.. Full details of the trials are given in webtable 9.

† pN unknown: as radiotherapy was given before surgery, to avoid bias pathological nodal status is regarded as unknown.

Webtable 9. Randomised trials beginning before the year 2000 and comparing radiotherapy to the chest wall and regional lymph nodes versus not before mastectomy and axillary dissection (Mast+AD) or axillary sampling (Mast+AS) – treatment details.

Year code and study name	Breast surgery	Axillary Surgery* (number of patients)	Chest wall RT	Supraclavicular and axillary fossa RT	Internal mammary chain RT	Boost RT to scar	Common systemic chemoendocrine therapy
62B Berlin-Bruch	RM	Axillary clearance (255)	55 Gy (u Gy/f) c	55 Gy (u Gy/f) c	55 Gy (u Gy/f) c	None	None
71B Stockholm A	MRM	Axillary sampling (637)	45 Gy (1.8 Gy/f) e	45 Gy de (1.8 Gy/f) c	45 Gy (1.8 Gy/f) e	None	None

* Based on the description of axillary surgery in the trial protocol or publications or on information on individual women. Women were classified as having axillary dissection if they were in a trial where the protocol required removal of axillary lymph nodes in at least Levels I & II or, if individual information was available, resection of ≥10 nodes. In other trials, women were classified as having axillary dissection if the trial publication indicated that the median number of nodes removed was ≥ 10. c=cobalt-60, e=electron, f=fraction, Gy=Gray (intended dose), MRM=modified radical mastectomy, RM=radical mastectomy (Halsted), RT=radiotherapy, u=unknown,

References for Webtable 9

Year code and study name	Reference
62B Berlin-Bruch	Berndt H, Eichhorn HJ, Widow W et al. Ein kontrollierter klinischer Versuch zur Zusatztherapie des operablen Brustdrusenkrebses mit Vorbestrahlung oder Cyclophosphamid. *Arch. Gesch* 1980; **50:** 168-479
71B Stockholm A	Gyenes G, Rutqvist LE, Liedberg A, Fornander T. Long-term cardiac morbidity and mortality in a randomized trial of pre- and postoperative radiation therapy versus surgery alone in primary breast cancer. *Radiother Oncol* 1998; **48**: 185–90.

Webfigure 50. Effect of radiotherapy (RT) to the chest wall and regional lymph nodes versus not before mastectomy and axillary dissection (Mast+AD): 10-year risk of locoregional recurrence and recurrence of any type and 15-year risk of breast cancer and all-cause mortality in 255 women with unknown pathological nodal status (pN?) disease. See webfigure 1 for methodological note and also webfigure 51.

255 pN? women with Mast+AD

Locoregional recurrence first

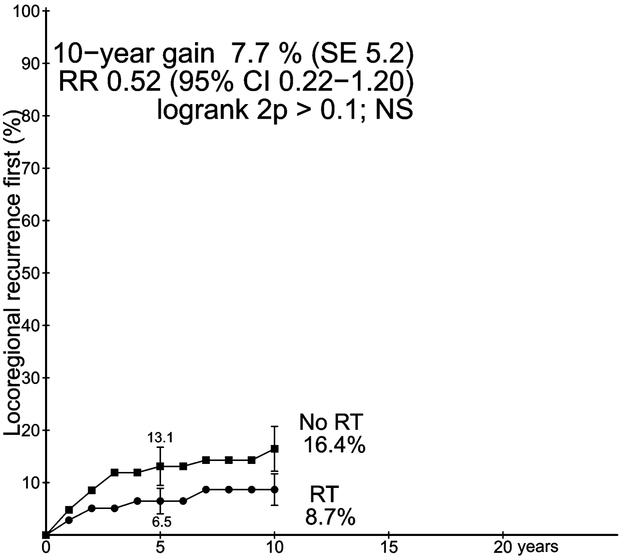

Locoregional recurrence first rates (%/year) and logrank analyses

	Years 0−4	Years 5−9
RT	1.43 (6/421)	0.43 (1/235)
No RT	3.37 (15/445)	0.76 (2/264)
Rate ratio, from	0.50 SE 0.33	0.65 SE 0.97
(O−E)/V	−3.3/4.7	−0.3/0.7

Any first recurrence

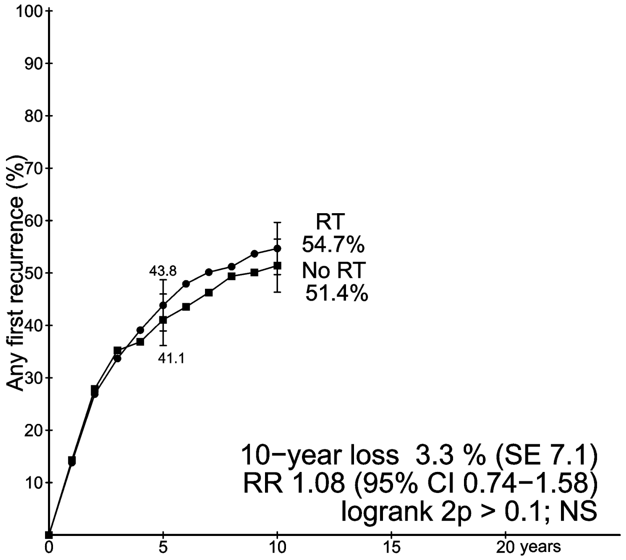

Any first recurrence rates (%/year) and logrank analyses

	Years 0−4	Years 5−9
RT	11.88 (50/421)	4.26 (10/235)
No RT	11.91 (53/445)	4.17 (11/264)
Rate ratio, from	1.07 SE 0.22	1.13 SE 0.48
(O−E)/V	1.4/21.8	0.6/4.9

Breast cancer mortality

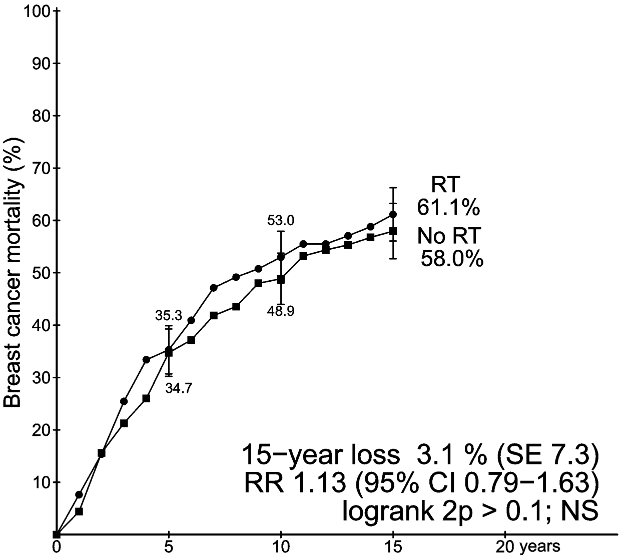

Breast cancer mortality rates (%/year) and logrank analyses

	Years 0−4	Years 5−9	Years 10−14	Years 15+
RT	8.56 (40/467)	6.58 (18/274)	3.21 (5/156)	9.47 (4/42)
No RT	8.52 (44/516)	5.14 (16/311)	4.57 (8/175)	3.17 (2/63)
Rate ratio, from	1.06 SE 0.24	1.43 SE 0.45	0.84 SE 0.56	1.52 SE 1.14
(O−E)/V	1.1/18.3	2.5/7.1	−0.5/2.7	0.5/1.2

Any death

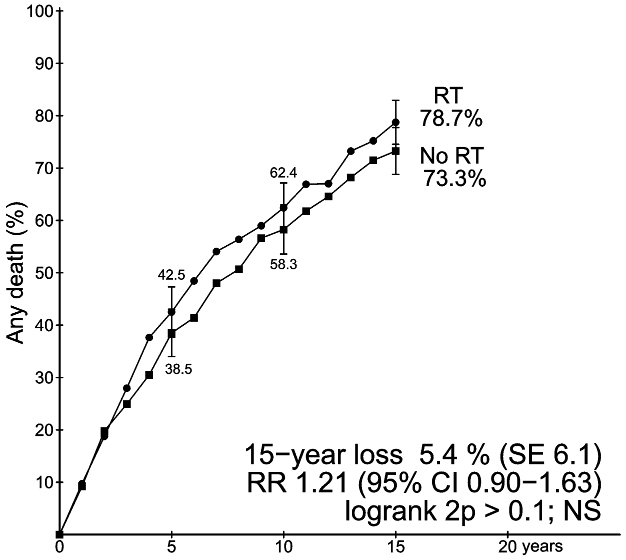

Any death rates (%/year) and logrank analyses

	Years 0−4	Years 5−9	Years 10−14	Years 15+
RT	11.13 (52/467)	9.14 (25/274)	11.54 (18/156)	23.66 (10/42)
No RT	9.88 (51/516)	8.03 (25/311)	9.13 (16/175)	6.34 (4/63)
Rate ratio, from	1.14 SE 0.22	1.13 SE 0.32	1.22 SE 0.41	2.70 SE 1.13
(O−E)/V	3.1/22.8	1.3/10.8	1.5/7.2	2.3/2.3

Webfigure 51. Effect of radiotherapy (RT) to the chest wall and regional lymph nodes versus not before mastectomy and axillary dissection (Mast+AD): 10-year risk of recurrence and type of first recurrence, by allocated treatment, in 255 women with unknown pathological nodal status (pN?). (r_L = number of women for whom first recurrence was locoregional, r_D = number women for whom distant recurrence was first.)

255 pN? women with Mast+AD

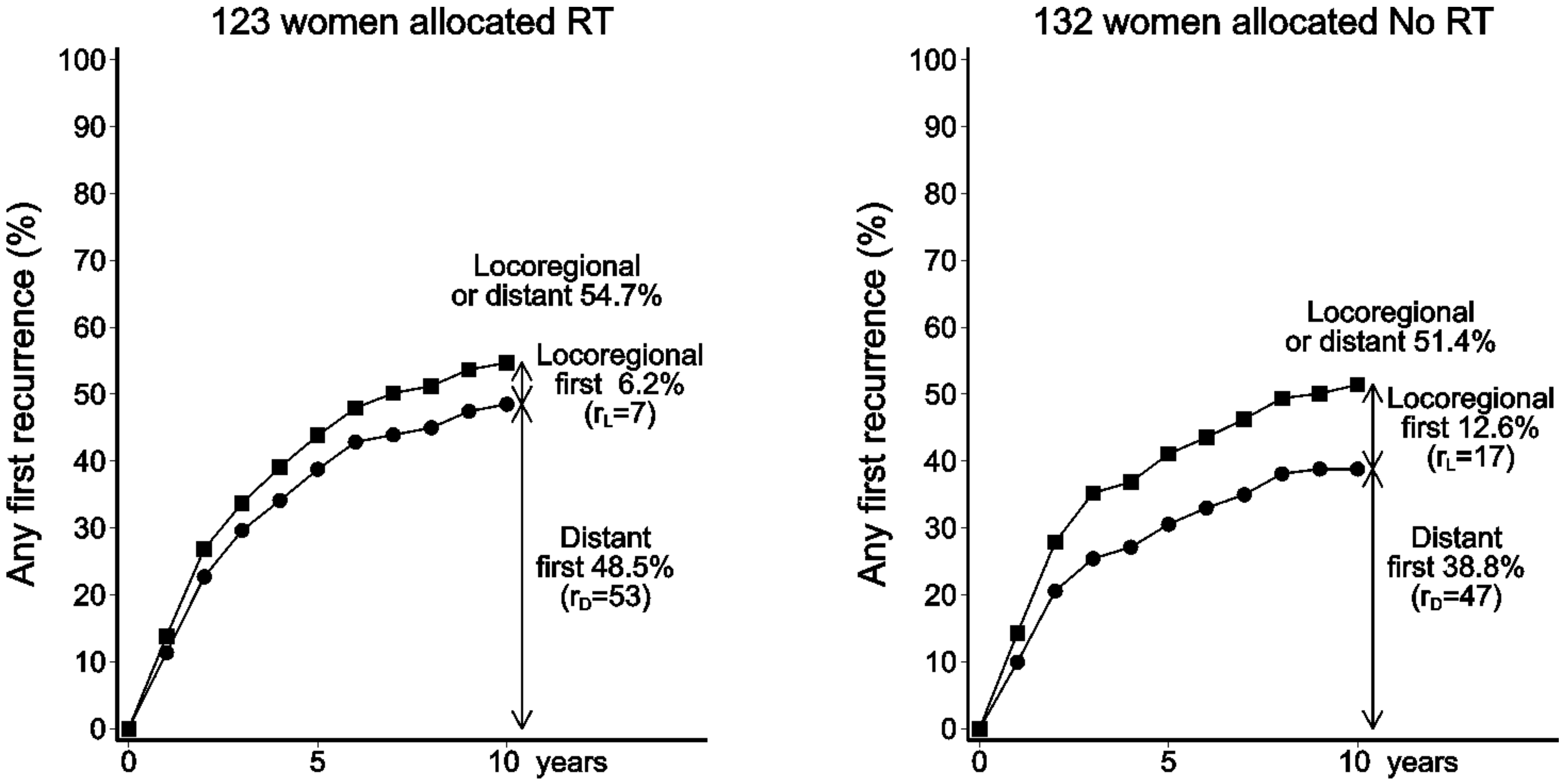

2p for difference between treatment arms in the proportion of all first recurrences that were locoregional: = 0.04

Webfigure 52. Effect of radiotherapy (RT) to the chest wall and regional lymph nodes versus not before mastectomy and axillary sampling (Mast+AS): 10-year risk of locoregional recurrence and recurrence of any type and 15-year risk of breast cancer and all-cause mortality in 637 women with unknown pathological nodal status (pN?) disease. See webfigure 1 for methodological note and also webfigure 53.

637 pN? women with Mast+AS

Locoregional recurrence first

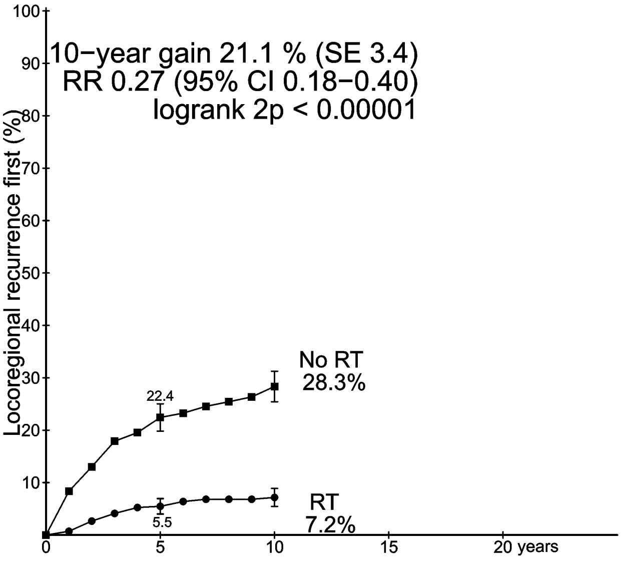

Locoregional recurrence first rates (%/year) and logrank analyses

	Years 0−4	Years 5−9
RT	1.24 (16/1290)	0.41 (4/968)
No RT	5.41 (63/1164)	1.61 (13/808)
Rate ratio, from	0.26 SE 0.13	0.28 SE 0.28
(O−E)/V	−25.2/18.9	−5.3/4.2

Any first recurrence

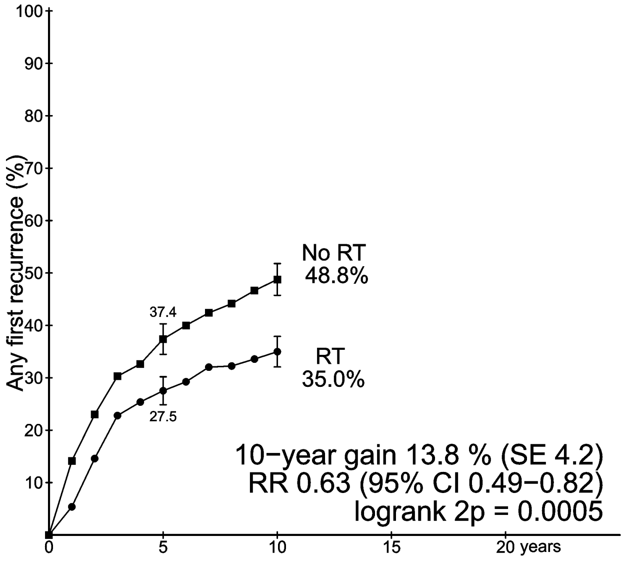

Any first recurrence rates (%/year) and logrank analyses

	Years 0−4	Years 5−9
RT	6.67 (86/1290)	2.48 (24/968)
No RT	9.88 (115/1164)	4.08 (33/808)
Rate ratio, from	0.66 SE 0.12	0.55 SE 0.21
(O−E)/V	−18.8/45.3	−7.8/13.2

Breast cancer mortality

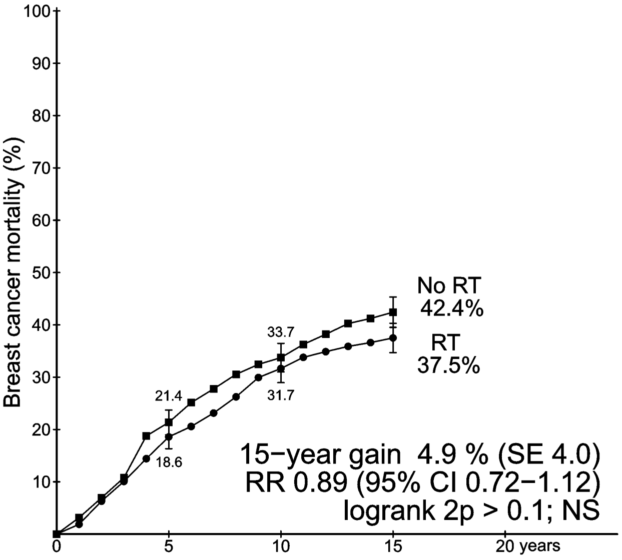

Breast cancer mortality rates (%/year) and logrank analyses

	Years 0−4	Years 5−9	Years 10−14	Years 15+
RT	4.10 (59/1440)	3.48 (40/1151)	1.70 (16/939)	1.89 (37/1962)
No RT	4.70 (67/1427)	3.48 (38/1090)	2.98 (27/907)	2.38 (43/1805)
Rate ratio, from	0.85 SE 0.17	1.00 SE 0.23	0.64 SE 0.25	1.05 SE 0.24
(O−E)/V	−4.9/29.7	−0.0/18.4	−4.6/10.3	0.9/18.8

Any death

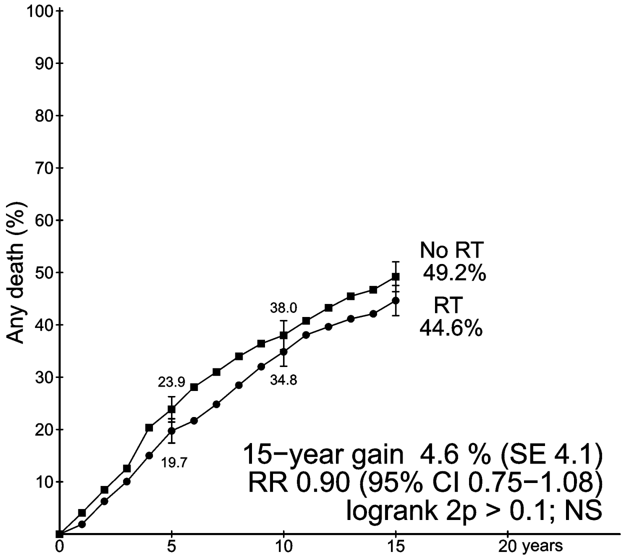

Any death rates (%/year) and logrank analyses

	Years 0−4	Years 5−9	Years 10−14	Years 15+
RT	4.38 (63/1440)	4.17 (48/1151)	3.41 (32/939)	5.15 (101/1962)
No RT	5.33 (76/1427)	4.13 (45/1090)	3.97 (36/907)	5.32 (96/1805)
Rate ratio, from	0.79 SE 0.16	0.99 SE 0.21	0.82 SE 0.23	0.97 SE 0.15
(O−E)/V	−7.6/32.9	−0.3/22.0	−3.2/15.9	−1.2/45.2

Webfigure 53. Effect of radiotherapy (RT) to the chest wall and regional lymph nodes versus not before mastectomy and axillary sampling (Mast+AS): 10-year risk of recurrence and type of first recurrence, by allocated treatment, in 637 women with unknown pathological nodal status (pN?). (r_L = number of women for whom first recurrence was locoregional, r_D = number women for whom distant recurrence was first.)

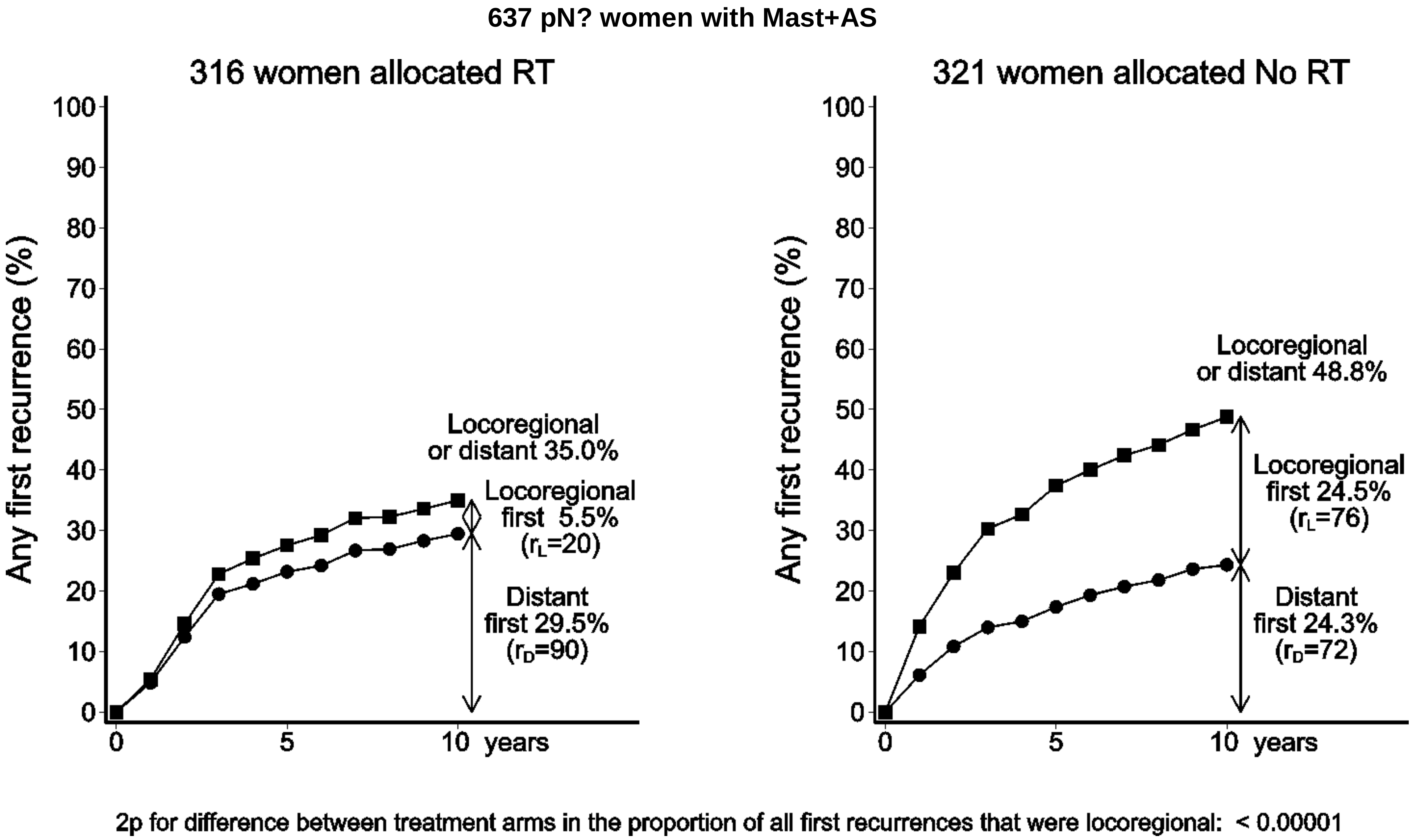

Webfigure 54. Effect of radiotherapy (RT) to the chest wall and regional lymph nodes versus not before mastectomy and axillary dissection (Mast+AD) or axillary sampling (Mast+AS): Event rate ratios, one line per trial, for locoregional recurrence and recurrence of any type during years 0-9 and for breast cancer and all-cause mortality in 892 women with unknown pathological nodal status (pN?).

892 pN? women

Locoregional recurrence first (years 0-9)

Year code, and study name	Treatment Information	Events/Women Allocated RT	Events/Women Allocated No RT	RT events Logrank O−E	RT events Variance of O−E
**(a) Axillary dissection**					
62B Berlin−Buch ABC	CW+AF+IMC	7/123	17/132	−3.6	5.4
**(a) Subtotal**		**7/ 123 (5.7%)**	**17/ 132 (12.9%)**	**−3.6**	**5.4**
**(b) Axillary sampling**					
71B Stockholm A	CW+AF+IMC	20/316	76/321	−30.5	23.1
**(b) Subtotal**		**20/ 316 (6.3%)**	**76/ 321 (23.7%)**	**−30.5**	**23.1**
**Total**		**27/ 439 (6.2%)**	**93/ 453 (20.5%)**	**−34.1**	**28.5**

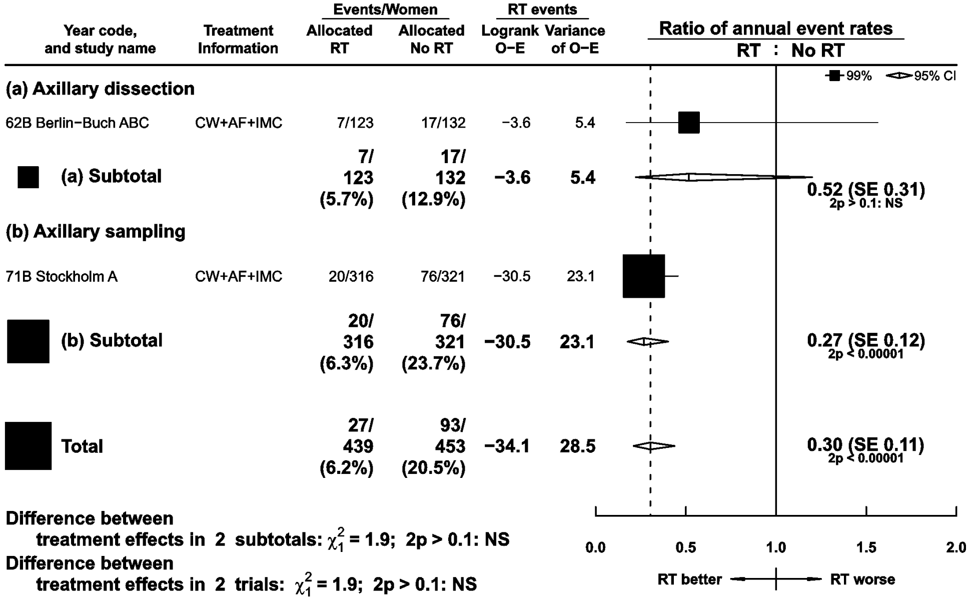

Difference between treatment effects in 2 subtotals: $\chi^2_1 = 1.9$; 2p > 0.1: NS
Difference between treatment effects in 2 trials: $\chi^2_1 = 1.9$; 2p > 0.1: NS

Any first recurrence (years 0-9)

Year code, and study name	Treatment Information	Events/Women Allocated RT	Events/Women Allocated No RT	RT events Logrank O−E	RT events Variance of O−E
**(a) Axillary dissection**					
62B Berlin−Buch ABC	CW+AF+IMC	60/123	64/132	2.0	26.8
**(a) Subtotal**		**60/ 123 (48.8%)**	**64/ 132 (48.5%)**	**2.0**	**26.8**
**(b) Axillary sampling**					
71B Stockholm A	CW+AF+IMC	110/316	148/321	−26.7	58.5
**(b) Subtotal**		**110/ 316 (34.8%)**	**148/ 321 (46.1%)**	**−26.7**	**58.5**
**Total**		**170/ 439 (38.7%)**	**212/ 453 (46.8%)**	**−24.6**	**85.2**

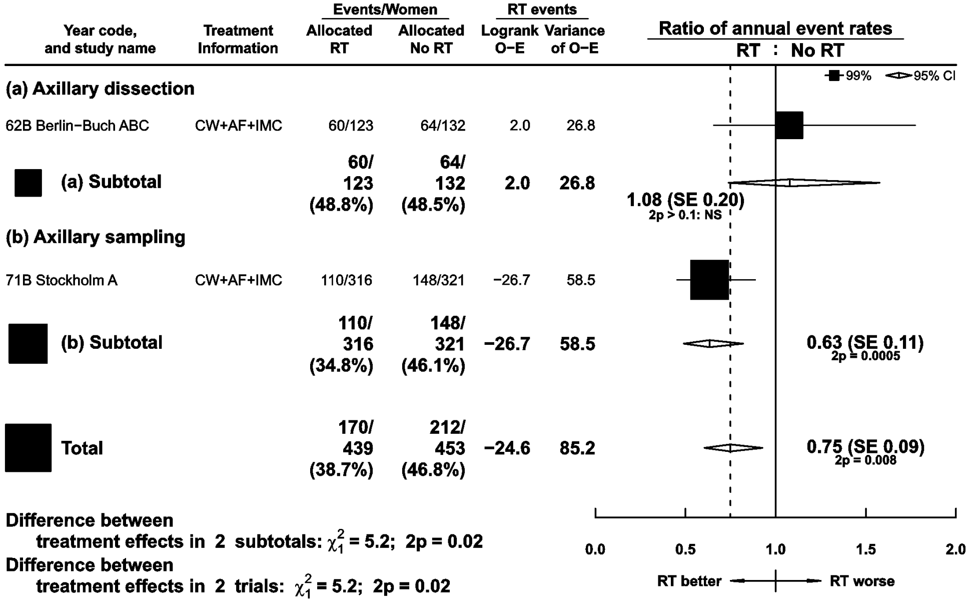

Difference between treatment effects in 2 subtotals: $\chi^2_1 = 5.2$; 2p = 0.02
Difference between treatment effects in 2 trials: $\chi^2_1 = 5.2$; 2p = 0.02

Radiotherapy sites: CW=chest wall, AF=Axilla and/or supraclavicular fossa, IMC=Internal mammary chain.

continued overleaf

892 pN? women

Breast cancer mortality

Year code, and study name	Treatment Information	Deaths/Women: Allocated RT	Deaths/Women: Allocated No RT	RT deaths: Logrank O−E	RT deaths: Variance of O−E	Ratio of annual death rates RT : No RT
**(a) Axillary dissection**						
62B Berlin−Buch ABC	CW+AF+IMC	67/123	70/132	3.6	29.3	
**(a) Subtotal**		**67/ 123 (54.5%)**	**70/ 132 (53.0%)**	**3.6**	**29.3**	**1.13 (SE 0.20)** 2p > 0.1: NS
**(b) Axillary sampling**						
71B Stockholm A	CW+AF+IMC	152/316	175/321	−8.6	77.1	
**(b) Subtotal**		**152/ 316 (48.1%)**	**175/ 321 (54.5%)**	**−8.6**	**77.1**	**0.89 (SE 0.11)** 2p > 0.1: NS
**Total**		**219/ 439 (49.9%)**	**245/ 453 (54.1%)**	**−4.9**	**106.4**	**0.95 (SE 0.09)** 2p > 0.1: NS

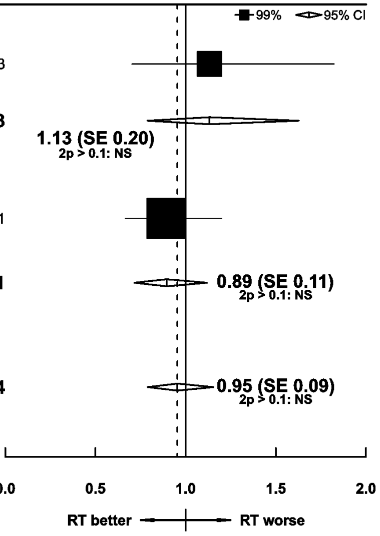

Difference between treatment effects in 2 subtotals: $\chi^2_1 = 1.2$; 2p > 0.1: NS
Difference between treatment effects in 2 trials: $\chi^2_1 = 1.2$; 2p > 0.1: NS

Any death

Year code, and study name	Treatment Information	Deaths/Women: Allocated RT	Deaths/Women: Allocated No RT	RT deaths: Logrank O−E	RT deaths: Variance of O−E	Ratio of annual death rates RT : No RT
**(a) Axillary dissection**						
62B Berlin−Buch ABC	CW+AF+IMC	105/123	96/132	8.1	43.1	
**(a) Subtotal**		**105/ 123 (85.4%)**	**96/ 132 (72.7%)**	**8.1**	**43.1**	**1.21 (SE 0.17)** 2p > 0.1: NS
**(b) Axillary sampling**						
71B Stockholm A	CW+AF+IMC	244/316	253/321	−12.3	116.1	
**(b) Subtotal**		**244/ 316 (77.2%)**	**253/ 321 (78.8%)**	**−12.3**	**116.1**	**0.90 (SE 0.09)** 2p > 0.1: NS
**Total**		**349/ 439 (79.5%)**	**349/ 453 (77.0%)**	**−4.1**	**159.2**	**0.97 (SE 0.08)** 2p > 0.1: NS

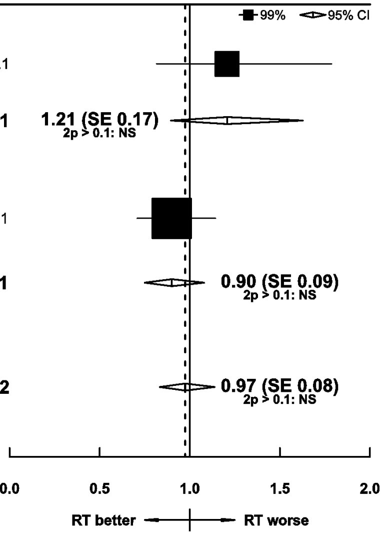

Difference between treatment effects in 2 subtotals: $\chi^2_1 = 2.7$; 2p = 0.10
Difference between treatment effects in 2 trials: $\chi^2_1 = 2.7$; 2p = 0.10

Radiotherapy sites: CW=chest wall, AF=Axilla and/or supraclavicular fossa, IMC=Internal mammary chain.

Webfigure 55. EBCTCG collaborators, listed alphabetically by institution and then name

ACETBC, Tokyo, Japan—O Abe, R Abe, K Enomoto, K Kikuchi, H Koyama, H Masuda, Y Nomura, Y Ohashi, K Sakai, K Sugimachi, M Toi, T Tominaga, J Uchino, M Yoshida.
Addenbrooke's Hospital, Cambridge, UK—J L Haybittle.
Anglo-Celtic Cooperative Oncology Group, UK—C F Leonard.
ARCOSEIN Group, France—G Calais, P Geraud.
ATLAS Trial Collaborative Study Group, Oxford, UK—V Collett, C Davies, A Delmestri, J Sayer.
Auckland Breast Cancer Study Group, New Zealand—V J Harvey, I M Holdaway, R G Kay, B H Mason.
Australian New Zealand Breast Cancer Trials Group, Sydney, Australia—J F Forbes, N Wilcken.
Austrian Breast Cancer Study Group, Vienna, Austria—R Bartsch, P Dubsky, C Fesl, H Fohler, M Gnant, R Greil, R Jakesz, A Lang, G Luschin-Ebengreuth, C Marth, B Mlineritsch, H Samonigg, C F Singer, G G Steger, H Stöger.
Beatson Oncology Centre, Glasgow, UK—P Canney, H M A Yosef.
Belgian Adjuvant Breast Cancer Project, Liège, Belgium—C Focan.
Berlin-Buch Akademie der Wissenschaften, Germany—U Peek.
Birmingham General Hospital, UK—G D Oates, J Powell.
Bordeaux Institut Bergonié, France—M Durand, L Mauriac.
Bordet Institute, Brussels, Belgium—A Di Leo, S Dolci, D Larsimont, J M Nogaret, C Philippson, M J Piccart.
Bradford Royal Infirmary, UK—M B Masood, D Parker, J J Price.
Breast Cancer International Research Group (BCIRG)—M A Lindsay, J Mackey, M Martin.
Breast Cancer Study Group of the Comprehensive Cancer Centre, Limburg, Netherlands—P S G J Hupperets.
British Association of Surgical Oncology BASO II Trialists, London, UK—T Bates, R W Blamey, U Chetty, I O Ellis, E Mallon, D A L Morgan, J Patnick, S Pinder.
British Columbia Cancer Agency, Vancouver, Canada—I Olivotto, J Ragaz.
Cancer and Leukemia Group B, Washington DC, USA—D Berry, G Broadwater, C Cirrincione, H Muss, L Norton, R B Weiss.
Cancer Care Ontario, Canada—H T Abu-Zahra.
Cancer Research Centre of the Russian Academy of Medical Sciences, Moscow, Russia—S M Portnoj.
Cancer Research UK Clinical Trials Unit (CRCTU), NCRI, Birmingham, UK—S Bowden, C Brookes, J Dunn, I Fernando, M Lee, C Poole, D Rea, D Spooner.
Cardiff Trialists Group, UK—P J Barrett-Lee, R E Mansel, I J Monypenny.
Case Western Reserve University, Cleveland, OH, USA—N H Gordon.
Central Oncology Group, Milwaukee, WI, USA—H L Davis.
Centre for Cancer Prevention, Wolfson Institute of Preventive Medicine, Queen Mary, University of London, UK—J Cuzick.
Centre Léon-Bérard, Lyon, France—Y Lehingue, P Romestaing.
Centre Paul Lamarque, Montpellier, France—J B Dubois.
Centre Regional François Baclesse, Caen, France—T Delozier, B Griffon, J Mace Lesech.
Centre René Huguenin, Paris, St Cloud, France— E Brain, B de La Lande, E Mouret-Fourme
Centro Oncologico, Trieste, Italy—G Mustacchi.
Charles University in Prague, First Faculty of Medicine, Department of Oncology of the First Faculty of Medicine and General Teaching Hospital, Czech Republic—L Petruzelka, O Pribylova.
Cheltenham General Hospital, UK—J R Owen.
Chemo N0 Trial Group, Germany—N Harbeck, F Jänicke, C Meisner, M Schmitt, C Thomssen.
Chicago University, IL, USA—P Meier.
Chinese Academy of Medical Sciences, Beijing, People's Republic of China (in collaboration with the Oxford CTSU)—Y Shan, Y F Shao, X Wang, D B Zhao (CTSU: Z M Chen, H C Pan).
Christie Hospital and Holt Radium Institute, Manchester, UK—A Howell, R Swindell.
Clinical Trial Service Unit, Oxford, UK (ie, EBCTCG Secretariat)—J A Burrett, M Clarke, R Collins, C Correa, D Cutter, S Darby, C Davies, K Davies, A Delmestri, P Elphinstone, V Evans, L Gettins, J Godwin, R Gray, C Gregory, D Hermans, C Hicks, S James, A Kerr, H Liu, E MacKinnon, M Lay, P McGale, T McHugh, P Morris, H C Pan, R Peto, J Sayer, C Taylor, Y Wang.
Coimbra Instituto de Oncologia, Portugal—J Albano, C F de Oliveira, H Gervásio, J Gordilho.
Copenhagen Breast Cancer Trials, Copenhagen, Denmark—B Ejlertsen, M-B Jensen, H Johansen, H Mouridsen, T Palshof.
Dana-Farber Cancer Institute, Boston, MA, USA—R S Gelman, J R Harris, D Hayes, C Henderson, C L Shapiro, E Winer.
Danish Breast Cancer Cooperative Group, Copenhagen, Denmark— P Christiansen, B Ejlertsen, M

Ewertz, M-B Jensen, S Møller, H T Mouridsen.
Düsseldorf University, Germany—H J Trampisch.
Dutch Working Party for Autologous Bone Marrow Transplant in Solid Tumours, Amsterdam & Groningen, Netherlands—O Dalesio, E G E de Vries, S Rodenhuis, H van Tinteren.
Eastern Cooperative Oncology Group, Boston, MA, USA—R L Comis, N E Davidson, R Gray, N Robert, G Sledge, L J Solin, J A Sparano, D C Tormey, W Wood.
Edinburgh Breast Unit, UK—D Cameron, U Chetty, J M Dixon, P Forrest, W Jack, I Kunkler.
Elim Hospital, Hamburg, Germany—J Rossbach.
Erasmus MC/Daniel den Hoed Cancer Center, Rotterdam, Netherlands— J G M Klijn, A D Treurniet-Donker, W L J van Putten.
European Institute of Oncology, Milan, Italy—N Rotmensz, U Veronesi, G Viale.
European Organization for Research and Treatment of Cancer, Brussels, Belgium—H Bartelink, N Bijker, J Bogaerts, F Cardoso, T Cufer, J P Julien, E Rutgers, C J H van de Velde.
Evanston Hospital, IL, USA—M P Cunningham.
Finnish Breast Cancer Group, Finland—R Huovinen, H Joensuu.
Fondazione Maugeri Pavia, Italy—A Costa.
Fondazione Michelangelo, Milan, Italy—G Bonadonna, L Gianni, P Valagussa.
Fox Chase Cancer Center, Philadelphia, PA, USA—L J Goldstein.
French Adjuvant Study Group (GFEA), Guyancourt, France—J Bonneterre, P Fargeot, P Fumoleau, P Kerbrat, E Luporsi, M Namer.
German Adjuvant Breast Group (GABG), Frankfurt, Germany—W Eiermann, J Hilfrich, W Jonat, M Kaufmann, R Kreienberg, M Schumacher.
German Breast Cancer Study Group (BMFT), Freiburg, Germany—G Bastert, H Rauschecker, R Sauer, W Sauerbrei, A Schauer, M Schumacher.
German Breast Group (GBG), Neu-Isenburg, Germany—J U Blohmer, S D Costa, H Eidtmann, B Gerber, C Jackisch, S Loibl, G von Minckwitz.
Ghent University Hospital, Belgium—A de Schryver, L Vakaet.
GIVIO Interdisciplinary Group for Cancer Care Evaluation, Chieti, Italy—M Belfiglio, A Nicolucci, F Pellegrini, M C Pirozzoli, M Sacco, M Valentini.
Glasgow Victoria Infirmary, UK—C S McArdle, D C Smith, S Stallard.
Groote Schuur Hospital, Cape Town, South Africa—D M Dent, C A Gudgeon, A Hacking, E Murray, E Panieri, ID Werner.
Grupo Español de Investigación en Cáncer de Mama (GEICAM), Spain—E Carrasco, M Martin, M A Segui.
Gruppo Oncologico Clinico Cooperativo del Nord Est, Aviano, Italy—E Galligioni.
Gruppo Oncologico Dell'Italia Meridionale (GOIM), Rome, Italy—M Lopez.
Guadalajara Hospital de 20 Noviembre, Mexico—A Erazo, J Y Medina.
Gunma University, Japan—J Horiguchi, H Takei.
Guy's Hospital, London, UK—I S Fentiman, J L Hayward, R D Rubens, D Skilton.
Heidelberg University I, Germany—H Scheurlen.
Heidelberg University II, Germany—M Kaufmann, H C Sohn.
Helios Klinikum Berlin-Buch, Germany—M Untch.
Hellenic Breast Surgeons Society, Greece—U Dafni, C Markopoulos.
Hellenic Cooperative Oncology Group, Athens, Greece—U Dafni, G Fountzilas.
Hellenic Oncology Research Group, Greece—D Mavroudis.
Helsinki Deaconess Medical Centre, Finland—P Klefstrom.
Helsinki University, Finland—C Blomqvist, T Saarto.
Hospital del Mar, Barcelona, Spain—M Gallen.
Humanitas Cancer Center, Milan, Italy—C Tinterri.
Innsbruck University, Austria—R Margreiter.
Institut Claudius Regaud, Toulouse, France—B de Lafontan, J Mihura, H Roché.
Institut Curie, Paris, France—B Asselain, R J Salmon, J R Vilcoq.
Institut Gustave-Roussy, Paris, France—F André, R Arriagada, S Delaloge, C Hill, S Koscielny,S Michiels, C Rubino.
Institute of Cancer Research Clinical Trials and Statistics Unit (ICR-CTSU, NCRI), UK—R A'Hern, J Bliss, P Ellis, L Kilburn, J R Yarnold.
Integraal Kankercentrum, Amsterdam, Netherlands—J Benraadt, M Kooi, A O van de Velde, J A van Dongen, J B Vermorken.
International Breast Cancer Study Group (IBCSG), Bern, Switzerland—M Castiglione, A Coates, M Colleoni, J Collins, J Forbes, R D Gelber, A Goldhirsch, J Lindtner, K N Price, M M Regan, C M Rudenstam, H J Senn, B Thuerlimann.

International Collaborative Cancer Group, Charing Cross Hospital, London, UK—J M Bliss, C E D Chilvers, R C Coombes, E Hall, M Marty.
International Drug Development Institute, Louvain-la-Neuve, Belgium—M Buyse.
International TABLE Study Group, Berlin, Germany—K Possinger, P Schmid, M Untch, D Wallwiener.
ISD Cancer Clinical Trials Team (incorporating the former Scottish Cancer Therapy Network), Edinburgh, UK—L Foster, W D George, H J Stewart, P Stroner.
Israel NSABC, Tel Aviv, Israel—R Borovik, H Hayat, M J Inbar, T Peretz, E Robinson.
Istituto Nazionale per la Ricerca sul Cancro, Genova, Italy—P Bruzzi, L Del Mastro, P Pronzato, M R Sertoli, M Venturini.
Istituto Nazionale per lo Studio e la Cura dei Tumori, Milan, Italy—T Camerini, G De Palo, M G Di Mauro, F Formelli, P Valagussa.
Istituto Oncologico Romagnolo, Forli, Italy—D Amadori.
Italian Cooperative Chemo-Radio-Surgical Group, Bologna, Italy—A Martoni, F Pannuti.
Italian Oncology Group for Clinical Research (GOIRC), Parma, Italy— R Camisa, G Cocconi, A Colozza, R Passalacqua.
Japan Clinical Oncology Group–Breast Cancer Study Group, Matsuyama, Japan—K Aogi, S Takashima.
Japanese Foundation for Multidisciplinary Treatment of Cancer, Tokyo, Japan—O Abe, T Ikeda, K Inokuchi, K Kikuchi, K Sawa.
Kawasaki Medical School, Japan—H Sonoo.
Krakow Institute of Oncology, Poland—S Korzeniowski, J Skolyszewski.
Kumamoto University Group, Japan—M Ogawa, J Yamashita.
Leiden University Medical Center, Netherlands—E Bastiaannet, C J H van de Velde, W van de Water, J G H van Nes.
Leuven Akademisch Ziekenhuis, Gasthuisberg, Belgium—R Christiaens, P Neven, R Paridaens, W Van den Bogaert.
Ludwig-Maximilians University, Munich, Germany—S Braun.
Marseille Laboratoire de Cancérologie Biologique APM, France—P Martin, S Romain.
Medical University Vienna – General Hospital - Department of Obstetrics and Gynaecology and Department of Medicine I, Vienna, Austria—M Janauer, M Seifert, P Sevelda, C C Zielinski.
Memorial Sloan-Kettering Cancer Center, New York, NY, USA—T Hakes, C A Hudis, L Norton, R Wittes.
Metaxas Memorial Cancer Hospital, Athens, Greece—G Giokas, D Kondylis, B Lissaios.
Mexican National Medical Center, Mexico City, Mexico—R de la Huerta, M G Sainz.
National Cancer Institute, Bethesda, MD, USA—R Altemus, K Camphausen, K Cowan, D Danforth, A Lichter, M Lippman, J O'Shaughnessy, L J Pierce, S Steinberg, D Venzon, J A Zujewski.
National Cancer Institute of Bari, Italy—C D'Amico, M Lioce, A Paradiso.
NCIC Clinical Trials Group, Kingston, Ontario, Canada—J-A W Chapman, K Gelmon, P E Goss, M N Levine, R Meyer, W Parulekar, J L Pater, K I Pritchard, L E Shepherd, D Tu, T Whelan.
National Kyushu Cancer Center, Japan—Y Nomura, S Ohno.
National Surgical Adjuvant Breast and Bowel Project (NSABP), Pittsburgh, PA, USA—S Anderson, G Bass, A Brown (deceased), J Bryant (deceased), J Costantino, J Dignam, B Fisher, C Geyer, E P Mamounas, S Paik, C Redmond, S Swain, L Wickerham, N Wolmark.
Nolvadex Adjuvant Trial Organisation, London, UK—M Baum, I M Jackson (deceased), M K Palmer.
North Central Cancer Treatment Group, Mayo Clinic, Rochester, MN, USA—E Perez, J N Ingle, V J Suman.
North Sweden Breast Cancer Group, Umeå, Sweden—N O Bengtsson, S Emdin, H Jonsson.
North-West Oncology Group (GONO), Italy—L Del Mastro, M Venturini.
North-Western British Surgeons, Manchester, UK—J P Lythgoe, R Swindell.
Northwick Park Hospital, London, UK—M Kissin.
Norwegian Breast Cancer Group, Oslo, Norway—B Erikstein, E Hannisdal, A B Jacobsen, J E Varhaug.
Norwegian Radium Hospital, Oslo, Norway—B Erikstein, S Gundersen, M Hauer-Jensen, H Høst, A B Jacobsen, R Nissen-Meyer, K Reinertsen
Nottingham City Hospital, UK—R W Blamey, A K Mitchell, D A L Morgan, J F R Robertson.
Oita Prefectural Hospital, Japan—H Ueo.
Oncofrance, Paris, France—M Di Palma, G Mathé (deceased), J L Misset.
Ontario Clinical Oncology Group, Hamilton, Canada—M Levine, K I Pritchard, T Whelan.
Osaka City University, Japan—K Morimoto.
Osaka National Hospital, Japan—K Sawa, Y Takatsuka.
Oxford Radcliffe Hospitals NHS Trust, Churchill Hospital, Oxford, UK—E Crossley, A Harris, D Talbot, M Taylor.
Parma Hospital, Italy—G Cocconi, B di Blasio.

Petrov Research Institute of Oncology, St Petersburg, Russia—V Ivanov, R Paltuev, V Semiglazov.
Piedmont Oncology Association, Winston-Salem, NC, USA—J Brockschmidt, M R Cooper.
Pretoria University, South Africa—C I Falkson.
Royal Marsden NHS Trust, London and Sutton, UK—R A'Hern, M Dowsett, A Makris, M Parton, K Pennert, T J Powles, I E Smith, J R Yarnold.
St George's Hospital, London, UK—J C Gazet.
St George Hospital, Sydney, Australia—L Browne, P Graham.
St Luke's Hospital, Dublin, Ireland—N Corcoran.
Sardinia Oncology Hospital A Businico, Cagliari, Sardinia—N Deshpande, L di Martino.
SASIB International Trialists, Cape Town, South Africa—P Douglas, A Hacking, H Høst, A Lindtner, G Notter.
Saskatchewan Cancer Foundation, Regina, Canada—A J S Bryant, G H Ewing, L A Firth, J L Krushen-Kosloski.
Scandinavian Adjuvant Chemotherapy Study Group, Oslo, Norway—R Nissen-Meyer.
South Sweden Breast Cancer Group, Lund, Sweden—H Anderson, F Killander, P Malmström, L Rydén.
South-East Sweden Breast Cancer Group, Linköping, Sweden—L-G Arnesson, J Carstensen, M Dufmats, H Fohlin, B Nordenskjöld, M Söderberg.
South-Eastern Cancer Study Group and Alabama Breast Cancer Project, Birmingham, AL, USA—J T Carpenter.
Southampton Oncology Centre, UK—N Murray, G T Royle, P D Simmonds.
Southwest Oncology Group, San Antonio, TX, USA—K Albain, W Barlow, J Crowley, D Hayes, J Gralow, G Hortobagyi, R Livingston, S Martino, C K Osborne, P M Ravdin.
Stockholm Breast Cancer Study Group, Sweden—J Adolfsson, J Bergh, T Bondesson, F Celebioglu, K Dahlberg, T Fornander, I Fredriksson, J Frisell, E Göransson, M Iiristo, U Johansson, E Lenner, L Löfgren, P Nikolaidis, L Perbeck, S Rotstein, K Sandelin, L Skoog, G Svane, E af Trampe, C Wadström.
SUCCESS-Study Group, University of Düsseldorf, Germany—W Janni.
Swiss Group for Clinical Cancer Research (SAKK), Bern, and OSAKO, St Gallen, Switzerland—M Castiglione, A Goldhirsch, R Maibach, H J Senn, B Thürlimann.
Tampere University Hospital, Finland— M Hakama, K Holli, J Isola, K Rouhento, R Saaristo.
Tel Aviv University, Israel—H Brenner, A Hercbergs.
Tokyo Cancer Institute Hospital, Japan—M Yoshimoto.
Toronto-Edmonton Breast Cancer Study Group, Canada—A H G Paterson, K I Pritchard.
Toronto Princess Margaret Hospital, Canada—A Fyles, J W Meakin, T Panzarella, K I Pritchard.
Tunis Institut Salah Azaiz, Tunisia—J Bahi.
UK Multicentre Cancer Chemotherapy Study Group, London, UK—M Reid, M Spittle.
UK/ANZ DCIS Trial—H Bishop, N J Bundred, J Cuzick, I O Ellis, I S Fentiman, J F Forbes, S Forsyth, W D George, S E Pinder, I Sestak.
UK/Asia Collaborative Breast Cancer Group, London, UK—G P Deutsch, R Gray, D L W Kwong, V R Pai, R Peto, F Senanayake.
Unicancer Breast Group— A L Martin, H Roché.
University and Istituto Nazionale per la Ricerca sul Cancro, Genoa, Italy on behalf of GROCTA trialists—F Boccardo, A Rubagotti.
University College London, UK—M Baum, S Forsyth, A Hackshaw, J Houghton, J Ledermann, K Monson, JS Tobias.
University Federico II, Naples, Italy—C Carlomagno, M De Laurentiis, S De Placido.
University of Edinburgh, UK—L Williams.
University of Michigan, USA—D Hayes, L J Pierce.
University of Texas MD Anderson Cancer Center, Houston, TX, USA—K Broglio, A U Buzdar, L Hsu.
University of Wisconsin, USA—R R Love.
Uppsala-Örebro Breast Cancer Study Group, Sweden—J Ahlgren, H Garmo, L Holmberg, G Liljegren, H Lindman, F Wärnberg.
U.S. Oncology, Houston, USA—L Asmar, S E Jones.
West German Study Group (WSG), Germany—O Gluz, N Harbeck, C Liedtke, U Nitz.
West of Scotland Breast Trial Group, Glasgow, UK—A Litton.
West Sweden Breast Cancer Study Group, Gothenburg, Sweden—A Wallgren, P Karlsson, B K Linderholm.
Western Cancer Study Group, Torrance, CA, USA—R T Chlebowski.
Würzburg University, Germany—H Caffier
